# Entomological assessment of hessian fabric transfluthrin vapour emanators as a means to protect against outdoor-biting *Aedes* after providing them to households for routine use in Port-au-Prince, Haiti

**DOI:** 10.1371/journal.pone.0298919

**Published:** 2024-05-28

**Authors:** Chicoye Supreme, Obrillant Damus, Joseph Frederick, Jean-Frantz Lemoine, Christian Raccurt, Justin McBeath, Nosrat Mirzai, Sheila B. Ogoma, Vincent Corbel, Daniel Impoinvil, Gerry F. Killeen, Cyrille Czeher

**Affiliations:** 1 Université Quisqueya, Port-au-Prince, Republic of Haiti; 2 Programme National de Contrôle de la Malaria, Ministère de la Santé Publique et de la Population, Port-au-Prince, Republic of Haiti; 3 Envu UK Ltd, Cambridge, Milton, Cambridge, United Kingdom; 4 Institute of Biodiversity, Animal Health and Comparative Medicine, University of Glasgow, Glasgow, United Kingdom; 5 Abt Associates, Nairobi, Kenya; 6 Institut de Recherche pour le Developpement, University of Montpellier, Montpellier, France; 7 Laboratório de Fisiologia e Controle de Artrópodes Vetores (Laficave), Instituto Oswaldo Cruz (IOC), Fundação Oswaldo Cruz (FIOCRUZ), Rio de Janeiro, RJ, Brazil; 8 Centers for Disease Control and Prevention, Atlanta, Georgia, United States of America; 9 Ifakara Health Institute, Ifakara, Morogoro, United Republic of Tanzania; 10 Liverpool School of Tropical Medicine, Department of Vector Biology, Liverpool, United Kingdom; 11 School of Biological Earth & Environmental Sciences, Environmental Research Institute, University College Cork, Cork, Republic of Ireland; 12 Entente Interdépartementale pour la Démoustication du Littoral Méditerranéen (EID Méditerranée), Montpellier, France; University of Glasgow College of Medical Veterinary and Life Sciences, UNITED KINGDOM

## Abstract

**Background:**

A simple treated fabric device for passively emanating the volatile pyrethroid transfluthrin was recently developed in Tanzania that protected against nocturnal *Anopheles* and *Culex* mosquitoes for several months. Here these transfluthrin emanators were assessed in Port-au-Prince, Haiti against outdoor-biting *Aedes*.

**Methods:**

Transfluthrin emanators were distributed to participating households in poor-to-middle class urban neighbourhoods and evaluated once every two months in terms of their effects on human landing rates of wild *Aedes* populations. A series of three such entomological assessment experiments were conducted, to examine the influence of changing weather conditions, various transfluthrin formulations and emanator placement on protective efficacy measurements. Laboratory experiments assessed resistance of local *Aedes aegypti* to transfluthrin and deltamethrin, and the irritancy and repellency of the transfluthrin-treated fabric used in the field.

**Results:**

Across all three entomological field assessments, little evidence of protection against wild *Ae*. *aegypti* was observed, regardless of weather conditions, transfluthrin formulation or emanator placement: A generalized linear mixed model fitted to the pooled data from all three assessment rounds (921 females caught over 5129 hours) estimated a relative landing rate [95% Confidence interval] of 0.87 [0.73, 1.04] for users of treated versus untreated emanators (P = 0.1241). Wild *Ae*. *aegypti* in this setting were clearly resistant to transfluthrin when compared to a fully susceptible colony.

**Conclusions:**

Transfluthrin emanators had little if any apparent effect upon *Aedes* landing rates by wild *Ae*. *aegypti* in urban Haiti, and similar results have been obtained by comparable studies in Tanzania, Brazil and Peru. In stark contrast, however, parallel sociological assessments of perspectives among these same end-users in urban Haitian communities indicate strong satisfaction in terms of perceived protection against mosquitoes. It remains unclear why the results obtained from these complementary entomological and sociological assessments in Haiti differ so much, as do those from a similar set of studies in Brazil. It is encouraging, however, that similar contrasts between the entomological and epidemiological results of a recent large-scale assessment of another transfluthrin emanator product in Peru, which indicate they provide useful protection against *Aedes*-borne arboviral infections, despite apparently providing only modest protection against *Aedes* mosquito bites.

## Background

The *Aedes* (*Stegomia*) mosquitoes that mediate most transmission of Dengue, Chikungunya, Yellow Fever and Zika viruses often attack people during daylight hours when they are awake and active, often outdoors, so there are limits to how much protection may be reasonably expected from indoor interventions [1, 2] like insecticidal bed nets that protect sleeping spaces [3] or even insecticidal screens that protect entire houses [4]. However, a recent large-scale trial of a spatial repellent product that emanates vapour of the volatile pyrethroid transfluthrin to designed to protect users in outdoor spaces and open structures successfully demonstrated that such devices may reduce incidence of arboviral infections [5]. Unfortunately, these devices and other existing repellent products currently available on the market only protect against mosquitoes for hours, days or weeks per application or dispensing dose, so they may be too expensive and impractical for continuous, indefinite use in low-income countries like Haiti [1, 6], and some formulations may even be hazardous [7, 8].

However, a low-technology transfluthrin emanator, which slowly and passively releases vapour of this volatile pyrethroid under ambient temperature conditions without any electricity or other power source, was recently developed in Tanzania [9] that provided >90% protection for >4 months against nocturnal *Anopheles* and *Culex* spp. vectors of malaria, filariasis and several arboviruses in urban Dar es Salaam [10]. In a subsequent study in rural Tanzania, >75% protection was sustained over 6 months and at least some degree of protection persisted over 2.5 years without any evidence of diversion to non-users [11]. Also, equivalent efficacy was achieved over 6 months with a 10-fold lower transfluthrin dosage, which costs only €0.10 and releases vapour concentrations of only 0.00013 mg/m^3^ [11], comparing well with its registered acceptable exposure concentration of 0.5 mg/m^3^ [12]. While the initial prototype was suspended on four poles placed around the user a more practical format has now been developed that is completely mobile and can be conveniently placed anywhere the user chooses to [9, 10].

If these transfluthrin emanator devices were to prove as effective against day-biting *Aedes* as they are against night-biting *Culex* and *Anopheles*, they could offer simultaneous, broad-spectrum daytime protection against Dengue, Chikungunya, Yellow Fever and Zika. The following series of studies was therefore carried out in Port-au-Prince, Haiti, to measure the extent and duration of entomologically measured protective efficacy of transfluthrin emanators against outdoor-biting *Aedes* under normal conditions of routine community use, as well as *Culex quinquefasciatus* feeding outdoors and indoors. Parallel social science studies to evaluate the perceived effectiveness and user acceptability of transfluthrin emanators are reported elsewhere in a complementary manuscript [13].

## Methods

### Field site and study design

**Fig 1:**
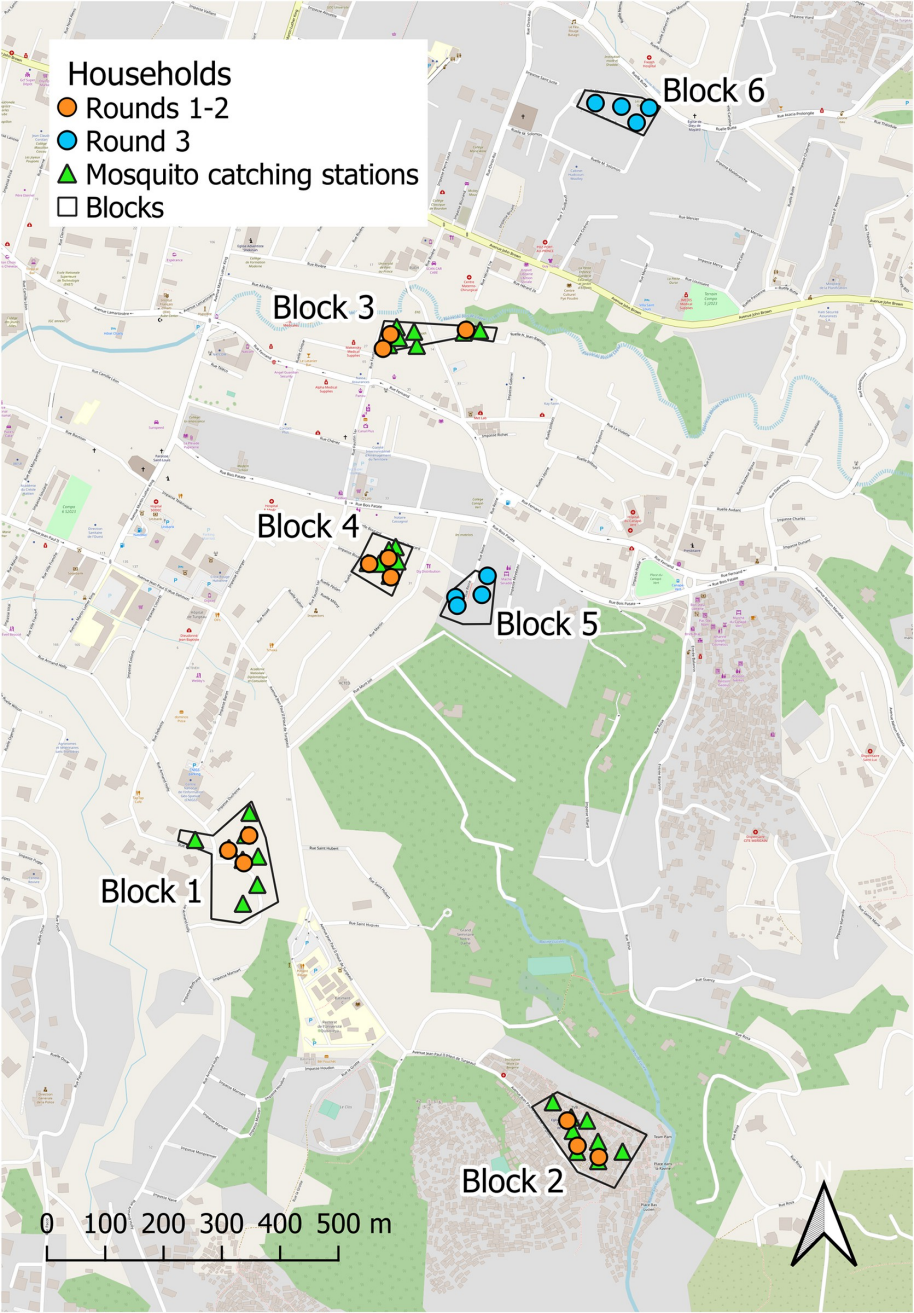
A map of the study site in the Haut-Turgeau neighbourhood in the city of Port-au-Prince, Haiti, illustrating the four clusters of three households where the three rounds of quantitative entomological assessments for transfluthrin emanators reported herein were carried out. Note that the three different assessment rounds otherwise differed only in that the emanators were treated with different formulations of transfluthrin and slightly different experimental procedures were used to assess their efficacy in entomological terms (Figs 2 and 3). The parallel qualitative social science assessments of user-perceived efficacy reported elsewhere [13] were conducted in the same four clusters as the entomological assessments reported herein for the first two assessment rounds. Note, however, that they were carried out in two geographically separate clusters of four households for the third assessment round. This separation of the entomological and social science assessments was intended to minimize risk of community perspectives being unduly influenced by competing financial interests (See *Ethical Considerations*) or by discussions with the entomological research team during the regular monitoring visits necessitated by those procedures [13]. This map was produced with *QGIS*® version 3.28.9 open source software, using a base map obtained from *OpenStreetMap*® under the Open Database License.

All procedures for this study, together with the complementary social science assessments of end user perceptions in these same Haitian communities [13], and a similar entomological assessment of transfluthrin emanator efficacy in Tanzania [14], both of which were carried out in parallel with this study, are provided as supporting information in S1–S3 Protocols. These simple transfluthrin emanators were distributed to participating households in poor-to-middle class urban neighbourhoods of Haut-Turgeau in the city of Port-au-Prince, Haiti (Fig 1) and evaluated as described herein, in terms their effects on landing rates of mosquitoes upon human users under experimentally controlled conditions. In addition to the quantitative entomological assessments reported herein, parallel qualitative social science surveys were conducted among community end-users to gather complementary data. The details of these surveys are reported elsewhere [13], revealing generally encouraging perceptions among community end-users with respect to the efficacy, safety and utility of these transfluthrin emanator devices.

**Fig 2:**
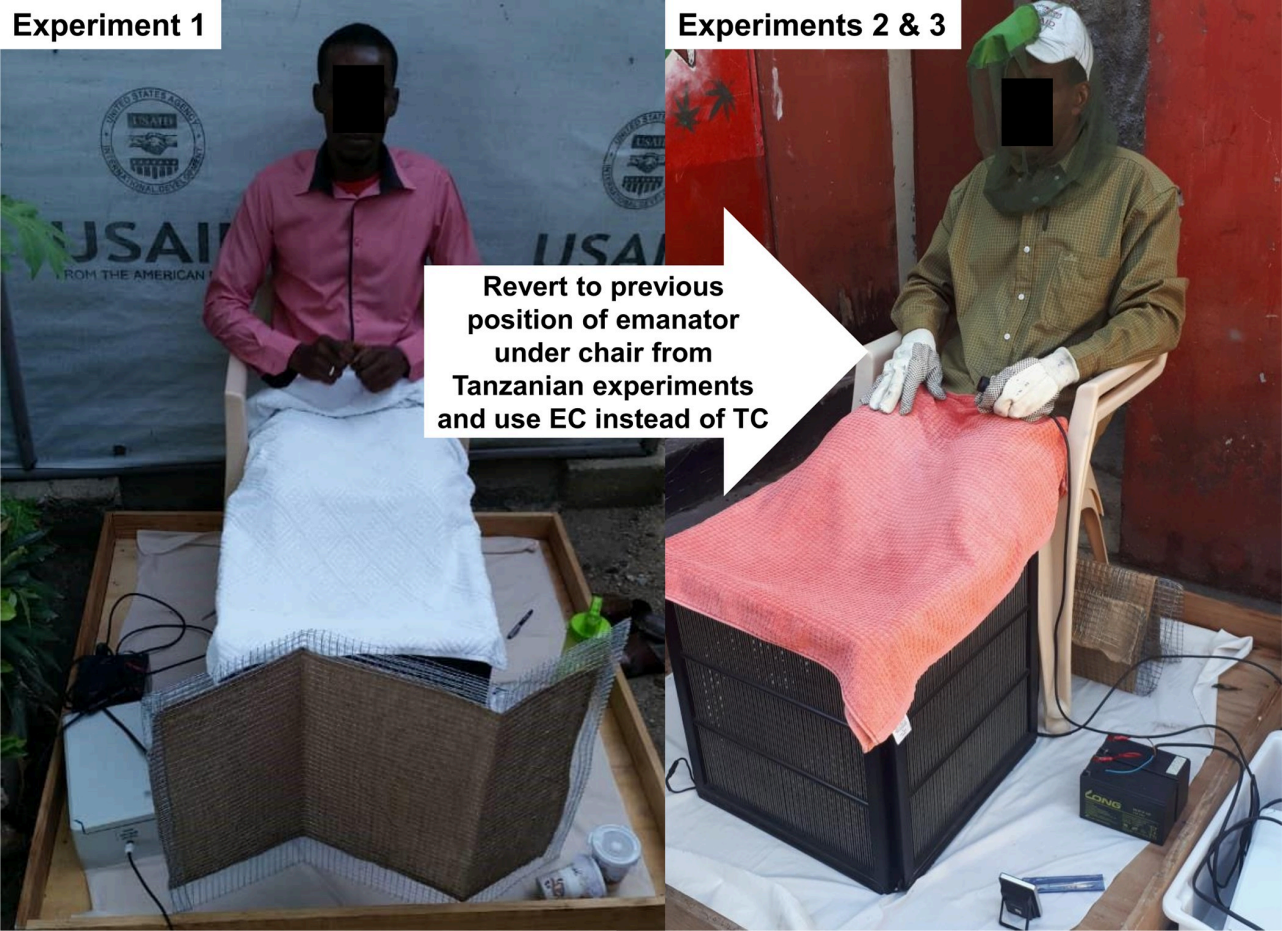
The experimental setup used to assess transfluthrin emanator effects on landing rates of outdoor-biting mosquitoes using Mosquito Electrocuting Traps (METs) [15–19]. This schematic illustrates how the arrangements of the emanator devices varied in terms placement of the emanators relative to the human user (Fig 2), as well as the choice of transfluthrin formulation (Emulsifiable concentrate (EC) versus technical concentrate (TC)) used to treat them. The Tanzanian studies referred herein to are described in detail in reference [14].

**Fig 3:**
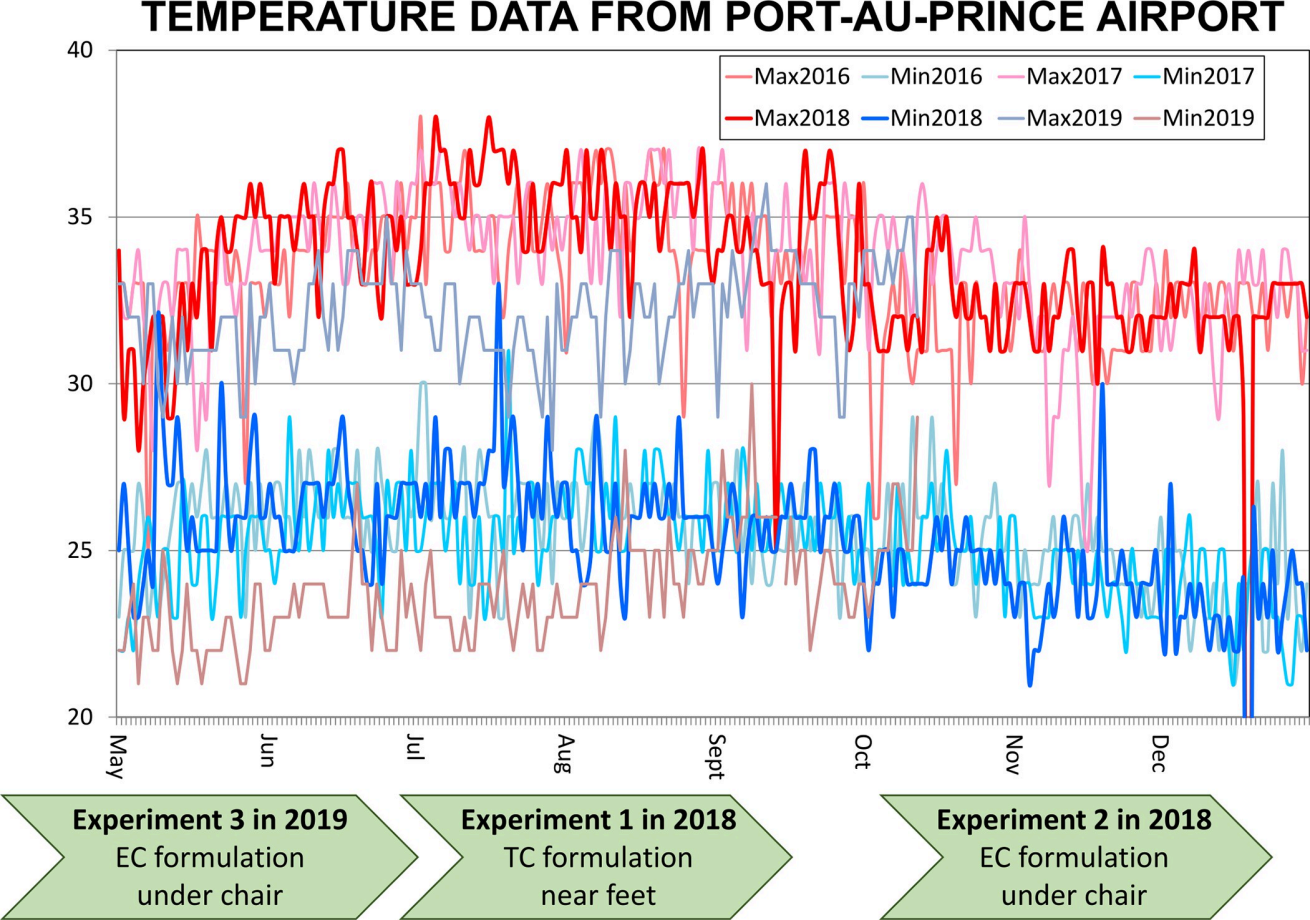
A schematic outline of how the three entomological efficacy assessment experiments described herein fitted into typical seasonal temperature trends in Port-au-Prince, Haiti. This schematic also illustrates how these three experiments differed from each other in terms of placement of the emanators relative to the human user (Fig 2), as well as the choice of transfluthrin formulation (Emulsifiable concentrate (EC) versus technical concentrate (TC)) used to treat them.

Each of the household clusters illustrated in Fig 1 consisted of the first four consenting households (See *Ethical considerations*) that could be identified by door-to-door convenience sampling, starting from a central point within that neighbourhood. All engagements with community members in these neighbourhoods of Port-au-Prince, including the formal social science surveys reported elsewhere [13], were carried out in fluent Haitian Creole by resident team members for whom this was their first language. While some trivial deviations from the following experimental procedures occurred in practice during implementation for practical reasons (eg. households withdrawing from the study or some household clusters omitted for safety reasons during periods of civil unrest), these slight variations in procedures were all minor and had no obvious implications for the interpretation of the results.

The original intention had been to carry out these quantitative entomological assessments, and the complementary qualitative assessments of community end-user perceptions [13], only once. As detailed below, however, the former entomological assessments yielded no evidence of significant protection in terms of reduced human landing rates at the first attempt. Both types of assessment were therefore repeated twice, with minor procedural variations to determine whether changing the transfluthrin formulation used or the position of the emanator relative to the user improved the levels of efficacy observed based on quantitative entomological indicators (Figs 2 and 3).

### Formulation of transfluthrin treated strips

Panels of hessian fabric, each measuring 70 × 40 cm, were made from jute rolls bought locally and then washed, dried and treated with 99% technical grade transfluthrin (Bayer AG, Environmental Sciences at the time, now trading as Envu AG, Germany) as follows. For each hessian panel, either a mixture of 3g of transfluthrin technical concentrate (TC) and 90ml of locally available liquid dish washing detergent (Apta Vaisselle, Intermarché), or the equivalent amount of active ingredient in emulsifiable concentrate (EC) form, were mixed with 400ml of water and then soaked into a the panel as evenly as possible, as previously described [11]. Each panel was then left to dry at room temperature indoors, for between two days and a week, before being distributed. Control panels were also soaked into similar mixtures of water and detergent but without transfluthrin, to create a suitable set of control devices with which the transfluthrin-treated panels could be compared. Before being distributed, the dried strips were each wrapped within a wire-mesh to form a folded, zig-zag-shaped, self-supporting emanator (Fig 2), essential identical to that similarly evaluated against *Ae*. *aegypti* in Tanzania [14]. The plastic-coated wire-mesh cover was designed to prevent dermal contact of participants and researchers with the treated hessian panels. It was also designed to provide enough rigidity, but also enough flexibility, to allow the devices to be folded into self-standing shapes like the cylindrical and zig-zag prototypes (Fig 2).

### Provision of transfluthrin emanators and usage guidance to households

**Fig 4:**
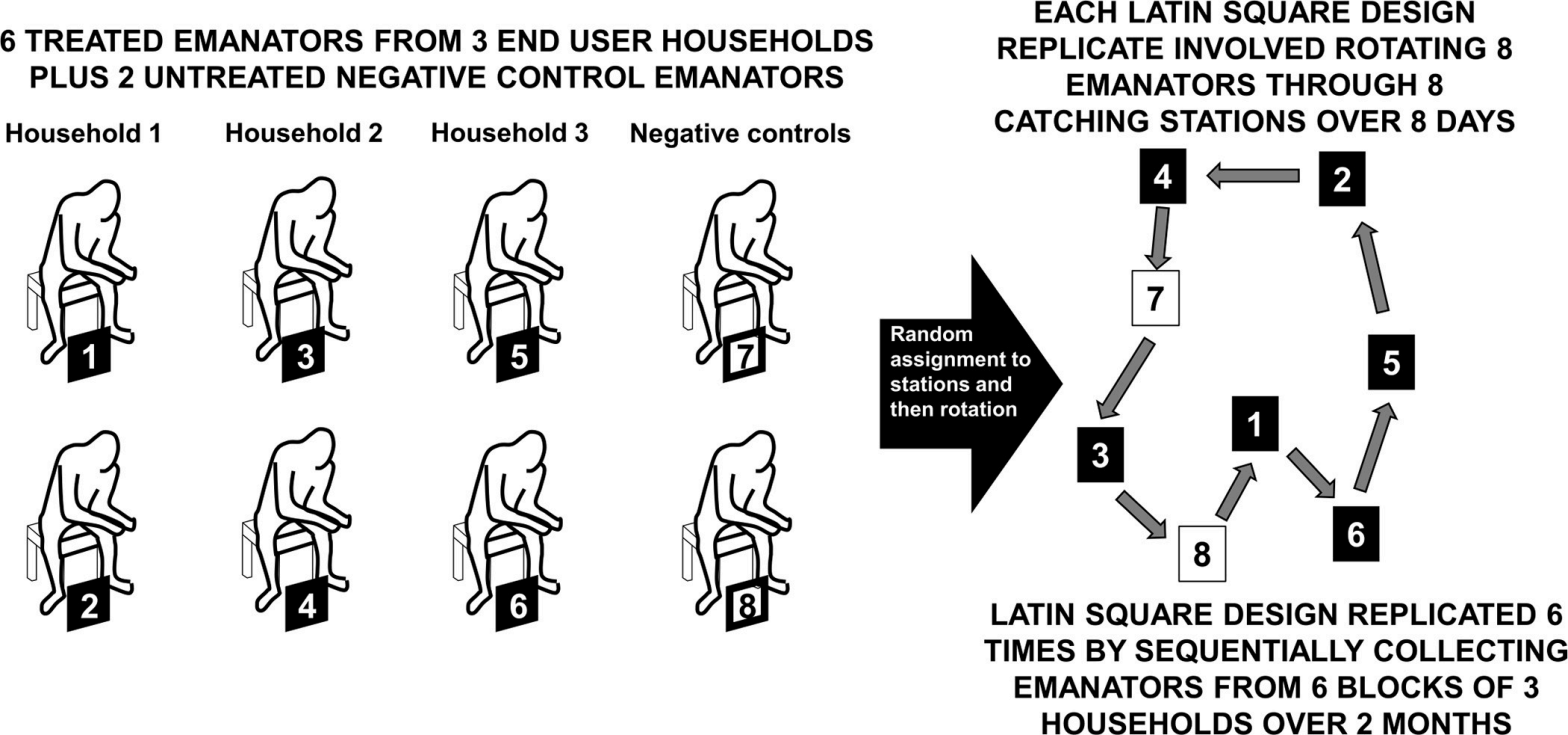
A schematic illustration of the experimental design used for entomological assessment of transfluthrin emanators using Mosquito Electrocuting Traps (METs) [15–19] (Fig 2) as a means of protection against *Aedes aegypti*, *Ae*. *albopictus* and other human biting mosquitoes.

Each participating household was provided with 2 freshly prepared transfluthrin emanators at the outset of an experiment, to be used freely by the householders following advisory discussions with the research team on how to safely and effectively deploy them. Specifically, they were advised that they were free to use the emanators in whatever way they perceive to be the most convenient and effective, so long as they did not open the protective holder or use it in any other way that would allow direct physical contact with the treated fabric inside. All treated emanators provided to households were taken back from them for entomological evaluations of their efficacy for only 8 days (Fig 4) every two months. During brief periods of transport between the residences of the community end-users and the test sites where they were evaluated under controlled conditions in entomological terms, the devices were fully shaded inside black plastic bags to protect them against the sun. Note that the two untreated emanators used to complete the experimentally controlled component of the assessment study design (Fig 4) were never provided to community members and were instead stored separately from any treated emanators when they were not in use.

For the remainder of each 2-month evaluation cycle, when they were not being assessed through controlled entomological experiments, the emanators were used freely within the bounds of the safety instructions provided. Participants were actively encouraged to use them creatively, in whatever way they perceived to be optimal in terms of convenience and protection against mosquito bites, so long as they did not open the protective holder or use them in any way that would allow direct physical contact with the treated fabric inside it. As an illustrative example, the research team explained how one investigator placed such a device beside the front door of his house at night to prevent house entry by *Culex* mosquitoes [11].

### Initial protocol for measuring the effects of transfluthrin emanators on outdoor landing rates of mosquitoes

The effects of transfluthrin-treated emanators upon human landing rates were measured with recently developed Mosquito Electrocuting Traps (METs), which were originally developed for night-biting *Anopheles* and *Culex* in East Africa [15, 16, 18] but have also proven useful for day-biting *Aedes* in both East Africa [19] and Latin America [17]. METs were placed around the feet of volunteers who were fully protected against mosquito bites with protective clothing and headgear (Fig 2), similarly to a parallel study in Tanzania where these traps were designed [14]. Only adult males (≥18 years) and adult females of non-child-bearing age (≥50 years) were recruited as volunteers, to comprehensively avoid any risks associated with infection with Zika or any other vector borne pathogen to which pregnant women are particularly vulnerable. All mosquitoes captured by each volunteer over each hour spent sitting in a MET were placed in a separate labelled paper cups which were coved on top by a piece of nets to prevent mosquito from escaping. After the morning shift of experiments, mosquitoes in each paper cups were first killed by using ethanol, and then sorted, counted and morphologically identified to genus level and classified by sex and abdominal status (Unfed, part fed, fully fed or gravid). For reasons explained in the *Results and Discussion* section, the third entomological assessment experiment supplemented these MET measurements of landing (presumably host-seeking) mosquitoes with prokopak aspirator collections of resting mosquitoes [20].

Eight catching stations 15 to 50 meters apart from each other were identified within each replicate block, each of which was located in a different part of the study area (Fig 1) to capture a diversity of environmental conditions. Catching stations were established in peri-domestic areas amongst houses, to maximize mosquito density because, in our experience and consistent with reports from Brazil [21, 22], *Aedes aegypti* thrive in these micro-environments and don’t fly far from them. Note also, however, that locations of these catching stations were chosen to minimize disturbance of the residents or accidental contact with the METs, particularly children and livestock.

In the first and second entomological assessment experiments, each day of work comprised the same sequence of 6 one-hour collection periods, with 3 being in the morning (6:00 to 7:00, 7:00 to 08:00 and 08:00 to 09:00) and 3 in the evening (16:00 to 17:00, 17:00 to 18:00 and 18:00 to 19:00), to match the known diurnal but crepuscular activity patterns of *Ae*. *aegypti* [17]. In order to average out potential biases arising from the prevailing directions of wind and sunshine, each of these one-hour periods for a given day was randomly allocated without replacement to one of 6 angles (0°, 60°, 120°, 180°, 240° and 300°, relative to North), which was the same for all 8 catching stations for that day and hour-long period. Each hour, the chair of the catcher and the MET [15–19] he or she used was rotated together around the centre of the catching station to face in that particular direction, so that all possible orientations relative to wind direction (measured with a miniature weather station placed nearby) were represented.

A replicated Latin square design was used for the entomological efficacy evaluation of the 3 pairs of treated emanators distributed to each of the 3 participating households (2 per household, 6 in total) in each experimental block. In each experimental block, each of which was matched to a specific housing cluster where 6 emanators were distributed to 3 households in the community, mosquito collections were conducted over a series of 8 continuous days once every 2 months. One complete replicate of the experimental design was completed in each block of 8 catching stations by rotating all 8 emanators (the 6 treated emanators used by the householders plus 2 negative controls treated with detergent and water only) through all 8 stations in a random order over the course of 8 days (Fig 4). Eight human volunteers assigned one of the 8 treated or untreated emanators and collected mosquitoes with METs [15–19] while using the emanator (Fig 2) assigned to them for that day (Fig 4). Each volunteer was allocated to a single, fixed catching station within the block for the duration of each replicate, so that the two sources of variation in capture rate associated with station and volunteer could be combined into a single source of variance captured with a single random effect and maximum statistical power in the analysis.

The 8×8 Latin square design described above was repeated 6 times for a single replicate in a single block of 8 catching stations, by repeating it in 6 distinct blocks in different parts of the field site, where all 18 participating households were each provided with 2 freshly prepared transfluthrin emanators at the outset of the experiment. Overall, one full round of this experimental design took 48 days of field work (8 days per block and rotation replicate × 6 blocks and replicates) that was distributed across a working period of 2 months, to allow personnel time to rest and attend to other commitments. These two-month rounds of evaluation were repeated up to three times over experimental periods of up to six months. Note that the 6 treated emanators distributed to household in each block were used for only 8 days of every two-month round of experimental entomological assessment. For the remainder of each 2-month evaluation cycle, the emanators were used freely by the households to whom they were given.

### Subsequent repetition and readjustment of the emanator evaluation protocol

The first attempt to evaluate the transfluthrin emanators (Experimental assessment round 1) yielded no evidence of significant protection against outdoor-biting *Ae*. *aegypti* (See *Results*), contrasting starkly with the encouraging perspectives shared by community end-users during parallel sociological assessments of their perceived effectiveness [13]. This evaluation procedure was therefore repeated from scratch twice thereafter, with changes made to the transfluthrin formulation used, the positioning of the emanator and the time of year over which each round of experimental assessment was carried out (Figs 2 and 3).

A particularly notable limitation of entomological experiment 2, and one which motivated one more repetition of the overall assessment protocol from scratch, was that it was conducted in the relatively cool months of the winter. Even though temperatures were nevertheless remarkably warm in the Caribbean at that time (Fig 3), and comparable with those at which transfluthrin had previously proven efficacious in Tanzania [9–11, 23, 24], it was considered prudent to repeat the assessment of this new emulsifiable concentrate (EC) formulation with the emanator device placed under the chair (Fig 2) during the warmer months of summer (Fig 3).

This third entomological assessment of efficacy (Experiment 3) was also complemented by a parallel repetition of the sociological assessments of perceived effectiveness [13]. However, this third set of social science investigations were conducted in separate housing clusters from the third set of entomological assessments (Fig 1), to reduce risk of bias arising from competing interests amongst participants caused by the generous renumeration associated with the latter (See *Ethical Considerations* and reference [13]).

Also, the complementary sociological investigations of community end-user perspectives indicated that the emanators were perceived to be most effective indoors at night by several participants [13], which suggested to the investigators that they might be more effective against nocturnal, endophilic *Culex quinquefasciatus* than against *Aedes aegypti*. Some informal discussions with participants outside these formal sociological studies also suggested some users were actually targeting mosquitoes while they rest indoors, rather than when they attempt to land and bite [13]. Consequently, the third round of entomological assessments (Experiment 3) collected human landing mosquitoes indoors as well as outdoors and shifted the the 6-hour time window for mosquito collection to either side of dusk (16:00 to 17:00, 17:00 to 18:00, 18:00 to 19:00, 19:00 to 20:00, 20:00 to 21:00 and 21:00 to 22:00).

### Protective efficacy field data management and analysis

All the data obtained from the field efficacy assessments (S1 Data) were entered into a pre-designed paper-based data collection form, and then entered, cleaned and linked using a standardized entomological data informatics system as previously described [25]. Generalized linear mixed models (GLMMs) were initially fitted to each subset of data comprising the first full two-month experimental replicate of the study design, before additional GLMMs were fitted to the full longitudinal datasets, with and without a term for time since treatment to allow for any longitudinal trends in protective efficacy. Models assuming simple Poisson distributions for the mosquito count outcomes were initially assessed with and without observation effects. However, the final reported models giving the best fit to the combined data from all 3 rounds of experimental assessment assumed negative binomial distributions for this dependent variable (AIC = 4887 versus 5018 for the equivalent Poisson model with an observation random effect and 5094 without it (P << 0.0001 in both cases), as per S1 File). All final reported models, fitted to either each separate round of experimental assessment, or to the pooled data from all three, accounted for the effects of spatiotemporal variations in mosquito density by including date, time of day and station within block as separate random effects (S1 File).

### Surveying resistance of the field population of *Aedes aegypti* to transfluthrin and deltamethrin

Resistance status of mosquitoes to transfluthrin was assessed using the CDC bottle bioassay [26] and a diagnostic dosage of 3μg of transfluthrin per bottle for *Aedes aegypti* [27]. Dose-response curves were also established in Haiti using wild specimens collected as larvae from the study blocks of Debussy (Block 1) and Pedant (Block 4) and compared to results obtained at the laboratories of the Institut de Recherche pour le Developpement (IRD) in Montpellier using a fully insecticide susceptible *Ae*. *aegypti* laboratory colony originating from French Polynesia (Bora Bora strain), in order to evaluate their level of resistance. Bottle tests from February were done on mixed specimens from both blocks due to low numbers, while tests from June were done on specimens from Debussy only. Even when collections were made in separate blocks, these were considered to represent a single population because the distance between breeding sites in the two blocks did not exceed 750m. Deltamethrin resistance phenotypes for the same mosquito batches were determined using standard World Health Organization (WHO) tube assays [26] and diagnostic concentration of 0.05% deltamethrin on impregnated papers, so that their responses to transfluthrin could be interpreted in the context of their observed resistance to more conventional solid-phased pyrethroids.

### Assays for the contact irritancy and spatial repellency of transfluthrin-treated hessian

The insecticide susceptible *Ae*. *aegypti* Bora Bora colony maintained at IRD Montpellier was also used for laboratory assessment of the contact irritancy and spatial repellency effects of the transfluthrin-treated hessian used in Haiti, using the high-throughput screening system (HITSS) developed by Grieco *et al*. [28, 29]. Only one concentration of transfluthrin per square meter of hessian was used (5.14 g/m^2^, i.e. same application rate used for the field evaluation), and two different fabric sizes were tested in order to study how both irritancy and spatial repellency are affected by varying quantities of transfluthrin vapor within the HITSS. The hessian samples were treated in Haiti at the end of February 2019 in the same way as for all emanators used in the field study. They were stored in sealed plastic bags at 4°C in the dark between experiments, so that they could be considered freshly treated and with maximum efficacy throughout the tests carried out in March and May 2019. All results were expressed as the mean proportion that were knocked down or died.

In the contact irritancy assay, female mosquitoes were introduced at the end of the treated chamber (holding the treated hessian on the inner side) and given 10 minutes in the dark to escape and rest inside the untreated chamber. The HITSS apparatus was fitted either with a full-size rectangle of hessian (10 × 29 cm) covering the whole internal surface of the chamber, or a strip of 1 × 29cm (1/10^th^ of the size of a full panel) fixed at the side of the treated chamber. The same tests (at least 8 replicates per condition) were repeated at two different temperatures to evaluate whether 2 to 3°C differences could modify the contact irritancy and spatial repellency observed using the HITSS experimental set up.

The spatial repellency assays used the same modular HITSS system but with a third compartment: clear untreated cylinder in the middle where females are introduced, control chamber on one side, treated chamber on the other side. This test allows to determine if a particular substance / concentration act from a distance as spatial repellent or attractant, and to estimate the resulting spatial activity index. All mortality, contact irritancy and spatial activity outcomes were calculated from the number of mosquitoes found in the different chambers at the end of each exposure period, as detailed by Grieco *et al*. [28, 29].

### Ethical considerations

The procedures for this study were reviewed and approved by the Comité Nationale de Bioéthique of the Ministère de la Santé Publique et de la Population of the Republic of Haiti (Ref. 1718–42) and the Research Ethics Committee of the Liverpool School of Tropical Medicine in the United Kingdom (Ref. 16–037).

At the outset of the study, the concentrations of tranfluthrin vapour released by these emanator devices had previously been measured as only 0.00013 mg/m^3^ [11], which compares very well (<1/1000th) with its registered acceptable exposure concentration of 0.5 mg/m^3^ for the European Union [12]. Inhalation exposure to transfluthrin was therefore considered to present negligible risk to participants at the outset of this study.

The MET device is designed to kill mosquitoes before they can bite, so human volunteers sitting within it are not exposed to increased risk of mosquito-borne infections [15–19]. Each participant in mosquito landing catches sat on a chair with his or her legs protected within the square plastic frame of the MET, while the rest of body was protected from mosquito bites by a wearing hat with a netting curtain, a long sleeve shirt and gloves (Right hand panel of Fig 2). From within the square PVC/wooden frame is lined up with insulating plastic fiber mesh which serves not only for protection of mosquito entry, but also prevent volunteer’s limbs from making contact with the exterior electrified wires of the MET device [15–19]. Furthermore, only adult males (≥18 years) and adult females of non-child-bearing age (≥50 years) were recruited as participants in mosquito landing catches, to comprehensively avoid any risk of infection with Zika, malaria or any other vector borne pathogen to which pregnant women are particularly vulnerable.

Participants in the study were recruited between May 2018 and February 2019. All participants in this study were fully informed of these potential risks and benefits of participation in the study, as well as their freedom to withdraw at any stage, and were given every opportunity to ask any questions they had before informed consent was documented in writing. No personal information was collected from any participants, other than their names as recorded on the informed consent forms, all of which were stored in locked filing cabinets. Although several of the investigator knew the participants by name and could therefore identify them as individuals in the datasets based on their recorded initials, none of the data provided in S1 Data can be linked to any individual by any other person. Overall, no personally identifiable data or images are presented in this publication and written consent has been obtained from both individuals depicted in Fig 2.

The remuneration rate of $15 per day offered to participants in mosquito landing catches with METs [15–19] had been standardized across all PNCM activities at the time, to strike a balance between being enough to provide fair compensation for time and discomfort, without inducing volunteers to participate despite any reservations they may have. Nevertheless, this represented a significant amount of money in this low-income context, raising the possibility that community perspectives might be unduly influenced by competing financial interests and/or discussions with the entomological research team during the regular visits necessitated by those procedures [13]. For the third and final round assessments, the entomological assessments reported herein and the social science investigations reported elsewhere [13] were completely separated and carried out in distinct housing clusters (Fig 1).

## Results

**Fig 5:**
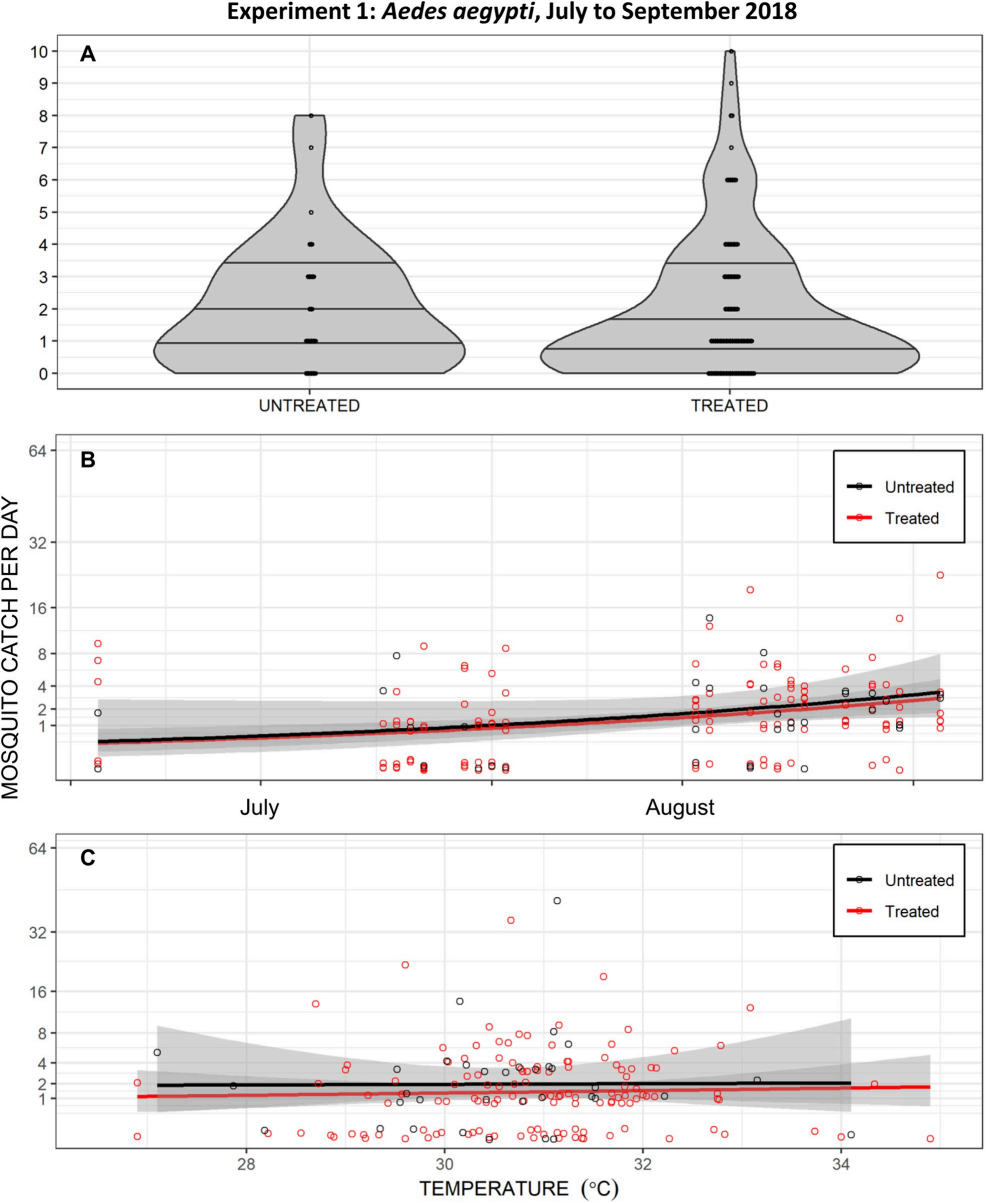
Daily *Aedes aeg*y*pti* landing catches from the first entomological experiment to compare transfluthrin-treated emanators with untreated negative control (placebo) emanators, carried out in in Port-au-Prince from July to September 2018. In this first experiment, emanators were treated with 3g of the technical concentrate (TC) formulation emulsified with liquid dishwashing detergent [9–11] and placed in front of the chairs of users (Left-hand panel of Fig 2) sitting outdoors. **A**: Outdoor catches with treated and untreated emanators presented as separate violin plots of density distribution with the first quartile, median and third quartile indicated by three horizontal lines and overlain by a dot plot of the individual daily total catch observations. Note that three times as many landing catches were carried out on users of treated emanators than untreated emanators (Fig 4), so the width of the probability density violin graphs may be directly compared in absolute terms but not those of the dot plots. **B**: Presented as a longitudinal time course, with separate longitudinal trends for the treated and untreated emanators over time estimated and plotted using the *geom_smooth* function of the *ggplot2* package in R, specifying the general linear model (*glm*) method with time as the independent variable and mosquito catch as the dependent variable with a Poisson distribution. **C**: Presented as a function of daily mean temperature, with separate trends for the treated and untreated emanators with temperature variations estimated and plotted using the *geom_smooth* function of the *ggplot2* package in R, specifying the *glm* method with temperature as the independent variable and mosquito catch as the dependent variable with a Poisson distribution.

As illustrated in Fig 5, the first round of entomological evaluations carried out in the middle of the Haitian summer yielded little evidence of protection against *Aedes aegypti*. Based on 437 females caught over a total of 985 hours of collection, the best fit GLMM indicated no statistically significant reduction in mosquito landing rates (Relative rate (RR) of mosquito landing upon users of treated versus untreated emanators [95% confidence intervals (CI)] = 0.85 [0.63, 1.14], z = -1.092, P = 0.275). Graphical inspection of the distribution of the pooled data reveals no obvious difference between landing rates on volunteers using emanators treated with 3g of transfluthrin TC and those using untreated devices within 6 weeks of treatment (Fig 5A). Examining these same data as a function of time (Fig 5B) or ambient temperature (Fig 5C) also indicates negligible differences between treated and untreated emanators and suggests no confounders or other obvious alternative explanation for the apparent lack of protective efficacy observed. Fig 5C is particularly informative because it reveals no treatment-dependent effect of temperature on mosquito catches.

In this first entomological experiment to assess transfluthrin emanators (Fig 5), the devices were treated with the TC formulation emulsified with liquid dishwashing detergent [9, 10, 23] and placed in front of the legs of the user, so the two subsequent experiments instead used an EC formulation and placed the device under the chair of the users (Figs 2 and 3). They were also carried out at different times of the year, with experimental assessment 2 being conducted in the Haitian winter while experiment 3 extended from late spring to early summer (Fig 3).

**Fig 6:**
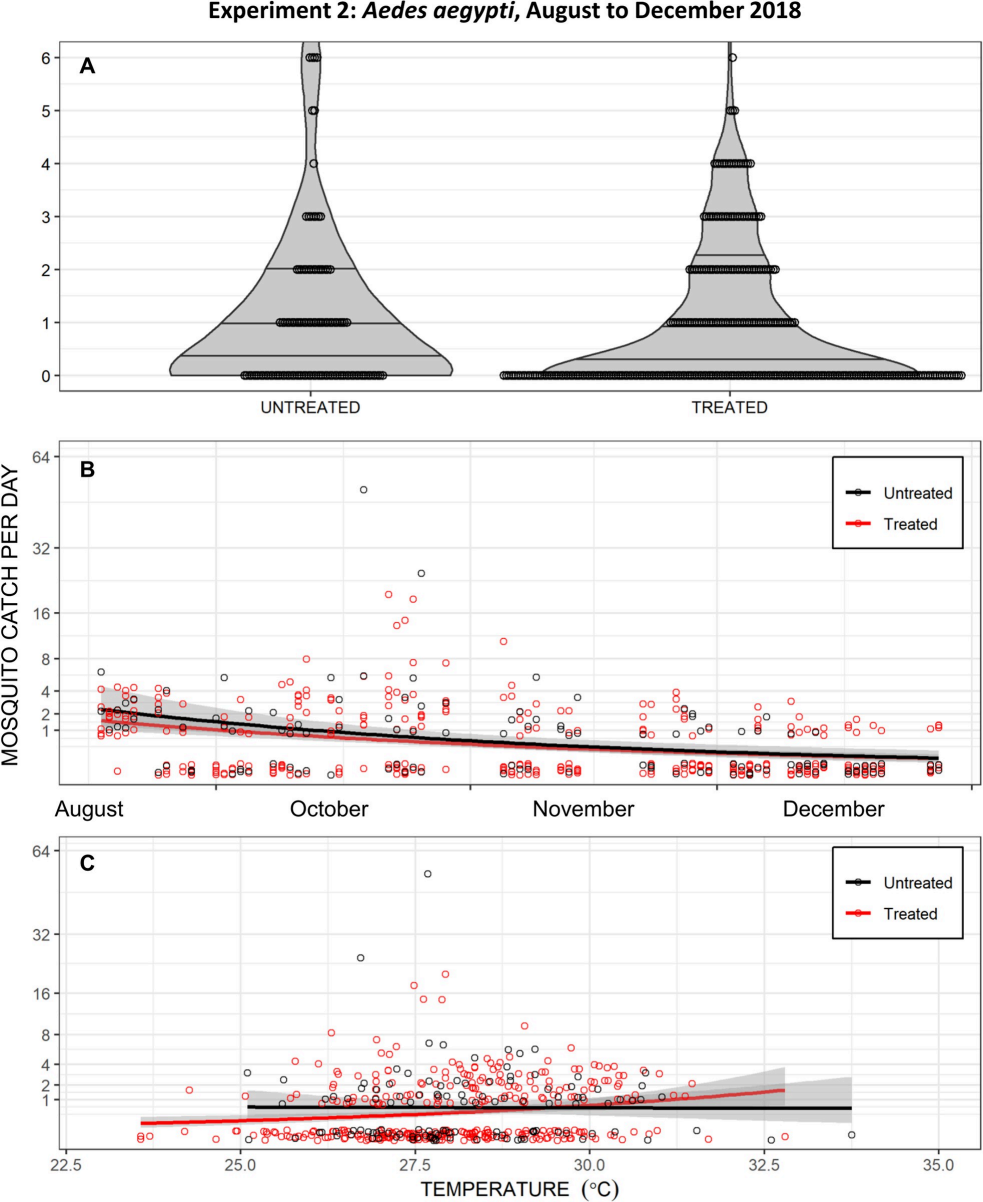
Daily *Aedes aegypti* catches from the second entomological experiment to evaluate transfluthrin emanators in Port-au-Prince, carried out from October 2018 to January 2019. In this experiment, emanators were treated with the emulsifiable concentrate (EC) formulation and placed under the chairs of users (Right-hand panel of Fig 2) sitting outdoors**. A**: Outdoor catches with treated and untreated emanators presented as separate violin plots of density distribution with the first quartile, median and third quartile indicated by three horizontal lines and overlain by a dot plot of the individual daily total catch observations. Note that three times as many landing catches were carried out on users of treated emanators than untreated emanators (Fig 4), so the width of the probability density violin graphs may be directly compared in absolute terms but not those of the dot plots. **B**: Presented as a longitudinal time course, with separate longitudinal trends for the treated and untreated emanators over time estimated and plotted using the *geom_smooth* function of the *ggplot2* package in R, specifying the general linear model (*glm*) method with time as the independent variable and mosquito catch as the dependent variable with a Poisson distribution. **C**: Presented as a function of daily mean temperature, with separate trends for the treated and untreated emanators with temperature variations estimated and plotted using the *geom_smooth* function of the *ggplot2* package in R, specifying the *glm* method with temperature as the independent variable and mosquito catch as the dependent variable with a Poisson distribution.

The second entomological assessment of transfluthrin efficacy against *Ae*. *aegypti* also yielded no evidence of protection against mosquito bites (Fig 6), with GLMM analysis indicating only a very modest and non-significant difference between *Ae*. *aegypti* landing rates on users of treated versus untreated emanators (RR [95% CI] = 0.84 [0.66, 1.07], z = -1.386, P = 0.166, from 449 females caught over 2644 hours). Graphical inspection of the explicit data for human landing rates of *Aedes aegypti* reveals no obvious reduction of landing rates by treated emanators (Fig 6A), regardless of time since treatment (Fig 6B) or temperature (Fig 6C). Indeed, even the trivial differences that are seen in the temperature dependence trends for treated and untreated emanators are the opposite of what would be expected if the former provided any protection that relied on high temperatures to facilitate evaporation of the active ingredient: Mosquito landing rates on users of treated emanators actually increased slightly with temperature and crossed over the flatter trend line for users of placebo devices.

**Fig 7:**
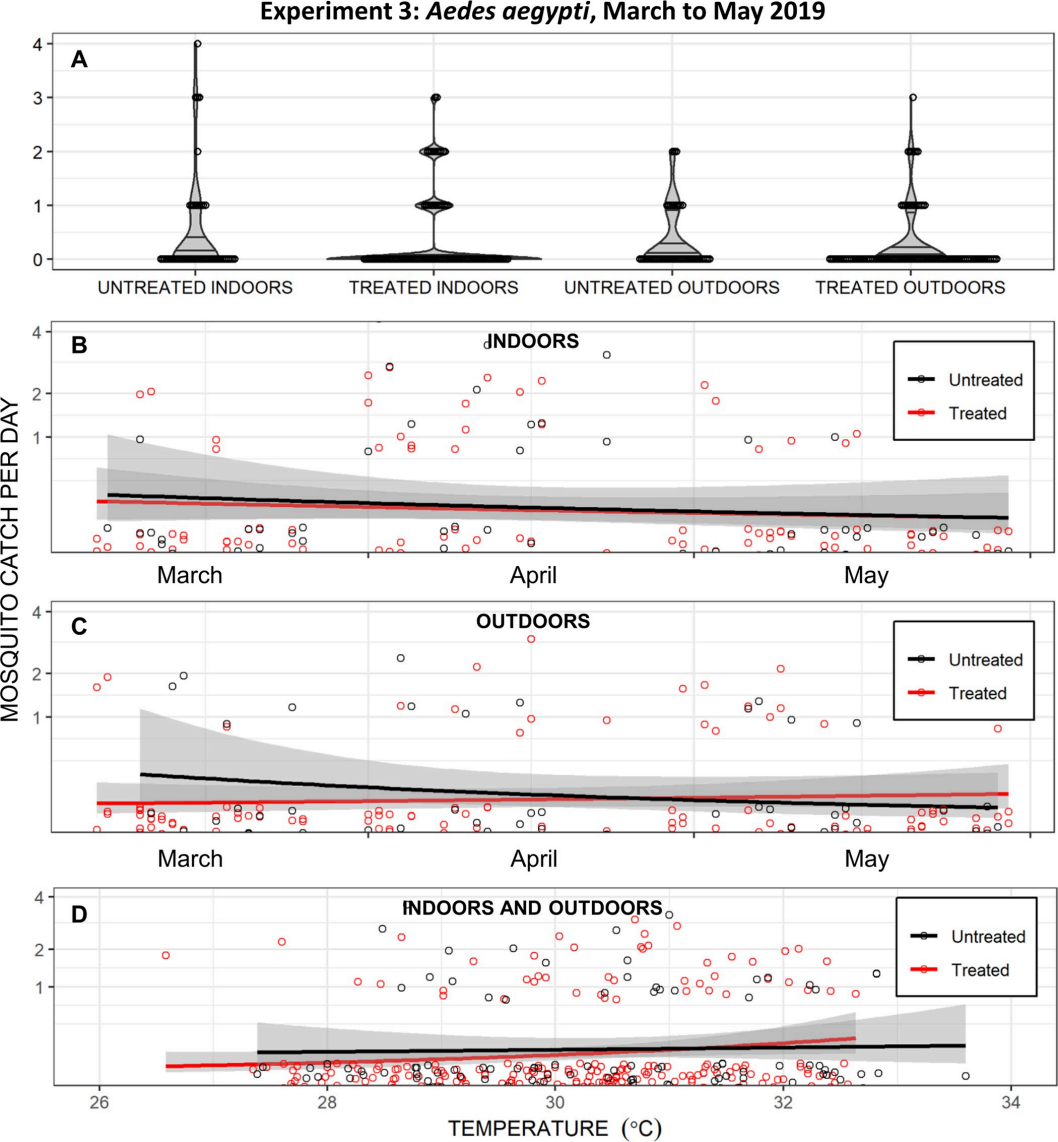
Daily *Aedes aegypti* catches from the third entomological experiment to evaluate transfluthrin emanators in Port-au-Prince, carried out from March to May 2019. In this experiment, emanators were treated with the emulsifiable concentrate (EC) formulation and placed under the chairs of users (Right-hand panel of Fig 2) sitting either indoors or outdoors**. A**: Indoor and outdoor catches with treated and untreated emanators presented as separate violin plots of density distribution with the first quartile, median and third quartile indicated by three horizontal lines and overlain by a dot plot of the individual daily total catch observations. Note that three times as many landing catches were carried out on users of treated emanators than untreated emanators (Fig 4), so the width of the probability density violin graphs may be directly compared in absolute terms but not those of the dot plots. **B**: Indoor catches presented as a longitudinal time course, with separate longitudinal trends for the treated and untreated emanators over time estimated and plotted using the *geom_smooth* function of the *ggplot2* package in R, specifying the general linear model (*glm*) method with time as the independent variable and mosquito catch as the dependent variable with a Poisson distribution. **C**: Outdoor catches presented as a longitudinal time course in exactly the same way as panel **B**. **D**: Combined indoor and outdoor catches presented as a function of daily mean temperature, with separate trends for the treated and untreated emanators with temperature variations estimated and plotted using the *geom_smooth* function of the *ggplot2* package in R, specifying the *glm* method with temperature as the independent variable and mosquito catch as the dependent variable with a Poisson distribution.

As illustrated in Fig 7, the third round of experimental entomological evaluation yielded no evidence of protection against *Ae*. *aegypti*, although the sparse mosquito densities during this period badly constrained statistical power: GLMM analyses indicate negligible reductions of landing rates, albeit with very wide confidence intervals, both indoors (RR [95% confidence intervals (CI)] = 1.05 [0.57, 1.94], z = 0.167, P = 0.868 from 63 females caught over 900 hours) and outdoors (RR [95% CI] = 0.89 [0.44, 1.78], z = -0.329, P = 0.742, from 43 females caught, also over 900 hours). No hint of reduced landing rates on users of treated emanators were obvious indoors (Fig 7A and 7B) or outdoors (Fig 7A and 7C), regardless of time since treatment (Fig 7A and 7B) or mean daily temperature (Fig 7D). Again, what little evidence of temperature dependence could be seen was negligible and with an opposite trend to that expected for temperature-dependent evaporation of a repellent active ingredient: The trend line for treated emanators has a slight upward slope and crosses over that for untreated emanators (Fig 7D).

Similar to the results from separate analysis of individual rounds of experimental assessment, pooled analysis of all the data to obtain improved statistical power yielded no evidence of significant protection against *Ae*. *aegypti* (Table 1). While landing rates were somewhat lower outdoors than indoors, and assessment rounds two and especially three were carried out at much lower densities of *Ae*. *aegypti* mosquitoes, what little protection transfluthrin emanators appeared to provide only distantly approached significance (Table 1).

**Table 1:** Statistical estimates for the protective efficacy of transfluthrin-treated emanator devices and other variables influencing the densities of female *Aedes aegypti* mosquitoes, based on the best fit generalized linear mixed model (GLMM) of the pooled data from all three rounds of experimental assessment (921 females caught over 5129 hours, as illustrated in Figs 5–7 and shared in S1 Data), assuming a negative binomial distribution for this positive integer dependent variable to account for overdispersion (S1 File).

Variable	Statistical parameter estimates		
Fixed Effects	Mean [95%CI]	z	P
*Intercept*			
Mosquito density under reference conditions	0.52 [0.24, 1.10]	-1.717	0.0896
	RR [95%CI]	z	P
*Emanator device treatment status*			
Untreated	1.0 [NA]	NA	NA
Transfluthrin treated	0.87 [0.73, 1.04]	-1.538	0.1241
*Experimental assessment*			
Round one	1.0 [NA]	NA	NA
Round two	0.34 [0.22, 0.53]	-0.472	<0.0001
Round three	0.09 [0.04, 0.18]	-6.655	<0.0001
*Location*			
Indoors (Assessment round 3 only)	1.0 [NA]	NA	NA
Outdoors	0.59 [0.35, 0.99]	-1.972	0.0486
Random effects	σ	SD	
Date	0.5643	0.7512	
Block and station	0.5616	0.7494	
Time of day	0.1035	0.3217	

CI: Confidence interval

NA: Not applicable because this was the reference value specified in the model

RR: Relative Rate

σ: Variance

SD: Standard Deviation

**Fig 8:**
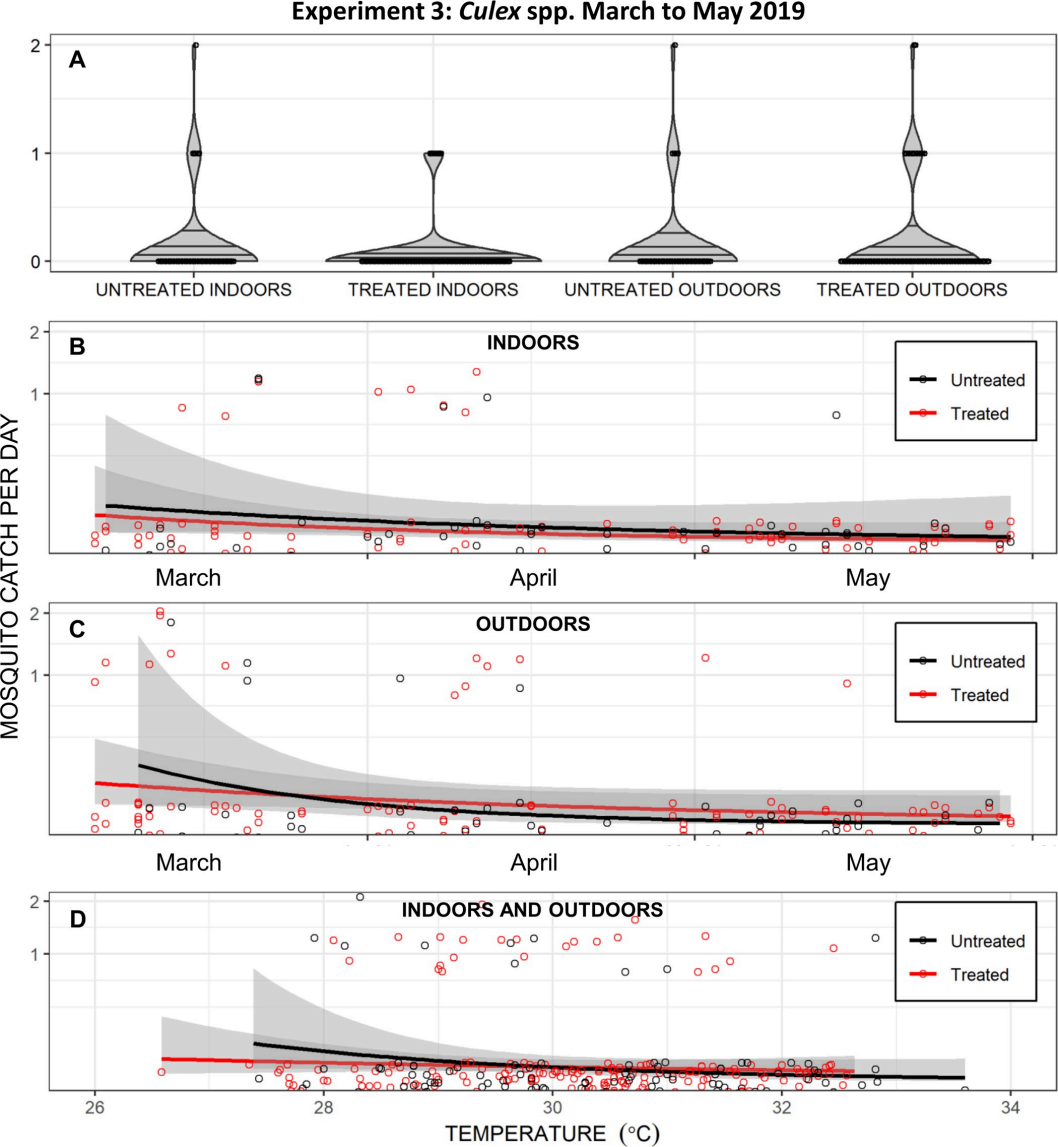
Daily catches of *Culex* spp. mosquitoes from the third entomological experiment to evaluate transfluthrin emanators in Port-au-Prince, carried out from March to May 2019. In this experiment, emanators were treated with the emulsifiable concentrate (EC) formulation and placed under the chairs of users (Right-hand panel of Fig 1) sitting either indoors or outdoors**. A**: Presented as a violin plot of density distribution with the first quartile, median and third quartile indicated by three horizontal lines and overlain by a dot plot of the individual daily total catch observations. Note that three times as many landing catches were carried out on users of treated emanators than untreated emanators (Fig 4), so the width of the probability density violin graphs may be directly compared in absolute terms but not those of the dot plots. **B**, **C** and **D**: Presented as a longitudinal time course for either the indoor (**B**) or outdoor (**C**) observations presented separately or pooled together and presented as a function of daily mean temperature (**D**), with separate Poisson-distributed smoothed averages for the treated and untreated emanators.

Insufficient numbers of *Aedes albopictus* (37 females across all three assessment rounds) were caught to allow similar assessment of transfluthrin emanator efficacy against this mosquito species. Although far too few *Culex* spp. mosquitoes were caught to allow rigorous comparison (67, 34 and 23 females in assessment rounds one, two and three, respectively), the frequency distributions of landing rates appeared similar indoors and outdoors for users of treated and untreated emanators, regardless of temperature or time since treatment (Fig 8). Correspondingly, GLMM analysis of the pooled *Culex* spp. data from all three assessment rounds, similar to that described for *Ae*. *aegypti* in Table 1, indicated little if any protective effect (RR [95%CI] = 0.96 [0.63, 1.46], z = -0.203, P = 0.807) against this genus.

**Fig 9:**
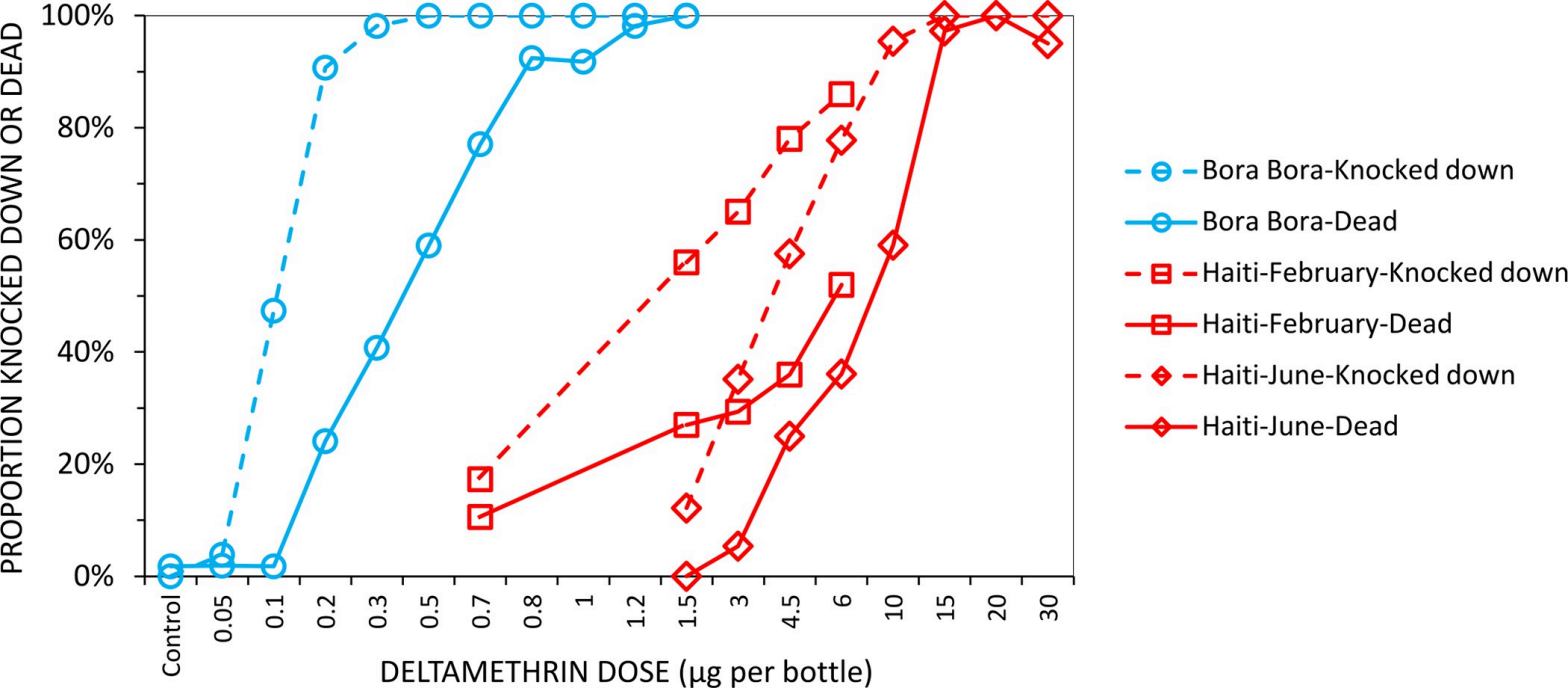
Results of transfluthrin resistance tests. Dose-response curves established using wild females collected as larvae in February and June 2019.

All pyrethroid resistance assays were carried out in February and June 2019, before and immediately after the third field evaluation (Figs 7 and 8). At the transfluthrin diagnostic concentration of 3 μg/bottle, an average of 65% of females tested were knocked down at 1h and 29% were dead at 24h using field specimens collected in February 2019 (n = 143), while specimens collected in June 2019 showed 46% of knockdown at 1h and only 10% mortality after the usual 24h recovery period (n = 68). Those results indicate a high phenotypic resistance level among wild *Ae*. *aegypti* in the study area of Port-au-Prince following WHO criteria. More bottle tests were then conducted with various transfluthrin concentrations between 1.5 and 30 μg/bottle to establish a concentration-response curve, revealing a high phenotypic resistance ratio for transfluthrin among wild *Ae*. *aegypti* in Port-au-Prince when compared to the susceptible colony (Fig 9). Notably, 100% of knockdown and 97% of mortality were reached with bottles coated with the 15μg dose, which is 5 times higher than the diagnostic concentration.

Results from the contact irritancy assessments for the transfluthrin-treated hessian panels used in Haiti with the HITSS experimental system [28, 29] are summarized in Table 2. The first tests were carried out in a manner that allowed the test mosquitoes could touch and land on the treated hessian panel surface. Most females were knocked down after few seconds and only 5% were able to escape. The HITSS was then modified using fine mesh mosquito proofing sheets to avoid direct contact with the treated material for all subsequent tests, so as to expose mosquitoes to transfluthrin vapour only. When the larger treated hessian panels were used, with mosquitoes allowed no direct contact, between 48 and 61% of females escaped from the treated chamber while 92 to 97% of those recovered from the chambers with treated and untreated hessian panels were knocked down after 10 minutes of exposure. Treated hessian panels that were 10 times smaller nevertheless yielded a similar proportion of females escaping the treated chamber (44 to 48%), although less were knocked down by the transfluthrin vapor (47 to 60%).

**Table 2:** Results of laboratory contact irritancy assays in the high-throughput screening system (HITSS) developed by Grieco *et al*. [28, 29]. Each value was calculated from a minimum of 8 replicates, using either large (290 cm^2^) or small (29 cm^2^) hessian panels treated with 5.14 g/m^2^ transfluthrin.

Date	Conditions	Total Mosquitoes (n)	Escape (%)	Knock Down (%)	Temperature (°C)	Relative Humidity (%)
March 2019	Untreated controls	255	16.5%	0.0%	24.5–25.8	24–32
	Treated-large-with contact	40	5.0%	100.0%	24.5–25.8	24–32
	Treated-large-no contact	106	61.3%	92.5%	24.5–25.8	24–32
	Treated-small-no contact	142	44.4%	59.9%	24.5–25.8	24–32
May 2019	Untreated controls	318	22.0%	0.0%	27.1–28.0	29–35
	Untreated controls	60	33.3%	0.0%	27.5–28.1	21–23
	Treated-large-no contact	122	48.4%	97.5%	27.5–28.1	21–23
	Untreated controls	70	4.3%	0.0%	27.8–28.1	39–42
	Treated-small-no contact	80	47.5%	47.5%	27.8–28.1	39–42

Results from the spatial repellency assessments for the transfluthrin-treated hessian panels used in Haiti with the HITSS experimental system [28, 29] are summarized in Table 3. In all test conditions of hessian surface and temperature, most females did not leave the central chamber where they were introduced, even though the doors between compartments were open for 10 minutes (61 to 92%). Some modest spatial repellency was observed across all tests, except for one assessment of the full-size treated hessian panel under the warmer of the two conditions that yielded a null spatial activity index. Spatial activity index varied between 0.00 and only 0.12 overall and the strongest repellency was observed with the smaller hessian piece at lower temperature. Overall, between 58 and 91% of female mosquitoes that entered the treated chamber were knocked down, while only 0 to 5% females recovered from the untreated central cylinder and the control chamber were knocked down.

**Table 3:** Results of spatial repellency assays carried out in the laboratory using the high-throughput screening system (HITSS) developed by Grieco *et al*. [28, 29]. Each value was calculated from a minimum of 8 replicates, using either large (290 cm^2^) or small (29 cm^2^) hessian panels treated with 5.14 g/m^2^ transfluthrin.

Period (Temperature)	Size	Treated chamber		Central Chamber		Control chamber		Spatial activity Index
		Recovered (%)	Knocked Down (%)	Recovered (%)	Knocked Down (%)	Recovered (%)	Knocked Down (%)	
March 2019	Large	9.6	85.7	76.3	3.0	14.2	0.0	0.05
(24.5–25.4°C)	Small	13.1	57.9	61.4	1.1	25.5	0.0	0.12
May 2019	Large	13.9	90.9	72.2	5.3	13.9	4.5	0.00
(27.7–27.9°C)	Small	2.5	75.0	92.4	0.0	5.1	0.0	0.03

## Discussion

Taken at face value, these results consistently indicate that the emanator prototypes and transfluthrin formulations evaluated here provided negligible protection against wild, free-flying populations of *Ae*. *aegypti* in Port-au-Prince, Haiti. Over the course of three separate and carefully controlled experimental evaluations, no statistically significant protective effect could be demonstrated (Figs 5–7 plus Table 1), regardless of the transfluthrin formulation used, positioning of the emanator or weather conditions at the time (Figs 2 and 3).

Although the wild field populations of *Aedes aegypti* in Haiti appear strongly resistant to the lethal effects of contact exposure to transfluthrin (Fig 9) and pyrethroid resistance is known to be associated with reduced behavioural responsiveness to the spatial repellency of this active ingredient [30], it remains unclear whether such physiological resistance could have contributed to the apparent lack of protection against *Ae*. *aegypti* reported here from Haiti. Indeed, dose-response experiments with the same prototype inside large cage semi-field systems in Tanzania indicated no substantial difference in behavioural response profiles between modestly resistant wild populations of *Ae*. *aegypti* and a fully susceptible colony of the same species originating from the same setting or a fully susceptible colony of *Anopheles gambiae* [14] thus confirming that physiological resistance is unlikely to have played a major role in the generally disappointing entomological results against this species. Furthermore, similar large-cage assessments of a sandal format emanator against *Ae*. *aegypti* from a fully susceptible colony indicate that this prototype prevented only a third of bites [31], suggesting that mechanisms other than physiological resistance may be responsible for their apparently limited efficacy as spatial repellents against *Aedes* when used to treat hessian strips in this manner.

Regarding the counterintuitive laboratory results obtained using the HITSS system [28, 29] (Table 2), it is known that different concentrations of transfluthrin vapor can lead to varying repellency levels, sometimes even attracting mosquitoes [30, 32], so this could explain why the smaller piece of treated hessian seemed to have a stronger apparent repellent effect. While it is also possible that the higher concentration of vapor produced by the full-size hessian panel could quickly saturate the whole system and confound the intended function of the test, the low knockdown rates in the central and control chambers suggest this was probably not a major issue in this case. A more direct and parsimonious interpretation of these results is that non-lethal repellency *per se* may play a relatively minor role in the overall mode of action of transfluthrin when deployed through this hessian emanator format.

The prototype emanator design evaluated here differs substantively from the suspended ribbon prototypes that have proven successful against night biting *Anopheles* and *Culex* in Africa [9–11, 33]. However, it is notable that several other studies in rural Tanzania confirm satisfactory efficacy against *Anopheles* and *Culex* for similar portable designs [23, 24] to that used in this evaluation against *Aedes* in urban Haiti. Also, a quite similar entomological evaluation in urban Dar es Salaam in Tanzania, involving minor variations on the same emanator design and transfluthrin formulations from the same manufacturer, also indicated little if any reduction of human landing rates [14]. Semi-field evaluations in Tanzania using large cages and insectary-reared mosquitoes, even including those derived from recently wild-caught stock, confirmed that neither replacing the METs [15–19] used here with human landing catches (HLCs) [14, 19, 34, 35] nor changing the position of the emanator [14] led to any apparent improvements in protective efficacy. Also, entomological field evaluations of a quite different sandal format of emanator [31, 36] in Brazil, which also used the gold standard HLC method, yielded no consistent evidence of satisfactory protection against *Ae*. *aegypti* (Alvaro Eiras, Personal communication). Furthermore, a recent large-scale field trial of a different transfluthrin emanator device in urban Iquitos, Peru, which surveyed human exposure levels based on the number of blood fed *Aedes* inside the houses of end users [5], yielded statistically significant but otherwise identical results to those reported here.

On the other hand, however, these consistently outcomes from entomological assessments in Haiti, together with similar results from Tanzania [14] and Brazil (Alvaro Eiras, Personal communication) contrast starkly with the observations of complementary social science investigations [13] that engaged with the Haitian households whose same treated emanators were intermittently borrowed for the entomological evaluations reported herein. These carefully triangulated sociological investigations, using several complementary survey methods, consistently indicate moderate-to-high levels of user satisfaction, even in their third iteration when they were redesigned to minimize biases introduced by the investigators and by competing interests among the end users (See *Methods*, *Ethical Considerations* and reference [13]). Similarly, in Brazil, end users of a sandal format of transfluthrin emanator [31, 36] also expressed surprizing levels of satisfaction with the protection provided against mosquito bites, despite yielding generally unsatisfactory results against *Aedes* in open field assessments (Alvaro Eiras, personal communication). Furthermore, all rounds of social science assessment in this Haitian setting [13] used exactly the same individual emanator devices as the entomological evaluations reported herein, so this clear contrast cannot be explained in terms of differing prototype designs, transfluthrin formulations or treatment procedures.

It may therefore be useful to consider the potential influence of emanator deployment practices upon objective and subjective measures of protective efficacy. For example, some of the Haitian community participants who routinely used the same individual emanator devices [13] suggest that use of a single emanator device, rather than two, might explain the apparently minimal protective efficacy observed here under similar full field conditions:

*“I had given an emanator* [away]. *I still have one left*. *When I had two*, *it was more efficient*. *Now I only have one*. *It lacks efficiency*.*”* Community end user, Haiti (Obrilliant, Unpublished)

Thus, it seems that using two or more emanators might be more effective, creating a protective “bubble” [32, 37] even in windy open outdoor spaces, where mosquitoes could otherwise safely attack users of a single emanator by flying with or across the wind on their approach. On the other hand, recent semi-field assessments of sitting in between two similar self-standing emanators in Tanzania yielded modest estimates of protective efficacy against *Ae*. *aegypti* [19, 34, 35], similar to those reported for a single device under similar conditions in the same country [14]. It therefore seems unlikely that the discouraging results against wild, free-flying populations of the same species in the same African setting arose from using only one emanator rather than two or more.

Interestingly, community users in both Haiti and Brazil described household deployment practices that seemed to target indoor resting mosquitoes rather than host-seeking mosquitoes (Reference [13] and Alvaro Eiras, personal communication), so this may be a potential application worth investigating in the future. Given that we rarely observed *Ae*. *aegypti* resting indoors in Port-au-Prince, it seemed reasonable at the time to speculate that community end users were instead targeting the *Culex quinquefasciatus* that can be so abundant indoors in such urban tropical settings, so our third entomological evaluation extended collections into the hours of darkness to target this nocturnal species. Although no evidence of efficacy against *Culex* spp. was obvious from those data (Fig 8), insufficient numbers of *Culex* spp. mosquitoes were captured to reach any firm conclusion.

Although questions have been raised about the validity of the MET method [15–19] for collecting human-biting mosquitoes [19], the traditional and reliable HLC method has repeatedly yielded essentially identical results under semi-field conditions in Tanzania [14, 19, 34, 35]. However, both approaches actually record the rates at which mosquitoes land rather than bite *per se*, but transfluthrin and other pyrethroids are known to incapacitate mosquitoes so that they cannot feed again for up to a day [32, 38]. Furthermore, the investigators have sometimes observed mosquitoes landing on them but not biting them while using such emanators during previous studies [11]. It may therefore be worth considering non-entomological indicators of exposure to biting *Aedes* mosquitoes [39–45] and the arboviruses they carry [5] as alternative methods for assessing the efficacy of such spatial repellent products.

Having said all that, the low and non-significant levels of apparent efficacy estimated here using entomological methods are remarkably similar to those recently estimated for a quite different transfluthrin emanator device in Iquitos, Peru [5], based more reliably upon direct surveys of the numbers of blood-fed *Ae*. *aegypti* inside the homes of end-users (13% versus 12% reductions, respectively). Interestingly, the carefully controlled large scale trial of Morrison *et al*. in Peru [5] also demonstrated a larger effect size for protection against arboviral infections (34% reduction), similar to the contrasting entomological observations reported herein and the more encouraging perspectives shared by end-users in the same neighbourhoods of Port-au-Prince [13]. Nevertheless, it remains unclear how the very different results of these two distinct assessments in Haiti may be reconciled with each other and with complementary assessments in Tanzania and Brazil.

## Conclusions

The underlying reasons for the apparent contradiction between the lack of entomological evidence for protection against host-seeking *Aedes* and more encouraging results from social science and epidemiological assessments, collectively spanning studies from Tanzania [14, 31], Haiti [13], Brazil (Alvaro Eiras *et al*., Personal communication) and Peru [5], therefore remains unresolved. While it may be useful to explore whether serological indicators of infection [5] and of exposure to mosquitoes [39–45] can resolve this dilemma in the future, for now it remains unclear whether these particular long-lasting transfluthrin emanator devices are effective against the *Aedes* species responsible for most of the world’s arbovirus transmission. It also remains to be determined whether they may have useful alternative applications against *Culex* mosquitoes indoors, as suggested by some of the shared perspectives of end users from both Haiti [13] and Brazil (Alvaro Eiras, Personal communication). More encouragingly, similar contrasts between the entomological and epidemiological results from a recent large scale trial of a different transfluthrin emanator product in Peru suggest that, for reasons that remain to be understood, such devices may provide useful protection against *Aedes*-borne arboviral infections despite apparently providing only modest protection against biting *Aedes* mosquitoes [5].

## Supporting information

S1 Data All the entomological data used to generate Figs 5 to 9 and Tables 1–3. (CSV)

S1 Protocol Full approved English language version of the protocol for this study, together with the complementary social science assessments of end user perceptions in these same Haitian communities [13] and a similar entomological assessment of transfluthrin emanator efficacy in Tanzania [14], both of which were carried out in parallel with this study. See also S2 Protocol for all relevant annexes in English and S3 Protocol for the approved protocol and annexes as translated into French and Haitian Creole.
(PDF)

S2 Protocol English language versions of all annexes to the approved protocol for this study, together with the complementary social science assessments of end-user perceptions in these same Haitian communities [13] and a similar entomological assessment of transfluthrin emanator efficacy in Tanzania [14], both of which were carried out in parallel with this study. See also S1 Protocol for the main protocol document itself in English and S3 Protocol for the approved protocol and annexes as translated into French and Haitian Creole.
(PDF)

S3 Protocol Full approved protocol (French only) and all relevant annexes (French and Haitian Creole) for this study, together with the complementary social science assessments of end-user perceptions that were carried out in parallel in these same Haitian communities [13] (Figs 1 and 2), as translated, reviewed, approved and used in Port-au-Prince, Haiti. See also S1 and S2 Protocols for the approved protocol and annexes in English, respectively.
(PDF)

S1 File The R script used to generate Table 1. (R)

S2 File (DOCX)

## Abbreviations

CI: Confidence interval
EC: Emulsifiable Concentrate
GLMM: Generalized Linear Mixed Model
HITSS: High-Throughput Screening System
NA: Not applicable because this was the reference value specified in the model
RR: Relative Rate
σ: Variance
SD: Standard Deviation
TC: Technical Concentrate
WHO: World Health Organization
