# Supplementary material for: Entomological assessment of hessian fabric transfluthrin vapour emanators as a means to protect against outdoor-biting Aedes after providing them to households for routine use in Port-au-Prince, Haiti
Source: PLoS One. 2024 May 28;19(5):e0298919. doi: 10.1371/journal.pone.0298919 (PMC11132518; doi:10.1371/journal.pone.0298919)
Supplement: S1 Protocol — See also S2 Protocol for all relevant annexes in English and S3 Protocol for the approved protocol and annexes as translated into French and Haitian Creole. (PDF) [file pone.0298919.s002.pdf]

Please do not staple, paperclip only

Liverpool School of Tropical Medicine
Research Ethics Committee
INTERNAL LSTM APPLICANTS ONLY

APPLICATION FORM FOR ETHICAL APPROVAL

(Revised December 2013)

Please refer closely to the Guidance Notes when completing this form.
All questions must be answered. Any form with sections left blank or answered with N/A will be returned. This form must contain all information necessary for the Research Ethics Committee to make a decision on Ethical Approval.

**APPLICANT FULL NAME (w/ title)**	Dr Gerard F. Killeen<br><br>Reader at LSTM and Resident Guest Scientist at the Ifakara Health Institute (IHI) in Tanzania
**PROJECT TITLE**	Affordable, scalable, low-technology transfluthrin emanators for protecting against transmission of Zika, Dengue and Chikungunya viruses

Ethical Approvals

Have you submitted this proposal to the LSTM Research Ethics Committee before?	**YES** ☒	**NO** ☐
If 'YES', please give date of previous review:	31st October 2016	

To which other ethical committees has/will this protocol be submitted? (please list)

This revised protocol has been reviewed and approved (certificate attached) by the Institutional Review Board of the Ifakara Health Institute in Tanzania, and will be reviewed by the Medical Research Coordination Committee (MRCC) at the National Institute for Medical Research (NIMR) at national level in Tanzania, as well as the Ethical review board of the Ministry of Public Health and Population in Haiti following approval at LSTM.

APPROVALS – Please list any ethical review committees which have already approved the protocol.

Institutional Review Board of the Ifakara Health Institute in Tanzania (Certificate attached)

In-Country Ethical Approval - Please list the country(ies) where the research will be carried out and whether or not in-country ethical approval is required.

Country 1: Tanzania	**YES** ☒	**NO** ☐
Country 2: Haiti	**YES** ☒	**NO** ☐
Country 3:	**YES** ☐	**NO** ☐
Country 4:	**YES** ☐	**NO** ☐

Add additional lines if your research is taking place in more than four countries.

Please note if you have marked 'NO' for any country above WRITTEN EVIDENCE must be provided to confirm that in-country ethical approval is not required. This evidence should be attached as an annex to the application. Acceptable evidence includes:

- a letter from the national Ministry of Health or other relevant regulatory authority
- a letter from an authorised signatory at a local partner

A letter from a co-investigator or other researcher at a local partner institution is NOT sufficient evidence. Your ethics application will not be considered until evidence is provided.

Research Team

List LSTM research team and all collaborators.
(Please include all overseas collaborators and give their affiliations, qualifications and role in the study).

NAME	ORGANISATION	QUALIFICATIONS	ROLE IN STUDY	GEOGRAPHIC LOCATION
Gerry F. Killeen	LSTM/IHI	BSc, PhD	Principal Investigator	Ifakara, Tanzania
Nicodem J. Govella	IHI	BSc, PhD	Co-Investigator	Dar es Salaam, Tanzania
Joseph Frederick	Progamme National de Controle de la Malaria (PNCM)	30 years of vector control experience Haitian PhD equivalent	Co-Investigator	Port-au-Prince, Haiti
Sheila B. Ogoma	Kenya Medical Research Institute	BSc, MSc, PhD	Collaborator	Kisumu, Kenya

Daniel Impoinvil	Centers for Disease Control and Prevention	BSc, MPH, PhD	Collaborator	Atlanta, USA
Cyrille Czeher	Institut de Recherche pour le Développement	BSc, PhD	Collaborator	Montpellier, France
Thom Eisele	Tulane University	BSc, MPH, PhD	Collaborator	New Orleans, USA
Thom Dreutz	Tulane University	BSc, MSc, PhD	Collaborator	New Orleans, USA
Katherine Andrinopoulos	Tulane University	BSc, MPH, PhD	Collaborator	New Orleans, USA

**If proposal is for work relating to a MPhil/PhD, please state the name and Department of Supervisor/Tutor**	
Supervisor	Not applicable
Department	Not applicable
Signature	Not applicable

**Sponsorship and Indemnity Cover** Please see sponsorship and indemnity guidance notes and request form on the LSTM intranet http://pcwww.liv.ac.uk/lstmintranet/research_management/applyingforRG/intro.htm		
Have you submitted a sponsorship and indemnity request form to the Research Office?	YES (circled)	NO
If no, do you intend to submit a form and if so, when?		
If you do not intend to submit the form please give reasons.	Not applicable	

GLOSSARY OF TERMS

Please provide a list of acronyms used in the application with their full name and any relevant explanation that would be helpful to committee members that may not be an expert in your area of work.

SECTION A

STUDY OUTLINE

A.1	**LAY SUMMARY:** Please use simple language which is understandable to a non-scientific/non-academic audience. Sufficient detail of the protocol must be given to allow the Committee to make an informed decision without reference to other documents. Please spell out all acronyms. *(max 300 words)*

Pandemic urban Zika transmission across Africa, Asia and Latin America is predominantly mediated by *Aedes aegypti* and *Ae. albopictus* [1-5]. Existing repellent products for protecting against such day-biting mosquitoes, especially while active outdoors, only last hours, days or weeks per dose or application. Sustaining continuous protection is therefore impractical, and repeated replacement is unaffordable to low-income populations. We recently developed a low-technology emanator [6], which releases repellent transfluthrin vapour more slowly, providing at least 4 months of >90% protection against night-biting *Culex* and *Anopheles* mosquitoes in urban Dar es Salaam, Tanzania, despite the presence of considerable pyrethroid resistance [7]. This novel emanator consists only of a Hessian fabric strip, which can be safely treated and re-treated by any individual, community, program or local manufacturer [6, 7]. Furthermore, equivalent efficacy and durability has since been achieved with a 10-fold lower transfluthrin dosage of only 1ml, costing only £0.09 per treatment. We have also recently developed a new electric grid trap for measuring attack rates of mosquitoes, which prevents exposure of human volunteers to potentially infectious bites [8]. We therefore propose to apply this novel trapping device to demonstrate that this new repellent technology provides ≥6 months of ≥80% protection against day-biting *Aedes aegypti*, probably the most important vector of pandemic Zika transmission globally, in relevant field settings on the coast of Tanzania. We will then determine the optimum size, orientation and shape for the Hessian fabric strip within a protective cover, in terms of minimizing size and convenience without compromising efficacy. This optimized emanator design will subsequently be evaluated under normal conditions of routine community use by representative end users in Haiti, in terms of the extent and duration of their effectiveness, as well as their potential programmatic scale up.

A.2	**IMPORTANCE OF THE RESEARCH:** Please state the intended value of the research and explain how this research fits in with national/international research priorities. *(max 300 words)*

While Zika transmission in Africa has been historically enzoonotic, the new pandemic virus lineage spreading across Asia and Latin America has adapted to transmission betweens humans [9, 10]. Diverse *Aedes*, *Culex*, *Mansonia* and *Anopheles* mosquitoes may act as vectors, but pandemic urban transmission across Africa, Asia and Latin America appears predominantly mediated by *Aedes aegypti* and *Ae. albopictus* [1-5]. The Zika virus was originally identified in neighbouring Uganda [11, 12], has since been detected in numerous locations across tropical Africa [2], and is now spreading all across the tropics through anthroponotic transmission chains through urban *Aedes* vectors [1, 4, 5, 9, 10]. New preventative measures are therefore urgently required to protect the public against the Zika virus, as well as the Dengue and Chikungunya viruses transmitted by the same vectors [5].

It may take years to develop and evaluate any vaccine or drug therapy for Zika virus, and these are likely to be specific for that single pathogen. The *Aedes* mosquitoes that transmit Zika typically attack people during daylight hours when they are awake and active, often outdoors, so bed nets or even mosquito-proofed housing provide limited protection. Affordable new repellent products are therefore urgently needed to protect at-risk populations all across the tropics, against its outdoor-biting *Aedes* vectors. Existing repellent products only protect against mosquitoes for hours, days or weeks per application or dispensing dose, so they are too expensive and impractical for continuous, indefinite use in low-income countries [13, 14], and some formulations may even be dangerous [15].

A.3	**DUPLICATION OF RESEARCH:** Indicate what steps have been taken to ensure this work has not already been carried out. If this project or a similar one has been done before what is the value of repeating it? *(max 300 words)*

Comprehensive literature searches and monitoring over the years, including derived authoritative review articles we have published [13, 14, 16, 17], reveal no equivalent technologies to either these low technology transfluthrin emanators or the electric grid mosquito traps described in this proposal. This protocol therefore duplicates no preceding research. This protocol not only evaluates this new repellent technology against day-biting *Aedes* mosquitoes in Tanzania, where it was developed, but also in Haiti to determine whether the observed efficacy can be reproduced in another challenging low-income setting on another continent, where local *Aedes aegypti* populations are distantly related and genetically far more structured [18]. Given the overall paucity of high quality evidence for the effectiveness of any vector control tool against *Aedes* mosquitoes, such carefully-controlled and replicated experimental studies have recently been recommended as a top global priority [19].

A.4	**OBJECTIVES:** List the major objectives of the study. These must be clearly stated and achievable by the proposed design and methods

Our overall goal is to apply this novel trapping device to demonstrate that this new repellent technology provides ≥6 months of ≥80% protection against day-biting *Aedes aegypti*, which is probably the most important vector of Dengue, Chikungunya and Zika transmission globally. Our specific objectives, to be addressed in field settings with high densities of this vector in both Tanzania and Haiti, are as follows:

1. Demonstrate that the new transfluthrin emanator provides ≥80% protection against wild *Aedes aegypti* populations.
2. Demonstrate that this minimum level of protection lasts at least six months, and ideally one year.
3. Demonstrate that emanator use does not divert *Aedes aegypti* mosquitoes to feed on unprotected non-users.
4. Determine the optimum shape, orientation and size of the emanator, in terms of minimizing size and maximizing convenience without compromising efficacy.
5. Measure the extent and duration of entomologically-measured protective efficacy of these optimized prototypes by end users in the community.
6. Evaluate the perceived protective effectiveness and user acceptability of transfluthrin emanators, and survey end user perspectives of potential pitfalls, opportunities, and optimal communication tactics for programmatic scale up.

A.5	**METHODOLOGY:** Please include the methodology for each objective (if different) and justify the rationale behind the use of the chosen methodology. Please use simple language which is understandable to a non-scientific/non-academic audience and spell out all acronyms.

Objectives 1 to 3: All of the first three objectives will be addressed by adapting a single, integrated experimental design that has already been successfully applied to assess the protection this device provides against nocturnal mosquitoes (Figure 6). The only major modifications will be to:

(1) Conduct these experiments during daytime hours, when *Aedes* Zika vectors are most active.

(2) Measure human biting rates with recently designed, user-insulated electric grid traps placed around the feet of operators who are fully protected against mosquito bites with protective netting [8], rather than traditional human landing catches which inevitably expose those operators to potentially infectious bites [20].

(3) We have now modified the physical format of the treated Hessian strips, so that they can be suspended above the user or placed under their chair (Figure 5B), so this revised format will be used instead of the original format suspended upon four poles placed around the user (Figure 5A [6, 7]).

Only adult males (≥18 years) and adult females of non-child-bearing age (≥50 years) will be recruited as volunteers, to comprehensively avoid any risk of infection with Zika, malaria or any other vector borne pathogen to which pregnant women are particularly vulnerable.

Each working week, the following procedure will be completed to achieve one full experimental replicate of one

experimental block. Each experimental block will consist of an open field or garden with dimensions of at least 160 x 80m, many of which exist within *Aedes*-infested commercial properties in the north of Dar es Salaam in Tanzania [21, 22] and will also be sought for in Haiti. In order to ensure that each experimental block at least matches the mosquito attack rates assumed in the sample size calculations (A.9), each open field or garden considered for use as an experimental block will be surveyed for three full days with the electric grid trap to ensure at least 20 *Aedes aegypti* are captured per person day of trapping. Only candidate sites matching this minimum requirement will be included as experimental blocks in the following experiments.

Two pairs of volunteers will be assigned treated (T) emanators or untreated negative control (C) emanators, with the latter being a placebo with no active ingredient. The revised format of the emanator described in figure 5B will be used, placed leaning up against the back of chair of the user sits in, immediately behind user's feet and the electric grid trap they are enclosed within. Two emanator users will sit at two locations situated 80m apart within the open field or garden (Figure 6). The two locations will be assigned the numeric identifiers 1 and 2, so they are described as C1 or C2 when volunteers sitting there are using the untreated control and T1 or T2 when using the treated strips. One of the following 3 arrangements will be randomly assigned to one day of experimentation out of every 3 working days in the working week: T1-C2, C1-T2 or C1-C2. This three-day experimental cycle will be conducted from Tuesday to Thursday of each working week, with Mondays and Fridays set aside for various logistical requirements, moving experimental equipment to and from the experimental block, and community sensitisation through discussions with local residents, property owners, and leaders.

Electric grid trapping will be conducted for 6 hours, split into 2 shifts of 3 hours in the morning and evening of each experimental day. Provisionally, these two shifts will be from 06:00 to 09:00 hours in the morning and 15:00 to 18:00 hours in the evening, but the exact start time of each three-hour shift, the duration of which will be fixed, will be adapted to local conditions depending on when exactly dawn and dusk and *Aedes aegypti* activity peaks occur locally. Each of the two strip users will sit at their assigned location within an electric grid, and by a matching pair of additional operators without strips will sit nearby (strip non-users). These two non-users will sit in chairs at the same randomly assigned angle (0°, 60°, 120°, 180°, 240° and 300°) and distance (2m, 5m, 10m, 15m, 20m and 25m) from each of the two strip-users sitting within electric grid traps. Each day, these angles and distances will be randomly assigned afresh without replacement to the 6-hour sequence of one-hour periods encompassing the two 3-hour morning and evening shifts described above. Catching will be conducted for 45 minutes each hour, with 15 minutes allowed for rest, refreshment, and collecting mosquitoes from the grids. At the end of this 15 minute break, the two volunteers assigned to be the strip user and non-user at each location will exchange positions, so that each volunteer spends and equal, exactly-balanced amount of time at the user and non-user stations, so that biases associated with the differential attractiveness of individuals to mosquitoes are minimized.

Based on the sample size calculations detailed in section A.9, eight replicate pairs of treated strips (T) and matched placebo controls (C) treated with the detergent diluent only, will each be assigned to one of 8 such experimental bocks (fields/gardens), each of which will have 4 locally-recruited volunteers, so that the variations between individual treatment replicates, block-specific sets of volunteers, and experimental block replicates can be combined into a single source of variance to be measured and accounted for in the statistical analysis, thus minimizing the number of degrees of freedom and maximizing statistical power. One full experimental replicate, across all 8 blocks and assigned pairs of strips will be accomplished by randomly assigning each block and assigned strip pair to one of eight working weeks within a two-month survey cycle.

Objective 4: First we will evaluate four different shape-orientation formats for the holders (Figure 8) of a standard Hessian fabric strip of approximately the same size as the existing emanator (1.0 m^2). The optimum shape and orientation (selected primarily based on efficacy, and secondarily based on observations of practicality and convenience by the investigators) will then be evaluated with different sizes of contained Hessian fabric strip.

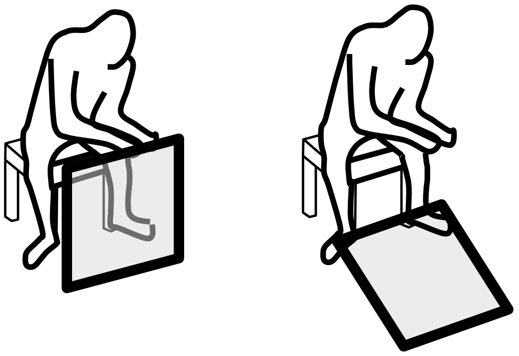

Figure 8. Different emanator holder shape and orientation formats to be assessed with through objective 4.

To evaluate and compare the efficacy of different strip holder formats, a replicated Latin square design will be used. One complete replicate of the experimental design will be completed in one block of 8 catching stations, through which one treated (2ml of transfluthrin) and one untreated (water and detergent carrier only) emanator in each of the 4 design formats described in figure 8 (4×2 = 8 format-treatment units) will be rotated in a random order over the course of 8 days.

Eight human volunteers assigned one of the eight format-treatment combinations each day will collect mosquitoes using the same mosquito electrocuting traps applied in exactly the same way as described above for objectives 1 to 3 (Figure 7). However, no nearby non-user at varying distances and angles will be required, because potential for protection/diversion will have already been addressed by objectives 2 and 3, so the large open field sites described in figure 6 will no longer be necessary. The catching stations will therefore be placed in among houses to maximize mosquito density (*Aedes aegypti* thrive in peri-domestic living areas but don't fly far from them, so in our experience densities are noticeably higher in these micro-environments), but will also be chosen to minimize disturbance of the residents or accidental contact with the electric grids, which we will fence off to protect children and livestock against potential exposure. Note that each volunteer will be allocated to a single, fixed station at each block for the duration of that replicate, so that the two sources of variations in capture rates associated with station and volunteer are combined into a single source of variance that can be captured with a single random effect and maximum statistical power in the analysis.

Each day of work will comprise the same sequence of 6 one-hour collection periods (3 in the morning and 3 in the evening) as the procedures for objectives 1 to 3. Each of these one-hour periods for a given day will be randomly allocated without replacement to one of 6 angles (0°, 60°, 120°, 180°, 240° and 300°, relative to North), which will be the same for all 8 catching stations for that day and hour period. Each hour, the chair of the catcher and the mosquito electrocuting grid he uses will be rotated together around the centre of the catching station to face in that particular direction, so that all possible orientations relative to wind direction (measured with a miniature weather station placed nearby) are represented in the resulting dataset.

Based on the sample size calculations described in section A.9, we will repeat 6 times the 8×8 Latin square design described above for a single replicate in a single block of 8 catching stations, by repeating it in 6 distinct blocks in different parts of Dar es Salaam, with freshly-prepared replicate specimens of the 8 format-treatment combinations for each block. While the experimental design for objective 4 is different from objectives 1 to 3 in terms of replication, rotation, and the shape and size of the emanator devices, the dose of transfluthrin applied will be identical, and the number of volunteers has been adjusted to the experimental design. The procedures each individual volunteer will participate in each working day will therefore be identical to those for objectives 1 to 3.

Once an optimal emanator holder format has been selected based on the results of the experiment described immediately above, we will then evaluate a range of different sizes of fabric strips to be assessed in this format using essentially the same procedure. Strips of the following sizes, with a constant 2:1 aspect ratio 0.01, 0.02, 0.05, 0.1, 0.2, 0.5 and 1.0m^2, will be treated with 2ml of transfluthrin and fitted to holding frames scaled in size proportionally to the strips themselves. These seven different sizes of transfluthrin treated emanators combined with a negative control (no device) gives 8 different experimental treatments to be assessed, just as per the experimental design and sample size calculations described above. Correspondingly, 6 replicate sets of all these 8 treatments will be independently assessed in 6 different blocks of 8 catching stations. As described for the previous experiment, each replicate set of 8 treatments will be rotated through the 8 catching stations in a Latin Squares design over 8 nights in a single block. For the next new block of catching

stations in a different part of Dar es Salaam, a freshly prepared replicate of the 8 different emanator treatments (including the negative control) will be assessed in the same way, and this procedure will be repeated until 6 completely independent replicates of this single rotation experimental design have been completed. Overall, this experiment will take 48 days of field work (8 days per block and rotation replicate × 6 blocks and replicates) that will be distributed across a working period of 2 months to allow personnel time to rest and attend to other commitments.

Objective 5: The emanator design optimized in Tanzania as described under objective 4 will be then evaluated in Haiti in terms of both their entomologically measured protective efficacy as measured with mosquito electric grids, and their perceived effectiveness by end users in the community. Both of these outcomes will be monitored over the full course of the Haitian summer (6 months from May to October) to establish how both measured efficacy and perceived effectiveness vary with time since treatment and weather conditions.

The protective efficacy of treated emanators distributed selectively to the consenting households within the community will be assessed using the same electric grids described in detail under objective 1 to 3 (Figure 7). As per objective 4, no nearby non-user at varying distances and angles will be required, because potential for protection/diversion will have already been addressed by objectives 2 and 3, so the large open field sites described in figure 6 will no longer be necessary. The catching stations will therefore be placed in among houses to maximize mosquito density as described for objective 4. Similarly to objective 4, a replicated Latin square design will be used to conduct the entomological efficacy evaluation, with 3 pairs of treated emanators distributed to each of 3 participating households (2 per household, 6 in total) in each experimental block. These recruited households will also consent to allow the research team to conduct mosquito collections over a series of 8 continuous days (6 treated emanators used by the householders plus a pair (2) of untreated (detergent and water only) negative controls) once every 2 months at 2 stations per household in or around their peri-domestic living area. A further 2 catching stations will be established with consent from relevant households in the same area to give a total of 8 catching stations to accommodate the 8 experimental treatment units (6 treated emanators plus two negative controls).

One complete replicate of the experimental design will be completed in one such block of 8 catching stations by rotating all 8 experimental treatments through these 8 stations in a random order over the course of 8 days. Eight human volunteers assigned one of the eight of the 8 treated or untreated emanators each day will collect mosquitoes using the same mosquito electrocuting traps applied as described above for objectives 1 to 3. Each volunteer will be allocated to a single, fixed station at each block for the duration of that replicate, so that the two sources of variations in capture rates associated with station and volunteer are combined into a single source of variance that can be captured with a single random effect and maximum statistical power in the analysis.

Each day of work will comprise the same sequence of 6 one-hour collection periods (3 in the morning and 3 in the evening) as the procedures for objectives 1 to 4. Each of these one-hour periods for a given day will be randomly allocated without replacement to one of 6 angles (0°, 60°, 120°, 180°, 240° and 300°, relative to North), which will be the same for all 8 catching stations for that day and hour period. Each hour, the chair of the catcher and the mosquito electrocuting grid he uses will be rotated together around the centre of the catching station to face in that particular direction, so that all possible orientations relative to wind direction (measured with a miniature weather station placed nearby) are represented in the resulting dataset.

Based on the sample size calculations described in section A.9, we will repeat 6 times the 8×8 Latin square design described above for a single replicate in a single block of 8 catching stations, by repeating it in 6 distinct blocks in different parts of the Haitian field site (Probably Gressiers just outside of Port au Prince), where all 18 participating households will each be provided with 2 freshly-prepared transfluthrin emanators at the outset of the experiment. Overall, one full round of this experimental design will take 48 days of field work (8 days per block and rotation replicate × 6 blocks and replicates) that will be distributed across a working period of 2 months to allow personnel time to rest and attend to other commitments. Three such two-month rounds of evaluation will be conducted over the six months of the Haitian summer, so that variations in efficacy arising from weather conditions and loss of active ingredient can be captured over the full relevant duration of the transmission season for mosquito-borne illnesses in the Caribbean. While the experimental design for objective 5 is different from objectives 1 to 3 in terms of replication, rotation, and the shape and size of the emanator devices, the dose of transfluthrin applied will be identical, and the number of volunteers has been adjusted to the experimental design. The procedures each individual volunteer will participate in each working day will therefore be identical to those for objectives 1 to 3.

Note that the 6 treated emanators distributed to household in each block will only be used in these entomological efficacy evaluation experiments for only 8 days of every two-month round of assessment. For the remainder of each 2-month evaluation cycle, the emanators will be used freely by the households to whom they were given, following advisory discussions with the research team on how safely and effectively deploy them. Therefore, these assessments of duration of protective efficacy in Haiti will differ from those measured in Tanzania to address

objectives 1 to 3 in that the emanators will have been exposed to representative conditions of routine household use in a relevant low income tropical setting.

Objective 6: The 6 treated emanators distributed to household in each block will only be taken back from them ad used in these entomological efficacy evaluation experiments (Objective 5) for only 8 days of every two-month round of assessment. For the remainder of each 2-month evaluation cycle, when they are not being assessed entomologically, the emanators will be used freely by the households to whom they were given, following advisory discussions with the research team on how safely and effectively deploy them. Nevertheless, they will also be encouraged to try using them in creative ways, so long as they do not open the protective holder or use it in any way that would allow direct physical contact with the treated fabric within it. As an illustrative example, the research team will explain how one of the investigators places such a device beside the front door of his house at night to prevent house entry by the *Culex* mosquitoes that he has observed routinely wait to enter when it is opened early in the morning[23]. Participants will be encouraged to utilize the emanators, within the bounds of the safety instructions provided, in whatever way they perceive to be optimal in terms of convenience and protection against mosquito bites. Each participating household will be asked to record what they perceive to be the most and least effective use practices with the disposable cameras they will be provided with for this purpose, as per our previous *Photovoice* (PV) surveys[24-27] in Dar es Salaam[28]. One participant in the photographic component of this study will be recruited per household, who will be responsible for taking photos on behalf of the entire household. Before using these cameras, participants in this component of the study will take part in a short training meeting to explain the subject matter of the survey, the principles of personal data confidentiality protection, and outline acceptable ways of using the camera without compromising the safety, privacy, or other rights of individuals or the community as a whole [28]. This two to three-hour meeting will be involve a series of explanations, questions and discussions, structured according to the guideline document provided in Annex 12. This guideline document was prepared based on existing literature recommendations for best practices [29, 30], adapted to these two distinct contexts based on our extensive combined experience of Tanzania and Haiti.

Each household will be visited once every two months when the efficacy experiments are undertaking, and surveyed with a very brief semi-structured questionnaire to assess their level of satisfaction with the perceived level of protection against mosquito bites. All consenting adult household members will be surveyed on each occasion. Perceived satisfaction will be recorded numerically as a graded scale from 0 to 5, with separate scores recorded for indoor and outdoor exposure. At the start of each visit to the block, any fully-used disposable cameras will be collected so that the photos can be developed in duplicate, with one of these duplicates will be returned to the household within a few days[28] while the other is retained by the research team in case of loss before the Photovoice group discussions (PVGDs) described below.

At the end of the study, four focus group discussions (FGDs) (one voluntary participant of each gender from each of the 3 participating households in 3 blocks per group) will be conducted in each experimental block. The participants for the focus group discussions will include adult male and female in separate groups, and these four groups will be interviewed separately to enhance participation. These semi-structured discussions will be conducted in Haitian Creole using a brief topic guides (Annex 9) to obtain thematic insights into how they perceived the value, advantages and disadvantages, affordability and practicality of these devices. At the end of these discussions, the interviewers will also canvass the participants for advice on how best to take this technology forward through further possible product modifications, programmatic evaluation and operational research (Annex 9). For each focus group discussion, a facilitator will coordinate discussion and an observer will take notes on expressed verbal and non-verbal forms of communication.

Once all the pictures have been developed and returned to the photographer participants, they will be engaged in a two-stage process of participatory analysis; selecting photographs for discussion and then contextualizing or storytelling. In the first stage, developed pictures will be given back to photographers at the end of the study, each of whom will be given approximately one week to select what he/she considered to be his/her 10 best or most significant photographs. By selecting photographs for discussion, participants will lead the overall direction of subsequent PV group discussions (PVGDs) [26, 27]. The second stage will consist of contextualizing, or telling stories about what the photograph meant to the photographer, during the PVGD. PVGDs. Each participant will display his/her photographs on a table, introduce them to the group, narrated the meaning of his/her photographs, and explain his/her interpretation of the images (Annex 11). These PVGDs will be conducted informally, but based on an adapted version of the SHOWeD model [26, 27]. At this stage of the discussion, each photographer will identify different themes that emerged after re-examining the contents of their photographs and remembering where, when and why they took them. This will be followed by a more specific discussion of the advantages, disadvantages and limitations of these devices, factors influencing their use, and ideas for improving the devices themselves or for optimal delivery and maintenance in the future (Annex 11). At the end of the discussion, the participants will select

the 10 best pictures out of all of the photographs taken.

All interviews and group discussions will be conducted in Haitian Creole and digital audio recordings of the FGDs and PVGDs will be made. These recordings will be subsequently transcribed verbatim (with identifiers removed), and translated into English, as Microsoft Word® documents.

A.6 **PARTICIPANTS**: Please state the number of research participants to be recruited. If you are unable to give precise figures, please give estimates.

**A.6.1 AGE/SEX**	**Neonates (<28 days)**	**Infants (1-11 months)**	**Young children (1-9 years)**	**Adolescents (10-18 years)**	**Adults (>18years)**
**Males**	1	3	20	20	24
**Females**	1	3	20	20	24

A.6.2 **ELIGIBILITY CRITERIA**

**Inclusion Criteria**	**Exclusion Criteria**
Only adult males (≥18 years) and adult females of non-child-bearing age (≥50 years) will be recruited as experimental mosquito catchers. Entire households will be recruited for objectives 5 and 6 as representative end users of the emanator devices.	Age <18 years for males or <50 years for females for mosquito catches. No exclusion criteria for household recruited as representative end users of the emanator devices.

Justification for eligibility criteria – if you are excluding a particular group you should justify their exclusion.

Minors are excluded as participants in experimental mosquito catching activities, as this vulnerable population subset is not required to address the overall goal, which should be generalizable to the entire population without their involvement as participants. Women of child-bearing age are excluded to comprehensively avoid any risk of infection with Zika, malaria or any other vector borne pathogen to which pregnant women are particularly vulnerable. As the emanator devices are intended to protect entire household rather than just specific, deliberately targeted subsets of members, no exclusion criteria are applied to the recruitment of households in this study as representative end users.

A.6.3 **VULNERABLE GROUPS:** Please identify vulnerable groups that will be included in this study. Also state how you will minimise any harm to each group identified.

None

A.6.4 **RECRUITMENT PROCESS:** Please detail the procedures for how you will be approaching each group of participants to take part in the project. Where will recruitment take place? Who will be responsible for recruitment of participants?

In Tanzania, we will first identify sensitize the ward leaders about our study by visiting them in their offices, and then discussing with them where suitable open plots of land may be found using participatory mapping tools we have previously described [22, 31, 32]. We will then visit and examine candidate study locations and discuss with relevant local neighbourhood (*Mtaa*) chairpersons, followed by the representatives of Ten-Cell Unit (TCU) housing cluster(s) ((*ma*)*shina* [31]) where those plots are located. A TCU typically comprises only 10 to 20 houses, so most TCU representatives know and can engage all households within their housing cluster, so in our experience this is an ideal mechanism for engagement of local communities with very fine scale geographic specificity [22, 31-35].

In Haiti, we will first contact the director of the Haitian department (administrative unit) public health office by visiting them in their office to explain the project and get their support. We will work initially in field sites used by the University of Florida and/or University of Notre Dame. We will work with the local community leaders such as the community Mayors, the Haitian CASEC (Conseil d'Administration de la Section Communale/Board of Directors of the Communal Section) and public health officers, community health workers and local doctors of clinics to work in these communities. This will involve visiting the community and determining suitability informing the community in immediate vicinity of the work that will be done and why there areas was selected.

With the assistance of the relevant local community leaders, described above for each country, we will then recruit owners and/or users of the land to be used for these experiments, who will be first informed of the purpose and procedures of the study, as well as the potential risks and benefits, and their rights to participate, refuse and withdraw, and then asked to provide informed consent to use these plots as experimental blocks as described in the methods

section above.

Details of opportunities to participate in the research, as one of the individuals sitting in an electric grid trap to attract and capture mosquitoes, will then be disseminated through these selected, local community leaders, complete with details of the purpose and procedures of the study, inclusion and exclusion criteria, potential risks and benefits, the rights to participate, refuse and withdraw, and compensation for time, inconvenience and discomfort. In addition to providing participation opportunities to residents of the communities who will host the research, which are often viewed as casual employment opportunities, previous experience with community owned resource persons for larval surveillance in the Tanzanian study site has conclusively demonstrated that recruiting through such fine-scale local government systems results in improved personnel performance [33, 36].

A.7	**OUTCOMES:** What is the primary outcome measure for the study?<br>What are the secondary outcome measures? (if any)

Primary Outcomes:

1. Relative decrease of biting rates by *Aedes aegypti* mosquitoes upon users of transfluthrin-treated strips, which are vectors of Dengue, Chikungunya and Zika.
2. Duration of protection after treatment, with 80% reduction of mosquito bites defined as the minimum acceptable level of protection.

Secondary Outcomes:

3. Relative decrease or increase of biting rates by *Aedes aegypti* mosquitoes upon non-users of transfluthrin-treated strips nearby users of transfluthrin-treated strips.
4. User-perceived levels of protection reported by representative end user households.
5. User-perceived best practices for routine use and ideas for optimal further development and deployment.

A.8	**MAJOR METHODS OF ANALYSIS:** What are the major methods you intend to use to analyse the data?

Objective 1: The impact of treated Hessian strips on risk of exposure to mosquito bites over each two-month survey cycle will be quantified for *Aedes aegypti* and any other common day-biting mosquito species caught, by fitting generalized linear mixed models (GLMMs) with Poisson distributions to the number of mosquitoes caught at each station on each day for the user of a strip. The treatment status combination of the strips of the users (T opposite C versus C opposite C, rather than C opposite T, which may increase biting rates due to diversion) will be treated as the independent variable of primary interest. Nuisance variables such as station, individual human subject, date and direction will be treated as random effects. Results will be presented as similarly to figures 1 and 2.

Objective 2:

First, a separate Poisson-distributed GLMM will be fitted to each subset of data comprising one full two-month experimental replicate of the study design, exactly as described above for objective 1. Furthermore, an additional GLMM will be fitted to the full 12 months of longitudinal data, encompassing 6 full experimental replicates over one full year, with time since treatment with transfluthrin (but not the placebo) included as a continuous variable, to allow estimation of the efficacy decay rate. Results will be presented similarly to figures 1 and 2.

Objective 3: In order to quantify any protection over distance, or diversion of mosquitoes to unprotected non-users, separate GLMMs with Poisson distributions will be fitted to the subset of data from non-users at each distance from non-users. Catches by non-users at each of a range of distances will be treated as the dependent variable, with treatment status of the nearby user's strip (T or C) as the independent variable of primary interest. Results will be presented similarly to figure 3.

Objective 4: The impact of each different format and size of treated Hessian strips on risk of exposure to mosquito bites will be quantified for *Aedes aegypti* and any other common day-biting mosquito species caught, by fitting generalized linear mixed models (GLMMs) with Poisson distributions to the number of mosquitoes caught at each station on each day.

The allocated experimental treatment (Format × transfluthrin treatment for the first experiment, or emanator size for the second experiment) for that station on that day will be treated as the independent variable of primary interest. Nuisance variables such as station, date and direction faced by the volunteer will be treated as random effects. Results for the second experiment, for which the independent variable of interest fit along a continuous scale, will be presented similarly to figure 4.

Objective 5: First, a separate Poisson-distributed GLMM will be fitted to each subset of data comprising one full two-month experimental replicate of the study design, exactly as described above for objective 1. Furthermore, an additional GLMM will be fitted to the full 6 months of longitudinal data, encompassing 3 full experimental replicates, with time since treatment with transfluthrin (but not the placebo) included as a continuous variable, to allow estimation of the efficacy decay rate. Results will be presented similarly to figures 1 and 2.

Objective 6: A mixture of both qualitative (Thematic analysis of unstructured content of monthly questionnaires, associated photographs and final FGDs) and quantitative methods (Frequency distribution analyses, non-parametric rank tests, and GLMMs) will be used to analyse and interpret these data, as we have on previous occasions in relation to other mosquito control service delivery issues[28, 33, 36].

The semi-quantitative data collected, in the form of rated categorical levels of satisfaction with the protection provided by transfluthrin emanators, will be primarily be analysed graphically by comparing the mean/median and distributions of these recorded perceptions with the quantitative entomological estimates (Objective 4). Spearman's rank tests and GLMMs will also be used to test for associations between these subjectively perceived and objective measured indicators of protection. A specific goal of this analysis is to at least approximately identify thresholds of protective efficacy or crude biting exposure that are consistently considered satisfactory by end users.

For the qualitative FGD and PV-FGD data, analysis will be conducted in an iterative manner, adopting a framework approach involving five key stages of analysis [37]: 1) familiarisation through transcription and reading of data; 2) identifying a thematic framework, developed from a combination of *a priori* questions and issues that emerged during the familiarisation stage; 3) indexing, also known as coding, by applying the thematic framework to classify the data; 4) charting, by creating tables from the coded data; and 5) mapping and interpretation; searching for patterns and associations and testing potential explanations and interpretations in terms of logical plausibility and consistency with the full data set. To ensure triangulation of different perspectives, the research team will regularly findings that emerged to optimize the process through consensus.

**A.9**	**SAMPLE SIZE:** Please justify your choice of sample size (as described in A.6.1). Please ensure that the sample size calculation is based on the primary outcome measure as detailed in A7.

Objectives 1 to 3: The minimum number of volunteers (only 32) is determined primarily by the chosen number of independent experiment blocks needed to achieve the required sample size (8 replicate experiment blocks), the minimum number of volunteers required per rotation through an experimental block (4), and the decision to recruit volunteers locally for each block, rather than use the same volunteers in all experimental blocks. The number of experimental blocks to use was determined by the following sample size calculations.

The number of independent replicate experimental blocks was calculated based on the following assumptions:
1. Eighty percent power (1 - β = 80%) for detect an effect with a Type I error probability of one in twenty (P=5%).
2. A mean mosquito attack rate of 20 mosquitoes caught per person per day of experimentation in the untreated negative controls near another untreated control (Both C observations in C1-C2). The corresponding minimum intolerable attack rate on users of untreated control strip near users of treated strips (Both T observations in C1-T2 and T1-C2) is therefore 20 x 125% = 25 mosquitoes caught per person per day of experimentation.
3. Minimum sample size should be determined by potential risks rather than benefits. Given the long-standing safety record of transfluthrin, the primary risk associated with use of these emantors is assumed to be re-distribution of mosquito biting activity onto unprotected non-users, which could arise from the diversion of deterred mosquitoes away from users to non-users, thus exacerbating existing inequities in personal protection. A maximum tolerable effect size for increased biting rates upon non-users of ≥25% was assumed and used to calculate the sample size, rather than the much greater protective effect that these emanators need to provide to users (≥80%).
Assuming a Poisson distribution of these count data, and then applying Lehr's equation [38], the following minimum number of observations was estimated for each full round of replication:

$$N = 4 / [(\lambda_1^{1/2}) + (\lambda_2^{1/2})]^2 = 4 / [(20^{1/2}) + (25^{1/2})]^2 = 14.4$$

However, because of the experimental design of each three day replicate in each experimental block, two observations will be made for each treatment (Two C observations in C1-C2 and two T observations in T1-C2 plus C1-T2). The estimated minimum number of experimental blocks required is therefore N =14.4 / 2 = 7.2, which was rounded upwards to yield a sample size of 8 independent replicate experimental blocks.

Objective 4: For the purposes of sample size calculations, we assume the same mean mosquito density of 20 per person per day, a minimum efficacy for the optimal format of 80% (resulting in a biting density of 4 per person per day) that should be distinguishable from another format with an efficacy of 60% or less (8 or more mosquitoes per person per day). Assuming a Poisson distribution of these count data, and then applying Lehr's equation [38], the following minimum required number of independent observations was estimated:

$$N = 4 / [(\lambda_1^{1/2}) + (\lambda_2^{1/2})]^2 = 4 / [(4^{1/2}) + (8^{1/2})]^2 = 5.8$$

Objectives 5 and 6: For the purposes of sample size calculations, we assume the same mean mosquito density of 20 per person per day as for the previous objectives, a minimum efficacy for a freshly-treated emanator of 80% (resulting in a biting density of 4 per person per day) that should be distinguishable from an older emanator for which the efficacy has declined to 60% or less (8 or more mosquitoes per person per day). Assuming a Poisson distribution of these count data, and then applying Lehr's equation[38], the following minimum required number of independent observations was estimated:

$$N = 4 / [(\lambda_1^{1/2}) + (\lambda_2^{1/2})]^2 = 4 / [(4^{1/2}) + (8^{1/2})]^2 = 5.8$$

**A.10**	**QUALITY ASSURANCE:** Quality assurance is more than just for data analysis – it should include procedures in design and data collection. What procedures are in place to ensure the quality of the data? What consideration has been given to methods of analysis to ensure efficiency of data use?

Mosquito samples will be packed, labelled and stored at IHI in locked sample storage freezers. Data collected in paper form will also be stored securely in locked filing cabinets in a room with restricted access. Electronic data will be stored on secure, routinely backed up servers at IHI. This central server uses a new, innovative schema [39] which allows any entomological data to be stored in only 4 entomology-specific data tables with predefined relational linkages (Figure 9), enabling rapid, even automated synthesis and analysis.

SECTION B

PROCEDURES AND PATIENT CARE

**B.1**	**PROCEDURES**	Please detail any clinical or other research procedures to which participants will be subjected.
**Procedure**	**To be carried out by:**	**Who is the person employed by?**
Mosquito catches with electric grid traps in Tanzania and Haiti	One to-be-recruited BSC graduate Research Assistant at each study site.	Tanzania: Dr Nicodem Govella at IHI, with additional supervisory support from Dr Gerry Killeen<br>Haiti: Mr Frederick, with additional supervisory support from Dr Daniel Impoinvil, Dr Gerry Killeen and Dr Cyrille Czeher.
Social science questionnaire surveys, focus group discussions, and *Photovoice* surveys in Haiti	One to-be-recruited BA graduate Research Assistant in Haiti	Mr Frederick, with additional supervisory support from Prof Thom Eisele, Dr Thom Dreutz, Prof Katherine Andrinopoulos, Dr Daniel Impoinvil, Dr Cyrille Czeher and Dr Gerry Killeen

**B.2**	**STANDARD PATIENT CARE:** Please explain if the procedures outlined in B.1 are part of the normal clinical work of the staff who will perform the procedure. If not applicable to your study, please write a sentence stating that this is not a clinical study.
This is not a clinical study and involves no patients or health care service provision setting of any description.	

**B.3**	**END OF TRIAL TREATMENT:** For intervention trials, what steps will be taken to make successful interventions or treatment available to all trial participants at the end of the trial? If not applicable to your study, please write a sentence stating that this is not a clinical study.
Not applicable: No registration or other approval for general public use will be in place at the end of the study.	

**B.4**	**TRAINING**	Please indicate the basis on which the persons identified in B.1 are thought to be competent to carry out these procedures. List any training of staff which will be required prior to commencement of the study.
**Staff Member**	**Experience/competencies**	**Training Required**
To-be-recruited Research Assistants	BSc graduates, but also most probably recruited from existing, experienced technical staff at IHI and PNCM, many of whom have extensive experience of this kind of work	If the recruited individual does not already hold a certificate in the protection of human subjects in medical research from the NIH online course, s/he will be required to complete this course and present their certificate before being employed on this project.

SECTION C
RISKS AND CONSEQUENCES

**C.1**	**ADVERSE EFFECTS, DISCOMFORT OR RISKS:** Outline the potential adverse effects, discomfort or risks that may result from the study for participants, investigators and members of the public and how you will minimise them.
**C.1.1 Participants**	Potential adverse effects, discomfort or risks<br><br>The concentrations of tranfluthrin vapour released by these emanator devices has been measured as only 0.00013 mg/m^3, comparing very well (<1/1000th) with its registered acceptable exposure concentration of 0.5 mg/m3 [40], and are therefore considered to be of negligible risk.<br><br>Electric Grid Trap is an exposure-free tool for catching host-seeking mosquitoes, meaning that it catches and kills mosquitoes before they can bite, so human volunteers are not exposed to increased risk of mosquito-borne infections. The tool is comprised of 4 panels with electrified wire mesh spread 5mm apart forming a square. Volunteers sit on chair with their legs placed in a square frame while the rest of body protected from mosquito bites by wearing hats and shirt/jacket with long sleeve (Figure 7), which is obviously very uncomfortable.<br><br>There are minor risks to privacy and security associated with disseminating photographs of the exterior or interior of participants' houses or their contents. Also, minor risks to privacy and security may be incurred by presenting the facial features or other identifiable personal information in such published photographs. The only other personal information to be collected from any participants will be their name, age and gender, but none of these data will be published, disseminated or accessible to the public.
	Steps to be taken to minimise adverse effects, discomfort and risks<br><br>The square PVC/wooden frame is lined from within with insulating plastic fibre mesh, which serves not only for protection of mosquito entry, but also protect volunteers limbs against any possible contact with the exterior electrified wires, which carries low voltage direct current for electrocuting mosquitoes.<br><br>The participants will be given every opportunity to discuss the study and any questions they may have will be answered. Informed consent will be documented and participants will be allowed to withdraw at any stage. In the case of withdrawal of a mosquito catcher prior to completion of the study, we will replace them as soon as possible with a new recruit and continue with the experimental design as described above. All these studies will be conducted during hours of broad daylight, when *Anopheles* malaria vectors are no longer active, and volunteers will be full protected against daytime-biting *Aedes agypti* by the electric grids and protective clothing. Thus, unlike previous mosquito exposure studies we have conducted, no drug prophylaxis against malaria, or regular screening for malaria infection, is required or will be provided to volunteers. Also, only adult males (≥18 years) and adult females of non-child-bearing age (≥50 years) will be recruited as volunteers, to comprehensively avoid any risk of infection with Zika, malaria or any other vector borne pathogen to which pregnant women are particularly vulnerable.<br><br>Risks associated with disseminating photographs of the exterior and interior of participants' houses will be mitigated by seeking for written permission before any specific photograph is published or shared with anyone other than the investigators, with any facial features or other identifiable personal information masked out if requested by the participant (Annex 3). At the request of the participants, we will also mask out any personally identifiable or sensitive elements of the photographs, such as their belongings or security precautions. The only other personal information to be collected from any participants will be their name, age and gender, all of which will be kept confidentially, with original hard copies stored in locked filing cabinets while all electronic files containing this data, as well as the computers it is stored on, will be password-protected.
**C.1.2 Investigators**	Potential adverse effects, discomfort or risks<br><br>No investigator will be exposed to any higher air concentration of transfluthrin vapour than the participants during the experiments. As described above for the participants, they will therefore be working >1000 fold below the maximum acceptable limits for chronic exposure to this insecticide, which

	is considered pose negligible risk after decades of use in a plethora of common household products. However, there are minor risks of acute transfluthrin exposure when formulating the emanators using initially undiluted transfluthrin stock.
	Steps to be taken to minimise adverse effects, discomfort and risks<br><br>All investigators will wear gloves, protective clothing, safety spectacles and face masks when preparing the emanators.
**C.1.3 Members of the public**	Potential adverse effects, discomfort or risks<br><br>Exposure to transfluthrin while visiting the participating households in Haiti.
	Steps to be taken to minimise adverse effects, discomfort and risks<br><br>As described in section C1.1, the transfluthrin vapour concentrations involved are considered to pose negligible risk.

**C.2**	**CONSEQUENCES FOR LOCAL HEALTH SERVICES**
**C.2.1**	**IMPACT ON LOCAL SERVICES:** What demands will this research place on local health services?
None	
**C.2.2**	**MINIMISING IMPACT ON LOCAL SERVICES:** Detail how the design of the research project takes into account the demands described above in C.2.1
Not applicable	

SECTION D

PRIVACY AND INFORMED CONSENT

D.1	**INFORMED CONSENT (please pay particular attention to the guidance notes for this section)**
D.1.1	**OBTAINING INFORMED CONSENT**: Please give details of how you will obtain informed consent. You must include details of (i) information given to participants, (ii) who will deliver the information and (iii) consideration of local circumstances. Please note that reference of transport of samples to another country must be included in the consent forms. Please also give special consideration to whether proxy consent is required (for those lacking capacity to consent, minor etc) and give details as to how this would be obtained.

The informed consent process will be explained and obtained in the local language, namely *kiSwahili* in Tanzania and French or Haitian Creole in Haiti, and volunteers will be allowed to withdraw at any time. Participants will still be reminded about the risks of obtaining malaria every Monday morning throughout the duration of the study. The information will be delivered, and informed consent obtained, in the presence of the relevant local community leader (described for each country in section A.6.4) of each participant, who will act as the signatory witness**.**

D.1.2	**CONSTRAINTS:** Please outline any potential constraints to consent and indicate how you will reduce the impact of these constraints.

D.2	**ASSISTANCE:** Please outline any assistance (financial or otherwise) that will be offered to potential participants or individuals in return for their participation in this research.

The participants will not benefit from participation, other than remuneration for their time and discomfort at a rate of Tsh20,000 per day in Tanzania or $15 per day in Haiti (PNCM uses routinely remunerates participants with US$ because of the instability of the Haitian Gourde). These rates have been standardized across IHI and PNCM, respectively, to strike a balance between being enough to provide fair compensation for time and discomfort, without inducing volunteers to participate despite any reservations they may have.

The outcome from this study will have direct, significant policy implications relevant to the implementation of vector control and disease prevention strategies. If these emanators prove to be effective against *Aedes aegypti*, they can not only be deployed to protect against any Zika outbreaks that may occur in the future, they can also be deployed now to provide simultaneous, broad-spectrum daytime protection against ongoing transmission of Dengue and Chikungunya by the same vectors, as well as night time protection against other mosquitoes carrying malaria and filariasis.

D.3	**PRIVACY AND CONFIDENTIALITY:** Please describe how participant privacy and confidentiality will be maintained during data collection, analysis and storage. Please include what will be collected (data, samples etc.) and where and for how long it will be stored.

No sensitive personal data will be collected. Participants' full names will not be recorded in writing at any stage anywhere other than the informed consent forms, and they will instead be assigned anonymized participant numbers so that their identities cannot be established from the data.

D.4	**DISSEMINATION:** Please outline what plans you have for dissemination of results

Results from this project will be published in open access peer reviewed journals to engage wider international scientific community. These results will also be presented at meetings of the National Steering Committee for Neglected Vector-Borne Diseases of the Ministry of Health, Community Development, Gender, Elderly and Children, which will allow disseminating information to wide range of government stakeholders, up to the level of the cabinet, as well as print, radio and TV media. Similarly in Haiti, these results will also be presented to government stakeholders in the Ministry of Public Health and Population, foreign and local partners of the Ministry of Public Health and Population, and local media such as print, radio and TV media. The results will also be presented directly to the residents of the study areas in both Tanzania and Haiti, through community meetings. The results will also be disseminated prospective local commercial partners, who could potentially register and supply emanator devices and pre-diluted transfluthrin sachets for treating and retreating them.

SECTION E

MAJOR ETHICAL ISSUES

E.1	**MAJOR ETHICAL ISSUES**<br>Outline what you consider to be the major ethical issues involved in this research.<br>Please indicate how you plan to deal with these ethical issues.

We do not foresee any major ethical issues. First, the active ingredient already registered for use all over the world, including Tanzania, and experimental registration in Haiti will also be secured before the study begins there. Also, these emanators proven to release active ingredient at less than 1/1000th of the maximum acceptable concentration [40]. Second, the electric grids to be used prevent exposure to mosquitoes, so participants are at lower risk than they would be during their normal activities, and have been specifically designed to pose no risk and only very mild discomfort to any individual exposed to this very minor electrical shock [8].

The participants will be given every opportunity to discuss the study and any questions they may have will be answered. Informed consent will be documented and participants will be allowed to withdraw at any stage. In the case of withdrawal of a mosquito catcher prior to completion of the study, we will replace them as soon as possible with a new recruit and continue with the experimental design as described above. All these studies will be conducted during hours of broad daylight, when *Anopheles* malaria vectors are no longer active, and volunteers will be full protected against daytime-biting *Aedes agypti* by the electric grids and protective clothing. Thus, unlike previous mosquito exposure studies we have conducted, no drug prophylaxis against malaria, or regular screening for malaria infection, is required or will be provided to volunteers. Also, only adult males (≥18 years) and adult females of non-child-bearing age (≥50 years) will be recruited as volunteers, to comprehensively avoid any risk of infection with Zika, malaria or any other vector borne pathogen to which pregnant women are particularly vulnerable.

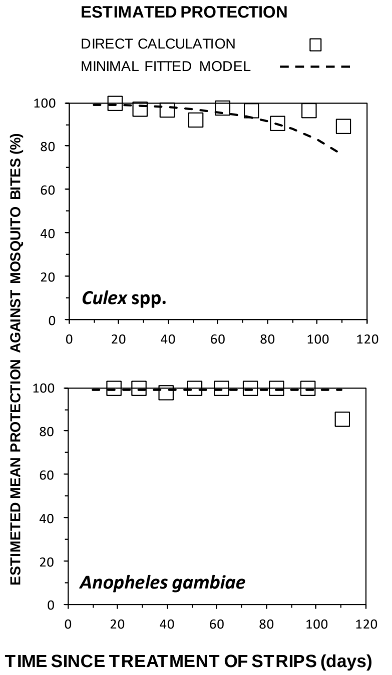

Figure 1. Protection provided by Hessian strips treated with 10ml of Transfluthrin against *Culex* spp. and *Anopheles gambiae* mosquitoes biting outdoors at night in urban Tanzania

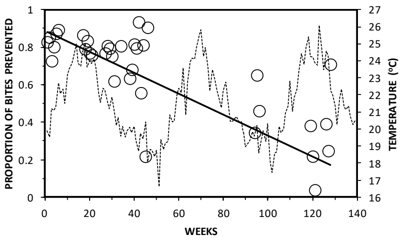

Figure 2. Duration of protection conferred by Hessian strips treated with 10ml of transfluthrin against wild, nocturnal, outdoor-biting *Anopheles arabiensis*, under full open field conditions in rural southern Tanzania.

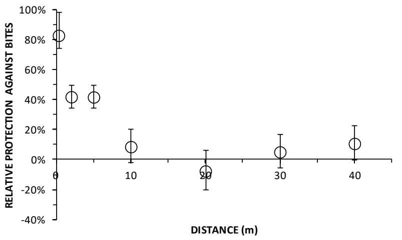

Figure 3. Protective effect of transfluthrin-treated Hessian strips against outdoor-biting *An. arabiensis* for the users (distance 0m) and non-users at a range of distances from a user, under full open field conditions in rural southern Tanzania.

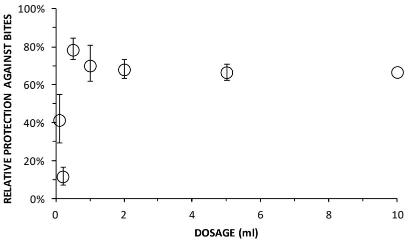

Figure 4. Dose-response curve for transfluthrin-treated Hessian strips under full open-field conditions in rural southern Tanzania

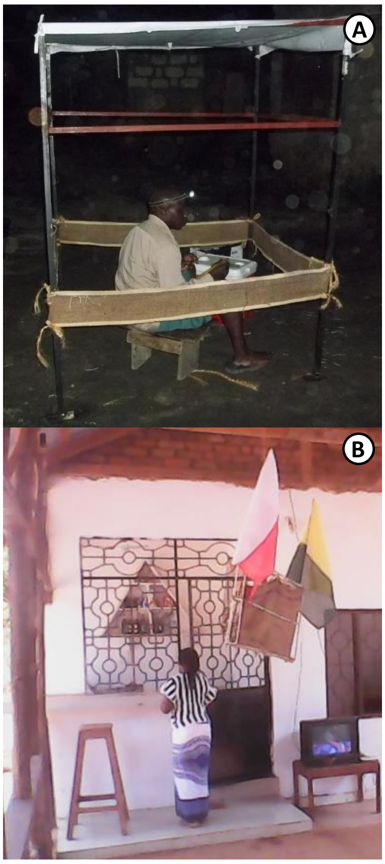

Figure 5. The original prototype of the transfluthrin-treated Hessian strip that will be used in this project for experimental design purposed (A) and the more convenient, scalable format now available hanging in a public bar.

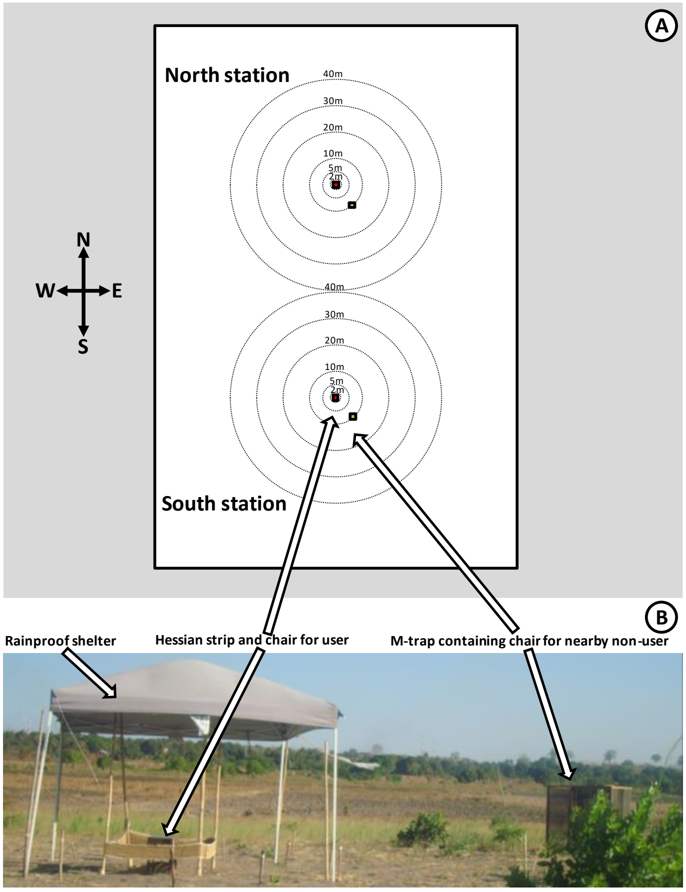

Figure 6. A schematic example of the open-field experimental design used to generate the data presented in figures 2, 3 and 4. Note that both human landing catches by strip users, and large cage trapping by non-users will be replaced by electric grid traps in the study proposed herein.

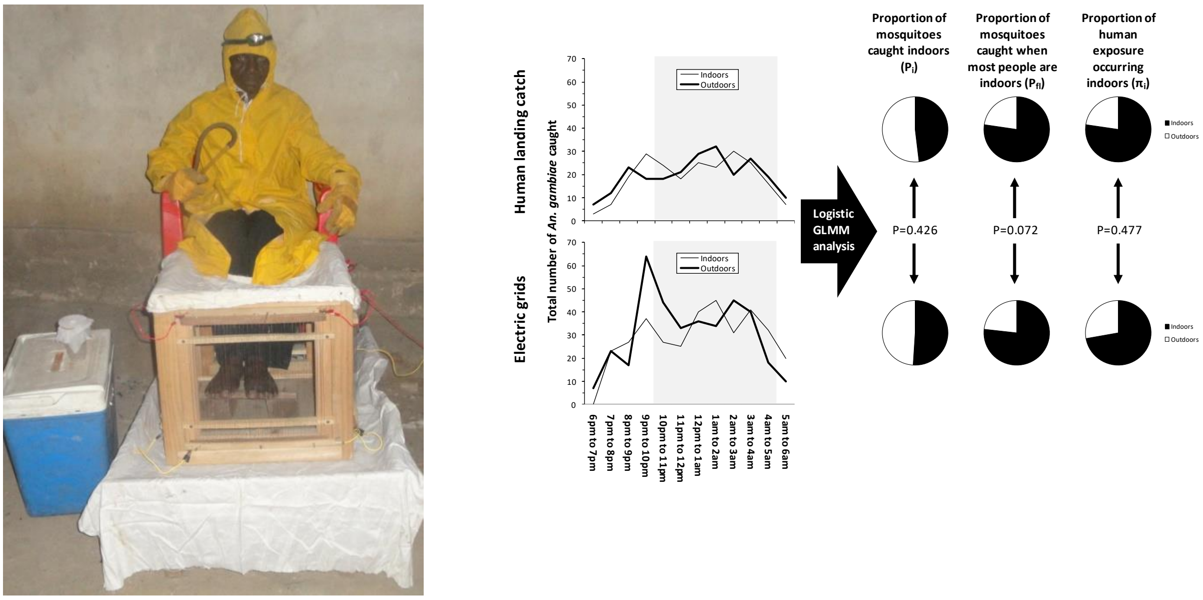

Figure 7. The left-hand panel illustrates electrocuting grids in use with a human volunteer, while the right hand panel illustrates how these traps gives estimates of key behavioural determinants of malaria vector control impact that are equivalent to gold standard human landing catch.

Figure 8. Effect of temperature on efficacy of hessian strip transfluthrin emanators against biting *Culex* mosquitoes. Effect of daily mean temperature for the day preceding each night of experimentation upon efficacy of transfluthrin emanators. Each data points represent the mean relative rate mosquito capture by human landing catches conducted by users of treated emanators, averaged within each one degree temperature range category.

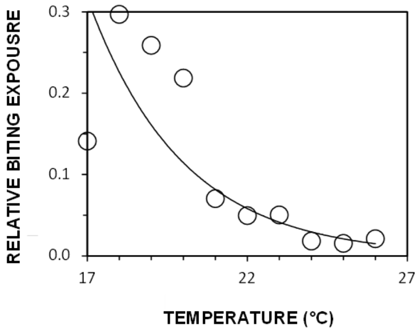

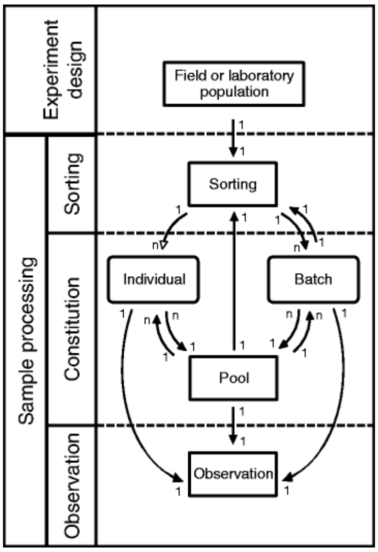

Figure 9. Data collection is based on a simple foundation of recording the experimental design followed by sample processing. Sample processing (dashed boxes) involves the sorting and observation of mosquito samples. As mosquito biology experiments are highly variable in structure, there are many possible ways in which to move between the generic schemas. The arrows indicate the direction and function (e.g., one-to-many: 1…n) of the relationships between the entities.

References

1. Ayres CF. Identification of Zika virus vectors and implications for control. Lancet Infect Dis. 2016. Epub 2016/02/09. doi: 10.1016/S1473-3099(16)00073-6. PubMed PMID: 26852727.
2. Faye O, Freire CC, Iamarino A, de Oliveira JV, Diallo M, Zanotto PM, Sall AA. Molecular evolution of Zika virus during its emergence in the 20th century. PLoS Negl Trop Dis. 2014;8(1):e2636. Epub 2014/01/15. doi: 10.1371/journal.pntd.0002636. PubMed PMID: 24421913; PubMed Central PMCID: PMC3888466.
3. Diallo D, Sall AA, Diagne CT, Faye O, Ba Y, Hanley KA, Buenemann M, Weaver SC, Diallo M. Zika virus emergence in mosquitoes in southeastern Senegal, 2011. PLoS One. 2014;9(10):e109442. Epub 2014/10/14. doi: 10.1371/journal.pone.0109442. PubMed PMID: 25310102; PubMed Central PMCID: PMC4195678.
4. Grard G, Caron M, Mombo IM, Nkoghe D, Mboui Ondo S, Jiolle D, Fontenille D, Paupy C, Leroy EM. Zika virus in Gabon (Central Africa)--2007: a new threat from Aedes albopictus? PLoS Negl Trop Dis. 2014;8(2):e2681. Epub 2014/02/12. doi: 10.1371/journal.pntd.0002681. PubMed PMID: 24516683; PubMed Central PMCID: PMC3916288.
5. Musso D, Cao-Lormeau VM, Gubler DJ. Zika virus: following the path of dengue and chikungunya? Lancet. 2015;386(9990):243-4. Epub 2015/07/22. doi: 10.1016/S0140-6736(15)61273-9. PubMed PMID: 26194519.
6. Ogoma SB, Ngonyani H, Simfukwe ET, Mseka A, Moore J, Killeen GF. Spatial repellency of transfluthrin-treated hessian strips against laboratory-reared *Anopheles arabiensis* mosquitoes in a semi-field tunnel cage. Parasit Vectors. 2012;5:54. Epub 2012/03/22. doi: 10.1186/1756-3305-5-54. PubMed PMID: 22433128; PubMed Central PMCID: PMC3338372.
7. Govella NJ, Ogoma SB, Paliga J, Chaki PP, Killeen G. Impregnating hessian strips with the volatile pyrethroid transfluthrin prevents outdoor exposure to vectors of malaria and lymphatic filariasis in urban Dar es Salaam, Tanzania. Parasit Vectors. 2015;8:322. Epub 2015/06/13. doi: 10.1186/s13071-015-0937-8. PubMed PMID: 26063216; PubMed Central PMCID: PMC4465323.
8. Maliti DV, Govella NJ, Killeen GF, Mirzai N, Johnson PCD, Kreppel K, Ferguson HM. Development and evaluation of mosquito-electrocuting traps as alternatives to the human landing catch technique for sampling host-seeking malaria vectors Malar J. 2015;14:502. doi: 10.1186/s12936-015-1025-4.
9. Freire CCdM, Lamarino A, Neto DFdL, Sall AA, Zanotto PMdA. Spread of the pandemic Zika virus lineage is associated with NS1 codon usage adaptation in humans (Online pre-print). BioRxiv. 2016;DOI:10.1101/032839.
10. Zanluca C, de Melo VC, Mosimann AL, Dos Santos GI, Dos Santos CN, Luz K. First report of autochthonous transmission of Zika virus in Brazil. Memorias do Instituto Oswaldo Cruz. 2015;110(4):569-72. Epub 2015/06/11. doi: 10.1590/0074-02760150192. PubMed PMID: 26061233; PubMed Central PMCID: PMC4501423.
11. Dick GW. Zika virus. II. Pathogenicity and physical properties. Trans R Soc Trop Med Hyg. 1952;46(5):521-34. Epub 1952/09/01. PubMed PMID: 12995441.
12. Dick GW, Kitchen SF, Haddow AJ. Zika virus. I. Isolations and serological specificity. Trans R Soc Trop Med Hyg. 1952;46(5):509-20. Epub 1952/09/01. PubMed PMID: 12995440.
13. Achee NL, Bangs MJ, Farlow R, Killeen GF, Lindsay S, Logan JG, Moore SJ, Rowland M, Sweeney K, Torr SJ, Zwiebel LJ, Grieco JP. Spatial repellents: from discovery and development to evidence-based validation. Malar J. 2012;11:164. doi: 10.1186/1475-2875-11-164.
14. Killeen GF. Characterizing, controlling and eliminating residual malaria transmission. Malar J. 2014;13:330. Epub 2014/08/26. doi: 10.1186/1475-2875-13-330. PubMed PMID: 25149656; PubMed Central PMCID: PMC4159526.
15. Hogarh JN, Antwi-Agyei P, Obiri-Danso K. Application of mosquito repellent coils and associated self-reported health issues in Ghana. Malar J. 2016;15:61. doi: 10.1186/s12936-016-1126-8.
16. Killeen GF, Moore SJ. Target product profiles for protecting against outdoor malaria transmission. Malar J. 2012;11:17. doi: 10.1186/1475-2875-11-17.
17. Killeen GF, Seyoum A, Gimnig JE, Stevenson JC, Drakeley CJ, Chitnis N. Made-to-measure malaria vector control strategies: rational design based on insecticide properties and coverage of blood resources for mosquitoes. Malar J. 2014;13:146.
18. Gloria-Soria A, Ayala D, Bheecarry A, Calderon-Arguedas O, Chadee DD, Chiappero M, Coetzee M, Elahee KB, Fernandez-Salas I, Kamal HA, Kamgang B, Khater EI, Kramer LD, Kramer V, Lopez-Solis A, Lutomiah J, Martins A, Jr., Micieli MV, Paupy C, Ponlawat A, Rahola N, Rasheed SB, Richardson JB, Saleh AA, Sanchez-Casas RM, Seixas G, Sousa CA, Tabachnick WJ, Troyo A, Powell JR. Global genetic diversity of Aedes aegypti. Mol Ecol. 2016. doi: 10.1111/mec.13866. PubMed PMID: 27671732.
19. Bowman LR, Donegan S, McCall PJ. Is Dengue Vector Control Deficient in Effectiveness or Evidence?: Systematic Review and Meta-analysis. PLoS Negl Trop Dis. 2016;10(3):e0004551. doi: 10.1371/journal.pntd.0004551. PubMed PMID: 26986468; PubMed Central PMCID: PMCPMC4795802.

20. Achee NL, Youngblood L, Bangs MJ, Lavery JV, James S. Considerations for the Use of Human Participants in Vector Biology Research: A Tool for Investigators and Regulators. Vector Borne Zoonotic Dis. 2015;15:89-102.
21. Mboera LE, Mweya CN, Rumisha SF, Tungu PK, Stanley G, Makange MR, Misinzo G, De Nardo P, Vairo F, Oriyo NM. The Risk of Dengue Virus Transmission in Dar es Salaam, Tanzania during an Epidemic Period of 2014. PLoS Negl Trop Dis. 2016;10(1):e0004313. Epub 2016/01/27. doi: 10.1371/journal.pntd.0004313. PubMed PMID: 26812489; PubMed Central PMCID: PMC4728062.
22. Dongus S, Nyika D, Kannady K, Mtasiwa D, Mshinda H, Fillinger U, Drescher A, Tanner M, Castro MC, Killeen GF. Participatory mapping of target areas to enable routine comprehensive larviciding of malaria vector mosquitoes in Dar es Salaam, Tanzania. Int J Health Geogr. 2007;6:37.
23. Ogoma SB, Mmando A, Kyeba JS, Horstmann S, Malone D, Killeen GF. A low technology emanator treated with the volatile pyrethroid Transfluthrin confers long term protection against outdoor-biting vectors of lymphatic filariasis, arboviruses and malaria. PLoS Negl Trop Dis. 2017;11:e0005455.
24. Iskander D. Re-imaging malaria in the Philippines: how photovoice can help to re-imagine malaria. Malar J. 2015;14:257.
25. Iskander D. Parasites, Power, and Photography. Trend Parasitol. 2016;32:2-3. doi: 10.1016/j.pt.2015.11.002.
26. Wang C, Burris MA. Photovoice: Concept, methodology, and use for participatory needs assessment. Health Educ Behav. 1997;24:369-87.
27. Wang CC. Photovoice: A participatory action research strategy applied to women's health. J Womens Health. 1999;8:185-92.
28. Makungu C, Stephen S, Kumburu S, Govella NJ, Dongus S, Hildon ZJL, Killeen GF, Jones C. Informing new or improved vector control tools for reducing the malaria burden in Tanzania: a qualitative exploration of perceptions of mosquitoes and methods for their control among the residents of Dar es Salaam Malar J. 2017;16:410. doi: 10.1186/s12936-017-2056-9.
29. Wang CC, Redwood-Jones YA. Photovoice ethics: perspectives from Flint Photovoice. Health Educ Behav. 2001;28(5):560-72. doi: 10.1177/109019810102800504. PubMed PMID: 11575686.
30. Bugos E, Frasso R, FitzGerald E, True G, Adachi-Mejia AM, Cannuscio C. Practical guidance and ethical considerations for studies using photo-elicitation interviews. Prev Chronic Dis. 2014;11:E189. doi: 10.5888/pcd11.140216. PubMed PMID: 25357257; PubMed Central PMCID: PMCPMC4215569.
31. Dongus S, Mwakalinga V, Kannady K, Tanner M, Killeen GF. Participatory mapping as a component of operational malaria vector control in Tanzania. Geospatial Analysis of Environmental Health2010. p. In Press.
32. Fillinger U, Kannady K, William G, Vanek MJ, Dongus S, Nyika D, Geissbuehler Y, Chaki PP, Govella NJ, Mathenge EM, Singer BH, Mshinda H, Lindsay SW, Tanner M, Mtasiwa D, de Castro MC, Killeen GF. A tool box for operational mosquito larval control: preliminary results and early lessons from the Urban Malaria Control Programme in Dar es Salaam, Tanzania. Malar J. 2008;7(1):20. PubMed PMID: 18218148.
33. Chaki PP, Dongus S, Kannady K, Fillinger U, Kelly A, Killeen GF. Community-Owned Resource Persons for malaria vector control: enabling factors and challenges in an operational programme in Dar es Salaam, Tanzania. Human Res Health. 2011;9:21.
34. Chaki PP, Mlacha Y, Msellemu D, Muhili A, Malishee AD, Mtema ZJ, Kiware S, Russell TL, Zhou Y, Lobo N, Dongus S, Govella NJ, Killeen GF. An affordable, quality-assured community-based system for high resolution entomological surveillance of vector mosquitoes that reflects human malaria infection risk patterns. Malar J. 2012;11:172.
35. Chaki PP, Kannady K, Mtasiwa D, Tanner M, Mshinda H, Kelly AH, Killeen GF. Institutional evolution of a community-based programme for malaria control through larval source management in Dar es Salaam, United Republic of Tanzania: A case study. Malar J. 2014;13:245.
36. Chaki PP, Govella NJ, Shoo B, Hemed A, Tanner M, Fillinger U, Killeen GF. Achieving high coverage of larval-stage mosquito surveillance: challenges for a community-based mosquito control programme in urban Dar es Salaam, Tanzania. Malar J. 2009;8(1):311. Epub 2010/01/01. doi: 1475-2875-8-311 [pii] 10.1186/1475-2875-8-311. PubMed PMID: 20042071; PubMed Central PMCID: PMC2806382.
37. Richie J, Lewis J. Qualitative research: a guide for social science student and researcher. London: Sage Publications; 2003.
38. van Belle G. Sample size. Statistical rules of thumb. Wiley Series in Probability and Statistical Series. 2 ed: Wiley; 2008. p. 27-51.
39. Kiware SS, Russell TL, Mtema ZJ, Malishee AD, Chaki P, Lwetoijera D, Chanda J, Chinula D, Majambere S, Gimnig JE, Smith TA, Killeen GF. A generic schema and data collection forms applicable to diverse entomological studies of mosquitoes. Source Code Biol Med. 2016;11:4. doi: 10.1186/s13029-016-0050-1. PubMed PMID: 27022408; PubMed Central PMCID: PMCPMC4809029.
40. EU Standing Committeeon Biocidal Products. Evaluation of active substances assessment report: Transfluthrin (insecticides, acaricides and products to control other arthropods). 2014.
