# Supplementary material for: Entomological assessment of hessian fabric transfluthrin vapour emanators as a means to protect against outdoor-biting Aedes after providing them to households for routine use in Port-au-Prince, Haiti
Source: PLoS One. 2024 May 28;19(5):e0298919. doi: 10.1371/journal.pone.0298919 (PMC11132518; doi:10.1371/journal.pone.0298919)
Supplement: S2 Protocol — See also S1 Protocol for the main protocol document itself in English and S3 Protocol for the approved protocol and annexes as translated into French and Haitian Creole. (PDF) [file pone.0298919.s003.pdf]

Affordable, scalable, low-technology transfluthrin emanators for protecting against transmission of Zika, Dengue and Chikungunya viruses.

ANNEX 1A (IN ENGLISH FOR TRANSLATION INTO KISWAHILI IN TANZANIA)

PARTICIPANT INFORMATION SHEET FOR MOSQUITO CAPTURE EXPERIMENTS

The black mosquitoes with white markings that commonly bite during daylight hours already transmit Dengue and Chikungunya viruses, which are common causes of fever, body pains and a number of other symptoms of illness here in Dar es Salaam. Furthermore, this mosquito is also known to transmit the Zika virus in other countries, which is thought to cause severe birth defects among children of pregnant women who become infected. Because they bite outdoors during the day when people are usually active, neither bed nets nor mosquito-proofed housing can provide satisfactory protection against these diseases. Unfortunately, mosquito coils and rub-on topical repellents for outdoor use only protect against mosquitoes for hours at a time, so they are too expensive and impractical for continuous use.

The team of investigators responsible for this study recently developed a low-technology transfluthrin emanator, which provides more than 90% protection for over 4 months against the night-biting mosquitoes in Dar es Salaam.

We therefore invite you to participate in a study to evaluate this new tool for preventing bites by these day-biting mosquitoes. The goal of the study is to demonstrate that this new, locally-designed reformulation of transfluthrin can provide affordable protection against these important mosquitoes for at least 6 months.

While transfluthrin-treated mosquito coils always burn, so that release of the insecticide is always associated with smoke, this simpler new device allows it to evaporate at normal air temperatures, so that it protects an outdoor space with repellent vapour that will not be visible to you. The device itself consists only of a strip of Hessian fabric, the material used to make sacks for storing and transporting cereals like maize and rice, into which a widely-used insecticide called *Transfluthrin* is impregnated. The transfluthrin insecticide used to treat these fabric strips costs less than Tsh500/= per strip, and the fabric strips can be safely hand-treated using only plastic gloves for protection. Transfluthrin has a very positive, long-standing safety record after decades of use all over the world. Transfluthrin is commonly used in repellent emanator devices like mosquito coils, has been registered for use as a repellent all across Europe, America, Asia and Africa for several decades, and is approved for experimental evaluation in Tanzania.

Several of the experiments in this study will measure how much protection various forms of these devices provide against mosquito bites. As a participant in these experiments, you will catch mosquitoes with an electric grid trap, comprised of a square plastic or wooden flame frame holding electrified wires.. The trap frame is lined with plastic mesh, to protect your limbs against contact with the exterior electrified wires, which carry enough electricity to kill mosquitoes but not enough to harm a human. On each day of experimentation, you will use this trap for 3 hours in the morning (06:30 to 09:30 hours) and 3 hours in the evening (15:30 to 18:30 hours). During those two daily shifts, you will be required to sit on a chair with your legs placed inside the square frame, while the rest of your body,

except for your face, will be covered with protective clothes to prevent mosquito bites. These protective clothes will be provided free of charge by the project.

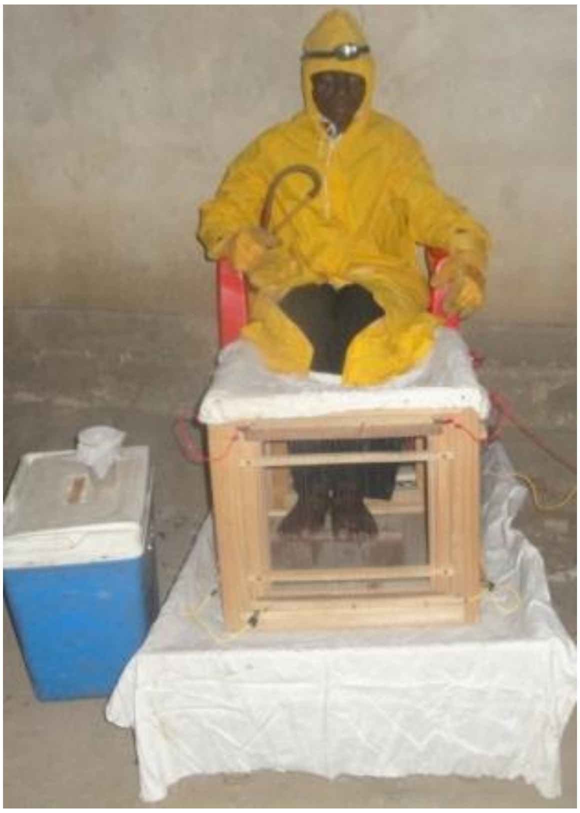

You will sit with your feet within the wooden frame of this electrical mosquito-trapping device for 45 minutes at a time during each one hour period. At the end of every 45-minute period of sitting with your feet within this device, you will take a 15 minute break to rest, relax and enjoy refreshments plus snacks, which will be provide to you free of charge. You will then move to another location nearby to sit in the same way in another, identical electrical mosquito trap. You will repeat this cycle of 45 minutes of sitting and then 15 minutes resting twice during the 3-hour working shifts in the morning (06:30 to 09:30 hours) and evening (15:30 to 18:30 hours) of each day of participation. Participation will be required for three days in a row (Tuesday to Thursday) in a single working week, which will occur once every two months for a full year or until you decide to withdraw from the study. While you are unlikely to be bitten by mosquitoes while sitting with your legs inside the trapping device, you are just as likely to be bitten during your 15 minute breaks as you would be outdoors at your home. We therefore encourage you to protect your legs during these breaks by rolling down your trousers and wearing shoes and socks, but request that you do not apply repellents which will interfere with the study.

These activities will all be conducted in the open field/or garden near your house, which we have visited with you to discuss the study.

On some days, you will be provided with an open framework containing a strip of fabric that may be treated with Transfluthrin, while on other days the strip will contain no insecticide, and on others you may be provided with no such device. If you receive one, the frame containing the treated fabric strip will be placed leaning up against your chair, immediately behind you. If you participate in this study you will not be required to handle transfluthrin, or even transfluthrin-treated fabrics: All treatment procedures and experimental manipulations of these materials will be conducted by the investigators. The only transfluthin you will be exposed to will therefore be the vapour emanating from the nearby treated strips, which are expected to deter mosquitoes by evaporating slowly into the air at concentrations more than 1000 times lower than the approved safe limit for use of this commonly-used, safe insecticide. The concentrations of transfluthrin released by this device are therefore so low that no health risks are anticipated.

Your participation is entirely voluntary and you are free to leave the study at any time if you choose to. The only risks of taking part in this study are that you may be exposed to mild electric shock, but only if you accidentally touch the outer surface of the electrocuting grid before switching off the power supply. This may be uncomfortable but is entirely harmless at the voltage used. If you happen not to like this, you are free to leave the study at any time.

You will receive an allowance of Tsh20,000/= for each day of participation, to compensate you for meals, inconvenience and discomfort. The only benefit you will receive is that, we expect that results from this study will enable mosquitoes, Dengue, Chikungunya and Zika viruses to be controlled more effectively, thereby allowing you personally, your family and the country at large to enjoy increased productivity, while also saving money which would otherwise be spent on medical care. This is because the results from this study should have very direct and significant implications for policy makers in Tanzania, as well as decision makers at their supporting donor support agencies.

The responsible scientist for this study is Dr Nicodem James Govella. This study has been approved by the Ethical Institutional Review Board of the Ifakara Health Institute. If you have any questions about this study, please contact Dr Govella at +255-686-997298. In case of further information you may need to contact Mr Fakih Bakar +255713545802 the acting secretary of Ifakara institutional ethical research committee.

MSPP | CDC Centers for Disease Control and Prevention | IRD Institut de Recherche pour le Développement France | Tulane University School of Public Health and Tropical Medicine | LSTM Liverpool School of Tropical Medicine

Affordable, scalable, low-technology transfluthrin emanators for protecting against transmission of Zika, Dengue and Chikungunya viruses.

ANNEX 1B (IN ENGLISH FOR TRANSLATION INTO FRENCH AND CREOLE IN HAITI)

PARTICIPANT INFORMATION SHEET FOR

MOSQUITO CAPTURE EXPERIMENTS

The black mosquitoes with white markings that commonly bite during daylight hours already transmit Dengue and Chikungunya viruses, which are common causes of fever, body pains and a number of other symptoms of illness here in Haiti. Furthermore, this mosquito is also known to transmit the Zika virus, which is thought to cause severe birth defects among children of pregnant women who become infected. Because they bite outdoors during the day when people are usually active, neither bed nets nor mosquito-proofed housing can provide satisfactory protection against these diseases. Unfortunately, mosquito coils and rub-on topical repellents for outdoor use only protect against mosquitoes for hours at a time, so they are too expensive and impractical for continuous use.

The team of investigators responsible for this study recently developed a low-technology transfluthrin emanator, which provides more than 90% protection for over 4 months against the night-biting mosquitoes in Africa.

We therefore invite you to participate in a study to evaluate this new tool for preventing bites by these day-biting mosquitoes here in Haiti. The goal of the study is to demonstrate that this new reformulation of transfluthrin can provide affordable protection against these important mosquitoes for at least 6 months.

While transfluthrin-treated mosquito coils always burn, so that release of the insecticide is always associated with smoke, this simpler new device allows it to evaporate at normal air temperatures, so that it protects an outdoor space with repellent vapour that will not be visible to you. The device itself consists only of a strip of Hessian fabric, the material used to make sacks for storing and transporting cereals like maize and rice, into which a widely-used insecticide called *transfluthrin* is impregnated. The transfluthrin insecticide used to treat these fabric strips costs less than $0.20 per strip, and the fabric strips can be safely hand-treated using only plastic gloves for protection. Transfluthrin has a very positive, long-standing safety record after decades of use all over the world. Transfluthrin is commonly used in repellent emanator devices like mosquito coils, has been registered for use as a repellent all across Europe, America, Asia and Africa for several decades, and is approved for experimental evaluation in Tanzania.

Several of the experiments in this study will measure how much protection various forms of these devices provide against mosquito bites. As a participant in these experiments, you will catch mosquitoes with an electric grid trap, comprised of a square plastic or wooden frame holding electrified wires. The trap frame is lined with plastic mesh, to protect your limbs against contact with the exterior electrified wires, which carry enough electricity to kill mosquitoes but not enough to harm a human. On each day of experimentation, you will use this trap for a specified period of 3 hours in the early morning and another specified period of 3 hours in the evening. During those two daily shifts,

you will be required to sit on a chair with your legs placed inside the square frame, while the rest of your body, except for your face, will be covered with protective clothes to prevent mosquito bites. These protective clothes will be provided free of charge by the project.

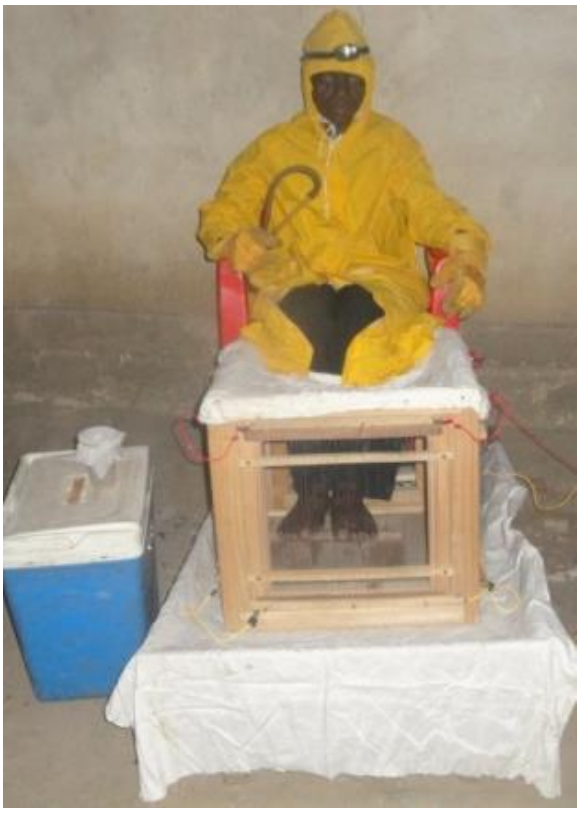

You will sit with your feet within the wooden frame of this electrical mosquito-trapping device for 45 minutes at a time during each one hour period. At the end of every 45 minute period of sitting with your feet within this device, you will take a 15 minute break to rest, relax and enjoy refreshments plus snacks, which will be provide to you free of charge. You will then move to another location nearby to sit in the same way in another, identical electrical mosquito trap. You will repeat this cycle of 45 minutes of sitting and then 15 minutes resting twice during the 3-hour working shifts in the morning and evening of each day of participation. Participation will be required for three days in a row (Tuesday to Thursday) in a single working week, which will occur once every two months for a full year or until you decide to withdraw from the study. While you are unlikely to be bitten by mosquitoes while sitting with your legs inside the trapping device, you are just as likely to be bitten during your 15 minute breaks as you would be outdoors at your home. We therefore encourage you to protect your legs during these breaks by rolling down your trousers and wearing shoes and socks, but request that you do not apply repellents which will interfere with the study.

These activities will all be conducted in the open field/or garden near your house, which we have visited with you to discuss the study.

On some days, you will be provided with an open framework containing a strip of fabric that may be treated with Transfluthrin, while on other days the strip will contain no insecticide, and on others you may be provided with no such device. If you receive one, the frame containing the treated fabric strip will be placed leaning up against your chair, immediately behind you. If you participate in this study you will not be required to handle transfluthrin, or even transfluthrin-treated fabrics: All treatment procedures and experimental manipulations of these materials will be conducted by the investigators. The only transfluthin you will be exposed to will therefore be the vapour emanating from the nearby treated strips, which are expected to deter mosquitoes by evaporating slowly into the air at concentrations more than 1000 times lower than the approved safe limit for use of this commonly-used, safe insecticide. The concentrations of transfluthrin released by this device are therefore so low that no health risks are anticipated.

Your participation is entirely voluntary and you are free to leave the study at any time if you choose to. The only risks of taking part in this study are that you may be exposed to mild electric shock, but only if you accidentally touch the outer surface of the electrocuting grid before switching off the power supply. This may be uncomfortable but is entirely harmless at the voltage used. If you happen not to like this, you are free to leave the study at any time.

You will receive an allowance of $15 for each day of participation, to compensate you for meals, inconvenience and discomfort. The only benefit you will receive is that, we expect that results from this study will enable mosquitoes, Dengue, Chikungunya and Zika viruses to be controlled more effectively, thereby allowing you personally, your family and the country at large to enjoy increased productivity, while also saving money which would otherwise be spent on medical care. This is because the results from this study should have very direct and significant implications for policy makers in Haiti, as well as decision makers at their supporting donor support agencies.

The responsible scientist for this study is Mr. Joseph Frederick, the National Malaria Control Program's vector control specialist. This study has been approved by Ethical Review Board of the Ministry of Public Health and Population. If you have any questions about this study, please contact Mr. Frederick at +509 3604 4503.

In case of further information you may need to contact Dr. Jean Frantz Lemoine at +509 3744 8755, who is the Director of the National Malaria Control Program. If you are not satisfied with the explanations provided, you may report your concerns to Dr. Gerald Lerebours, President of the Comité National de Bioéthique, c/o AMH, 29, Avenue de la Ligue Féminine ci-devant 1e Avenue du Travail, Port-au-Prince; Tel +509 3701 5766

Low-technology transfluthrin emanators for protecting against transmission of Zika, Dengue and Chikungunya viruses

ANNEX 2A (IN ENGLISH FOR TRANSLATION INTO KISWAHILI IN TANZANIA)

INFORMED CONSENT AGREEMENT FOR ADULT PARTICIPANT IN MOSQUITO-TRAPPING SURVEYS

INFORMED CONSENT AGREEMENT SERIAL NUMBER (ICF1):___________________

Aedes aegypti are the black mosquitoes with white markings that commonly bite residents of Dar es Salaam during daylight hours. Here in Tanzania, they already transmit Dengue and Chikungunya viruses, which are common causes of fever, body pains and a number of other symptoms of illness. Furthermore, this mosquito in also known to transmit the Zika virus in other countries, which is thought to cause severe birth defects among children of pregnant women who become infected.

We invite you to participate in a study to evaluate a new tool for preventing bites by these mosquitoes. The goal of the study is to demonstrate that a new, locally-made reformulation of Transfluthrin, the same safe insecticide that is commonly used in mosquito coils, can provide affordable protection against these mosquitoes for at least 6 months. Note that, while in our new approach the insecticides just evaporate and spread over space in vapor phase, in mosquito coils combustion is required and always associated with smoke.

As a participant you will catch mosquitoes with an electric grid trap, comprised of a square plastic or wooden frame holding electrified wires. The trap frame is lined with plastic mesh to protect your limbs against contact with the exterior wires, which carry enough electricity to kill mosquitoes but not enough to harm a human. On each day of experimentation, you will use this trap for 3 hours in the morning (06:30 to 09:30 hours) and 3 hours in the evening (15:30 to 18:30 hours). During those two daily shifts, you will sit on a chair with your legs placed inside the square frame, while the rest of body, except the face, will be covered with protective clothes to prevent mosquito bites. These protective clothes will be provided free of charge. On some days, you will be encircled with a strip of fabric that may be treated with a widely-used and very safe insecticide called Transfluthrin. The transfluthin treatment will deter mosquitoes by evaporating slowly into the air at concentrations more than 1000 times lower than the approved safe limit for this insecticide, which is used all over the world in mosquito coils and other repellent devices.

Your participation is voluntary and you are free to leave the study at any time if you choose to. The only risks of taking part in this study are that you may be exposed to mild electric shock, if you accidentally touch the outer surface of the electrocuting grid before switching off the power supply. This may be uncomfortable but is entirely harmless at the voltage used. If you happen not to like this, you are free to leave the study. You will receive an allowance to compensate you for meals, inconvenience and discomfort of Tsh20,000/= per day. The only other benefit you will receive is that, we expect that results from this study will enable mosquitoes, Dengue, Chikungunya and Zika viruses to be controlled more effectively, thereby allowing you personally, your family and the country at large to enjoy increased productivity, while also saving money which would otherwise be spent on medical care.

The responsible scientist for this study is Dr. Nicodem J Govella. This study has been approved by the Ethical review board of the Ifakara Health Institute. If you have any questions about this study, please contact Dr Govella at +255-686-997298.

In case of further information you may need to contact Mr Fakih Bakar +255713545802 the acting secretary of Ifakara institutional ethical research committee.

Informed consent record for the research participant

I, ………………………………………………. clearly understand the aims of the project entitled "**LOW-TECHNOLOGY TRANSFLUTHRIN EMANATORS FOR PROTECTING AGAINST TRANSMISSION OF ZIKA, DENGUE AND CHIKUNGUNYA VIRUSES**" and I agree to participate in the study. I also understand that using the electric grid trap may expose me to increased risk of electric shock with a voltage so low that it is uncomfortable but harmless. I understand that I may revoke my consent and leave the study at any stage.

Research Participant Name: ____

Gender (Male/Female: _________________________ Age:____________________

Research Participant Signature: _______________________Date_________________

Witness Name: ____

Witness signature: ____________________________________Date______________

Low-technology transfluthrin emanators for protecting against transmission of Zika, Dengue and Chikungunya viruses

ANNEX 2B (IN ENGLISH FOR TRANSLATION INTO FRENCH AND CREOLE IN HAITI)

INFORMED CONSENT AGREEMENT FOR ADULT PARTICIPANT IN MOSQUITO-TRAPPING SURVEYS

INFORMED CONSENT AGREEMENT SERIAL NUMBER:____________________

Aedes aegypti are the black mosquitoes with white markings that commonly bite during daylight hours. Here in Haiti, they transmit Dengue and Chikungunya viruses, which are common causes of fever, body pains and a number of other symptoms of illness. Furthermore, this mosquito also transmits the Zika virus, which is thought to cause severe birth defects among children of pregnant women who become infected.

We invite you to participate in a study to evaluate a new tool for preventing bites by these mosquitoes. The goal of the study is to demonstrate that a new, locally-made reformulation of Transfluthrin, the same safe insecticide that is commonly used in mosquito coils, can provide affordable protection against these mosquitoes for at least 6 months. Note that, while in our new approach the insecticides just evaporate and spread over space in vapor phase, in mosquito coils combustion is required and always associated with smoke.

As a participant you will catch mosquitoes with an electric grid trap, comprised of a square plastic or wooden frame holding electrified wires. The trap frame is lined with plastic mesh to protect your limbs against contact with the exterior wires, which carry enough electricity to kill mosquitoes but not enough to harm a human. On each day of experimentation, you will use this trap for a specified period of 3 hours in the morning and another specified period of 3 hours in the evening. During those two daily shifts, you will sit on a chair with your legs placed inside the square frame, while the rest of body, except the face, will be covered with protective clothes to prevent mosquito bites. These protective clothes will be provided free of charge. On some days, you will be encircled with a strip of fabric that may be treated with a widely-used and very safe insecticide called Transfluthrin. The transfluthin treatment will deter mosquitoes by evaporating slowly into the air at concentrations more than 1000 times lower than the approved safe limit for this insecticide, which is used all over the world in mosquito coils and other repellent devices.

Your participation is voluntary and you are free to leave the study at any time if you choose to. The only risks of taking part in this study are that you may be exposed to mild electric shock, if you accidentally touch the outer surface of the electrocuting grid before switching off the power supply. This may be uncomfortable but is entirely harmless at the voltage used. If you happen not to like this, you are free to leave the study. You will receive an allowance of $15 per day to compensate you for meals, inconvenience and discomfort. The only other benefit you will receive is that, we expect that results from this study will enable mosquitoes, Dengue, Chikungunya and Zika viruses to be controlled more effectively, thereby allowing you personally, your family and the country at large to enjoy increased productivity, while also saving money which would otherwise be spent on medical care.

The responsible scientist for this study is Mr. Joseph Frederick, the National Malaria Control Program's vector control specialist. This study has been approved by Ethical Review Board of the Ministry of Public Health and Population. If you have any questions about this study, please contact Mr. Frederick at +509 3604 4503.

In case of further information you may need to contact Dr. Jean Frantz Lemoine at +509 3744 8755, who is the Director of the National Malaria Control Program. If you are not satisfied with the explanations provided, you may report your concerns to Dr. Gerald Lerebours, President of the Comité National de Bioéthique, c/o AMH, 29, Avenue de la Ligue Féminine ci-devant 1e Avenue du Travail, Port-au-Prince; Tel +509 3701 5766

Informed consent record for the research participant

I, ……………………………………………… clearly understand the aims of the project entitled "**LOW-TECHNOLOGY TRANSFLUTHRIN EMANATORS FOR PROTECTING AGAINST TRANSMISSION OF ZIKA, DENGUE AND CHIKUNGUNYA VIRUSES**" and I agree to participate in the study. I also understand that using the electric grid trap may expose me to increased risk of electric shock with a voltage so low that it is uncomfortable but harmless. I understand that I may revoke my consent and leave the study at any stage.

Research Participant Name: ____

Gender (Male/Female: __________________________ Age:_____________________

Research Participant Signature: ________________________Date_________________

Witness Name: ____

Witness signature: _____________________________________Date_______________

Low-technology transfluthrin emanators for protecting against transmission of Zika, Dengue and Chikungunya viruses

ANNEX 3A (IN ENGLISH FOR TRANSLATION INTO KISWAHILI IN TANZANIA)

INFORMED CONSENT AGREEMENT FOR OWNER OR PERSON RESPONSIBLE FOR MANAGING AN OPEN PLOT OF LAND TO ALLOW EVALUATIONS OF MOSQUITO REPELLENTS

INFORMED CONSENT AGREEMENT SERIAL NUMBER (IC1):________________

Aedes aegypti are the black mosquitoes with white markings that commonly bite residents of Dar es Salaam during daylight hours. Here in Tanzania, they already transmit Dengue and Chikungunya viruses, which are common causes of fever, body pains and a number of other symptoms of illness. Furthermore, this mosquito in also known to transmit the Zika virus in other countries, which is thought to cause severe birth defects among children of pregnant women who become infected.

We would like your permission for you and your household to participate in a study to evaluate a new tool for preventing bites by these mosquitoes. The goal of the study is to demonstrate that a new, locally-made reformulation of Transfluthrin, the same safe insecticide that is commonly used in mosquito coils, can provide affordable protection against these mosquitoes for at least 6 months.

Specifically, we wish to trap mosquitoes and request permission for four members of our research team to trap mosquitoes attempting to bite them within your compound/plot today, for three hours in the morning (06:30 to 09:30 hours) and three hours in the evening (15:30 to 18:30 hours). He/she will sit on a chair with his/her legs placed in a mosquito trap. The traps consists of an electrified square frame, while the rest of body, except the face, will be covered with protective clothing to prevent mosquito bites. They should not disturb anyone, nor request anything from you, your household or your neighbours. On some occasions, the participants will be provided with a strip of fabric that may be treated with a widely-used and very safe insecticide called Transfluthrin. The transfluthin treatment will deter mosquitoes by evaporating slowly into the air at concentrations more than 1000 times lower that the approved safe limit for use of this insecticide, which is used all over the world in mosquito coils and other repellent devices.

Your participation is completely voluntary and you are free to leave the study at any time if you choose to. There are no risks to you, your family or your neighbours from taking part in this study. The only benefit you will receive is that, we expect that results from this study will enable mosquitoes, Dengue, Chikungunya and Zika viruses to be controlled more effectively, thereby allowing you personally, your family and the country at large to enjoy increased productivity, while also saving money which would otherwise be spent on medical care.

The responsible scientist for this study is Dr. Nicodem J Govella. This study has been approved by the Ethical review board of the Ifakara Health Institute. If you have any questions about this study, please contact Dr Govella at +255-686-997298.

For further follow-up you may need to contact Mr. Fakih Bakar +255713545802 the acting Secretary of Ifakara Institutional Ethical Research Committee.

I, ………………………………………………. clearly understand the aims of the project entitled "**LOW-TECHNOLOGY TRANSFLUTHRIN EMANATORS FOR PROTECTING AGAINST TRANSMISSION OF ZIKA, DENGUE AND CHIKUNGUNYA VIRUSES**" and I agree to allow my compound/plot to be used for mosquito-catching in this study. I understand that four member of the research team will catch mosquitoes outdoors within this compound/plot today. I expect that they should not disturb anyone staying in this plot or compound nor should they request anything from me, my household or my neighbours. I understand that participation is voluntary and I may revoke my consent to this study at any stage.

Research Participant Name: ____

Research Participant Signature: _____________________Date___________________

Witness Name: ____

Witness signature: ___________________________________Date__________

Low-technology transfluthrin emanators for protecting against transmission of Zika, Dengue and Chikungunya viruses

ANNEX 3B (IN ENGLISH FOR TRANSLATION INTO FRENCH AND CREOLE IN HAITI)

INFORMED CONSENT AGREEMENT FOR OWNER OR PERSON RESPONSIBLE FOR MANAGING AN OPEN PLOT OF LAND TO ALLOW EVALUATIONS OF MOSQUITO REPELLENTS

INFORMED CONSENT AGREEMENT SERIAL NUMBER (IC1):___________________

Aedes aegypti are the black mosquitoes with white markings that commonly bite during daylight hours. Here in Haiti, they transmit Dengue and Chikungunya viruses, which are common causes of fever, body pains and a number of other symptoms of illness. Furthermore, this mosquito also transmits the Zika virus, which is thought to cause severe birth defects among children of pregnant women who become infected.

We would like your permission for you and your household to participate in a study to evaluate a new tool for preventing bites by these mosquitoes. The goal of the study is to demonstrate that a new, locally-made reformulation of Transfluthrin, the same safe insecticide that is commonly used in mosquito coils, can provide affordable protection against these mosquitoes for at least 6 months.

Specifically, we wish to trap mosquitoes and request permission for four members of our research team to trap mosquitoes attempting to bite them within your compound/plot today, for a specified period of 3 hours in the morning and another specified period of 3 hours in the evening. He/she will sit on a chair with his/her legs placed in a mosquito trap. The traps consists of an electrified square frame, while the rest of body, except the face, will be covered with protective clothing to prevent mosquito bites. They should not disturb anyone, nor request anything from you, your household or your neighbours. On some occasions, the participants will be provided with a strip of fabric that may be treated with a widely-used and very safe insecticide called Transfluthrin. The transfluthin treatment will deter mosquitoes by evaporating slowly into the air at concentrations more than 1000 times lower that the approved safe limit for use of this insecticide, which is used all over the world in mosquito coils and other repellent devices.

Your participation is completely voluntary and you are free to leave the study at any time if you choose to. There are no risks to you, your family or your neighbours from taking part in this study. The only benefit you will receive is that, we expect that results from this study will enable mosquitoes, Dengue, Chikungunya and Zika viruses to be controlled more effectively, thereby allowing you personally, your family and the country at large to enjoy increased productivity, while also saving money which would otherwise be spent on medical care.

The responsible scientist for this study is Mr. Joseph Frederick, the National Malaria Control Program's vector control specialist. This study has been approved by Ethical Review Board of the Ministry of Public Health and Population. If you have any questions about this study, please contact Mr. Frederick at +509 3604 4503.

In case of further information you may need to contact Dr. Jean Frantz Lemoine at +509 3744 8755, who is the director of the National Malaria Control Program. If you are not satisfied with the explanations provided, you may report your concerns to Dr. Gerald Lerebours, President of the Comité National de Bioéthique, c/o AMH, 29, Avenue de la Ligue Féminine ci-devant 1e Avenue du Travail, Port-au-Prince; Tel +509 3701 5766)

I, ……………………………………………. clearly understand the aims of the project entitled "**LOW-TECHNOLOGY TRANSFLUTHRIN EMANATORS FOR PROTECTING AGAINST TRANSMISSION OF ZIKA, DENGUE AND CHIKUNGUNYA VIRUSES**" and I agree to allow my compound/plot to be used for mosquito-catching in this study. I understand that four member of the research team will catch mosquitoes outdoors within this compound/plot today. I expect that they should not disturb anyone staying in this plot or compound nor should they request anything from me, my household or my neighbours. I understand that participation is voluntary and I may revoke my consent to this study at any stage.

Research Participant Name: ____

Research Participant Signature: _____________________Date___________________

Witness Name: ____

Witness signature: ___________________________________Date___________________

Affordable, scalable, low-technology transfluthrin emanators for protecting against transmission of Zika, Dengue and Chikungunya viruses.

ANNEX 4 (IN ENGLISH FOR TRANSLATION INTO FRENCH AND CREOLE IN HAITI)

PARTICIPANT INFORMATION SHEET FOR ROUTINE HOUSEHOLD USE OF TRANSFLUTHRIN EMANATORS

The black mosquitoes with white markings that commonly bite during daylight hours already transmit Dengue and Chikungunya viruses, which are common causes of fever, body pains and a number of other symptoms of illness here in Haiti. Furthermore, this mosquito is also known to transmit the Zika virus, which is thought to cause severe birth defects among children of pregnant women who become infected. Because they bite outdoors during the day when people are usually active, neither bed nets nor mosquito-proofed housing can provide satisfactory protection against these diseases. Unfortunately, mosquito coils and rub-on topical repellents for outdoor use only protect against mosquitoes for hours at a time, so they are too expensive and impractical for continuous use.

The team of investigators responsible for this study recently developed a low-technology transfluthrin emanator, which provides more than 90% protection for over 4 months against the night-biting mosquitoes in Africa.

We therefore invite you to participate in a study to evaluate this new tool for preventing bites by these day-biting mosquitoes here in Haiti. The goal of the study is to demonstrate that this new reformulation of transfluthrin can provide affordable protection against these important mosquitoes for at least 6 months.

While transfluthrin-treated mosquito coils always burn, so that release of the insecticide is always associated with smoke, this simpler new device allows it to evaporate at normal air temperatures, so that it protects an outdoor space with repellent vapour that will not be visible to you. The device itself consists only of a strip of Hessian fabric, the material used to make sacks for storing and transporting cereals like maize and rice, into which a widely-used insecticide called *transfluthrin* is impregnated. The transfluthrin insecticide used to treat these fabric strips costs less than $0.20 per strip, and the fabric strips can be safely hand-treated using only plastic gloves for protection. Transfluthrin has a very positive, long-standing safety record after decades of use all over the world. Transfluthrin is commonly used in repellent emanator devices like mosquito coils, has been registered for use as a repellent all across Europe, America, Asia and Africa for several decades, and is approved for experimental evaluation in Tanzania.

One of the experiments in this study will involve providing two of these emanator devices to a small number of consenting households, for them to use as protection against mosquitoes over the course of 6 months. If you and your household agree to participate in this part of the study, the research team will visit your house once every two months and take back the emanator devices for approximately two weeks to measure how much protection they provide against mosquito bites at that time. Following advisory discussions with the research team on how safely and effectively deploy

them, you will be free to use the emanators in whatever way you perceive to be the most convenient and effective, so long as you do not open the protective holder or use it in any other way that would allow direct physical contact with the treated fabric inside. Also, one member of your household will be asked to record what your household perceives to be the most and least effective ways to use these devices, using disposable cameras that will be provided by the study team. Before using these cameras, the person taking responsibility for taking the photographs will take part in a short training meeting to outline acceptable ways of using the camera without compromising the safety, privacy, or other rights of your fellow householders or your neighbours. Also, when each household is visited once every two months, to pick up and return the emanators to be tested for protection level, you will be asked to answer a very brief semi-structured questionnaire to assess your level of satisfaction with the protection provided against mosquito bites. At the start of each visit to the household, any fully-used disposable cameras will be collected by the research team so that the two copies of each photo can be developed. One copy will be returned to you within a few days, while the other will be retained confidentially by the research team as a replacement in case of loss. At the end of the study, one consenting adult female member of your household will be asked to participate in a group discussion about the device with a group of up to 8 other female participants, from other households to whom these devices were provided. Similarly, one consenting male participant from each household will engage in a group discussion about the device with an all-male group of the same size. At each group discussion, a facilitator working for the study team will coordinate discussion while an observer will make an audio recording and take notes about what was said. Similarly, the household member responsible for taking photographs will be asked to select their best or most informative photographs and then discuss their significance with small groups of photographers from other households.

The participation of your household, and each of its members is entirely voluntary and you are free to leave the study at any time if you choose to. If the research team wishes to publish any photographs taken by the householders of their house, its contents or its occupants, the head of the household will first be consulted and asked to provide written consent, which they may freely refuse.

You will receive no compensation for your participation in this study. The only direct benefit you will receive is direct protection against mosquito bites. We also expect that you may benefit indirectly, because results from this study will enable mosquitoes, Dengue, Chikungunya and Zika viruses to be controlled more effectively. Such an indirect benefit over the long term would allow you, your family and the country at large to enjoy increased productivity, while also saving money which would otherwise be spent on medical care. This is because the results from this study should have very direct implications for policy makers in Haiti, as well as decision makers at their supporting donor support agencies.

The responsible scientist for this study is Mr. Joseph Frederick, the National Malaria Control Program's vector control specialist. This study has been approved by Ethical Review Board of the Ministry of Public Health and Population. If you have any questions about this study, please contact Mr. Frederick at +509 3604 4503.

In case of further information you may need to contact Dr. Jean Frantz Lemoine at +509 3744 8755, who is the Director of the National Malaria Control Program. If you are not satisfied with the explanations provided, you may report your concerns to Dr. Gerald Lerebours, President of the Comité National de Bioéthique, c/o AMH, 29, Avenue de la Ligue Féminine ci-devant 1e Avenue du Travail, Port-au-Prince; Tel +509 3701 5766

Low-technology transfluthrin emanators for protecting against transmission of Zika, Dengue and Chikungunya viruses

ANNEX 5 (IN ENGLISH FOR TRANSLATION INTO FRENCH AND CREOLE IN HAITI)

INFORMED CONSENT AGREEMENT FOR HEADS OF HOUSEHOLDS ROUTINELY USING TRANSFLUTHRIN EMANATORS

INFORMED CONSENT AGREEMENT SERIAL NUMBER:____________________

Aedes aegypti are the black mosquitoes with white markings that commonly bite during daylight hours. Here in Haiti, they transmit Dengue and Chikungunya viruses, which are common causes of fever, body pains and a number of other symptoms of illness. Furthermore, this mosquito also transmits the Zika virus, which is thought to cause severe birth defects among children of pregnant women who become infected.

We invite you to participate in a study to evaluate a new tool for preventing bites by these mosquitoes. The goal of the study is to demonstrate that a new, locally-made reformulation of Transfluthrin, the same safe insecticide that is commonly used in mosquito coils, can provide affordable protection against these mosquitoes for at least 6 months. Note that, while in our new approach the insecticides just evaporate and spread over space in vapor phase, in mosquito coils combustion is required and always associated with smoke.

One of the experiments in this study will involve providing two of these emanator devices to a small number of consenting households, for them to use as protection against mosquitoes over the course of 6 months. If you and your household agree to participate in this part of the study, the research team will visit your house once every two months and take back the emanator devices for approximately two weeks to measure how much protection they provide against mosquito bites at that time. Following advisory discussions with the research team on how safely and effectively deploy them, you will be free to use the emanators in whatever way you perceive to be the most convenient and effective, so long as you do not open the protective holder or use it in any other way that would allow direct physical contact with the treated fabric inside.

Also, one member of your household will be asked to record what your household perceives to be the most and least effective ways to use these devices, using disposable cameras that will be provided by the study team. Before using these cameras, the person taking responsibility for taking the photographs will take part in a short training meeting to outline acceptable ways of using the camera without compromising the safety, privacy, or other rights of your fellow householders or your neighbours. Also, when each household is visited once every two months, to pick up and return the emanators to be tested for protection level, you will be asked to answer a very brief semi-structured questionnaire to assess your level of satisfaction with the protection provided against mosquito bites. At the start of each visit to the household, any fully-used disposable cameras will be collected by the research team so that the two copies of each photo can be developed. One copy will be returned to you within a few days, while the other will be retained confidentially by the research team as a replacement in case of loss. At the end of the study, one consenting adult female member of your household will be asked to participate in a group discussion about the device with a group of up to 8 other female participants, from other households to whom these devices were provided. Similarly,

one consenting male participant from each household will engage in a group discussion about the device with an all-male group of the same size. At each group discussion, a facilitator working for the study team will coordinate discussion while an observer will make an audio recording and take notes about what was said. Similarly, the household member responsible for taking photographs will be asked to select their best or most informative photographs and then discuss their significance with small groups of photographers from other households. No personal information will be recorded, other than your name, gender and age, and these details will be kept confidential. The participation of you and your household is entirely voluntary and you are free to leave the study at any time if you choose to.

The participation of your household, and each of its members is entirely voluntary and you are free to leave the study at any time if you choose to. If the research team wishes to publish any photographs taken by the householders of their house, its contents or its occupants, the head of the household will first be consulted and asked to provide written consent, which he or she may freely refuse.

You will receive no compensation for your participation in this study. The only direct benefit you will receive is direct protection against mosquito bites. We also expect that you may benefit indirectly, because results from this study will enable mosquitoes, Dengue, Chikungunya and Zika viruses to be controlled more effectively. Such an indirect benefit over the long term would allow you, your family and the country at large to enjoy increased productivity, while also saving money which would otherwise be spent on medical care. This is because the results from this study should have very direct implications for policy makers in Haiti, as well as decision makers at their supporting donor support agencies.

The responsible scientist for this study is Mr. Joseph Frederick, the National Malaria Control Program's vector control specialist. This study has been approved by Ethical Review Board of the Ministry of Public Health and Population. If you have any questions about this study, please contact Mr. Frederick at +509 3604 4503.

In case of further information you may need to contact Dr. Jean Frantz Lemoine at +509 3744 8755, who is the Director of the National Malaria Control Program. If you are not satisfied with the explanations provided, you may report your concerns to Dr. Gerald Lerebours, President of the Comité National de Bioéthique, c/o AMH, 29, Avenue de la Ligue Féminine ci-devant 1e Avenue du Travail, Port-au-Prince; Tel +509 3701 5766

Informed consent record for the research participant

As head of my household, I ………………………………………………. clearly understand the aims of the project entitled "**LOW-TECHNOLOGY TRANSFLUTHRIN EMANATORS FOR PROTECTING AGAINST TRANSMISSION OF ZIKA, DENGUE AND CHIKUNGUNYA VIRUSES**". I agree for my household to participate in the study by using the repellent emanators provided by the study team. I also agree for my household members to participate in recorded discussions about these devices and for one of my household members to take photographs illustrating how these devices are used. I also understand that the study team must consult me to obtain my written consent before publishing any of these photographs, or sharing them with anyone other than our household and the study team. I understand that I may revoke my consent and leave the study at any stage.

Research Participant Name: ____

Gender (Male/Female): __________________________ Age:_____________________

Research Participant Signature: ________________________Date__________________

Witness Name: ____

Witness signature: _______________________________________Date________________

Low-technology transfluthrin emanators for protecting against transmission of Zika, Dengue and Chikungunya viruses

ANNEX 6 (IN ENGLISH FOR TRANSLATION INTO FRENCH AND CREOLE IN HAITI)

INFORMED CONSENT AGREEMENT FOR QUESTIONAIRE RESPONDENTS FROM HOUSEHOLDS USING TRANSFLUTHRIN EMANATORS

INFORMED CONSENT AGREEMENT SERIAL NUMBER:____________________

Aedes aegypti are the black mosquitoes with white markings that commonly bite during daylight hours. Here in Haiti, they transmit Dengue and Chikungunya viruses, which are common causes of fever, body pains and a number of other symptoms of illness. Furthermore, this mosquito also transmits the Zika virus, which is thought to cause severe birth defects among children of pregnant women who become infected.

We invite you to participate in a study to evaluate a new tool for preventing bites by these mosquitoes. The goal of the study is to demonstrate that a new, locally-made reformulation of Transfluthrin, the same safe insecticide that is commonly used in mosquito coils, can provide affordable protection against these mosquitoes for at least 6 months. Note that, while in our new approach the insecticides just evaporate and spread over space in vapor phase, in mosquito coils combustion is required and always associated with smoke.

One of the experiments in this study will involve providing two of these emanator devices to a small number of consenting households, for them to use as protection against mosquitoes over the course of 6 months. If your household agrees to participate in this part of the study, the research team will visit your house once every two months and take back the emanator devices for approximately two weeks to measure how much protection they provide against mosquito bites at that time. Following advisory discussions with the research team on how safely and effectively deploy them, you will be free to use the emanators in whatever way you perceive to be the most convenient and effective, so long as you do not open the protective holder or use it in any other way that would allow direct physical contact with the treated fabric inside. Also, as a member of your household, you will be asked to answer a very brief semi-structured questionnaire to assess your level of satisfaction with the protection provided against mosquito bites. No personal information will be recorded, other than your name, gender and age, and these details will be kept confidential. The participation of you and your household is entirely voluntary and you are free to leave the study at any time you choose to.

You will receive no compensation for your participation in this study. The only direct benefit you will receive is direct protection against mosquito bites. We also expect that you may benefit indirectly, because results from this study will enable mosquitoes, Dengue, Chikungunya and Zika viruses to be controlled more effectively. Such an indirect benefit over the long term would allow you, your family and the country at large to enjoy increased productivity, while also saving money which would otherwise be spent on medical care. This is because the results from this study should have very direct implications for policy makers in Haiti, as well as decision makers at their supporting donor support agencies.

The responsible scientist for this study is Mr. Joseph Frederick, the National Malaria Control Program's vector control specialist. This study has been approved by Ethical Review Board of the Ministry of Public Health and Population. If you have any questions about this study, please contact Mr. Frederick at +509 3604 4503.

In case of further information you may need to contact Dr. Jean Frantz Lemoine at +509 3744 8755, who is the Director of the National Malaria Control Program. If you are not satisfied with the explanations provided, you may report your concerns to Dr. Gerald Lerebours, President of the Comité National de Bioéthique, c/o AMH, 29, Avenue de la Ligue Féminine ci-devant 1e Avenue du Travail, Port-au-Prince; Tel +509 3701 5766

Informed consent record for the research participant

As a member of my household, I ……………………………………………. clearly understand the aims of the project entitled "**LOW-TECHNOLOGY TRANSFLUTHRIN EMANATORS FOR PROTECTING AGAINST TRANSMISSION OF ZIKA, DENGUE AND CHIKUNGUNYA VIRUSES**". I agree for my household to participate in the study by using the repellent emanators provided by the study team. I also agree to participate in a brief questionnaire survey about my satisfaction with these devices. I understand that I may revoke my consent and leave the study at any stage.

Research Participant Name: ____

Gender (Male/Female: ____________________________ Age:______________________

Research Participant Signature: _________________________Date___________________

Witness Name: ____

Witness signature: _______________________________________Date________________

MSPP
CDC CENTERS FOR DISEASE CONTROL AND PREVENTION
IRD Institut de Recherche pour le Développement FRANCE
Tulane University School of Public Health and Tropical Medicine
LSTM LIVERPOOL SCHOOL OF TROPICAL MEDICINE

Low-technology transfluthrin emanators for protecting against transmission of Zika, Dengue and Chikungunya viruses

ANNEX 7 (IN ENGLISH FOR TRANSLATION INTO FRENCH AND CREOLE IN HAITI)

QUESTIONNAIRE FOR SURVEYING HOUSEHOLDER SATISFACTION WITH TRANSFLUTHRIN EMANATORS

House number:___ Participant Name:__ _ Gender (M/F):__ _ Date:_________

Informed consent agreement serial number (ICF1) for participant: ____________________

1. How well do the repellent emanators protect you against mosquitoes when you are indoors? Circle one of the answers below, or circle the following statement: **NO ANSWER PROVIDED**.

1<br>NOT AT ALL	2<br>ONLY SLIGHTLY	3<br>WELL	4<br>VERY WELL	5<br>COMPLETELY

2. How well do the repellent emanators protect you and your household against other insect pests when you are indoors? Circle one of the answers below, or the following statement: **No answer provided**.

1<br>NOT AT ALL	2<br>ONLY SLIGHTLY	3<br>WELL	4<br>VERY WELL	5<br>COMPLETELY

3. How well do the repellent emanators protect you against mosquitoes when you are outdoors? Circle one of the answers below, or circle the following statement: **NO ANSWER PROVIDED**.

1<br>NOT AT ALL	2<br>ONLY SLIGHTLY	3<br>WELL	4<br>VERY WELL	5<br>COMPLETELY

4. How well do the repellent emanators protect you and your household against other insect pests when you are outdoors? Circle one of the answers below, or the following statement: **No answer provided**.

1<br>NOT AT ALL	2<br>ONLY SLIGHTLY	3<br>WELL	4<br>VERY WELL	5<br>COMPLETELY

5. Name and/or describe any other advantage or disadvantage of having these repellent emanators:____
____
____

6. Classify the extent of this advantage/disadvantage by circling one of the answers below, or one of the following statements: **NOT APPLICABLE** or **NO ANSWER PROVIDED**.

1<br>BIG DISADVANTAGE	2<br>MODERATE DISADVANTAGE	3<br>SMALL DISADVANTAGE	4<br>SMALL ADVANTAGE	5<br>MODERATE ADVANTAGE	6<br>BIG ADVANTAGE

7. Name and/or describe any other advantage or disadvantage of having these repellent emanators:____
____

8. Classify the extent of this advantage/disadvantage by circling one of the answers below, or one of the following statements: **NOT APPLICABLE** or **NO ANSWER PROVIDED**.

1	2	3	4	5	6
BIG DISADVANTAGE	MODERATE DISADVANTAGE	SMALL DISADVANTAGE	SMALL ADVANTAGE	MODERATE ADVANTAGE	BIG ADVANTAGE

9. Name and/or describe any other advantage or disadvantage of having these repellent emanators:____
____
____ __________________

10. Classify the extent of this advantage/disadvantage by circling one of the answers below, or one of the following statements: **NOT APPLICABLE** or **NO ANSWER PROVIDED**.

1	2	3	4	5	6
BIG DISADVANTAGE	MODERATE DISADVANTAGE	SMALL DISADVANTAGE	SMALL ADVANTAGE	MODERATE ADVANTAGE	BIG ADVANTAGE

Low-technology transfluthrin emanators for protecting against transmission of Zika, Dengue and Chikungunya viruses

ANNEX 8 (IN ENGLISH FOR TRANSLATION INTO FRENCH AND CREOLE IN HAITI)

INFORMED CONSENT AGREEMENT FOR FOCUS GROUP DISCUSSION PARTICIPANTS FROM HOUSEHOLDS USING TRANSFLUTHRIN EMANATORS

INFORMED CONSENT AGREEMENT SERIAL NUMBER:____________________

Aedes aegypti are the black mosquitoes with white markings that commonly bite during daylight hours. Here in Haiti, they transmit Dengue and Chikungunya viruses, which are common causes of fever, body pains and a number of other symptoms of illness. Furthermore, this mosquito also transmits the Zika virus, which is thought to cause severe birth defects among children of pregnant women who become infected.

We invite you to participate in a study to evaluate a new tool for preventing bites by these mosquitoes. The goal of the study is to demonstrate that a new, locally-made reformulation of Transfluthrin, the same safe insecticide that is commonly used in mosquito coils, can provide affordable protection against these mosquitoes for at least 6 months. Note that, while in our new approach the insecticides just evaporate and spread over space in vapor phase, in mosquito coils combustion is required and always associated with smoke.

One of the experiments in this study will involve providing two of these emanator devices to a small number of consenting households, for them to use as protection against mosquitoes over the course of 6 months. If your household agrees to participate in this part of the study, the research team will visit your house once every two months and take back the emanator devices for approximately two weeks to measure how much protection they provide against mosquito bites at that time. Following advisory discussions with the research team on how safely and effectively deploy them, you will be free to use the emanators in whatever way you perceive to be the most convenient and effective, so long as you do not open the protective holder or use it in any other way that would allow direct physical contact with the treated fabric inside.

You are requested to represent your household by participating in a group discussion about the device with a group of up to 8 other participants of the same gender. These other members of the discussion group will come from the other households to whom these devices were provided. A facilitator working for the study team will coordinate discussion while an observer will make an audio recording and take notes about what was said. No personal information will be recorded, other than your name, gender and age, and these details will be kept confidential. The participation of you and your household is entirely voluntary and you are free to leave the study at any time if you choose to.

You will receive no compensation for your participation in this study. The only direct benefit you will receive is direct protection against mosquito bites. We also expect that you may benefit indirectly, because results from this study will enable mosquitoes, Dengue, Chikungunya and Zika viruses to be controlled more effectively. Such an indirect benefit over the long term would allow you, your family

and the country at large to enjoy increased productivity, while also saving money which would otherwise be spent on medical care. This is because the results from this study should have very direct implications for policy makers in Haiti, as well as decision makers at their supporting donor support agencies.

The responsible scientist for this study is Mr. Joseph Frederick, the National Malaria Control Program's vector control specialist. This study has been approved by Ethical Review Board of the Ministry of Public Health and Population. If you have any questions about this study, please contact Mr. Frederick at +509 3604 4503.

In case of further information you may need to contact Dr. Jean Frantz Lemoine at +509 3744 8755, who is the Director of the National Malaria Control Program. If you are not satisfied with the explanations provided, you may report your concerns to Dr. Gerald Lerebours, President of the Comité National de Bioéthique, c/o AMH, 29, Avenue de la Ligue Féminine ci-devant 1e Avenue du Travail, Port-au-Prince; Tel +509 3701 5766

Informed consent record for the research participant

As a representative of my household, I ………………………………………………. clearly understand the aims of the project entitled "**LOW-TECHNOLOGY TRANSFLUTHRIN EMANATORS FOR PROTECTING AGAINST TRANSMISSION OF ZIKA, DENGUE AND CHIKUNGUNYA VIRUSES**". I agree for my household to participate in the study by using the repellent emanators provided by the study team. I also agree to participate in recorded discussions about these devices. I understand that I may revoke my consent and leave the study at any stage.

Research Participant Name: ____

Gender (Male/Female: __________________________ Age:_____________________

Research Participant Signature: ________________________Date_________________

Witness Name: ____

Witness signature: _____________________________________Date_______________

Low-technology transfluthrin emanators for protecting against transmission of Zika, Dengue and Chikungunya viruses

ANNEX 9 (IN ENGLISH FOR TRANSLATION INTO FRENCH AND CREOLE IN HAITI)

FOCUS GROUP DISCUSSION TOPIC GUIDE

1. Pre-existing perceptions of mosquitoes and means to prevent them

    1a. Problems or benefits associated with mosquitoes.

    1b. Actions commonly taken to avoid mosquito bites or kill mosquitoes.

    1c. Limitations of pre-existing means of protection against mosquitoes.

2. Perceptions of the advantages and disadvantages of the reprellent emanator devices

    2a. Protection against mosquito bites.

    2b. Other advantages

    2c. Limitations of protection

    2d. Any disadvantages.

    2e. Other factors influencing their use.

3. Use practices for the repellent emanator devices

    3a. Good use practices

    3b. Bad use practices

    3c. Limitations and disadvantages

    3d. Ideas for improvement of the devices themselves

    3e. Ideas for delivery and maintenance of the devices

Low-technology transfluthrin emanators for protecting against transmission of Zika, Dengue and Chikungunya viruses

ANNEX 10 (IN ENGLISH FOR TRANSLATION INTO FRENCH AND CREOLE IN HAITI)

INFORMED CONSENT AGREEMENT FOR PHOTOVOICE PARTICIPANTS FROM HOUSEHOLDS USING TRANSFLUTHRIN EMANATORS

INFORMED CONSENT AGREEMENT SERIAL NUMBER:____________________

Aedes aegypti are the black mosquitoes with white markings that commonly bite during daylight hours. Here in Haiti, they transmit Dengue and Chikungunya viruses, which are common causes of fever, body pains and a number of other symptoms of illness. Furthermore, this mosquito also transmits the Zika virus, which is thought to cause severe birth defects among children of pregnant women who become infected.

We invite you to participate in a study to evaluate a new tool for preventing bites by these mosquitoes. The goal of the study is to demonstrate that a new, locally-made reformulation of Transfluthrin, the same safe insecticide that is commonly used in mosquito coils, can provide affordable protection against these mosquitoes for at least 6 months. Note that, while in our new approach the insecticides just evaporate and spread over space in vapor phase, in mosquito coils combustion is required and always associated with smoke.

One of the experiments in this study will involve providing two of these emanator devices to a small number of consenting households, for them to use as protection against mosquitoes over the course of 6 months. If your household agrees to participate in this part of the study, the research team will visit your house once every two months and take back the emanator devices for approximately two weeks to measure how much protection they provide against mosquito bites at that time. Following advisory discussions with the research team on how safely and effectively deploy them, you will be free to use the emanators in whatever way you perceive to be the most convenient and effective, so long as you do not open the protective holder or use it in any other way that would allow direct physical contact with the treated fabric inside.

As a representative of your household, we request that you record what your household perceives to be the most and least effective ways to use these devices, using disposable cameras that will be provided by the study team. Before using these cameras, you will take part in a short training meeting to outline acceptable ways of using the camera without compromising the safety, privacy, or other rights of your fellow householders or your neighbours. Also, when your household is visited once every two months, any camera you have that has been fully used will be collected by the research team, so that the two copies of each photo can be developed. One copy will be returned to you within a few days, while the other will be retained confidentially by the research team as a replacement in case of loss. We also request that you participate in a group meeting with photographers from other households in your neighbourhood, to select the most informative photographs and then discuss their significance. A facilitator working for the study team will coordinate discussion while an observer will make an audio recording and take notes about what was said. No personal information will be recorded, other than your name, gender and age, and these details will be kept confidential. The

participation of you and your household is entirely voluntary and you are free to leave the study at any time if you choose to.

You will receive no compensation for your participation in this study. The only direct benefit you will receive is direct protection against mosquito bites. We also expect that you may benefit indirectly, because results from this study will enable mosquitoes, Dengue, Chikungunya and Zika viruses to be controlled more effectively. Such an indirect benefit over the long term would allow you, your family and the country at large to enjoy increased productivity, while also saving money which would otherwise be spent on medical care. This is because the results from this study should have very direct implications for policy makers in Haiti, as well as decision makers at their supporting donor support agencies.

The responsible scientist for this study is Mr. Joseph Frederick, the National Malaria Control Program's vector control specialist. This study has been approved by Ethical Review Board of the Ministry of Public Health and Population. If you have any questions about this study, please contact Mr. Frederick at +509 3604 4503.

In case of further information you may need to contact Dr. Jean Frantz Lemoine at +509 3744 8755, who is the Director of the National Malaria Control Program. If you are not satisfied with the explanations provided, you may report your concerns to Dr. Gerald Lerebours, President of the Comité National de Bioéthique, c/o AMH, 29, Avenue de la Ligue Féminine ci-devant 1e Avenue du Travail, Port-au-Prince; Tel +509 3701 5766

Informed consent record for the research participant

As a representative of my household, I ………………………………………………. clearly understand the aims of the project entitled "**LOW-TECHNOLOGY TRANSFLUTHRIN EMANATORS FOR PROTECTING AGAINST TRANSMISSION OF ZIKA, DENGUE AND CHIKUNGUNYA VIRUSES**". I agree to take photographs using the camera provided to illustrate how these devices are used, and to participate in recorded discussions about these devices. I understand that I may revoke my consent and leave the study at any stage.

Research Participant Name: ____

Gender (Male/Female: __________________________ Age:_____________________

Research Participant Signature: ________________________Date__________________

Witness Name: ____

Witness signature: _____________________________________Date_______________

Low-technology transfluthrin emanators for protecting against transmission of Zika, Dengue and Chikungunya viruses

ANNEX 11 (IN ENGLISH FOR TRANSLATION INTO FRENCH AND CREOLE IN HAITI)

PHOTOVOICE FOCUS GROUP DISCUSSION TOPIC GUIDE

1. Introduce pictures/photos and ask participants to narrate their meaning and significance.

2. Probe perceptions of how the photographs illustrate the advantages and disadvantages of the repellent emanator devices.

    2a. Protection against mosquito bites.

    2b. Other advantages

    2c. Limitations of protection

    2d. Any disadvantages.

    2e. Other factors influencing their use.

3. Probe perceptions of how the photographs illustrate use practices for the repellent emanator devices and ideas for optimizing them, or their delivery and maintenance, in the future.

    3a. Good use practices

    3b. Bad use practices

    3c. Limitations and disadvantages

    3d. Ideas for improvement of the devices themselves

    3e. Ideas for delivery and maintenance of the devices

Low-technology transfluthrin emanators for protecting against transmission of Zika, Dengue and Chikungunya viruses

ANNEX 12 (IN ENGLISH FOR TRANSLATION INTO FRENCH AND CREOLE IN HAITI)

SUPPLEMENTARY INFORMED CONSENT FORM FOR PUBLICATION OR DISSEMINATION OF A SPECIFIC PHOTOGRAPH

Informed consent agreement serial number (ICF1):_____________ House number:_______

As previous documented in writing (ICF1______________), you and your household kindly agreed to participate in a research project to evaluate new repellent emanator devices for protecting you and your household against mosquito bites. Please refer to the participant information sheet for further details. Also, one member of your household agreed to take photographs to illustrate how you and your household use these devices, as well as ideas for improving their design, delivering them and maintaining them The research team now wish to publish or otherwise share the following photograph of your house, its contents and/or its occupants, and request your written permission to do so. We also request written permission from anyone who features in this photograph to publish or otherwise disseminate it, and would be grateful for your assistance in identifying, contacting and seeking written consent from these individuals, documented on this same form. We are happy to edit this photograph to mask out any personally identifiable information, such as facial features, or about your household, house or belongings, until you are satisfied to allow its use. Your participation in this study is entirely voluntary, and you are free to refuse permission for us to publish this photograph.

INSERT PHOTOGRAPH HERE

You will receive no compensation for your participation in this study. The only direct benefit you will receive is direct protection against mosquito bites. We also expect that you may benefit indirectly, because results from this study will enable mosquitoes, Dengue, Chikungunya and Zika viruses to be controlled more effectively. Such an indirect benefit over the long term would allow you, your family and the country at large to enjoy increased productivity, while also saving money which would otherwise be spent on medical care. This is because the results from this study should have very direct implications for policy makers in Haiti, as well as decision makers at their supporting donor support agencies.

The responsible scientist for this study is Mr. Joseph Frederick, the National Malaria Control Program's vector control specialist. This study has been approved by Ethical Review Board of the Ministry of Public Health and Population. If you have any questions about this study, please contact Mr. Frederick at +509 3604 4503.

In case of further information you may need to contact Dr. Jean Frantz Lemoine at +509 3744 8755, who is the Director of the National Malaria Control Program. If you are not satisfied with the explanations provided, you may report your concerns to Dr. Gerald Lerebours, President of the Comité National de Bioéthique, c/o AMH, 29, Avenue de la Ligue Féminine ci-devant 1e Avenue du Travail, Port-au-Prince; Tel +509 3701 5766

Informed consent record of the household head

As head of our household, I …………………………………………….. clearly understand the aims of the project entitled "**Insecticide-treated eave baffles for killing malaria mosquitoes and preventing them from entering houses**" and I agree that the photograph above, taken in or around my household, may be published or otherwise shared freely in the exact form presented above.

Research Participant Name: ____

Gender (Male/Female: _________________________ Age:______________________

Research Participant Signature: ________________________Date___________________

Witness Name: ____

Witness Signature: _____________________________________Date________________

Informed consent/assent record of individuals featured in the photograph

As an individual featured in the above photograph, or their parent/legal guardian, I ………………………………………………. clearly understand the aims of the project entitled "**Insecticide-treated eave baffles for killing malaria mosquitoes and preventing them from entering houses**" and I agree that it may be published or otherwise shared freely in the exact form presented above.

Research Participant Name: ____

Gender (Male/Female: ________________________ Age:_____________________

Research Participant Signature: ________________________Date___________________

Witness Name: ____

Witness Signature: _____________________________________Date________________

As an individual featured in the above photograph, I ………………………………………………. clearly understand the aims of the project entitled "**Insecticide-treated eave baffles for killing malaria mosquitoes and preventing them from entering houses**" and I agree that it may be published or otherwise shared freely in the exact form presented above.

Research Participant Name: ____

Gender (Male/Female: ________________________ Age:_____________________

Research Participant Signature: ________________________Date___________________

Witness Name: ____

Witness Signature: _____________________________________Date________________

As an individual featured in the above photograph, I ………………………………………………. clearly understand the aims of the project entitled "**Insecticide-treated eave baffles for killing malaria mosquitoes and preventing them from entering houses**" and I agree that it may be published or otherwise shared freely in the exact form presented above.

Research Participant Name: ____

Gender (Male/Female: ________________________ Age:_____________________

Research Participant Signature: ________________________Date___________________

Witness Name: ____

Witness Signature: _____________________________________Date________________

As an individual featured in the above photograph, I ……………………………………………. clearly understand the aims of the project entitled "**Insecticide-treated eave baffles for killing malaria mosquitoes and preventing them from entering houses**" and I agree that it may be published or otherwise shared freely in the exact form presented above.

Research Participant Name: ____

Gender (Male/Female: _________________________ Age:______________________

Research Participant Signature: _________________________Date____________________

Witness Name: ____

Witness Signature: _______________________________________Date_________________

As an individual featured in the above photograph, I ……………………………………………. clearly understand the aims of the project entitled "**Insecticide-treated eave baffles for killing malaria mosquitoes and preventing them from entering houses**" and I agree that it may be published or otherwise shared freely in the exact form presented above.

Research Participant Name: ____

Gender (Male/Female: _________________________ Age:______________________

Research Participant Signature: _________________________Date____________________

Witness Name: ____

Witness Signature: _______________________________________Date_________________

As an individual featured in the above photograph, I ……………………………………………. clearly understand the aims of the project entitled "**Insecticide-treated eave baffles for killing malaria mosquitoes and preventing them from entering houses**" and I agree that it may be published or otherwise shared freely in the exact form presented above.

Research Participant Name: ____

Gender (Male/Female: _________________________ Age:______________________

Research Participant Signature: _________________________Date____________________

Witness Name: ____

Witness Signature: _______________________________________Date_________________

As an individual featured in the above photograph, I ……………………………………………. clearly understand the aims of the project entitled "**Insecticide-treated eave baffles for killing malaria mosquitoes and preventing them from entering houses**" and I agree that it may be published or otherwise shared freely in the exact form presented above.

Research Participant Name: ____

Gender (Male/Female: ________________________ Age:_____________________

Research Participant Signature: ________________________Date___________________

Witness Name: ____

Witness Signature: _____________________________________Date________________

As an individual featured in the above photograph, I ………………………………………………. clearly understand the aims of the project entitled "**Insecticide-treated eave baffles for killing malaria mosquitoes and preventing them from entering houses**" and I agree that it may be published or otherwise shared freely in the exact form presented above.

Research Participant Name: ____

Gender (Male/Female: ________________________ Age:_____________________

Research Participant Signature: ________________________Date___________________

Witness Name: ____

Witness Signature: _____________________________________Date________________

As an individual featured in the above photograph, I ………………………………………………. clearly understand the aims of the project entitled "**Insecticide-treated eave baffles for killing malaria mosquitoes and preventing them from entering houses**" and I agree that it may be published or otherwise shared freely in the exact form presented above.

Research Participant Name: ____

Gender (Male/Female: ________________________ Age:_____________________

Research Participant Signature: ________________________Date___________________

Witness Name: ____

Witness Signature: _____________________________________Date________________

As an individual featured in the above photograph, I ………………………………………………. clearly understand the aims of the project entitled "**Insecticide-treated eave baffles for killing malaria mosquitoes and preventing them from entering houses**" and I agree that it may be published or otherwise shared freely in the exact form presented above.

Research Participant Name: ____

Gender (Male/Female: ________________________ Age:_____________________

Research Participant Signature: ________________________Date___________________

Witness Name: ____

Witness Signature: _____________________________________Date________________
