# Supplementary material for: Entomological assessment of hessian fabric transfluthrin vapour emanators as a means to protect against outdoor-biting Aedes after providing them to households for routine use in Port-au-Prince, Haiti
Source: PLoS One. 2024 May 28;19(5):e0298919. doi: 10.1371/journal.pone.0298919 (PMC11132518; doi:10.1371/journal.pone.0298919)
Supplement: S3 Protocol — See also S1 and S2 Protocols for the approved protocol and annexes in English, respectively. (PDF) [file pone.0298919.s004.pdf]

PROTOCOLE DE RECHERCHE

Emanateurs de transfluthrine simples, abordables et évolutifs pour la protection contre la transmission des virus Zika, Dengue et Chikungunya

Christian Raccurt[1], Cyrille Czeher[2], Nicodem J Govella[3], Daniel Impoinvil[4] & Gerry F. Killeen[3,5]

[1] Faculté des Sciences de la Santé, Université Quisqueya, Port-au-Prince, Haïti

[2] Unité MIVEGEC, Institut de Recherche pour le Développement, Montpellier, France

[3] Ifakara Health Institute, Coordination Office, Environmental Health and Ecological Sciences Department, Dar es Salaam and Ifakara, United Republic of Tanzania

[4] Centers for Disease Control and Prevention, Atlanta, United States of America

[5] Department of Vector Biology, Liverpool School of Tropical Medicine, Liverpool, United Kingdom

Résumé du projet

Tandis que la transmission du Zika en Afrique a été historiquement enzoonotique, la nouvelle lignée pandémique du virus s'étendant à travers l'Asie et l'Amérique latine s'est adaptée à la transmission entre les humains [1, 2]. Divers moustiques appartenant aux genres *Aedes*, *Culex*, *Mansonia* et *Anopheles* pourraient agir comme vecteurs, mais la transmission urbaine pandémique à travers l'Afrique, l'Asie et l'Amérique latine apparaît principalement causée par *Aedes aegypti* et *Ae. Albopictus* [3-7]. Les produits répulsifs existants pour la protection contre ces moustiques qui piquent pendant les heures de jour, surtout en plein air, ne durent que quelques heures, jours ou semaines par dose ou application. Le maintien d'une protection continue n'est donc pas pratique et le remplacement répété est inabordable pour les populations à faible revenu. Nous avons récemment développé un émanateur simple [8], qui libère plus lentement la vapeur de transfluthrine répulsive, offrant au moins 4 mois de protection avec une réduction de plus de 90% des piqures des moustiques *Culex* et *Anopheles* dans la ville de Dar es Salaam en Tanzanie, malgré la présence d'une résistance considérable aux pyréthrinoïdes [9]. Ce nouvel émanateur se compose seulement d'une bande de toile de jute (« sac »), qui peut être traitée en toute sécurité et ré-traitée par tout individu, communauté, programme ou fabricant local [8, 9]. En outre, une efficacité et une durabilité équivalentes ont depuis été obtenues avec une dose de transfluthrine 10 fois inférieure soit seulement 1 ml, coûtant seulement US$0,13 par traitement. Nous avons également récemment mis au point un nouveau piège à grille électrique pour mesurer le taux d'attaque des moustiques qui empêche l'exposition de volontaires humains à des piqures potentiellement infectieuses [10]. Nous proposons donc d'appliquer ce nouveau dispositif de piégeage en Haïti pour démontrer que cette nouvelle technologie répulsive fournit au moins 6 mois de protection avec une réduction d'au moins 80% des piqures d'*Aedes aegypti*, probablement le vecteur le plus important de la transmission du Zika à l'échelle mondiale. Dans l'étude proposée ici, le prototype d'émanateur va maintenant être évalué dans des conditions d'utilisation normale et régulière dans les communautés haïtiennes par des utilisateurs représentatifs, dans le but de déterminer le degré et la durée de protection qu'ils apportent, ainsi que leur potentiel pour une utilisation programmatique à plus grande échelle.

Contexte et justificatif

Tandis que la transmission du virus Zika en Afrique a été historiquement enzoonotique, la nouvelle lignée du virus qui a causé une pandémie en se répandant à travers l'Asie et l'Amérique latine s'est adaptée à la transmission entre les humains [1, 2]. Divers moustiques des genres *Aedes, Culex, Mansonia* et *Anopheles* pourraient agir comme vecteurs, mais la transmission urbaine pandémique à travers l'Afrique, l'Asie et l'Amérique latine apparaît principalement due à *Aedes aegypti* et *Ae. Albopictus* [3-7]. Le virus du Zika a été initialement identifié en Ouganda [11, 12], a depuis été détecté dans de nombreux endroits à travers l'Afrique tropicale [4], et se propage maintenant partout dans les tropiques par les chaînes de transmission anthroponotiques par les vecteurs *Aedes* urbains [1-3, 6, 7]. De nouvelles mesures préventives sont nécessaires de toute urgence pour protéger les populations contre ce virus ainsi que les virus de la Dengue et du Chikungunya, également présents dans la région et transmis par les mêmes vecteurs [7].

Il faudra peut-être des années pour développer et évaluer tout vaccin ou traitement contre le virus Zika, et ceux-ci sont susceptibles d'être spécifiques pour ce seul pathogène. Les moustiques *Aedes* qui transmettent le Zika attaquent généralement les gens pendant les heures du jour lorsqu'ils sont éveillés et actifs, souvent à l'extérieur, de sorte que les moustiquaires ou même les habitations protégeant des moustiques offrent une protection limitée. De nouveaux produits répulsifs abordables sont donc nécessaires de toute urgence pour protéger les populations à risque partout dans les zones tropicales contre ces *Aedes* vecteurs piquant à l'extérieur. Les produits anti-moustiques existants protègent uniquement pendant quelques heures, jours ou semaines par

application ou dose, de sorte qu'ils sont trop coûteux et peu pratiques pour une utilisation continue et durable dans les pays à faible revenu comme Haïti [18, 19]. Quelques formulations peuvent même être dangereuses [20].

Nous avons récemment développé un émanateur de transfluthrine de conception simple [8], qui fournit une protection de >90% pendant >4 mois contre les *Anopheles* et *Culex* spp. piquant de nuit et vecteurs du paludisme, de la filariose et de plusieurs arbovirus dans les zones urbaines de Dar es Salaam, en Tanzanie (figure 1) [9]. Dans une étude rurale ultérieure, un certain degré de protection a été fourni pendant 2,5 ans (figure 2) sans détournement des moustiques vers les non-utilisateurs (figure 3). De plus, une efficacité équivalente a été obtenue sur 6 mois avec un dosage de transfluthrine 10 fois plus faible (Figure 4), qui coûte seulement US$0,13 et libère des concentrations de vapeur de seulement 0,00013 mg / m^3, bien inférieure en comparaison de la concentration d'exposition acceptable de 0,5 mg / m^3 [21].

Cet émanateur répulsif pourrait être rapidement étendu à plus grande échelle car il ne se compose que d'une toile de jute largement disponible, qui peut être traité et retraité en toute sécurité sur place par tout individu, communauté, programme ou fabricant local. Le principe actif de la transfluthrine est déjà disponible en vrac chez les fabricants de produits génériques, enregistrés dans le monde entier, non seulement comme un répulsif contre les moustiques, mais aussi contre les mouches, les blattes et autres nuisibles domestiques, causant une meilleure adoption par les utilisateurs (e.g. [21]). Alors que le prototype initial était suspendu sur quatre piquets placés autour de l'utilisateur [8, 9], nous avons maintenant développé un format plus pratique qui est complètement mobile et peut être commodément placé n'importe où l'utilisateur le choisit (Figure 5). Si ces émanateurs s'avèrent efficaces contre le vecteur majeur du Zika *Aedes aegypti*, ils offriront aussi simultanément une protection de jour à large spectre contre la transmission de la dengue, du chikungunya et de la fièvre jaune par les mêmes vecteurs, ainsi qu'une protection nocturne contre d'autres moustiques porteurs de paludisme et de filariose.

De plus, nous avons développé un nouveau prototype de piège à moustiques à grille électrique (figure 7) [10], ce qui nous place dans une position unique pour aborder les objectifs suivants sans exposer les volontaires humains à des piqûres de moustiques potentiellement infectieuses.

But et Objectifs du Projet

Notre objectif général est d'appliquer ce nouveau dispositif de piégeage pour démontrer que cette nouvelle technologie répulsive fournit au moins 6 mois de protection avec une réduction de ≥80% contre *Aedes aegypti*, qui est probablement le vecteur le plus important de transmission de la Dengue, du Chikungunya et du Zika dans le monde entier. Nos objectifs spécifiques, qui doivent être abordés dans un environnement de terrain ayant de fortes densités de ce vecteur en Haïti, sont les suivants :

1. Mesurer le degré et la durée de l'efficacité de protection des prototypes optimisés d'émanateurs utilisés dans la communauté, à l'aide d'indicateurs entomologiques.
2. Evaluer l'efficacité de protection perçue et l'acceptabilité des émanateurs de transfluthrine auprès des utilisateurs, et recueillir leurs avis sur les potentiels freins, opportunités ainsi que les meilleures stratégies de communication pour une utilisation à plus grande échelle.

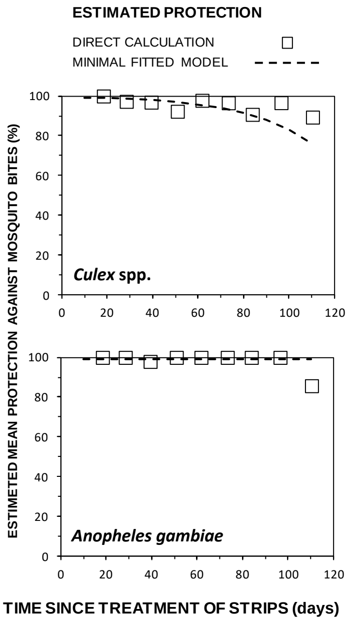

Figure 1. Protection provided by Hessian strips treated with 10ml of Transfluthrin against *Culex* spp. and *Anopheles gambiae* mosquitoes biting outdoors at night in urban Tanzania

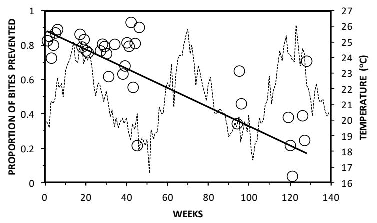

Figure 2. Duration of protection conferred by Hessian strips treated with 10ml of transfluthrin against wild, nocturnal, outdoor-biting *Anopheles arabiensis*, under full open field conditions in rural southern Tanzania.

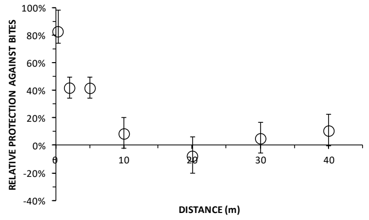

Figure 3. Protective effect of transfluthrin-treated Hessian strips against outdoor-biting *An. arabiensis* for the users (distance 0m) and non-users at a range of distances from a user, under full open field conditions in rural southern Tanzania.

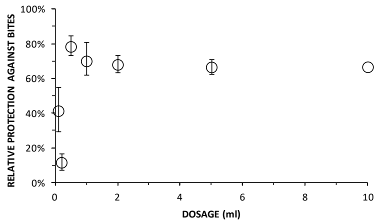

Figure 4. Dose-response curve for transfluthrin-treated Hessian strips under full open-field conditions in rural southern Tanzania

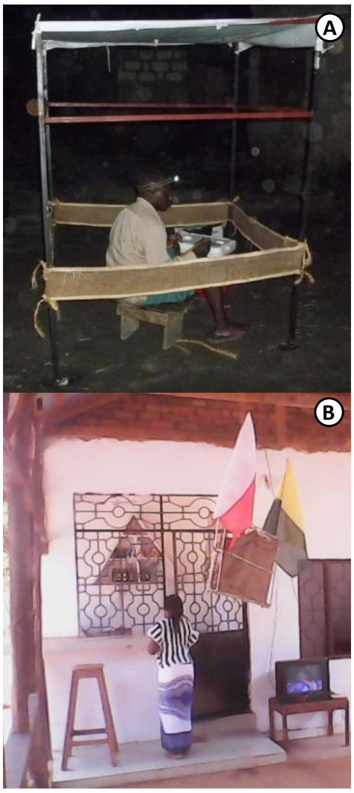

Figure 5. The original prototype of the transfluthrin-treated Hessian strip that will be used in this project for experimental design purposed (A) and the more convenient, scalable format now available hanging in a public bar.

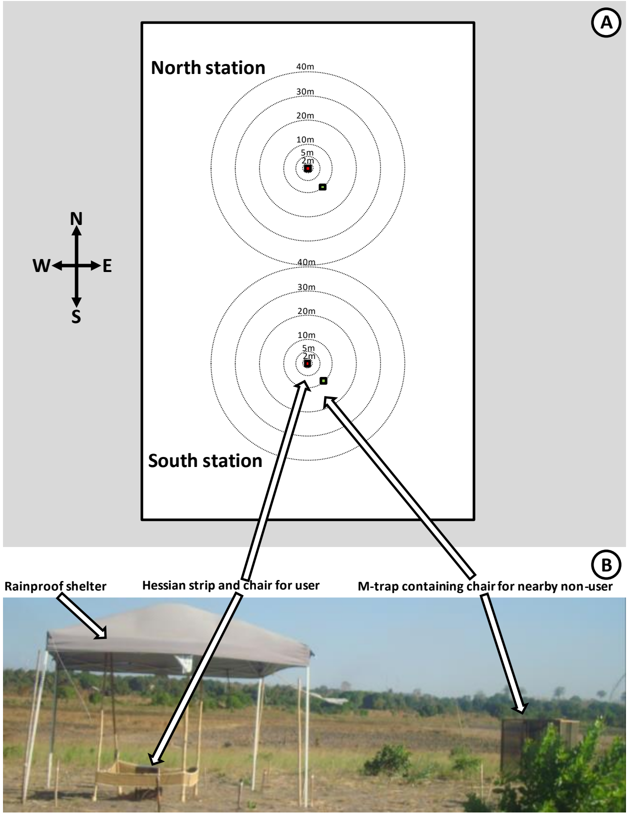

Figure 6. A schematic example of the open-field experimental design used to generate the data presented in figures 2, 3 and 4. Note that both human landing catches by strip users, and large cage trapping by non-users will be replaced by electric grid traps in the study proposed herein.

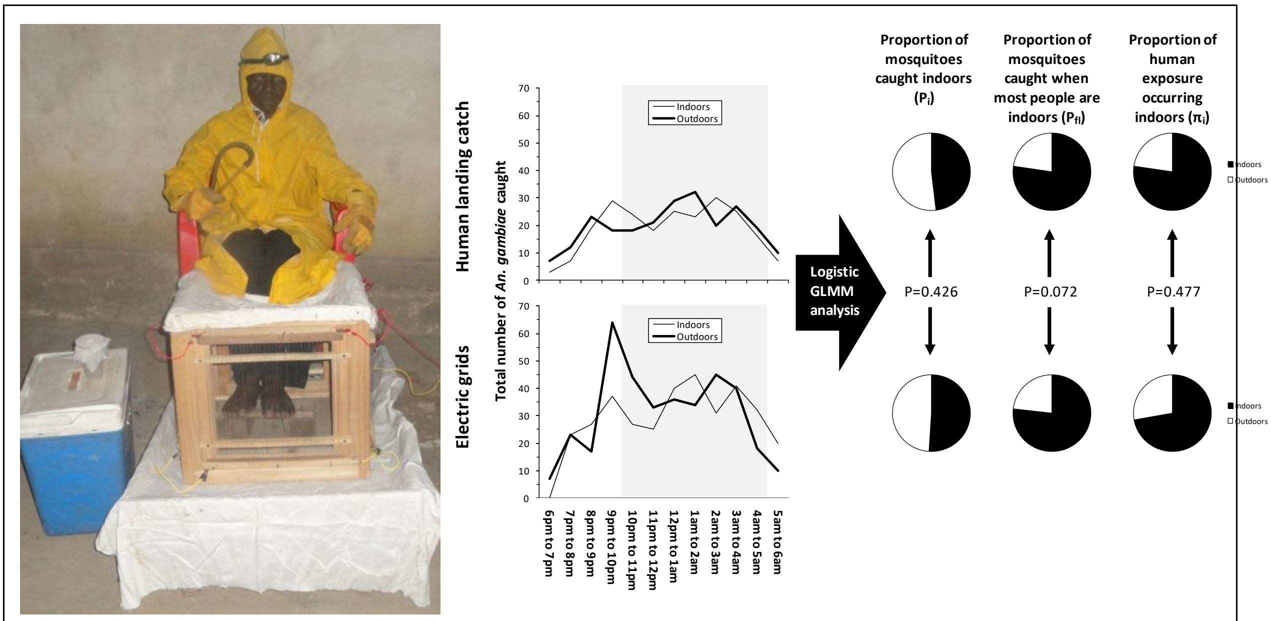

Figure 7. Le panneau de gauche illustre les grilles d'électrocution utilisées par un volontaire humain tandis que le panneau de droite illustre la façon dont ces pièges donnent des estimations des principaux déterminants comportementaux de l'impact du contrôle du paludisme qui sont équivalentes aux résultats obtenus à l'aide de captures sur sujets humains à l'atterrissage (*gold standard*).

Méthodologie

Procédures de terrain et calculs de la taille de l'échantillon

L'émanateur de transfluthrine développé en Tanzanie va maintenant être évalué à Haïti. Il sera évalué en termes, d'une part d'efficacité de protection à l'aide d'indicateurs entomologiques mesurés par les pièges à grille électrique, et d'autre part d'efficacité perçue par les utilisateurs dans la communauté. Ces deux paramètres seront suivis durant toute la durée de l'été haïtien (6 mois de mai à octobre) pour établir comment l'efficacité mesurée ainsi que perçue par les utilisateurs varient selon le temps depuis le traitement et les conditions météorologiques.

Objectif 1 : L'efficacité de protection des émanateurs traités, distribués aux foyers consentants sélectionnés dans la communauté, sera évaluée à l'aide des pièges à grille électrique montrés en figure 7. Voir les annexes 1 à 5 pour toutes les fiches d'information des participants et les formulaires de consentement relatifs à cet objectif.

Huit stations de capture de moustiques seront identifiées dans chaque bloc expérimental, chacune localisée dans un endroit différent de la zone d'étude afin de capturer une variété de conditions environnementales différentes. Les stations de capture seront placées parmi des habitations pour maximiser la densité de moustiques. *Aedes aegypti* se développe dans l'environnement péri-domestique et ne vole pas sur de longues distances, et nous savons par expérience que les densités sont les plus élevées dans ces micro-environnements. Néanmoins, nous choisirons également les emplacements des stations de capture afin de minimiser les dérangements pour les résidents ainsi que les contacts accidentels avec les grilles électriques. Ces dernières seront également entourées d'un grillage dans le but d'éviter qu'enfants et animaux domestiques n'entrent en contact avec le piège sous tension. Chaque jour de travail comprendra la même séquence de 6 périodes de capture d'une heure chacune (3 le matin et 3 le soir). Pour chaque période d'une heure, un jour donné, on

attribuera aléatoirement sans remplacement un des 6 angles (0°, 60°, 120°, 180°, 240° et 300°, par rapport au Nord), qui sera identique pour toutes les 8 stations de capture. Chaque heure, la chaise du collecteur et le piège à grille électrique qu'il utilise seront tournés ensemble autour du centre de la station pour pointer cette direction particulière. De cette manière, toutes les orientations possibles par rapport à la direction du vent (mesurée par une station météorologique miniature placée à proximité) seront représentées dans le jeu de données collectées. L'évaluation entomologique utilisera un échantillonnage en carré latin répliqué, avec 3 paires d'émanateurs traités distribués aux 3 foyers participants (2 par foyer, 6 au total) dans chaque bloc expérimental. Les foyers recrutés donneront également leur consentement pour permettre à l'équipe de recherche de mener des collectes de moustiques durant plusieurs périodes de 8 jours consécutifs, utilisant les 6 émanateurs traités utilisés par les foyers plus une paire (2) d'émanateurs contrôles non traités (détergent et eau uniquement). Ces 8 jours de collecte seront menés une fois tous les deux mois avec deux stations par foyer, à l'intérieur ou à proximité de leur environnement péri-domestique. Deux stations supplémentaires seront établies dans la même zone avec le consentement des foyers concernés pour arriver à un total de 8 stations de collecte et permettre la rotation en carré latin (6 émanateurs traités et deux contrôles négatifs).

Une réplication du protocole expérimental sera complétée dans un bloc par la rotation quotidienne des 8 émanateurs parmi les 8 stations de collecte de manière aléatoire durant la période de 8 jours. Huit personnes volontaires se verront attribuer un des huit émanateurs chaque jour, et collecteront les moustiques avec le même piège à grille électrique tel que décrit dans la figure 7. Chaque volontaire occupera une même station de collecte fixe durant l'ensemble d'une réplication, de manière à ce que les deux sources de variation du taux de capture associées à la station et au volontaire soient combinées en une seule source de variance qui peut être capturée par un unique effet aléatoire et avec une puissance statistique maximum au cours de l'analyse.

Seuls des hommes adultes (≥ 18 ans) et des femmes adultes non en âge de porter des enfants (≥ 50 ans) seront recrutés en tant que volontaires, afin d'éviter tout risque d'infection par Zika, le paludisme ou tout autre pathogène véhiculé par un vecteur auquel Les femmes enceintes sont particulièrement vulnérables.

Dans le but de calculer les tailles d'échantillons, on suppose un taux d'attaque moyen de 20 moustiques par personne par jour, une efficacité minimum d'un émanateur fraichement traité de 80% (Résultant en un taux de 4 piqûres par personne par jour) qui devrait pouvoir être distingué d'un émanateur plus ancien pour lequel l'efficacité a diminué jusqu'à 60% ou moins (8 moustiques ou plus par personne par jour). En supposant une distribution de Poisson de ces données de comptage, puis en appliquant l'équation de Lehr [22], on a pu estimer le nombre minimum d'observations indépendantes nécessaires :

$$N = 4 / [(\lambda_1^{1/2}) + (\lambda_2^{1/2})]^2 = 4 / [(4^{1/2}) + (8^{1/2})]^2 = 5.8$$

On répètera donc 6 fois le protocole en carré latin de 8x8 décrit ci-dessus pour une réplication dans un seul bloc de 8 stations de collecte, en le répétant dans 6 blocs distincts choisis dans différentes parties de la zone d'étude (Probablement Gressier, à proximité de Port au Prince). Chacun des 18 foyers participants se verra remettre 2 émanateurs de transfluthrine fraîchement traités au début de l'expérimentation. Un série complète d'expérimentation dans tous les sites nécessitera 48 jours de travail de terrain (8 jours par bloc et réplication x 6 blocs) qui seront distribués sur une période de travail de 2 mois et permettront ainsi à l'équipe de se reposer et d'être disponible pour d'autres engagements. Trois de ces séries de 2 mois d'évaluation seront complétées au cours des six mois de l'été haïtien, afin de permettre de capturer les variations d'efficacité dues aux conditions météorologiques et à la perte d'ingrédient actif pendant toute la période de plus forte transmission des maladies à transmission vectorielle dans les Caraïbes.

<u>Objectif 2</u> : L'efficacité perçue des émanateurs de transfluthrine par les utilisateurs dans la communauté sera évaluée grâce à une combinaison de méthodes qualitatives complémentaires. Voir les annexes 6 à 12 pour toutes les fiches d'information des participants et les formulaires de consentement éclairé relatifs à cet objectif.

Les 6 émanateurs traités distribués aux foyers dans chaque bloc ne seront récupérés et utilisés pour l'évaluation entomologique de leur efficacité (objectif 1) que pendant 8 jours au cours de chaque cycle d'évaluation de 2 mois. Le reste du temps, ces émanateurs seront utilisés librement par les foyers auxquels ils ont été donnés, après avoir discuté avec l'équipe de recherche sur les modes d'utilisation sûrs et efficaces. Cependant, ils seront aussi encouragés à essayer d'utiliser les émanateurs de manière créative, tant qu'ils n'ouvrent pas le support de protection ou ne l'utilisent d'aucune façon qui pourrait entrainer un contact physique direct avec le matériau imprégné. Pour donner un exemple, l'équipe expliquera comment un des investigateurs place un émanateur devant la porte d'entrée de sa maison pendant la nuit pour prévenir l'entrée de moustiques Culex dans la maison lorsqu'il ouvre la porte tôt le matin [23]. Les participants seront encouragés à utiliser les émanateurs de la manière qu'ils perçoivent comme optimale en termes de commodité et de protection contre les piqûres de moustiques. Chaque ménage participant sera invité à enregistrer ce qu'il perçoit comme les pratiques d'utilisation les plus et les moins efficaces avec les appareils photo jetables qui seront fournis à cet effet, conformément à nos précédentes enquêtes Photovoice (PV) [24-27] à Dar es Salaam [28]. Dans chaque foyer impliqué dans l'étude, un participant au volet photographique sera chargé de prendre des photos pour le compte de l'ensemble du ménage. Avant d'utiliser ces appareils photo, les participants à ce volet de l'étude participeront à une courte réunion de formation pour expliquer le sujet de l'enquête, les principes de protection de la confidentialité des données personnelles et décrire les manières acceptables d'utiliser la caméra sans compromettre la sécurité, la vie privée, ou d'autres droits des individus ou de la communauté dans son ensemble [28].

Chaque ménage sera visité une fois tous les deux mois lorsque les expériences d'efficacité sont entreprises, et enquêté avec un bref questionnaire semi-structuré pour évaluer leur niveau de satisfaction avec le niveau perçu de protection contre les piqûres de moustiques. Tous les membres adultes du ménage consentants seront interrogés à chaque occasion. La satisfaction perçue sera estimée numériquement sur une échelle graduée de 0 à 5, avec des scores séparés enregistrés pour l'exposition intérieure et extérieure. Au début de chaque visite d'un bloc, tous les appareils photo jetables entièrement utilisées seront collectées afin que les photos puissent être développées en double. Un exemplaire sera retourné au ménage en quelques jours [28] tandis que l'autre sera conservé par l'équipe de recherche en cas de perte avant les discussions de groupe Photovoice (DGPV) décrites ci-dessous.

À la fin de l'étude, quatre groupes de discussion thématiques (GDT) seront menés pour l'ensemble des blocs expérimentaux (un participant volontaire de chaque sexe de chacun des 3 ménages participants dans 3 blocs par groupe). Les participants aux groupes de discussion incluront des hommes et des femmes adultes dans des groupes distincts, et ces quatre groupes seront interviewés séparément pour améliorer la participation. Ces discussions semi-structurées seront menées en créole haïtien en utilisant un bref guide thématique (Annexe 9) pour obtenir des aperçus thématiques sur la façon dont ils perçoivent la valeur, les avantages et les inconvénients, le caractère abordable et la facilité d'utilisation des dispositifs. À la fin de ces discussions, les enquêteurs solliciteront également les participants pour obtenir des conseils sur la meilleure façon de faire progresser cette technologie grâce à d'autres modifications du produit, à l'évaluation programmatique et à la recherche opérationnelle (annexe 9). Pour chaque discussion de groupe, un facilitateur coordonnera la discussion et un observateur prendra des notes sur les formes de communication verbales et non verbales.

Une fois que toutes les photos auront été développées et retournées aux participants photographes, ils seront engagés dans un processus d'analyse participative en deux étapes ; en sélectionnant les photographies pour la discussion et ensuite en les contextualisant et en racontant leur histoire. Au cours de la première étape, les photographies développées seront remises aux photographes à la fin de l'étude, et chacun disposera d'environ une semaine pour sélectionner les 10 photos qu'il/elle considère comme les meilleures ou les plus significatives. En sélectionnant les photographies pour la discussion, les participants vont donner la direction générale des discussions de groupe PV (DGPV) à venir [26, 27]. La deuxième étape consistera à contextualiser ou raconter les histoires derrière les photographies et ce que les photos représentent pour le photographe, pendant les DGPV. Chaque participant montrera ses photos sur une table, les présentera au groupe, donnera leur signification, et expliquera son interprétation des images (Annexe 11). Ces DGPV seront conduites de manière informelle, mais basées sur une version adaptée du model SHOWeD [26, 27]. À ce stade de la discussion, chaque photographe identifiera les différents thèmes qui ont émergé après avoir réexaminé le contenu de leurs photographies et se souvenir où, quand et pourquoi ils les ont prises. Il s'en suivra une discussion plus spécifique sur les avantages, les inconvénients et les limites de ces dispositifs, les facteurs influençant leur utilisation et les idées pour améliorer les dispositifs eux-mêmes ou pour une distribution et un entretien optimaux à l'avenir (Annexe 11). À la fin de la discussion, les participants sélectionneront les 10 meilleures photos de toutes les photographies prises par le groupe.

Tous les entretiens et discussions de groupe seront menés en créole haïtien et des enregistrements audio numériques des groupes de discussion et des DGPV seront réalisés. Ces enregistrements seront ensuite transcrits textuellement (avec les identifiants supprimés) et traduits en anglais, sous forme de documents Microsoft Word®.

Méthodes analytiques quantitatives et qualitatives

Objectif 1: Tout d'abord, un modèle mixte linéaire généralisé (GLMM) avec une distribution de Poisson séparé sera ajusté à chaque sous-ensemble de données comprenant une réplication expérimentale complète de deux mois du protocole d'étude, exactement comme décrit ci-dessus pour l'objectif 1. De plus, un GLMM supplémentaire sera ajusté à la période complète de 6 mois de données longitudinales, comprenant 3 réplications expérimentales complètes, avec le temps écoulé depuis le traitement par la transfluthrine (mais pas le placebo) inclus comme variable continue, pour permettre d'estimer le taux de décroissance de l'efficacité. Les résultats seront présentés de manière similaire aux figures 1 et 2.

Objectif 2: Un mélange de méthodes qualitatives (analyse thématique du contenu non structuré des questionnaires mensuels, photos associées et final FGD) et quantitatives (analyses de distribution de fréquence, tests de rangs non paramétriques et GLMM) sera utilisé pour analyser et interpréter ces données, comme nous l'avons déjà fait en relation avec des questions d'accès à d'autres interventions de lutte contre les moustiques [28-30].

Les données semi-quantitatives recueillies, sous la forme de niveaux catégoriques de satisfaction avec la protection fournie par les émanateurs de transfluthrine, seront principalement analysées graphiquement en comparant la moyenne/médiane et les distributions de ces perceptions enregistrées aux estimations entomologiques quantitatives (Objectif 1). Le test de rang de Spearman et les GLMM seront également utilisés pour tester les associations entre ces indicateurs de protection subjectivement perçus et objectifs mesurés. Un objectif spécifique de cette analyse est d'identifier au moins approximativement les seuils d'efficacité protectrice ou d'exposition aux piqûres qui sont systématiquement considérés comme satisfaisants par les utilisateurs finaux.

Pour les données qualitatives issues des groupes de discussion (FGD et PV-FGD), l'analyse sera conduite de manière itérative, en adoptant une approche cadre impliquant cinq étapes clés d'analyse [31]: 1) la familiarisation par la transcription et la lecture des données; 2) l'identification d'un cadre thématique, élaboré à partir d'une combinaison de questions a priori et de problèmes qui ont émergé durant la phase de familiarisation; 3) l'indexation, également appelée codage, en appliquant le cadre thématique pour classer les données; 4) Créer des tableaux à partir des données codées; et 5) cartographie et interprétation; rechercher des modèles et des associations et tester les explications et interprétations possibles en termes de plausibilité logique et de cohérence avec l'ensemble de données complet. Pour assurer la triangulation de différentes perspectives, l'équipe de recherche partagera régulièrement les résultats qui ont émergé pour optimiser le processus par consensus.

Durée de l'étude

Le travail de terrain dans le cadre de cette étude durera 6 mois, du 1er mai au 31 octobre 2018, à la suite de quoi l'analyse, la rédaction et le reporting s'étendront sur 4 mois supplémentaires jusqu'au 28 février 2019.

Considérations éthiques

Aucun participant ne sera exposé à un risque accru d'exposition aux piqûres de moustiques ou à tout agent pathogène transmis par les moustiques, car un nouveau piège à grille électrique [10] sera utilisé pour tuer et retenir les moustiques qui tentent d'attaquer les volontaires humains. Les bénévoles assis dans le piège à grille électrique seront entièrement protégés contre l'exposition à l'électrocution avec un grillage intérieur en plastique isolant. Tous les risques potentiels associés à la collecte de renseignements personnels sont mineurs et seront minimisés comme suit.

Risques potentiels

Objectif 1 : Le piège à grille électrique est un outil sans exposition humaine pour attraper les moustiques à la recherche d'un hôte, ce qui signifie qu'il attrape et tue les moustiques avant qu'ils ne puissent piquer, de sorte que les volontaires humains ne sont pas exposés à un risque accru d'infections transmises par les moustiques. L'outil est composé de 4 panneaux avec treillis métallique électrifié dans les brins sont écartés de 5 mm, formant un carré. Les bénévoles sont assis sur une chaise avec leurs jambes placées dans un cadre carré tandis que le reste du corps est protégé contre les piqûres de moustiques en portant des chapeaux et chemises / vestes à manches longues (Figure 7). De l'intérieur le cadre carré de PVC bois est tapissé d'un grillage en plastique isolant qui protège les membres des volontaires contre tout contact possible avec les fils électrifiés extérieurs qui transporte le courant continu à basse tension pour l'électrocution des moustiques.

La concentration de vapeur de transfluthrine libérée par ces dispositifs émanateurs a été mesurée à seulement 0,00013 mg/m^3, bien inférieure (<1 / 1000e) à la concentration d'exposition acceptable de 0,5 mg/m^3 [16], et est donc considérée comme présentant un risque négligeable. Aucun investigateur ne sera exposé à une concentration plus élevée de vapeur de transfluthrine dans l'air que les participants pendant les expérimentations. Comme on l'a décrit ci-dessus pour les participants, ils travailleront donc avec des concentrations 1000 fois au-dessous des limites maximales acceptables pour l'exposition chronique à cet insecticide, ce qui est considéré comme négligeable après des décennies d'utilisation dans une pléthore de produits ménagers courants. Cependant, il existe des risques mineurs d'exposition aiguë à la transfluthrine lors de la formulation des émanateurs utilisant un stock de transfluthrine initialement non dilué.

Objectif 2 : Il existe des risques mineurs pour la vie privée et la sécurité associés à la diffusion de photographies de l'extérieur ou de l'intérieur des maisons des participants ou de leur contenu. De plus, des risques mineurs pour la vie privée et la sécurité peuvent être encourus en présentant les traits du visage ou d'autres informations personnelles identifiables dans ces photographies publiées. Les seuls autres renseignements personnels à être collectés auprès des participants seront leur nom, leur âge et leur sexe, mais aucune de ces données ne sera publiée, diffusée ou accessible au public.

Minimiser les risques pour les participants

Objectif 1 : Les participants auront toutes les occasions de discuter de l'étude et toutes les questions qu'ils auront seront traitées. Le consentement éclairé sera documenté et les participants pourront se retirer à tout moment. Dans le cas du retrait d'un collecteur de moustiques avant l'achèvement de l'étude, nous le remplacerons dès que possible par un nouveau volontaire et continuerons le protocole expérimental tel que décrit ci-dessus. Toutes ces études seront effectuées pendant les heures de grand jour, lorsque les vecteurs antipaludéens Anophèles ne seront plus actifs et que les volontaires seront protégés contre *Aedes agypti* par les grilles électriques et les vêtements de protection. Ainsi, contrairement aux études antérieures sur l'exposition aux moustiques que nous avons menées, aucun médicament contre le paludisme ou dépistage régulier de l'infection de paludisme n'est requis ou ne sera fourni aux volontaires. En outre, seuls les hommes adultes (≥ 18 ans) et les femmes adultes plus en âge de porter des enfants (≥ 50 ans) seront recrutés en tant que volontaires, afin d'éviter tout risque d'infection par Zika, le paludisme ou tout autre pathogène véhiculé par un vecteur auquel Les femmes enceintes sont particulièrement vulnérables.

Afin d'éviter tout risque pour les investigateurs de l'exposition aiguë à la transfluthrine lors de la formulation des émanateurs, ils porteront des gants, des vêtements de protection, des lunettes de sécurité et des masques lors de la préparation des émanateurs.

Objectif 2 : Les risques associés à la dissémination de photographies de l'extérieur et de l'intérieur des maisons des participants seront atténués en demandant une autorisation écrite avant qu'une photographie ne soit publiée ou partagée avec quelqu'un d'autre que les enquêteurs, avec tout visage ou autre information personnelle identifiable masqués si le participant le demande (Annexe 3). À la demande des participants, nous masquerons également tous les éléments personnellement identifiables ou sensibles des photographies, tels que leurs effets personnels ou les précautions de sécurité. Les seuls autres renseignements personnels qui seront recueillis auprès des participants seront leur nom, leur âge et leur sexe, qui seront gardés confidentiellement, avec les copies papier originales stockées dans des classeurs verrouillés, et tous les fichiers électroniques contenant ces données ainsi que les ordinateurs sur lesquels ils sont stockés seront protégés par mot de passe.

Avantages potentiels de l'étude pour les participants et autres

Les participants ne tireront pas de bénéfice de leur participation autre qu'une rémunération pour leur temps et leur inconfort à un taux de 15 US$ par jour. Les rémunérations sont régulièrement effectuées en dollars américains en raison de l'instabilité de la Gourde haïtienne. Le montant a été fixé pour trouver un équilibre entre être assez pour fournir une compensation équitable pour le temps et l'inconfort, sans inciter les volontaires à participer malgré toutes les réserves qu'ils pourraient avoir.

Les résultats de cette étude auront des répercussions directes et significatives sur la politique en ce qui concerne la mise en œuvre de stratégies de lutte anti vectorielle et de prévention des maladies, comme cela a été souligné dans la section Contexte et Justification. Si ces émanateurs se révèlent efficaces contre *Aedes aegypti*, ils peuvent non seulement être déployés pour protéger contre les flambées de Zika qui pourraient se produire à l'avenir, mais ils peuvent également être déployés pour fournir une protection simultanée et à large spectre pendant la journée contre la transmission

continue de la Dengue et du Chikungunya par les mêmes vecteurs, ainsi que la protection nocturne contre les autres moustiques porteurs de la malaria et de la filariose.

Consentement Eclairé

La fiche d'information du participant et le formulaire de consentement éclairé seront fournis aux participants potentiels pour qu'ils les lisent eux-mêmes en détail. Ensuite, ces deux documents seront également lus en entier et expliqués en créole haïtien aux participants éventuels à l'étude. Les participants potentiels auront alors l'occasion de poser des questions et d'exprimer leurs préoccupations, qui seront abordées oralement par l'équipe de l'étude, se référant aux enquêteurs chaque fois que nécessaire pour toutes les questions imprévues que l'équipe de terrain ne se sente pas capable de répondre. Le participant documentera ensuite le consentement éclairé par écrit en signant le formulaire de consentement éclairé en présence d'un témoin qui signera également pour valider l'authenticité du processus et l'approbation du consentement. Pour les participants consentants qui ont une capacité limitée à lire ou à écrire, leur nom sera rempli pour eux, et le consentement sera documenté avec leur empreinte du pouce à l'encre au lieu de leur signature.

Dissémination

Les résultats de ce projet seront publiés dans des revues à comité de lecture et d'accès libre afin d'intéresser la communauté scientifique internationale. Les résultats seront également présentés aux acteurs gouvernementaux du Ministère de la Santé Publique et de la Population, aux partenaires étrangers et locaux du MSPP ainsi qu'aux médias locaux tels que les médias imprimés, radiophoniques et télévisuels. Les résultats seront également présentés directement aux résidants de la zone d'étude de Gressiers, par le biais de réunions communautaires. Les résultats seront également diffusés par des partenaires commerciaux locaux potentiels qui pourraient potentiellement enregistrer et fournir des dispositifs d'émanation et des sachets de transfluthrine pré-dilués pour les traiter et les retraiter.

Gestion de données

Les échantillons de moustiques seront mis en tubes, étiquetés et stockés dans des congélateurs de stockage d'échantillons verrouillés. Les données collectées sous forme papier seront également stockées en toute sécurité dans des classeurs verrouillés dans une pièce à accès restreint. Les données électroniques seront stockées sur des serveurs sécurisés, sauvegardés de manière régulière, à l'Université Quisqueya et à l'IHI. Ce serveur central utilise un nouveau schéma innovant [32] qui permet de stocker toutes les données entomologiques dans seulement 4 tables de données spécifiques à l'entomologie avec des liens relationnels prédéfinis, permettant une synthèse et une analyse rapides, voire automatisées.

Partage de données

L'accès et l'utilisation des données devront être conformes aux politiques de partage de données de l'Université Quisqueya. Si des données sont demandées et qu'aucun intérêt concurrent n'est apparent, les données demandées seront mises à disposition dans des conditions définies, exprimées par un échange de lettres entre les parties stipulant ces conditions et toute limite convenue d'utilisation des données.

Budget

Cette étude est financée par l'Agence Américaine pour le Développement International (USAID), à travers un sous-contrat de l'École de Médecine Tropicale de Liverpool (LSTM).

Description	Montant (US$)
Personnel	$136,124
Matériels, fournitures équipement	$8,393
Transport	$16,240
Espace de bureau	$8,526
Soutien informatique	$1,462
Divers coûts réglementaires et de dissémination	$9,300
Frais de voyage et de subsistance	$4,000
Coûts indirects (10%)	$18,405
**Total**	**$202,450**

Références

1. Freire CCdM, Lamarino A, Neto DFdL, Sall AA, Zanotto PMdA. Spread of the pandemic Zika virus lineage is associated with NS1 codon usage adaptation in humans (Online pre-print). BioRxiv. 2016;DOI:10.1101/032839.
2. Zanluca C, de Melo VC, Mosimann AL, Dos Santos GI, Dos Santos CN, Luz K. First report of autochthonous transmission of Zika virus in Brazil. Memorias do Instituto Oswaldo Cruz. 2015;110(4):569-72. Epub 2015/06/11. doi: 10.1590/0074-02760150192. PubMed PMID: 26061233; PubMed Central PMCID: PMC4501423.
3. Ayres CF. Identification of Zika virus vectors and implications for control. Lancet Infect Dis. 2016. Epub 2016/02/09. doi: 10.1016/S1473-3099(16)00073-6. PubMed PMID: 26852727.
4. Faye O, Freire CC, Iamarino A, de Oliveira JV, Diallo M, Zanotto PM, Sall AA. Molecular evolution of Zika virus during its emergence in the 20th century. PLoS Negl Trop Dis. 2014;8(1):e2636. Epub 2014/01/15. doi: 10.1371/journal.pntd.0002636. PubMed PMID: 24421913; PubMed Central PMCID: PMC3888466.
5. Diallo D, Sall AA, Diagne CT, Faye O, Ba Y, Hanley KA, Buenemann M, Weaver SC, Diallo M. Zika virus emergence in mosquitoes in southeastern Senegal, 2011. PLoS One. 2014;9(10):e109442. Epub 2014/10/14. doi: 10.1371/journal.pone.0109442. PubMed PMID: 25310102; PubMed Central PMCID: PMC4195678.
6. Grard G, Caron M, Mombo IM, Nkoghe D, Mboui Ondo S, Jiolle D, Fontenille D, Paupy C, Leroy EM. Zika virus in Gabon (Central Africa)--2007: a new threat from Aedes albopictus? PLoS Negl Trop Dis. 2014;8(2):e2681. Epub 2014/02/12. doi: 10.1371/journal.pntd.0002681. PubMed PMID: 24516683; PubMed Central PMCID: PMC3916288.
7. Musso D, Cao-Lormeau VM, Gubler DJ. Zika virus: following the path of dengue and chikungunya? Lancet. 2015;386(9990):243-4. Epub 2015/07/22. doi: 10.1016/S0140-6736(15)61273-9. PubMed PMID: 26194519.
8. Ogoma SB, Ngonyani H, Simfukwe ET, Mseka A, Moore J, Killeen GF. Spatial repellency of transfluthrin-treated hessian strips against laboratory-reared *Anopheles arabiensis* mosquitoes in a semi-field tunnel cage. Parasit Vectors. 2012;5:54. Epub 2012/03/22. doi: 10.1186/1756-3305-5-54. PubMed PMID: 22433128; PubMed Central PMCID: PMC3338372.
9. Govella NJ, Ogoma SB, Paliga J, Chaki PP, Killeen G. Impregnating hessian strips with the volatile pyrethroid transfluthrin prevents outdoor exposure to vectors of malaria and lymphatic filariasis in urban Dar es Salaam, Tanzania. Parasit Vectors. 2015;8:322. Epub 2015/06/13. doi: 10.1186/s13071-015-0937-8. PubMed PMID: 26063216; PubMed Central PMCID: PMC4465323.
10. Maliti DV, Govella NJ, Killeen GF, Mirzai N, Johnson PCD, Kreppel K, Ferguson HM. Development and evaluation of mosquito-electrocuting traps as alternatives to the human landing catch technique for sampling host-seeking malaria vectors Malar J. 2015;14:502. doi: 10.1186/s12936-015-1025-4.
11. Dick GW. Zika virus. II. Pathogenicity and physical properties. Trans R Soc Trop Med Hyg. 1952;46(5):521-34. Epub 1952/09/01. PubMed PMID: 12995441.
12. Dick GW, Kitchen SF, Haddow AJ. Zika virus. I. Isolations and serological specificity. Trans R Soc Trop Med Hyg. 1952;46(5):509-20. Epub 1952/09/01. PubMed PMID: 12995440.
13. Mboera LE, Mweya CN, Rumisha SF, Tungu PK, Stanley G, Makange MR, Misinzo G, De Nardo P, Vairo F, Oriyo NM. The Risk of Dengue Virus Transmission in Dar es Salaam, Tanzania during an Epidemic Period of 2014. PLoS Negl Trop Dis. 2016;10(1):e0004313. Epub 2016/01/27. doi: 10.1371/journal.pntd.0004313. PubMed PMID: 26812489; PubMed Central PMCID: PMC4728062.
14. Vairo F, Mboera LEG, De Nardo P, Oriyo NM, Meschi S, Rumisha SF, Colavita F, Mhina A, Carletti F, Mwakapeje E, Capobianchi MR, Castilletti C, Di Caro A, Nicastri E, Malecela MN, Ippolito G. Clinical, Virologic, and Epidemiologic Characteristics of Dengue Outbreak, Dar es Salaam, Tanzania, 2014. Emerg Infect Dis. 2016;22:ePub ahead of print.
15. Chipwaza B, Mugasa JP, Selemani M, Amuri M, Mosha F, Ngatunga SD, Gwakisa PS. Dengue and Chikungunya fever among viral diseases in outpatient febrile children in Kilosa district hospital, Tanzania. PLoS Negl Trop Dis. 2014;8(11):e3335. doi: 10.1371/journal.pntd.0003335. PubMed PMID: 25412076; PubMed Central PMCID: PMCPMC4239002.
16. Weller N, Clowes P, Dobler G, Saathoff E, Kroidl I, Ntinginya NE, Maboko L, Loscher T, Hoelscher M, Heinrich N. Seroprevalence of alphavirus antibodies in a cross-sectional study in southwestern Tanzania suggests endemic circulation of chikungunya. PLoS Negl Trop Dis. 2014;8(7):e2979. doi: 10.1371/journal.pntd.0002979. PubMed PMID: 25079964; PubMed Central PMCID: PMCPMC4117434.

17. Crump JA, Morrissey AB, Nicholson WL, Massung RF, Stoddard RA, Galloway RL, Ooi EE, Maro VP, Saganda W, Kinabo GD, Muiruri C, Bartlett JA. Etiology of severe non-malaria febrile illness in Northern Tanzania: a prospective cohort study. PLoS Negl Trop Dis. 2013;7(7):e2324. doi: 10.1371/journal.pntd.0002324. PubMed PMID: 23875053; PubMed Central PMCID: PMCPMC3715424.
18. Achee NL, Bangs MJ, Farlow R, Killeen GF, Lindsay S, Logan JG, Moore SJ, Rowland M, Sweeney K, Torr SJ, Zwiebel LJ, Grieco JP. Spatial repellents: from discovery and development to evidence-based validation. Malar J. 2012;11:164. doi: 10.1186/1475-2875-11-164.
19. Killeen GF. Characterizing, controlling and eliminating residual malaria transmission. Malar J. 2014;13:330. Epub 2014/08/26. doi: 10.1186/1475-2875-13-330. PubMed PMID: 25149656; PubMed Central PMCID: PMC4159526.
20. Hogarh JN, Antwi-Agyei P, Obiri-Danso K. Application of mosquito repellent coils and associated self-reported health issues in Ghana. Malar J. 2016;15:61. doi: 10.1186/s12936-016-1126-8.
21. EUStandingCommitteeonBiocidalProducts. Evaluation of active substances assessment report: Transfluthrin (insecticides, acaricides and products to control other arthropods). 2014.
22. van Belle G. Sample size. Statistical rules of thumb. Wiley Series in Probability and Statistical Series. 2 ed: Wiley; 2008. p. 27-51.
23. Ogoma SB, Mmando A, Kyeba JS, Horstmann S, Malone D, Killeen GF. A low technology emanator treated with the volatile pyrethroid Transfluthrin confers long term protection against outdoor-biting vectors of lymphatic filariasis, arboviruses and malaria. PLoS Negl Trop Dis. 2017;11:e0005455.
24. Iskander D. Re-imaging malaria in the Philippines: how photovoice can help to re-imagine malaria. Malar J. 2015;14:257.
25. Iskander D. Parasites, Power, and Photography. Trend Parasitol. 2016;32:2-3. doi: 10.1016/j.pt.2015.11.002.
26. Wang C, Burris MA. Photovoice: Concept, methodology, and use for participatory needs assessment. Health Educ Behav. 1997;24:369-87.
27. Wang CC. Photovoice: A participatory action research strategy applied to women's health. J Womens Health. 1999;8:185-92.
28. Makungu C, Stephen S, Kumburu S, Govella NJ, Dongus S, Hildon ZJL, Killeen GF, Jones C. Informing new or improved vector control tools for reducing the malaria burden in Tanzania: a qualitative exploration of perceptions of mosquitoes and methods for their control among the residents of Dar es Salaam Malar J. 2017;16:410. doi: 10.1186/s12936-017-2056-9.
29. Chaki PP, Dongus S, Kannady K, Fillinger U, Kelly A, Killeen GF. Community-Owned Resource Persons for malaria vector control: enabling factors and challenges in an operational programme in Dar es Salaam, Tanzania. Human Res Health. 2011;9:21.
30. Chaki PP, Govella NJ, Shoo B, Hemed A, Tanner M, Fillinger U, Killeen GF. Achieving high coverage of larval-stage mosquito surveillance: challenges for a community-based mosquito control programme in urban Dar es Salaam, Tanzania. Malar J. 2009;8(1):311. Epub 2010/01/01. doi: 1475-2875-8-311 [pii] 10.1186/1475-2875-8-311. PubMed PMID: 20042071; PubMed Central PMCID: PMC2806382.

31. Richie J, Lewis J. Qualitative research: a guide for social science student and researcher. London: Sage Publications; 2003.
32. Kiware SS, Russell TL, Mtema ZJ, Malishee AD, Chaki P, Lwetoijera D, Chanda J, Chinula D, Majambere S, Gimnig JE, Smith TA, Killeen GF. A generic schema and data collection forms applicable to diverse entomological studies of mosquitoes. Source Code Biol Med. 2016;11:4. doi: 10.1186/s13029-016-0050-1. PubMed PMID: 27022408; PubMed Central PMCID: PMCPMC4809029.

Notice biographique
(Avril 2018)

Christian Pierre RACCURT
Nationalité française
Né le 28 avril 1943 à Bourg-en-Bresse, France
Médecin, biologiste, professeur émérite des universités

Diplômes
- *Docteur en médecine* (**Lyon, 1970**)
- *Certifié en médecine tropicale* (**Lubumbashi, 1971**)
- *Certifié en parasitologie médicale technique* (**Lyon, 1973**)
- *Licencié en sciences naturelles* (**Dakar, 1974**)
- *Docteur d'État ès sciences* (**Lyon, 1984**)
- *Diplômé de mycologie médicale* (**Paris, 1992**)

Grades universitaires
- *Maître de conférences des Universités* (**1984-1992**)
- *Professeur des Universités* (**1992-2009**)
- *Professeur émérite de la Faculté de médecine d'Amiens* (**2009-2012**)

Distinctions honorifiques haïtiennes
- *Plaque Honneur et Mérite*, *Association médicale haïtienne* (avril 2001)
- *Plaque d'Honneur pour contribution scientifique*, *GHESKIO* (décembre 2001)
- *Plaque Honneur et Mérite pour contribution scientifique*, *Université Quisqueya* (janvier 2006)

Décorations françaises
- *Mérite national* au grade de *Chevalier* en 2002
- *Palmes académiques* au grade de *Chevalier* en 2009
- *Palmes académiques* au grade d'*Officier* en 2012

Carrière professionnelle et universitaire
- Pendant 15 ans, j'ai été en poste au titre de la Coopération française successivement à **Lubumbashi**, *République démocratique du Congo* **(1969-1971)**, **Kinshasa**, *Zaïre* **(1971-1972)**, **Dakar**, *Sénégal* **(1972-1974)**, et **Port-au-Prince**, *Haïti* **(1974-1984)**.
- En Haïti, j'ai été reconnu *expert international de la filariose lymphatique* par l'**Organisation mondiale de la Santé**. Responsable du laboratoire de microbiologie de la Faculté de médecine et de Pharmacie de l'Université d'État d'Haïti, j'ai dirigé des recherches sur les filarioses dues à *Wuchereria bancrofti* et à *Mansonella ozzardi* et sur leurs vecteurs (*Culex quinquefasciatus*, *Culicoides furens* et *C. barbosai*, *Leptoconops bequaerti*), en partenariat avec *Tulane University* (New Orleans, USA), *les Facultés de médecine de Strasbourg et de Lyon* (France) et le *Muséum national d'Histoire naturelle de Paris* (France).

- De retour en France en juillet 1984, j'ai poursuivi pendant 25 ans une carrière hospitalo-universitaire, successivement affecté à **Bordeaux (1984-1990)** et à **Pointe-à-Pitre (1990-1992)**, comme *Maître de conférences des universités, praticien hospitalier*, puis à **Cayenne (1992-1995)** et **Amiens (1996-2009)** comme *Professeur des universités, praticien hospitalier*.
- À Amiens, j'ai mis en place une *convention interuniversitaire* entre l'*Université Jules Verne de Picardie* (**UPJV**) et l'*Université Notre Dame d'Haïti* (**UNDH**) où j'ai assuré plusieurs missions d'enseignement. J'ai également œuvré à la mise en place de stages d'été dans les services du Centre hospitalier universitaire d'Amiens au profit d'étudiants en médecine haïtiens finissants (UNDH et Quisqueya).
- De 2001 à 2004, j'ai dirigé le **Bureau Caraïbe** de **l'Agence universitaire de la Francophonie** (**AUF**) à **Port-au-Prince (Haïti)**.
- J'ai terminé ma carrière hospitalo-universitaire comme *professeur de première classe à la Faculté de médecine d'Amiens et chef de service du laboratoire de parasitologie et de mycologie médicales du Centre hospitalier universitaire d'Amiens*.
- Admis à faire valoir mes droits à la retraite le 1er septembre 2009, je suis depuis installé en Haïti où j'ai été consultant au *Laboratoire national de Santé publique* jusqu'au 30 septembre 2014, œuvrant à l'amélioration de la qualité du diagnostic du paludisme, effectuant des tournées de supervision des laboratoires publiques sur l'ensemble du territoire et des sessions de mise à niveau pour les techniciens de laboratoire, réalisant des recherches sur l'épidémiologie du paludisme et l'étude de la sensibilité à la chloroquine des souches haïtiennes de *Plasmodium falciparum*.
- Je suis associé aux études en cours sur le paludisme en Haïti menées par *University of Florida (États-Unis d'Amérique)*.
- J'ai participé à l'enseignement de la médecine tropicale dans les Facultés de médecine des *universités Quisqueya* (**uniQ**) et *Notre-Dame d'Haïti* (**UNDH**).
- Actuellement je dirige la *Faculté des sciences de la santé de l'Université Quisqueya* (**uniQ**) en tant que *Doyen* depuis le 1er septembre 2017.
- À ce jour, j'ai publié, en tant qu'auteur ou co-auteur, près de 200 articles scientifiques dont **125 sont référencés dans PubMed** (*US National Library of Medicine National Institutes of Health*).

Emanateurs de transfluthrine simples, abordables et évolutifs pour protéger contre la transmission des virus Zika, Dengue et Chikungunya

ANNEXE 1
FICHE D'INFORMATION DES PARTICIPANTS AUX CAPTURES DE MOUSTIQUES

Les moustiques noirs avec des marques blanches qui piquent habituellement pendant les heures du jour transmettent les virus de la dengue et du chikungunya, qui sont des causes courantes de fièvre, de douleurs corporelles et d'un certain nombre d'autres symptômes ici en Haïti. En plus, ce moustique transmet également le virus Zika, qui cause de graves malformations chez les enfants de femmes enceintes qui ont été infectées. Parce qu'ils piquent à l'extérieur pendant la journée quand les gens sont généralement actifs, ni les moustiquaires ni les logements protégés des moustiques ne peuvent fournir une protection satisfaisante contre ces maladies. Malheureusement, les spirales anti-moustiques et les répulsifs pour la peau protègent seulement quelques heures après application, ce qui les rend trop chers et peu pratiques pour une utilisation continue.

L'équipe de recherche responsable de cette étude a récemment développé un émanateur de transfluthrine simple, qui fournit plus de 90% de protection pendant plus de 4 mois contre les moustiques qui piquent la nuit en Afrique.

Nous vous invitons donc à participer à une étude pour évaluer ce nouvel outil de prévention des piqûres de ces moustiques piquant la journée ici en Haïti. Le but de l'étude est de démontrer que cette nouvelle reformulation de la transfluthrine peut fournir une protection abordable contre ces moustiques importants pendant au moins 6 mois.

Alors que les spirales anti-moustiques à transfluthrine doivent être brulées et produisent de la fumée, avec ce nouveau dispositif plus simple, le produit s'évapore naturellement et se propage dans l'air à température ambiante, de sorte qu'il protège un espace extérieur avec une vapeur répulsive qui sera invisible pour vous. Le dispositif lui-même n'est constitué que d'une bande de toile de jute (sac), un matériau utilisée pour fabriquer des sacs pour le stockage et le transport de céréales comme le maïs et le riz, dans lequel est imprégné un insecticide largement utilisé, la transfluthrine. L'insecticide utilisé pour traiter ces bandes de tissu coûte moins de 15 gourdes par bande, et les bandes de tissu peuvent être traitées en toute sécurité en utilisant uniquement des gants en plastique comme protection. Des décennies d'utilisation de la transfluthrine partout dans le monde montrent un bilan très positif pour la sécurité des utilisateurs. La transfluthrine est couramment utilisée dans des dispositifs diffusant un répulsif comme les spirales anti-moustiques, et est enregistrée pour être utilisée comme répulsif dans toute l'Europe, l'Amérique, l'Asie et l'Afrique depuis plusieurs décennies et est approuvée pour l'évaluation expérimentale en Tanzanie.

Dans cette étude, Plusieurs expérimentations vont mesurer quelle est la protection apportée par différentes formes de l'émanateur contre les piqûres de moustiques. En tant que participant, vous attraperez les moustiques avec un piège à grille électrique, composé d'un cadre carré en plastique contenant des fils électrifiés. Le cadre du piège est doublé avec un filet en plastique, pour protéger

vos jambes contre le contact avec les fils électrifiés extérieurs, qui transportent suffisamment d'électricité pour tuer les moustiques, mais pas assez pour nuire à un être humain. Chaque jour d'expérimentation, vous utiliserez ce piège pour une période de 3 heures en début de matinée et une autre période de 3 heures le soir. Lors de ces deux séances de capture quotidiennes, vous devrez vous asseoir sur une chaise avec les jambes placées à l'intérieur du cadre carré, tandis que le reste de votre corps, à l'exception de votre visage, sera recouvert de vêtements de protection pour prévenir les piqûres de moustiques. Ces vêtements de protection seront fournis gratuitement par le projet.

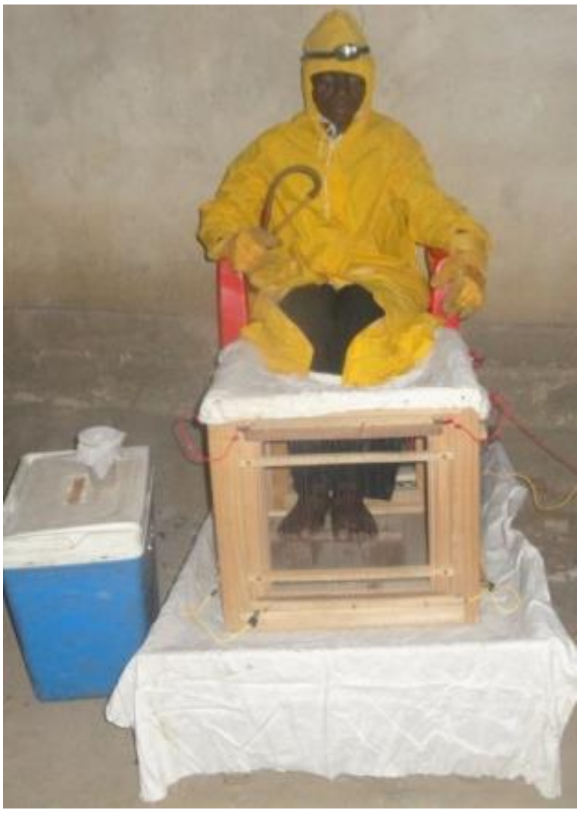

Vous vous assoirez avec vos pieds dans le cadre en plastique du dispositif de piégeage de moustiques électrique pendant 45 minutes pendant chaque période d'une heure. À la fin de chaque séance de 45 minutes assis avec vos pieds dans cet appareil, vous prendrez une pause de 15 minutes pour vous reposer, vous détendre et profiter des rafraîchissements qui vous seront fournis gratuitement. Vous répéterez ce cycle de 45 minutes et ensuite 15 minutes de repos deux fois pendant les quarts de 3 heures de travail le matin et le soir de chaque jour de participation. Vous devez participer pendant huit jours répartis sur deux semaines de travail, ce qui aura lieu une fois tous les deux mois pendant une période de six mois ou jusqu'à ce que vous décidez de vous retirer de l'étude. Bien qu'il soit peu probable que vous soyez piqué par les moustiques assis avec vos jambes à l'intérieur du dispositif de piégeage, il est possible que vous soyez piqué pendant vos pauses de 15 minutes, comme vous le seriez à l'extérieur de votre maison. Nous vous encourageons donc à protéger vos jambes pendant ces pauses en déroulant votre pantalon et en portant des chaussures et des chaussettes, mais nous vous demandons de ne pas appliquer de répulsifs qui vont interférer avec l'étude.

Ces activités seront toutes menées dans l'espace extérieur ou le jardin près de votre maison, que nous avons visité avec vous pour discuter de l'étude.

Certains jours, on vous fournira un cadre contenant une bande de toile qui peut être traitée avec la transfluthrine, tandis que d'autres jours, la bande ne contiendra aucun insecticide. Le cadre contenant la bande de toile (traitée ou non) sera placé appuyé contre votre chaise, immédiatement derrière vous. Si vous participez à cette étude, vous n'aurez pas à manipuler la transfluthrine, ni même les tissus traités à la transfluthrine: Toutes les procédures de traitement et les manipulations de ces matériaux seront effectuées par les investigateurs. Votre seule exposition à la transfluthine sera donc la vapeur émanant des bandes traitées présentes à proximité, qui devraient dissuader les

moustiques en s'évaporant lentement dans l'air à des concentrations plus de 1000 fois inférieures à la limite de sécurité approuvée pour l'utilisation de ce produit, qui est utilisé dans le monde entier dans les spirales et autres dispositifs anti-moustiques. Les concentrations de Transfluthrine émises par ce dispositif sont tellement faibles qu'on n'anticipe aucun risque pour la santé.

Votre participation est volontaire et vous êtes libre de quitter l'étude à n'importe quel moment si vous le souhaitez. Les seuls risques de participation à cette étude sont la possibilité d'être exposé à un choc électrique léger si vous touchez accidentellement la surface extérieure de la grille d'électrique avant de l'éteindre. Cela peut être inconfortable, mais est totalement inoffensif à la tension utilisée. Si jamais vous cela vous pose problème, vous êtes libre de quitter l'étude à tout moment.

Vous recevrez une indemnité de 15 US$ par jour de participation, pour vous indemniser pour les repas, les inconvénients et l'inconfort. Le seul autre avantage que vous recevrez est que, nous espérons que les résultats de cette étude permettront de contrôler plus efficacement les moustiques et les virus de la dengue, chikungunya et zika, permettant d'améliorer les conditions de vie pour vous, votre famille et le pays en général, tout en économisant de l'argent qui serait autrement consacré à des soins médicaux. En effet, les résultats de cette étude devraient avoir des implications très directes et significatives pour les décideurs politiques en Haïti, ainsi que les décideurs des organismes donateurs.

Le scientifique responsable de cette étude est le Pr Christian Raccurt, Doyen de la Faculté des Sciences Médicales de l'Université Quisqueya. Cette étude a été approuvée par le Comité National de Bioéthique du Ministère de la Santé publique et de la Population. Si vous avez des questions au sujet de cette étude, veuillez contacter Pr Raccurt au 3764 2584.
Pour plus d'information vous pourriez souhaiter contacter le Dr Cyrille Czeher au 3726 5230. Si vous n'êtes pas satisfait par les explications fournies, vous pouvez faire part de vos réserves auprès du Dr Gerald Lerebours, président du Comité National de Bioéthique, c/o AMH, 29, Avenue de la Ligue Féminine ci-devant 1 Avenue du Travail, Port-au-Prince; Téléphone 3701 5766.

Emanateurs de transfluthrine simples, abordables et évolutifs pour protéger contre la transmission des virus Zika, Dengue et Chikungunya

ANNEXE 2
ACCORD DE CONSENTEMENT ÉCLAIRÉ POUR LES ADULTES PARTICIPANT AUX CAPTURES DE MOUSTIQUES

NUMÉRO DE SÉRIE DE L'ACCORD DE CONSENTEMENT ÉCLAIRÉ:____________________

Les moustiques *Aedes aegypti* sont noirs avec des marques blanches et piquent habituellement pendant les heures du jour. Ici, en Haïti, ils transmettent les virus de la dengue et du chikungunya, qui sont des causes courantes de fièvre, de douleurs corporelles et d'un certain nombre d'autres symptômes. En plus, ce moustique transmet également le virus Zika, qui cause de graves malformations chez les enfants de femmes enceintes qui ont été infectées.

Nous vous invitons à participer à une étude pour évaluer un nouvel outil pour prévenir les piqures de ces moustiques. L'objectif de l'étude est de démontrer qu'une nouvelle formulation de transfluthrine, le même insecticide sûr et couramment utilisé dans les spirales anti-moustiques, peut fournir une protection abordable contre ces moustiques pendant au moins 6 mois. Notez que, dans notre nouvelle approche, les insecticides s'évaporent naturellement et se propagent dans l'air sous forme de vapeur, tandis que les spirales anti-moustiques doivent être brulées et produisent toujours de la fumée.

En tant que participant, vous capturerez des moustiques avec un piège à grille électrique, composé d'un cadre carré en plastique contenant des fils électrifiés. Le cadre du piège est doublé avec un grillage en plastique pour protéger vos membres du contact avec les fils extérieurs, qui ont suffisamment d'électricité pour tuer les moustiques, mais pas assez pour blesser un être humain. Chaque jour de travail, vous utiliserez ce piège pour une période de 3 heures le matin et une autre période de 3 heures le soir. Pendant ces deux séances quotidiennes, vous devrez vous asseoir sur une chaise avec les jambes placées à l'intérieur du cadre carré, tandis que le reste de votre corps, à l'exception de votre visage, sera recouvert de vêtements de protection pour prévenir les piqûres de moustiques. Ces vêtements de protection seront fournis gratuitement par le projet. Vous serez assis à proximité d'une bande de tissu qui pourra être traitée avec un insecticide largement utilisé et très sûr appelé transfluthrine. Le traitement à la transfluthine repoussera les moustiques en s'évaporant lentement dans l'air à des concentrations plus de 1000 fois inférieures à la limite de sécurité approuvée pour cet insecticide, qui est utilisé dans le monde entier dans des spirales et autres dispositifs anti-moustiques.

Votre participation est volontaire et vous êtes libre de quitter l'étude à n'importe quel moment si vous le souhaitez. Les seuls risques de participation à cette étude sont la possibilité d'être exposé à un choc électrique léger si vous touchez accidentellement la surface extérieure de la grille

d'électrique avant de l'éteindre. Cela peut être inconfortable, mais est totalement inoffensif à la tension utilisée. Si jamais vous cela vous pose problème, vous êtes libre de quitter l'étude à tout moment. Vous recevrez une indemnité de 15 US$ par jour de participation, pour vous indemniser pour les repas, les inconvénients et l'inconfort. Le seul autre avantage que vous recevrez est que, nous espérons que les résultats de cette étude permettront de contrôler plus efficacement les moustiques et les virus de la dengue, chikungunya et zika, permettant d'améliorer les conditions de vie pour vous, votre famille et le pays en général, tout en économisant de l'argent qui serait autrement consacré à des soins médicaux.

Le scientifique responsable de cette étude est le Pr Christian Raccurt, Doyen de la Faculté des Sciences Médicales de l'Université Quisqueya. Cette étude a été approuvée par le Comité National de Bioéthique du Ministère de la Santé publique et de la Population. Si vous avez des questions au sujet de cette étude, veuillez contacter Pr Raccurt au 3764 2584.
Pour plus d'information vous pourriez souhaiter contacter le Dr Cyrille Czeher au 3726 5230. Si vous n'êtes pas satisfait par les explications fournies, vous pouvez faire part de vos réserves auprès du Dr Gerald Lerebours, président du Comité National de Bioéthique, c/o AMH, 29, Avenue de la Ligue Féminine ci-devant 1 Avenue du Travail, Port-au-Prince; Téléphone 3701 5766.

Accord de Consentement Eclairé pour chaque participant à l'étude :

Je, soussigné …………………………………………… Comprend clairement les objectifs du projet intitulé **«ÉMANATEURS DE TRANSFLUTHRINE SIMPLES POUR PROTÉGER CONTRE LA TRANSMISSION DES VIRUS ZIKA, DENGUE ET CHIKUNGUNYA»** et je suis d'accord pour participer à l'étude. Je comprends également que l'utilisation du piège à grille électrique peut m'exposer à un risque de choc électrique avec un courant faible qui est inconfortable mais inoffensif. Je comprends que je peux révoquer mon consentement et quitter l'étude à tout moment.

Nom du participant à l'étude: ____

Sexe (Homme/Femme: _________________________ Âge:___________________

Signature du participant à l'étude: _______________________Date_________________

Nom du Témoin: ____

Signature du Témoin: ____________________________________Date______________

Emanateurs de transfluthrine simples, abordables et évolutifs pour protéger contre la transmission des virus Zika, Dengue et Chikungunya

ANNEXE 3
ACCORD DE CONSENTEMENT ÉCLAIRÉ DE PARTICIPATION POUR PROPRIÉTAIRE OU PERSONNE RESPONSABLE DE LA GESTION D'UN TERRAIN POUR PERMETTRE L'EVALUATION DES ANTI-MOUSTIQUES

NUMÉRO DE SÉRIE DE L'ACCORD DE CONSENTEMENT ÉCLAIRÉ:____________________

Les moustiques *Aedes aegypti* sont noirs avec des marques blanches et piquent habituellement pendant les heures du jour. Ici, en Haïti, ils transmettent les virus de la dengue et du chikungunya, qui sont des causes courantes de fièvre, de douleurs corporelles et d'un certain nombre d'autres symptômes. En plus, ce moustique transmet également le virus Zika, qui cause de graves malformations chez les enfants de femmes enceintes qui ont été infectées.

Nous aimerions avoir votre permission pour que vous et votre foyer puissiez participer à une étude pour évaluer un nouvel outil pour prévenir les piqures de ces moustiques. L'objectif de l'étude est de démontrer qu'une nouvelle formulation de transfluthrine, le même insecticide sûr et couramment utilisé dans des spirales anti-moustiques, peut fournir une protection abordable contre ces moustiques pendant au moins 6 mois.

Plus précisément, nous voulons capturer des moustiques et demandons votre autorisation pour que deux membres de notre équipe de recherche puissent attraper les moustiques qui essaient de les piquer sur votre terrain aujourd'hui, pour une période déterminée de 3 heures le matin et une autre période déterminée de 3 heures le soir. Il/elle va s'assoir sur une chaise avec ses jambes placées dans un piège à moustiques. Les pièges sont composés d'un cadre carré électrisé, tandis que le reste du corps, à l'exception du visage, sera recouvert de vêtements de protection pour prévenir les piqûres de moustiques. Ils ne devraient déranger personne, et ne rien demander à vous, votre foyer ou vos voisins. À certaines occasions, les participants recevront une bande de tissu qui peut être traitée avec un insecticide largement utilisé et très sûr appelé transfluthrine. Le traitement à la transfluthine éloignera les moustiques en s'évaporant lentement dans l'air à des concentrations plus de 1000 fois inférieures à la limite de sécurité approuvée pour cet insecticide, qui est utilisé dans le monde entier dans des spirales et autres dispositifs anti-moustiques.

Votre participation est volontaire et vous êtes libre de quitter l'étude à n'importe quel moment si vous le souhaitez. Il n'y a pas de risque pour vous, votre famille ou le voisinage si vous participez à l'étude. Le seul autre avantage que vous recevrez est que, nous espérons que les résultats de cette étude permettront de contrôler plus efficacement les moustiques et les virus de la dengue, chikungunya et zika, permettant d'améliorer les conditions de vie pour vous, votre famille et le

pays en général, tout en économisant de l'argent qui serait autrement consacré à des soins médicaux.

Le scientifique responsable de cette étude est le Pr Christian Raccurt, Doyen de la Faculté des Sciences Médicales de l'Université Quisqueya. Cette étude a été approuvée par le Comité National de Bioéthique du Ministère de la Santé publique et de la Population. Si vous avez des questions au sujet de cette étude, veuillez contacter Pr Raccurt au 3764 2584.
Pour plus d'information vous pourriez souhaiter contacter le Dr Cyrille Czeher au 3726 5230. Si vous n'êtes pas satisfait par les explications fournies, vous pouvez faire part de vos réserves auprès du Dr Gerald Lerebours, président du Comité National de Bioéthique, c/o AMH, 29, Avenue de la Ligue Féminine ci-devant 1 Avenue du Travail, Port-au-Prince; Téléphone 3701 5766.

Accord de Consentement Eclairé pour chaque participant à l'étude :

Je, soussigné ………………………………………………, comprend clairement les objectifs du projet intitulé **«ÉMANATEURS DE TRANSFLUTHRINE SIMPLES POUR PROTÉGER CONTRE LA TRANSMISSION DES VIRUS ZIKA, DENGUE ET CHIKUNGUNYA»** et j'autorise que mon terrain/parcelle soit utilisé(e) pour collecter des moustiques pour l'étude. Je comprends que deux membres de l'équipe de recherche attraperont des moustiques en plein air aujourd'hui sur ce terrain/parcelle. Je m'attends à ce qu'ils ne dérangent personne sur ce terrain/parcelle, et qu'ils ne demandent rien de moi, mon foyer ou mes voisins. Je comprends que la participation est volontaire et que je peux révoquer mon consentement et quitter l'étude à tout moment.

Nom du participant à l'étude: ____

Signature du participant à l'étude: ________________________Date__________________

Nom du Témoin: ____

Signature du Témoin: ____________________________________Date_______________

Emanateurs de transfluthrine simples, abordables et évolutifs pour protéger contre la transmission des virus Zika, Dengue et Chikungunya

ANNEXE 4

FICHE D'INFORMATION DES PARTICIPANTS POUR L'UTILISATION RÉGULIERE DES ÉMANATEURS DE TRANSFLUTHRINE DANS LES FOYERS

Les moustiques noirs avec des marques blanches qui piquent habituellement pendant les heures du jour transmettent les virus de la dengue et du chikungunya, qui sont des causes courantes de fièvre, de douleurs corporelles et d'un certain nombre d'autres symptômes ici en Haïti. En plus, ce moustique transmet également le virus Zika, qui cause de graves malformations chez les enfants de femmes enceintes qui ont été infectées. Parce qu'ils piquent à l'extérieur pendant la journée quand les gens sont généralement actifs, ni les moustiquaires ni les logements protégés des moustiques ne peuvent fournir une protection satisfaisante contre ces maladies. Malheureusement, les spirales anti-moustiques (placatox) et les répulsifs pour la peau protègent seulement quelques heures après application, ce qui les rend trop chers et peu pratiques pour une utilisation continue.

L'équipe de recherche responsable de cette étude a récemment développé un émanateur de transfluthrine simple, qui fournit plus de 90% de protection pendant plus de 4 mois contre les moustiques qui piquent la nuit en Afrique. Nous vous invitons donc à participer à une étude pour évaluer ce nouvel outil de prévention des piqûres de ces moustiques piquant la journée ici en Haïti. Le but de l'étude est de démontrer que cette nouvelle reformulation de la transfluthrine peut fournir une protection abordable contre ces moustiques importants pendant au moins 6 mois.

Alors que les spirales anti-moustiques à transfluthrine doivent être brulées et produisent de la fumée, avec ce nouveau dispositif plus simple, le produit s'évapore naturellement et se propage dans l'air à température ambiante, de sorte qu'il protège un espace extérieur avec une vapeur répulsive qui sera invisible pour vous. Le dispositif lui-même n'est constitué que d'une bande de toile de jute (sac), un matériau utilisé pour fabriquer des sacs pour le stockage et le transport de céréales comme le maïs et le riz, dans lequel est imprégné un insecticide largement utilisé, la transfluthrine. L'insecticide utilisé pour traiter ces bandes de tissu coûte moins de 15 gourdes par bande, et les bandes de tissu peuvent être traitées en toute sécurité en utilisant uniquement des gants en plastique comme protection. Des décennies d'utilisation de la transfluthrine partout dans le monde montrent un bilan très positif pour la sécurité des utilisateurs. La transfluthrine est couramment utilisée dans des dispositifs diffusant un répulsif comme les spirales anti-moustiques, et est enregistrée pour être utilisée comme répulsif dans toute l'Europe, l'Amérique, l'Asie et l'Afrique depuis plusieurs décennies et est approuvée pour l'évaluation expérimentale en Tanzanie.

Une des expérimentations prévues dans cette étude implique de donner deux de ces émanateurs à un petit nombre de foyers consentants, pour qu'ils les utilisent pour se protéger des moustiques pour une durée de 6 mois. Si vous et votre foyer acceptez de participer à cette partie de l'étude, l'équipe de recherche visitera votre maison une fois tous les deux mois et récupérera les émanateurs pour environ deux semaines dans le but de mesurer le degré de protection qu'ils fournissent contre les piqûres de moustiques à ce moment. Après avoir discuté avec l'équipe de recherche sur les modes d'utilisation sûrs et efficaces, vous serez libre d'utiliser ces émanateurs de la manière que

vous jugez la plus pratique et efficace, tant que vous n'ouvrez pas le support de protection ou ne l'utilisez d'aucune façon qui pourrait entrainer un contact physique direct avec le matériau imprégné.

Aussi, il sera demandé à un membre de votre foyer d'enregistrer ce que le foyer perçoit comme étant les façons les moins et les plus efficaces d'utiliser le dispositif, à l'aide d'un appareil photo jetable qui sera fourni par l'équipe de recherche. Avant d'utiliser l'appareil photo, la personne identifiée comme responsable de la prise des photos participera à une courte réunion de formation au cours de laquelle seront expliqué comment utiliser l'appareil photo de façon acceptable, sans compromettre la sécurité, la vie privée et les autres droits des habitants du foyer ou voisins. De plus, quand chaque foyer sera visité une fois tous les deux mois, pour récupérer et rapporter les émanateurs pour servir aux tests d'efficacité, il vous sera demandé de répondre à un bref questionnaire semi-structuré dans le but d'évaluer votre niveau de satisfaction par rapport à la protection contre les piqûres de moustiques. Au début de chaque visite du foyer, tout appareil photo totalement utilisé sera collecté par l'équipe de recherche pour permettre le développement des photos en deux exemplaires. Un exemplaire vous sera remis après quelques jours, l'autre sera conservé confidentiellement par l'équipe de recherche comme double en cas de perte.

A la fin de l'étude, il sera demandé à une femme adulte consentante de votre foyer de participer à une discussion de groupe au sujet des émanateurs, avec jusqu'à 8 autres femmes participantes faisant partie d'autres foyers qui ont également utilisé les émanateurs. Similairement, un homme adulte consentant de chaque foyer participera à une discussion de groupe au sujet de l'émanateur avec un groupe d'hommes de la même taille. Lors de chaque discussion de groupe, un facilitateur travaillant avec l'équipe de recherche va guider la discussion tandis qu'un observateur fera un enregistrement audio et prendra des notes sur ce qui a été dit. De même, il sera demandé au membre du foyer responsable de prendre les photographies de sélectionner les meilleures et les plus informatives, puis de discuter de leur signification au sein de petits groupes avec les photographes d'autres foyers. La participation de votre foyer et chacun de ses membres est entièrement volontaire, et vous êtes libres de quitter l'étude à tout moment si vous le décidez. Si l'équipe de recherche souhaite publier toute photographie prise par les membres du foyer montrant la maison, son contenu ou ses occupants, le chef de famille sera d'abord consulté et il lui sera demandé de donner son consentement écrit, ce qu'ils pourraient refuser librement.

Vous ne recevrez pas de compensation pour votre participation à cette étude. Le seul bénéfice direct que vous recevrez sera la protection contre les piqûres de moustiques. Vous devriez aussi bénéficier indirectement de l'étude, parce que ses résultats permettront de contrôler plus efficacement les moustiques et les virus de la dengue, chikungunya et zika, permettant d'améliorer les conditions de vie pour vous, votre famille et le pays en général, tout en économisant de l'argent qui serait autrement consacré à des soins médicaux. En effet, les résultats de cette étude devraient avoir des implications très directes et significatives pour les décideurs politiques en Haïti, ainsi que les décideurs des organismes donateurs.

Le scientifique responsable de cette étude est le Pr Christian Raccurt, Doyen de la Faculté des Sciences Médicales de l'Université Quisqueya. Cette étude a été approuvée par le Comité National de Bioéthique du Ministère de la Santé publique et de la Population. Si vous avez des questions au sujet de cette étude, veuillez contacter Pr Raccurt au 3764 2584.
Pour plus d'information vous pourriez souhaiter contacter le Dr Cyrille Czeher au 3726 5230. Si vous n'êtes pas satisfait par les explications fournies, vous pouvez faire part de vos réserves auprès du Dr Gerald Lerebours, président du Comité National de Bioéthique, c/o AMH, 29, Avenue de la Ligue Féminine ci-devant 1 Avenue du Travail, Port-au-Prince; Téléphone 3701 5766.

Emanateurs de transfluthrine simples, abordables et évolutifs pour protéger contre la transmission des virus Zika, Dengue et Chikungunya

ANNEXE 5
ACCORD DE CONSENTEMENT ÉCLAIRÉ POUR LES CHEFS DE FOYERS QUI UTILISENT RÉGULIEREMENT LES ÉMANATEURS DE TRANSFLUTHRINE

NUMÉRO DE SÉRIE DE L'ACCORD DE CONSENTEMENT ÉCLAIRÉ:___________________

Les moustiques *Aedes aegypti* sont noirs avec des marques blanches et piquent habituellement pendant les heures du jour. Ici, en Haïti, ils transmettent les virus de la dengue et du chikungunya, qui sont des causes courantes de fièvre, de douleurs corporelles et d'un certain nombre d'autres symptômes. En plus, ce moustique transmet également le virus Zika, qui cause de graves malformations chez les enfants de femmes enceintes qui ont été infectées.

Nous vous invitons à participer à une étude pour évaluer un nouvel outil pour prévenir les piqures de ces moustiques. L'objectif de l'étude est de démontrer qu'une nouvelle formulation de transfluthrine, le même insecticide sûr et couramment utilisé dans les spirales anti-moustiques, peut fournir une protection abordable contre ces moustiques pendant au moins 6 mois. Notez que, dans notre nouvelle approche, les insecticides s'évaporent naturellement et se propagent dans l'air sous forme de vapeur, tandis que les spirales anti-moustiques doivent être brulées et produisent toujours de la fumée.

Une des expérimentations prévues dans cette étude implique de donner deux de ces émanateurs à un petit nombre de foyers consentants, pour qu'ils les utilisent pour se protéger des moustiques pour une durée de 6 mois. Si vous et votre foyer acceptez de participer à cette partie de l'étude, l'équipe de recherche visitera votre maison une fois tous les deux mois et récupérera les émanateurs pour environ deux semaines dans le but de mesurer le degré de protection qu'ils fournissent contre les piqûres de moustiques à ce moment. Après avoir discuté avec l'équipe de recherche sur les modes d'utilisation sûrs et efficaces, vous serez libre d'utiliser ces émanateurs de la manière que vous jugez la plus pratique et efficace, tant que vous n'ouvrez pas le support de protection ou ne l'utilisez d'aucune façon qui pourrait entrainer un contact physique direct avec le matériau imprégné.

Aussi, il sera demandé à un membre de votre foyer d'enregistrer ce que le foyer perçoit comme étant les façons les moins et les plus efficaces d'utiliser le dispositif, à l'aide d'un appareil photo jetable qui sera fourni par l'équipe de recherche. Avant d'utiliser l'appareil photo, la personne identifiée comme responsable de la prise des photos participera à une courte réunion de formation au cours de laquelle seront expliqué comment utiliser l'appareil photo de façon acceptable, sans compromettre la sécurité, la vie privée et les autres droits des habitants du foyer ou voisins. De plus, quand chaque foyer sera visité une fois tous les deux mois, pour récupérer et rapporter les émanateurs pour servir aux tests

d'efficacité, il vous sera demandé de répondre à un bref questionnaire semi-structuré dans le but d'évaluer votre niveau de satisfaction par rapport à la protection contre les piqûres de moustiques. Au début de chaque visite du foyer, tout appareil photo totalement utilisé sera collecté par l'équipe de recherche pour permettre le développement des photos en deux exemplaires. Un exemplaire vous sera remis après quelques jours, l'autre sera conservé confidentiellement par l'équipe de recherche comme double en cas de perte.

A la fin de l'étude, il sera demandé à une femme adulte consentante de votre foyer de participer à une discussion de groupe au sujet des émanateurs, avec jusqu'à 8 autres femmes participantes faisant partie d'autres foyers qui ont également utilisé les émanateurs. Similairement, un homme adulte consentant de chaque foyer participera à une discussion de groupe au sujet de l'émanateur avec un groupe d'hommes de la même taille. Lors de chaque discussion de groupe, un facilitateur travaillant avec l'équipe de recherche va guider la discussion tandis qu'un observateur fera un enregistrement audio et prendra des notes sur ce qui a été dit. De même, il sera demandé au membre du foyer responsable de prendre les photographies de sélectionner les meilleures et les plus informatives, puis de discuter de leur signification au sein de petits groupes avec les photographes d'autres foyers. La participation de votre foyer et chacun de ses membres est entièrement volontaire, et vous êtes libres de quitter l'étude à tout moment si vous le décidez. Si l'équipe de recherche souhaite publier toute photographie prise par les membres du foyer montrant la maison, son contenu ou ses occupants, le chef de famille sera d'abord consulté et il lui sera demandé de donner son consentement écrit, ce qu'ils pourraient refuser librement.

Vous ne recevrez pas de compensation pour votre participation à cette étude. Le seul bénéfice direct que vous recevrez sera la protection contre les piqûres de moustiques. Vous devriez aussi bénéficier indirectement de l'étude, parce que ses résultats permettront de contrôler plus efficacement les moustiques et les virus de la dengue, chikungunya et zika, permettant d'améliorer les conditions de vie pour vous, votre famille et le pays en général, tout en économisant de l'argent qui serait autrement consacré à des soins médicaux. En effet, les résultats de cette étude devraient avoir des implications très directes et significatives pour les décideurs politiques en Haïti, ainsi que les décideurs des organismes donateurs.

Le scientifique responsable de cette étude est le Pr Christian Raccurt, Doyen de la Faculté des Sciences Médicales de l'Université Quisqueya. Cette étude a été approuvée par le Comité National de Bioéthique du Ministère de la Santé publique et de la Population. Si vous avez des questions au sujet de cette étude, veuillez contacter Pr Raccurt au 3764 2584.
Pour plus d'information vous pourriez souhaiter contacter le Dr Cyrille Czeher au 3726 5230. Si vous n'êtes pas satisfait par les explications fournies, vous pouvez faire part de vos réserves auprès du Dr Gerald Lerebours, président du Comité National de Bioéthique, c/o AMH, 29, Avenue de la Ligue Féminine ci-devant 1 Avenue du Travail, Port-au-Prince; Téléphone 3701 5766.

Accord de Consentement Eclairé pour chaque participant à l'étude :

En tant que chef de mon foyer, je soussigné ……………………………………………. comprend clairement les objectifs du projet intitulé **«ÉMANATEURS DE TRANSFLUTHRINE SIMPLES POUR PROTÉGER CONTRE LA TRANSMISSION DES VIRUS ZIKA, DENGUE ET CHIKUNGUNYA».** Je suis d'accord pour que mon foyer participe à l'étude en utilisant les émanateurs répulsifs fournis par l'équipe de recherche. J'accepte aussi que les membres de mon foyer participent à des discussions enregistrées au sujet de ces dispositifs, et qu'un des membres du foyer prenne des photographies qui illustrent comment ces émanateurs sont utilisés. Je comprends également que l'équipe de recherche doit me consulter pour obtenir mon consentement écrit avant de publier toute photographie prise dans mon foyer, ou de les partager avec quiconque d'extérieur au foyer et à l'équipe de recherche. Je comprends que je peux révoquer mon consentement et quitter l'étude à tout moment.

Nom du participant à l'étude: ____

Sexe (Homme/Femme: ________________________ Âge:____________________

Signature du participant à l'étude: _______________________Date_________________

Nom du Témoin: ____

Signature du Témoin: ___________________________________Date_______________

Emanateurs de transfluthrine simples, abordables et évolutifs pour protéger contre la transmission des virus Zika, Dengue et Chikungunya

ANNEXE 6
ACCORD DE CONSENTEMENT ÉCLAIRÉ POUR LES REPONDANTS AU QUESTIONNAIRE DANS LES FOYERS QUI UTILISENT LES EMANATEURS DE TRANSFLUTHRINE

NUMÉRO DE SÉRIE DE L'ACCORD DE CONSENTEMENT ÉCLAIRÉ:___________________

Les moustiques *Aedes aegypti* sont noirs avec des marques blanches et piquent habituellement pendant les heures du jour. Ici, en Haïti, ils transmettent les virus de la dengue et du chikungunya, qui sont des causes courantes de fièvre, de douleurs corporelles et d'un certain nombre d'autres symptômes. En plus, ce moustique transmet également le virus Zika, qui cause de graves malformations chez les enfants de femmes enceintes qui ont été infectées.

Nous vous invitons à participer à une étude pour évaluer un nouvel outil pour prévenir les piqures de ces moustiques. L'objectif de l'étude est de démontrer qu'une nouvelle formulation de transfluthrine, le même insecticide sûr et couramment utilisé dans les spirales anti-moustiques, peut fournir une protection abordable contre ces moustiques pendant au moins 6 mois. Notez que, dans notre nouvelle approche, les insecticides s'évaporent naturellement et se propagent dans l'air sous forme de vapeur, tandis que les spirales anti-moustiques doivent être brulées et produisent toujours de la fumée.

Une des expérimentations prévues dans cette étude implique de donner deux de ces émanateurs à un petit nombre de foyers consentants, pour qu'ils les utilisent pour se protéger des moustiques pour une durée de 6 mois. Si vous et votre foyer acceptez de participer à cette partie de l'étude, l'équipe de recherche visitera votre maison une fois tous les deux mois et récupérera les émanateurs pour environ deux semaines dans le but de mesurer le degré de protection qu'ils fournissent contre les piqûres de moustiques à ce moment. Après avoir discuté avec l'équipe de recherche sur les modes d'utilisation sûrs et efficaces, vous serez libre d'utiliser ces émanateurs de la manière que vous jugez la plus pratique et efficace, tant que vous n'ouvrez pas le support de protection ou ne l'utilisez d'aucune façon qui pourrait entrainer un contact physique direct avec le matériau imprégné. De plus, en tant que membre de votre foyer, il vous sera demandé de répondre à un court questionnaire semi-structuré pour évaluer votre niveau de satisfaction avec la protection apportée contre les piqures de moustiques. Aucune information personnelle ne sera enregistrée en dehors de vos noms, genre et âge, ces détails seront conservés de manière confidentielle. Votre participation et celle de votre foyer est entièrement volontaire et vous êtes libre de quitter l'étude à tout moment.

Vous ne recevrez pas de compensation pour votre participation à cette étude. Le seul bénéfice direct que vous recevrez sera la protection contre les piqûres de moustiques. Vous devriez aussi bénéficier indirectement de l'étude, parce que ses résultats permettront de contrôler plus

efficacement les moustiques et les virus de la dengue, chikungunya et zika, permettant d'améliorer les conditions de vie pour vous, votre famille et le pays en général, tout en économisant de l'argent qui serait autrement consacré à des soins médicaux. En effet, les résultats de cette étude devraient avoir des implications très directes et significatives pour les décideurs politiques en Haïti, ainsi que les décideurs des organismes donateurs.

Le scientifique responsable de cette étude est le Pr Christian Raccurt, Doyen de la Faculté des Sciences Médicales de l'Université Quisqueya. Cette étude a été approuvée par le Comité National de Bioéthique du Ministère de la Santé publique et de la Population. Si vous avez des questions au sujet de cette étude, veuillez contacter Pr Raccurt au 3764 2584.
Pour plus d'information vous pourriez souhaiter contacter le Dr Cyrille Czeher au 3726 5230. Si vous n'êtes pas satisfait par les explications fournies, vous pouvez faire part de vos réserves auprès du Dr Gerald Lerebours, président du Comité National de Bioéthique, c/o AMH, 29, Avenue de la Ligue Féminine ci-devant 1 Avenue du Travail, Port-au-Prince; Téléphone 3701 5766.

Accord de Consentement Eclairé pour chaque participant à l'étude :

En tant que membre de mon foyer, je soussigné …………………………………………….. comprend clairement les objectifs du projet intitulé **«ÉMANATEURS DE TRANSFLUTHRINE SIMPLES POUR PROTÉGER CONTRE LA TRANSMISSION DES VIRUS ZIKA, DENGUE ET CHIKUNGUNYA».** Je suis d'accord pour que mon foyer participe à l'étude en utilisant les émanateurs répulsifs fournis par l'équipe de recherche. J'accepte aussi de participer à une enquête utilisant un court questionnaire de satisfaction par rapport à ces dispositifs. Je comprends que je peux révoquer mon consentement et quitter l'étude à tout moment.

Nom du participant à l'étude: ____

Sexe (Homme/Femme): ________________________ Âge:____________________

Signature du participant à l'étude: ____________________ Date_________________

Nom du Témoin: ____

Signature du Témoin: __________________________________Date______________

Emanateurs de transfluthrine simples, abordables et évolutifs pour protéger contre la transmission des virus Zika, Dengue et Chikungunya

ANNEXE 7
QUESTIONNAIRE POUR L'ENQUETE DE SATISFACTION DES FOYERS AU SUJET DES ÉMANATEURS DE TRANSFLUTHRINE

Numéro de Maison:___ Nom du participant:________________ Genre (H/F):___ Date:_________

Numéro de série de l'accord de consentement éclairé (ICF1) du participant__________________

1. Dans quelle mesure les émanateurs répulsifs vous ont-ils protégé des moustiques quand vous êtes dans la maison? Entourez une des réponses ci-dessous, ou entourez la mention suivante : **PAS DE RÉPONSE DONNÉE**.

**1**<br>**PAS DU TOUT**	**2**<br>**MODÉRÉMENT**	**3**<br>**BIEN**	**4**<br>**TRES BIEN**	**5**<br>**TOTALEMENT**

2. Dans quelle mesure les émanateurs répulsifs vous protègent-ils ainsi que votre famille contre d'autres insectes nuisibles quand vous êtes dans la maison ? Entourez une des réponses ci-dessous, ou entourez la mention suivante : **PAS DE RÉPONSE DONNÉE**.

**1**<br>**PAS DU TOUT**	**2**<br>**MODÉRÉMENT**	**3**<br>**BIEN**	**4**<br>**TRES BIEN**	**5**<br>**TOTALEMENT**

3. Dans quelle mesure les émanateurs répulsifs vous ont-ils protégé des moustiques quand vous êtes à l'extérieur ? Entourez une des réponses ci-dessous, ou entourez la mention suivante : **PAS DE RÉPONSE DONNÉE**.

**1**<br>**PAS DU TOUT**	**2**<br>**MODÉRÉMENT**	**3**<br>**BIEN**	**4**<br>**TRES BIEN**	**5**<br>**TOTALEMENT**

4. Dans quelle mesure les émanateurs répulsifs vous protègent-ils ainsi que votre famille contre d'autres insectes nuisibles quand vous êtes à l'extérieur ? Entourez une des réponses ci-dessous, ou entourez la mention suivante : **PAS DE RÉPONSE DONNÉE**.

**1**<br>**PAS DU TOUT**	**2**<br>**MODÉRÉMENT**	**3**<br>**BIEN**	**4**<br>**TRES BIEN**	**5**<br>**TOTALEMENT**

5. Nommez et/ou décrivez tout autre avantage ou inconvénient d'avoir ces émanateurs répulsifs:
____
____
____

6. Classer l'importance de cet avantage/inconvénient en entourant one des réponses ci-dessous, ou une des mentions suivantes : **NE S'APPLIQUE PAS** ou **PAS DE RÉPONSE DONNÉE.**

**1**	**2**	**3**	**4**	**5**	**6**
**DÉSAVANTAGE IMPORTANT**	**DÉSAVANTAGE MODÉRÉ**	**DÉSAVANTAGE FAIBLE**	**AVANTAGE FAIBLE**	**AVANTAGE MODÉRÉ**	**AVANTAGE IMPORTANT**

7. Nommez et/ou décrivez tout autre avantage ou inconvénient d'avoir ces émanateurs répulsifs:
____
____
____

8. Classer l'importance de cet avantage/inconvénient en entourant one des réponses ci-dessous, ou une des mentions suivantes : **NE S'APPLIQUE PAS** ou **PAS DE RÉPONSE DONNÉE.**

**1**	**2**	**3**	**4**	**5**	**6**
**DÉSAVANTAGE IMPORTANT**	**DÉSAVANTAGE MODÉRÉ**	**DÉSAVANTAGE FAIBLE**	**AVANTAGE FAIBLE**	**AVANTAGE MODÉRÉ**	**AVANTAGE IMPORTANT**

9. Nommez et/ou décrivez tout autre avantage ou inconvénient d'avoir ces émanateurs répulsifs:
____
____
____

10. Classer l'importance de cet avantage/inconvénient en entourant one des réponses ci-dessous, ou une des mentions suivantes : **NE S'APPLIQUE PAS** ou **PAS DE RÉPONSE DONNÉE.**

**1**	**2**	**3**	**4**	**5**	**6**
**DÉSAVANTAGE IMPORTANT**	**DÉSAVANTAGE MODÉRÉ**	**DÉSAVANTAGE FAIBLE**	**AVANTAGE FAIBLE**	**AVANTAGE MODÉRÉ**	**AVANTAGE IMPORTANT**

Emanateurs de transfluthrine simples, abordables et évolutifs pour protéger contre la transmission des virus Zika, Dengue et Chikungunya

ANNEXE 8

ACCORD DE CONSENTEMENT ECLAIRE POUR LES PARTICIPANTS AUX GROUPES DE DISCUSSION DES FOYERS UTILISANT LES EMANATEURS DE TRANSFLUTHRINE

NUMÉRO DE SÉRIE DE L'ACCORD DE CONSENTEMENT ÉCLAIRÉ:___________________

Les moustiques *Aedes aegypti* sont noirs avec des marques blanches et piquent habituellement pendant les heures du jour. Ici, en Haïti, ils transmettent les virus de la dengue et du chikungunya, qui sont des causes courantes de fièvre, de douleurs corporelles et d'un certain nombre d'autres symptômes. En plus, ce moustique transmet également le virus Zika, qui cause de graves malformations chez les enfants de femmes enceintes qui ont été infectées.

Nous vous invitons à participer à une étude pour évaluer un nouvel outil pour prévenir les piqures de ces moustiques. L'objectif de l'étude est de démontrer qu'une nouvelle formulation de transfluthrine, le même insecticide sûr et couramment utilisé dans les spirales anti-moustiques, peut fournir une protection abordable contre ces moustiques pendant au moins 6 mois. Notez que, dans notre nouvelle approche, les insecticides s'évaporent naturellement et se propagent dans l'air sous forme de vapeur, tandis que les spirales anti-moustiques doivent être brulées et produisent toujours de la fumée.

Une des expérimentations prévues dans cette étude implique de donner deux de ces émanateurs à un petit nombre de foyers consentants, pour qu'ils les utilisent pour se protéger des moustiques pour une durée de 6 mois. Si vous et votre foyer acceptez de participer à cette partie de l'étude, l'équipe de recherche visitera votre maison une fois tous les deux mois et récupérera les émanateurs pour environ deux semaines dans le but de mesurer le degré de protection qu'ils fournissent contre les piqûres de moustiques à ce moment. Après avoir discuté avec l'équipe de recherche sur les modes d'utilisation sûrs et efficaces, vous serez libre d'utiliser ces émanateurs de la manière que vous jugez la plus pratique et efficace, tant que vous n'ouvrez pas le support de protection ou ne l'utilisez d'aucune façon qui pourrait entrainer un contact physique direct avec le matériau imprégné.

Il vous est demandé de représenter votre foyer en participant à une discussion de groupe au sujet du dispositif avec un groupe comptant jusqu'à 8 autres participants du même genre. Les autres participants de la discussion de groupe viendront des autres foyers auxquels des émanateurs ont été fournis. Un facilitateur travaillant avec l'équipe de recherche va guider la discussion tandis qu'un observateur fera un enregistrement audio et prendra des notes

sur ce qui a été dit. Aucune information personnelle ne sera enregistrée hormis votre nom, genre et âge, et ces détails seront gardés confidentiels. Votre participation et celle de votre foyer est entièrement volontaire et vous êtes libre de quitter l'étude à tout moment.

Vous ne recevrez pas de compensation pour votre participation à cette étude. Le seul bénéfice direct que vous recevrez sera la protection contre les piqûres de moustiques. Vous devriez aussi bénéficier indirectement de l'étude, parce que ses résultats permettront de contrôler plus efficacement les moustiques et les virus de la dengue, chikungunya et zika, permettant d'améliorer les conditions de vie pour vous, votre famille et le pays en général, tout en économisant de l'argent qui serait autrement consacré à des soins médicaux. En effet, les résultats de cette étude devraient avoir des implications très directes et significatives pour les décideurs politiques en Haïti, ainsi que les décideurs des organismes donateurs.

Le scientifique responsable de cette étude est le Pr Christian Raccurt, Doyen de la Faculté des Sciences Médicales de l'Université Quisqueya. Cette étude a été approuvée par le Comité National de Bioéthique du Ministère de la Santé publique et de la Population. Si vous avez des questions au sujet de cette étude, veuillez contacter Pr Raccurt au 3764 2584.
Pour plus d'information vous pourriez souhaiter contacter le Dr Cyrille Czeher au 3726 5230. Si vous n'êtes pas satisfait par les explications fournies, vous pouvez faire part de vos réserves auprès du Dr Gerald Lerebours, président du Comité National de Bioéthique, c/o AMH, 29, Avenue de la Ligue Féminine ci-devant 1 Avenue du Travail, Port-au-Prince; Téléphone 3701 5766.

Accord de Consentement Eclairé pour chaque participant à l'étude :

En tant que représentant de mon foyer, je soussigné ……………………………………………. comprends clairement les objectifs du projet intitulé **«ÉMANATEURS DE TRANSFLUTHRINE SIMPLES POUR PROTÉGER CONTRE LA TRANSMISSION DES VIRUS ZIKA, DENGUE ET CHIKUNGUNYA».** Je suis d'accord pour que mon foyer participe à l'étude en utilisant les émanateurs répulsifs fournis par l'équipe de recherche. J'accepte aussi de participer à des discussions enregistrées au sujet de ces dispositifs. Je comprends que je peux révoquer mon consentement et quitter l'étude à tout moment.

Nom du participant à l'étude: ____

Sexe (Homme/Femme: ___________________________ Âge:______________________

Signature du participant à l'étude: _________________________Date__________________

Nom du Témoin: ____

Signature du Témoin: _______________________________________Date________________

Emanateurs de transfluthrine simples, abordables et évolutifs pour protéger contre la transmission des virus Zika, Dengue et Chikungunya

ANNEXE 9
GUIDE THEMATIQUE POUR LES DISCUSSIONS DE GROUPE

1. Perceptions préexistantes sur les moustiques et les moyens de prévention

    1a. Problèmes ou bénéfices associés aux moustiques.

    1b. Mesures habituellement prises pour éviter les piqûres de moustiques ou pour tuer les moustiques.

    1c. Limites des moyens préexistants de protection contre les moustiques.

2. Perception des avantages et inconvénients du dispositif émanateur de répulsif.

    2a. Protection contre les piqûres de moustiques.

    2b. Autres avantages.

    2c. Limites de la protection.

    2d. Autres inconvénients.

    2e. Autres facteurs influençant leur utilisation.

3. Pratiques d'utilisation du dispositif émanateur de répulsif

    3a. Bonnes pratiques d'utilisation.

    3b. Mauvaises pratiques d'utilisation.

    3c. Limites et inconvénients.

    3d. Idées d'amélioration du dispositif en lui-même.

    3e. Idées pour la distribution et l'entretien du dispositif.

<u>Emanateurs de transfluthrine simples, abordables et évolutifs pour protéger contre la transmission des virus Zika, Dengue et Chikungunya</u>

ANNEXE 10

ACCORD DE CONSENTEMENT ECLAIRE POUR LES PARTICIPANTS A L'EXERCICE PHOTOVOICE DANS LES FOYERS UTILISANT LES EMANATEURS DE TRANSFLUTHRINE

NUMÉRO DE SÉRIE DE L'ACCORD DE CONSENTEMENT ÉCLAIRÉ:____________________

Les moustiques *Aedes aegypti* sont noirs avec des marques blanches et piquent habituellement pendant les heures du jour. Ici, en Haïti, ils transmettent les virus de la dengue et du chikungunya, qui sont des causes courantes de fièvre, de douleurs corporelles et d'un certain nombre d'autres symptômes. En plus, ce moustique transmet également le virus Zika, qui cause de graves malformations chez les enfants de femmes enceintes qui ont été infectées.

Nous vous invitons à participer à une étude pour évaluer un nouvel outil pour prévenir les piqures de ces moustiques. L'objectif de l'étude est de démontrer qu'une nouvelle formulation de transfluthrine, le même insecticide sûr et couramment utilisé dans les spirales anti-moustiques, peut fournir une protection abordable contre ces moustiques pendant au moins 6 mois. Notez que, dans notre nouvelle approche, les insecticides s'évaporent naturellement et se propagent dans l'air sous forme de vapeur, tandis que les spirales anti-moustiques doivent être brulées et produisent toujours de la fumée.

Une des expérimentations prévues dans cette étude implique de donner deux de ces émanateurs à un petit nombre de foyers consentants, pour qu'ils les utilisent pour se protéger des moustiques pour une durée de 6 mois. Si vous et votre foyer acceptez de participer à cette partie de l'étude, l'équipe de recherche visitera votre maison une fois tous les deux mois et récupérera les émanateurs pour environ deux semaines dans le but de mesurer le degré de protection qu'ils fournissent contre les piqûres de moustiques à ce moment. Après avoir discuté avec l'équipe de recherche sur les modes d'utilisation sûrs et efficaces, vous serez libre d'utiliser ces émanateurs de la manière que vous jugez la plus pratique et efficace, tant que vous n'ouvrez pas le support de protection ou ne l'utilisez d'aucune façon qui pourrait entrainer un contact physique direct avec le matériau imprégné.

En tant que représentant de votre foyer, nous vous demandons d'enregistrer ce que votre foyer perçoit comme les manières les plus et les moins efficaces d'utiliser ces dispositifs, en utilisant des appareils photo jetables qui seront fournis par l'équipe de recherche. Avant d'utiliser l'appareil photo, vous participerez à une courte réunion de formation au cours de laquelle seront expliqué comment utiliser l'appareil photo de façon acceptable, sans compromettre la sécurité, la vie privée et les autres droits des habitants du foyer ou voisins. Au début de chaque visite du foyer, une fois tous les deux mois, tout appareil photo totalement utilisé sera collecté par l'équipe de recherche

pour permettre le développement des photos en deux exemplaires. Un exemplaire vous sera remis après quelques jours, l'autre sera conservé confidentiellement par l'équipe de recherche comme double en cas de perte. Nous vous demandons aussi de participer à une réunion avec les photographes des autres foyers de votre voisinage, dans le but de sélectionner les photographies les plus informatives et de discuter de leur signification. Un facilitateur travaillant avec l'équipe de recherche va guider la discussion tandis qu'un observateur fera un enregistrement audio et prendra des notes sur ce qui a été dit. Aucune information personnelle ne sera enregistrée hormis votre nom, genre et âge, et ces détails seront gardés confidentiels. Votre participation et celle de votre foyer est entièrement volontaire et vous êtes libre de quitter l'étude à tout moment.

Vous ne recevrez pas de compensation pour votre participation à cette étude. Le seul bénéfice direct que vous recevrez sera la protection contre les piqûres de moustiques. Vous devriez aussi bénéficier indirectement de l'étude, parce que ses résultats permettront de contrôler plus efficacement les moustiques et les virus de la dengue, chikungunya et zika, permettant d'améliorer les conditions de vie pour vous, votre famille et le pays en général, tout en économisant de l'argent qui serait autrement consacré à des soins médicaux. En effet, les résultats de cette étude devraient avoir des implications très directes et significatives pour les décideurs politiques en Haïti, ainsi que les décideurs des organismes donateurs.

Le scientifique responsable de cette étude est le Pr Christian Raccurt, Doyen de la Faculté des Sciences Médicales de l'Université Quisqueya. Cette étude a été approuvée par le Comité National de Bioéthique du Ministère de la Santé publique et de la Population. Si vous avez des questions au sujet de cette étude, veuillez contacter Pr Raccurt au 3764 2584.
Pour plus d'information vous pourriez souhaiter contacter le Dr Cyrille Czeher au 3726 5230. Si vous n'êtes pas satisfait par les explications fournies, vous pouvez faire part de vos réserves auprès du Dr Gerald Lerebours, président du Comité National de Bioéthique, c/o AMH, 29, Avenue de la Ligue Féminine ci-devant 1 Avenue du Travail, Port-au-Prince; Téléphone 3701 5766.

Accord de Consentement Eclairé pour chaque participant à l'étude :

En tant que représentant de mon foyer, je soussigné ………………………………………………. comprends clairement les objectifs du projet intitulé **«ÉMANATEURS DE TRANSFLUTHRINE SIMPLES POUR PROTÉGER CONTRE LA TRANSMISSION DES VIRUS ZIKA, DENGUE ET CHIKUNGUNYA».** Je suis d'accord pour utiliser l'appareil photo fourni pour illustrer comment les émanateurs sont utilisés, et à participer à des discussions enregistrées au sujet de ces dispositifs. Je comprends que je peux révoquer mon consentement et quitter l'étude à tout moment.

Nom du participant à l'étude: ____

Sexe (Homme/Femme: ___________________________ Âge:______________________

Signature du participant à l'étude: _________________________Date___________________

Nom du Témoin: ____

Signature du Témoin: ______________________________________Date________________

Emanateurs de transfluthrine simples, abordables et évolutifs pour protéger contre la transmission des virus Zika, Dengue et Chikungunya

ANNEXE 11

GUIDE THEMATIQUE POUR LES GROUPES DE DISCUSSION SUR L'EXERCICE PHOTOVOICE

1. Présenter les images/photographies et demander aux participants d'expliquer leur signification et leur importance.

2. Obtenir les points de vue pour expliquer comment les photographies illustrent les avantages et inconvénients des dispositifs émanateurs de répulsif.

    2a. Protection contre les piqûres de moustiques.

    2b. Autres avantages.

    2c. Limites de la protection.

    2d. Autres inconvénients.

    2e. Autres facteurs influençant leur utilisation.

3. Obtenir les points de vue pour expliquer comment les photographies illustrent les pratiques d'utilisation des émanateurs répulsifs et les idées d'optimisation du dispositif, leur distribution ou leur entretien, dans le futur.

    3a. Bonnes pratiques d'utilisation.

    3b. Mauvaises pratiques d'utilisation.

    3c. Limites et inconvénients.

    3d. Idées d'amélioration du dispositif en lui-même.

    3e. Idées pour la distribution et l'entretien du dispositif.

Emanateurs de transfluthrine simples, abordables et évolutifs pour protéger contre la transmission des virus Zika, Dengue et Chikungunya

ANNEXE 12

FORMULAIRE DE CONSENTEMENT ECLAIRE SUPPLEMENTAIRE POUR LA PUBLICATION OU LA DISSEMINATION D'UNE PHOTOGRAPHIE SPECIFIQUE

NUMÉRO DE SÉRIE DE L'ACCORD DE CONSENTEMENT ÉCLAIRÉ (ICF1):___________________

NUMERO DE MAISON : _______________

Comme précédemment documenté par écrit (ICF1 __________), vous et votre foyer avez accepté de participer à un projet de recherche visant à évaluer un nouveau dispositif émanateur de répulsif pour la protection contre les piqûres de moustiques. Merci de consulter la fiche d'information des participants pour plus de détails. Un membre de votre foyer a également accepté de prendre des photographies pour illustrer comment vous et votre foyer utilisez ces dispositifs, ainsi que des idées pour améliorer leur conception, distribution et entretien. L'équipe de recherche souhaite maintenant publier ou partager la photographie ci-dessous de votre maison, son intérieur et/ou ses occupants, et vous demande votre permission écrite pour le faire. Nous demandons aussi l'autorisation écrite de toute personne visible sur la photographie pour la publier ou la partager, et nous vous serions reconnaissants d'assister pour identifier et contacter ces personnes afin d'obtenir leur consentement écrit sur ce même document. Nous sommes heureux d'éditer cette photographie pour masquer toute information personnellement identifiable, telle que des traits de visage, ou au sujet de votre ménage, maison ou effets personnels, jusqu'à ce que vous soyez satisfait et permettiez son utilisation. Votre participation à cette étude est entièrement volontaire et vous êtes libre de refuser que cette photographie soit publiée.

Vous ne recevrez pas de compensation pour votre participation à cette étude. Le seul bénéfice direct que vous recevrez sera la protection contre les piqûres de moustiques. Vous devriez aussi bénéficier indirectement de l'étude, parce que ses résultats permettront de contrôler plus efficacement les moustiques et les virus de la dengue, chikungunya et zika. Un tel bénéfice indirect sur le long terme permettrait d'améliorer les conditions de vie pour vous, votre famille et le pays en général, tout en économisant de l'argent qui serait autrement consacré à des soins médicaux. En effet, les résultats de cette étude devraient avoir des implications très directes et significatives pour les décideurs politiques en Haïti, ainsi que les décideurs des organismes donateurs.

INSERER LA PHOTOGRAPHIE ICI

Le scientifique responsable de cette étude est le Pr Christian Raccurt, Doyen de la Faculté des Sciences Médicales de l'Université Quisqueya. Cette étude a été approuvée par le Comité National de Bioéthique du Ministère de la Santé publique et de la Population. Si vous avez des questions au sujet de cette étude, veuillez contacter Pr Raccurt au 3764 2584.
Pour plus d'information vous pourriez souhaiter contacter le Dr Cyrille Czeher au 3726 5230. Si vous n'êtes pas satisfait par les explications fournies, vous pouvez faire part de vos réserves auprès du Dr Gerald Lerebours, président du Comité National de Bioéthique, c/o AMH, 29, Avenue de la Ligue Féminine ci-devant 1 Avenue du Travail, Port-au-Prince; Téléphone 3701 5766.

Accord de consentement éclairé du chef de foyer

En tant que chef de notre foyer, je soussigné ……………………………………………… comprends clairement les objectifs du projet intitulé **«ÉMANATEURS DE TRANSFLUTHRINE SIMPLES POUR PROTÉGER CONTRE LA TRANSMISSION DES VIRUS ZIKA, DENGUE ET CHIKUNGUNYA»**, et j'accepte que la photographie ci-dessus, prise dans ou autour de ma maison, peut être publiée ou partagée librement sous la forme exacte présentée ci-dessus.

Nom du participant à l'étude: ____

Sexe (Homme/Femme): ___________________________ Âge:_____________________

Signature du participant à l'étude: _________________________Date___________________

Nom du Témoin: ____

Signature du Témoin: _______________________________________Date________________

Accord de consentement éclairé de personnes présentes dans la photographie

En tant que personne présente dans la photographie ci-dessus, ou son parent/tuteur légal, je soussigné ……………………………………………… comprends clairement les objectifs du projet intitulé **«ÉMANATEURS DE TRANSFLUTHRINE SIMPLES POUR PROTÉGER CONTRE LA TRANSMISSION DES VIRUS ZIKA, DENGUE ET CHIKUNGUNYA»**, et j'accepte que la photographie peut être publiée ou partagée librement sous la forme exacte présentée ci-dessus.

Nom du participant à l'étude: ____

Sexe (Homme/Femme): ___________________________ Âge:_____________________

Signature du participant à l'étude: _________________________Date___________________

Nom du Témoin: ____

Signature du Témoin: _______________________________________Date________________

En tant que personne présente dans la photographie ci-dessus, ou son parent/tuteur légal, je soussigné ……………………………………………… comprends clairement les objectifs du projet intitulé **«ÉMANATEURS DE TRANSFLUTHRINE SIMPLES POUR PROTÉGER CONTRE LA TRANSMISSION DES VIRUS ZIKA, DENGUE ET CHIKUNGUNYA»**, et j'accepte que la photographie peut être publiée ou partagée librement sous la forme exacte présentée ci-dessus.

Nom du participant à l'étude: ____

Sexe (Homme/Femme): ___________________________ Âge:_____________________

Signature du participant à l'étude: _________________________Date___________________

Nom du Témoin: ____

Signature du Témoin: _______________________________________Date________________

En tant que personne présente dans la photographie ci-dessus, ou son parent/tuteur légal, je soussigné ……………………………………………… comprends clairement les objectifs du projet intitulé **«ÉMANATEURS DE TRANSFLUTHRINE SIMPLES POUR PROTÉGER CONTRE LA TRANSMISSION DES VIRUS ZIKA, DENGUE ET CHIKUNGUNYA»**, et j'accepte que la photographie peut être publiée ou partagée librement sous la forme exacte présentée ci-dessus.

Nom du participant à l'étude: ____

Sexe (Homme/Femme): ___________________________ Âge:_____________________

Signature du participant à l'étude: _________________________Date___________________

Nom du Témoin: ____

Signature du Témoin: _______________________________________Date________________

<u>**Emanatè transfluthrine senp, abòdab ak fleksib pou pwoteje kont transmisyon viris Zika, Dengue ak Chikungunya**</u>

ANEX 1

FÈY DE ENFÒMASYON DE PATISIPAN NAN KENBE MARENGWEN

Marengwen nwa ki gen mak blan ki an jeneral mòde pandan lajounen transmèt deng ak lòt viris tankou chikungunya, yo tout kapab lakòz lafyèv, kò fè mal ak yon kantite lòt sentòm lakay moun an Ayiti. Anplis de sa, marengwen sa a kapab transmèt viris Zika tou, ki lakòz malfòmasyon grav lakay ti bebe ki nan vant si fanm ansent lan enfekte. Paske yo mòde pandan jounen an, lè moun yo jeneralman ap fè aktivite, ni moustikè ni kay ki gen twil pa ka bay pwoteksyon adekwa kont maladi sa yo. Malerezman, plagatòks ak remèd po kont marengwen pa dire lontan leu w ou finn mete yo. Sa vinn rann mezi sa a yo twò chè pou goumen kont marengwen.

Gen you ekip rechèch ki fèt dènyèman epi yo devlope yon zouti ki senp, fasil pou itilize ki bay plis pase 90% pwoteksyon pou plis pase 4 mwa kont marengwen ki mòde nan mitan lannwit an Afrik. Nou envite ou patisipe nan yon etid pou evalye nouvo zouti sa a pou goumen kont marengwen ki mòde lajounen an Ayiti. Rezon ki fè etid la se pou yo demontre ke you nouvo fòm nan transfluthrin lan ka bay pwoteksyon abòdab kont marengwen ki bay pwoblem sa yo, pou omwen 6 mwa.

Pandan ke ansyen fòm transfluthrin lan (plakatòks) dwe boule epi bay lafimen, ak nouvo aparèy sa a ki pi senp, pwodwi a evapore natirèlman e li pwopaje nan lè a nan tanperati chanm kay la, se konsa ke li pwoteje espas deyò a ak yon vapè, se marengwen yo ki ap wè vapè sa a. Aparèy la senp, li genyen sèlman yon moso twal (sak) nan tèt li, sak yo konn itilize nan depo ak lan transpò sereyal tankou mayi ak diri, sak la tranpe avek transflutrine lan ki kouri dèyè marengwen, yo itilize pwodui sa a toupatou lan mond lan. Pwodui sa a pa chè li koute mwens pase 15 goud pou chak bal sak, yo trete twal la a san danje, sèl bagay yo sèvi avèk gan plastik kòm pwoteksyon. Depi plis pase dizan, itilizasyon transfluthrin toupatou lan mond lan montre yon dosye pozitif pou sekirite moun kap itilize li a. Sa fè plizyè dizènn lane depi yap sèvi ak transfluthrin lan nan aparèy kòm pwodui pou kouri dèyè marengwen toupatou nan Ewòp, Amerik, Lazi ak Lafrik epi peyi Tanzani apwouve li nan kad gwo evalyasyon eksperimantal.

Nan etid sa a, plizyè eksperyans ap mezire ki pwoteksyon yo bay nan diferan fòm emanatè kont marengwen ki mòde. Kòm yon patisipan, ou pral kenbe marengwen ak yon pèlen kadriyaj elektrik, li fèt ak yon ankadreman plastik kare ki gen fil ki gen kouran. Ankadreman ki nan pèlen an an plastik, lap pwoteje pye ou andedan pyej la, sou deyò ankadreman an genyen fil elektrik yo ki pote ase kouran pou yo touye marengwen ki wè pye w epi kap eseye vinn poze,men kouran sa a ka fè moun anyen . Chak jou nan eksperimantasyon, ou pral itilize pèlen sa a pou yon peryòd de 3 èdtan nan maten bonè ak yon lòt peryòd de 3 èdtan nan aswè . Pandan peryod sa a, ap gen kapti chak jou, ou pral bezwen chita sou yon chèz epi wap mete pye ou andedan kare ankadreman an, pandan yap kouvri rès kò ou,ekswepté figi ou, pou anpeche marengwen mòde ou. Rad sa yo pral kouvri kò w la gratis nan pwojè a.

Ou pral chita ak pye ou nan ankadreman an plastik la pou 45 minit pandan chak peryòd 1 èdtan. Nan fen chak 45 minit ou fè chita ak pye ou nan inite sa a, ou pral pran yon ti repo 15 minit, rilaks epi bwè you ti rafrechisman gratis. Ou pral repete egzèsis 45 minit sa a ak 15 minit rilaks la ankò, pandan 3 èdtan an tou nan maten e 3 èdtan nan aswè, chak jou ke w patisipe. Ou dwe patisipe pandan 8 jou kap gaye sou 2 semènn travay, ap genyen yon sèl seyans chak 2 mwa pou you peryod de 6 mwa oswa jiskaske ou deside kite etid la. Li pa fasil pou marengwen mòde w pandan w chita ak janm ou andedan aparèy la, men pandan 15-minit repo a, menm jan ak leu ou kanpe deyò lakay ou marengwen kapab pwofite mòde ou. Se poutèt sa nou ankouraje w pou pwoteje pye ou yo pandan 15 minit poz yo ak pantalon long epi soulye ak chosèt, men nap mande ou pou pa aplike remèd pou kouri dèyè marengwen, ki pral bay etid kap fèt la pwoblèm.

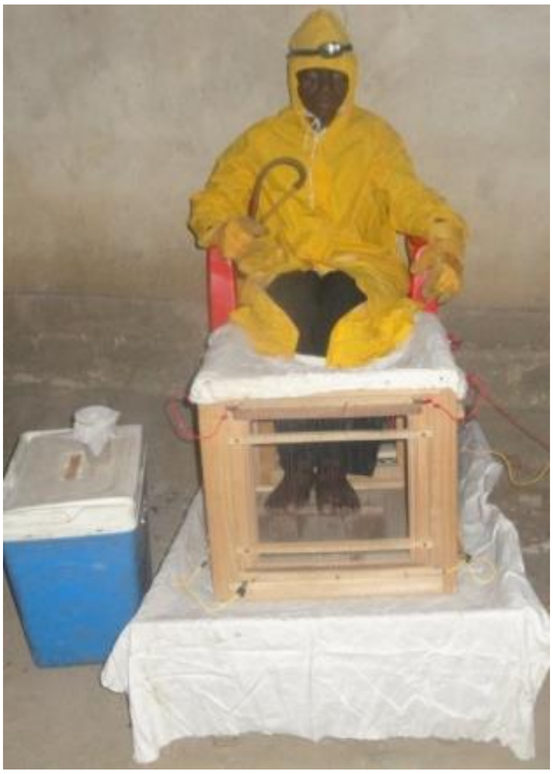

Aktivite sa yo ap fèt nan zòn ki an plenè, nan jaden tou pre lakay ou, ke nou te vizite avèk ou pou nou te diskite sou etid la. Kèk jou ou pral genyen pou ou resevwa yon ankadreman ki gen yon moso twal tranpe ak transfluthrin, tandis ke yon lòt jou, li pap genyen okenn remèd pou marengwen. Ankadreman ki gen moso twal (tranpe oswa pa tranpe) yo pral mete li nan do chèz ou a, imedyatman dèyè ou. Si ou patisipe nan etid sa a, ou pap gen pou manyen transfluthrin oswa menm moso twal ki tranpe ak pwodui sila a : Se envestigatè yo kap fè tout pwosedi tretman an, se yo kap manyen materyèl sa yo avek gan nan men yo. Sèlman ti kontak wap genyen ak transflitin lan se pral vapè moso twal ki tranpe yo pral degaje tou prè ou, pandan vapè a ap degaje dousman nan lè a epi dekouraje marengwen yo, konsantrasyon li plis pase 1000 fwa anba limit ke yo apwouve kòm san danje pou itilize. Pwodwi sa a itilize toupatou nan lemond sou fòm plagato ak lòt aparèy pou kouri dèyè marengwen. Nivo Transfluthrin ki degaje nan aparèy sa a yo tèlman ba ke yo pa reprezante ankenn danje pou lasante.

Wap patisipe kòm volontè e ou lib pou kite etid la nenpòt ki lè sou vle. Sèl risk ki genyen nan patisipasyon nan etid sa a se posibilite pou pran kouran si w pa fè atansyon nan manyen pati deyò gri a ki gen kouran ladan li avan yo koupe kouran an. Si w manyen kouran sa a li ka fè ou sezi e rann ou mal alèz, men se yon ti kouran ki pa ka ni tuye ou ni reprezante yon danje pou sante ou. Si ou gen pwoblèm avek kesyon kouran an, ou kapab kite etid la nan nenpòt ki lè. Ou pral resevwa yon ankourajman ki se 15 US $ pou chak jou ke ou patisipe, lajan sa a ap pèmèt ou manje, epi jere lòt ti malèz si genyen. Sèl lòt benefis ou pral resevwa a se ke, nou espere ke rezilta etid sa a ap pi efikas nan kontwole marengwen ak deng, chikungunya ak viris zika, amelyore kondisyon lavi yo pou ou, madanm ou, pitit ou fanmiy ou ak peyi a an jeneral, pandan yap ekonomize lajan ki ta ka depanse nan swen medikal.

Syantis ki responsab etid sa a se Pwofesè Christian Raccurt, Dwayen nan fakilte medsinn nan Inivèsite Quisqueya. Komite Bioetik Nasyonal nan Ministè Sante Piblik ak Popilasyon te apwouve etid sa a. Si w gen nenpòt kesyon sou etid sa a, tanpri kontakte Pwofesè Christian Raccurt nan 3764 2584. Pou plis enfòmasyon ou ka toujou kontakte doktè Cyrille Czeher nan 3726 5230. Si ou pa satisfè ak eksplikasyon yo bay yo, ou ka ekri doktè Gerald Lerebours, Prezidan Komite Bioethik Nasyonal la, c/o AMH, 29, Avenue de la Ligue Feminine ci-devant 1 Avenue du Travail, Pòtoprens; Telefòn 3701 5766.

Emanatè transfluthrine senp, abòdab ak fleksib pou pwoteje kont transmisyon viris Zika, Dengue ak Chikungunya

ANEX 2
AKÒ KONSANTMAN POU GRANMOUN PATISIPE NAN KENBE MARENGWEN

NIMEWO SERI AKÒ KONSANTMAN : ________________

Marengwen Aedes egipti gen koulè nwa ak mak blan, epi an jeneral li mòde pandan gwo midi. Isit la an Ayiti, yo transmèt viris deng ak chikungunya, ki lakòz le pli souvan lafyèv, doulè nan kò ak yon kantite lòt sentòm. Anplis de sa, moustik sa a transmèt viris Zika, ki ka lakòz domaj grav lakay ti bebe ki nan vant si fanm ansent lan te enfekte.

Nou envite ou patisipe nan yon etid pou evalye yon nouvo zouti ki anpeche marengwen mòde moun. Objektif etid sa a se demontre ke yon nouvo fòm transfluthrin, menm ensektisid san danje ke yo konn itilize nan plakatòks la ka bay pwoteksyon abòdab kont marengwen pandan omwen 6 mwa. Remake byen ke nan apwòch tou nèf nou an, ensektisid la evapore natirèlman epi vapè a gaye nan lè a, tandis ke plakatòks yo dwe boule li epi ou toujou ap pwodwi lafimen.

Kòm yon patisipan, ou pral kenbe marengwen ak yon pèlen kadriyaj elektrik, li fèt ak yon ankadreman plastik kare ki gen fil ki gen kouran. Ankadreman ki nan pèlen an an plastik, lap pwoteje pye ou andedan pyej la, sou deyò ankadreman an genyen fil elektrik yo ki pote ase kouran pou yo touye marengwen ki wè pye w epi kap eseye vinn poze, men kouran sa a ka fè moun anyen. Chak jou nan eksperimantasyon, ou pral itilize pèlen sa a pou yon peryòd de 3 èdtan nan maten bonè ak yon lòt peryòd de 3 èdtan nan aswè. Pandan peryod sa a, ap gen kapti chak jou, ou pral bezwen chita sou yon chèz epi wap mete pye ou andedan kare ankadreman an, pandan yap kouvri rès kò ou, eksepté figi ou, pou anpeche marengwen mòde ou. Rad sa yo pral kouvri kò w la gratis nan pwojè a. Wap gen pou chita tou kole avek yon moso twal ki genyen yon remèd kont marengwen ki rele transfluthrine. Remèd sa a kouri dèyè marengwen gras ak vapè li fè tou dousman nan lè a. Vapè ki ap degaje dousman nan lè a epi dekouraje marengwen yo , konsantrasyon li plis pase 1000 fwa anba limit ke yo apwouve kòm san danje pou itilize. Pwodwi sa a itilize toupatou nan lemond sou fòm plagatox ak lòt aparèy pou kouri dèyè marengwen. Nivo transfluthrine ki degaje nan aparèy sa a yo tèlman ba ke yo pa reprezante ankenn danje pou lasante.

Wap patisipe kòm volontè e ou lib pou kite etid la nenpòt ki lè sou vle. Sèl risk ki genyen nan patisipasyon nan etid sa a se posibilite pou pran kouran si w pa fè atansyon nan manyen pati deyò gri a ki gen kouran ladan li avan yo koupe kouran an. Si w manyen kouran sa a li ka fè ou sezi e rann ou mal alèz, men se yon ti kouran ki pa ka ni tuye w ni reprezante yon danje pou sante ou. Si ou gen pwoblèm avek kesyon kouran, ou kapab kite etid la nan nenpòt ki lè. Ou pral resevwa yon ankourajman ki se 15 US $ pou chak jou ke ou patisipe, lajan sa a ap pèmèt ou manje, epi jere lòt

ti malèz si genyen. Sèl lòt benefis ou pral resevwa a se ke, nou espere ke rezilta etid sa a ap pi efikas nan kontwole marengwen ak deng, chikungunya ak viris zika, amelyore kondisyon lavi yo pou ou, madanm ou, pitit ou, fanmiy ou ak peyi a an jeneral, pandan yap ekonomize lajan ki ta ka depanse nan swen medikal. Vreman vre, rezilta etid sa a ta dwe gen anpil angajman ak enplikasyon politisyen ayisyen yo, ak tout bayè yo.

Syantis ki responsab etid sa a se Pwofesè Christian Raccurt, Dwayen nan fakilte medsinn nan Inivèsite Quisqueya. Komite Bioetik Nasyonal nan Ministè Sante Piblik ak Popilasyon te apwouve etid sa a. Si w gen nenpòt kesyon sou etid sa a, tanpri kontakte Pwofesè Christian Raccurt nan 3764 2584. Pou plis enfòmasyon ou ka toujou kontakte doktè Cyrille Czeher nan 3726 5230. Si ou pa satisfè ak eksplikasyon yo bay yo, ou ka ekri doktè Gerald Lerebours, Prezidan Komite Bioethik Nasyonal la, c/o AMH, 29, Avenue de la Ligue Feminine, 1 Avenue du Travail, Pòtoprens; Telefòn 3701 5766.

Akò konsantman pou chak patisipan nan etid la:

Mwen, ki siyen an .... Mwen konprann a klè objèktif pwojè a ki gen pou tit **"Emanatè transfluthrine senp, abòdab ak fleksib pou pwoteje kont transmisyon viris Zika, Dengue ak Chikungunya"** epi mwen dakò pou patisipe nan etid la. Mwen konprann ke itilizasyon pèlen kadriyaj elektrik la ka ekspoze'm ak yon risk pou m pran kouran men kouran sa a fèb e li pa kapab tuyem ni banm pwoblem sante. Mwen konprann ke mwen ka anile konsantman mwen epi kite etid la nan nenpòt ki lè.

Non patisipan an nan etid la: ____

Sèks (Gason / Fi): ________________________ Laj ___________________________

Siyati patisipan an nan etid la: ______________________ Date ___________________

Non Temwen: ____

Siyati Temwen an: ___________________________________ Date ______________

Emanatè transfluthrine senp, abòdab ak fleksib pou pwoteje kont transmisyon viris Zika, Dengue ak Chikungunya

ANEX 3
AKÒ KONSANTMAN POU MOUN KI MÈT OSWA RESPONSAB JESYON YON TÈ POU EVALYASYON REMED KONT MARENGWEN

NIMEWO SERI AKÒ KONSANTMAN : ________________

Marengwen Aedes egipti gen koulè nwa ak mak blan, epi an jeneral li mòde pandan gwo midi. Isit la an Ayiti, yo transmèt viris deng ak chikungunya, ki lakòz le pli souvan lafyèv, doulè nan kò ak yon kantite lòt sentòm. Anplis de sa, moustik sa a transmèt viris Zika, ki ka lakòz domaj grav lakay ti bebe ki nan vant si fanm ansent lan te enfekte.

Nou ta renmen pèmisyon ou, pou ou menm ak tout moun lakay ou kapab patisipe nan yon etid pou evalye yon nouvo zouti pou anpeche marengwen mòde moun. Objektif etid la se pou demontre ke gen yon nouvo fòm transfluthrin, menm remèd pou marengwen ki san danje epi ke yo itilize souvan nan fè plagatoks la , ka bay pwoteksyon abòdab kont marengwen sa yo pandan omwen 6 mwa.

Espesyalman, nou vle kenbe marengwen ,donk nap mande ou pèmisyon ou pou 2 manm nan ekip rechèch nou an ka kenbe marengwen yo ki ap eseye mòde yo sou teren w lan jodi a, pou yon peryòd de 3 èdtan nan maten an ak yon lòt peryòd de 3 è nan aswè a. Manm lan ekip nou yo pral chita sou yon chèz ,yap mete janm yo nan yon pèlen pou marengwen. Pyèj yo fèt ak yon ankadreman kare ki gen kouran , pandan ke tout rès kò yo sof figi yo, pral kouvri ak rad pwoteksyon pou anpeche marengwen mòde yo . Yo pa ta dwe deranje pèsonn, epi pa fè ankenn egjijans ak moun lakay ou oswa vwazen ou yo. Nan kèk okazyon, patisipan yo ap resevwa yon moso twal ,moso twal sa a ki kapab tranpe avèk yon remèd kont marengwen ke yo itilize toupatou ,ki efikas,ki respekte sante ak sekirite moun ke yo rele transfluthrin. Transfluthrin sa pral repouse marengwen yo tou dousman, lap evapore nan lè a nan yon konsantrasyon plis pase 1000 fwa anba limit limit sekirite ke yo apwouve pou remèd pou marengwen ke yap itilize toupatou lan mond lan dwe genyen.

Wap patisipe kòm volontè e ou lib pou kite etid la nenpòt ki lè sou vle. Pa gen risk pou ou, fanmi ou oswa katye a si ou patisipe nan etid la. Sèl benefis ou pral resevwa a se ke, nou espere ke rezilta etid sa a ap pi efikas nan kontwole marengwen ak deng, chikungunya ak viris zika, amelyore kondisyon lavi yo pou ou, madanm ou, pitit ou, fanmiy ou ak peyi a an jeneral, pandan yap ekonomize lajan ki ta ka depanse nan swen medikal.

Syantis ki responsab etid sa a se Pwofesè Christian Raccurt, Dwayen nan fakilte medsinn nan Inivèsite Quisqueya. Komite Bioetik Nasyonal nan Ministè Sante Piblik ak Popilasyon te apwouve etid sa a. Si w gen nenpòt kesyon sou etid sa a, tanpri kontakte Pwofesè Christian Raccurt nan 3764 2584.

Pou plis enfòmasyon ou ka toujou kontakte doktè Cyrille Czeher nan 3726 5230. Si ou pa satisfè ak eksplikasyon yo bay yo, ou ka ekri doktè Gerald Lerebours, Prezidan Komite Bioethik Nasyonal la, c/o AMH, 29, Avenue de la Ligue Feminine ci-devant 1 Avenue du Travail, Pòtoprens; Telefòn 3701 5766.

Akò konsantman pou chak patisipan nan etid la:

Mwen, ki siyen an .... Mwen byen konprann a klè objèktif pwojè a ki gen pou tit **"Emanatè transfluthrine senp, abòdab ak fleksib pou pwoteje kont transmisyon viris Zika, Dengue ak Chikungunya"** e mwen otorize 2 manm nan ekip ke mwen rankontre jodia, kenbe marengwen sou teren mwen an pou yo kapab fè yon etid. Mwen konprann ke 2 manm nan ekip rechèch la pral kenbe marengwen an plenè deyò a jodi a sou teren mwen an. Mwen pa atann ke yap anmède pèsonn sou teren an epi yo pap mande m pou m peye pou sa a e yo pap fèm lòt egzijans nonplis, ni a mwen menm, ni ak moun lakay mwen oswa vwazen m 'yo. Mwen konprann ke mwen patisipe kòm volontè e mwen ka revoke konsantman mwen epi kite etid la nan nenpòt ki lè.

Non patisipan an nan etid la: ____

Sèks (Gason / Fi): _________________________ Laj ___________________________

Siyati patisipan an nan etid la: ______________________ Date ___________________

Non Temwen: ____

Siyati Temwen an: ___________________________________ Date _______________

Emanatè transfluthrine senp, abòdab ak fleksib pou pwoteje kont transmisyon viris Zika, Dengue ak Chikungunya

ANEX 4
FÈY DE ENFÒMASYON DE PATISIPAN KI POU SERVI AK TRANSFLUTHRINE LAN REGILYEMAN NAN KAY

Marengwen nwa ki gen mak blan ki an jeneral mòde pandan lajounen transmèt deng ak lòt viris tankou chikungunya, yo tout kapab lakòz lafyèv, kò fè mal ak yon kantite lòt sentòm lakay moun an Ayiti. Anplis de sa, marengwen sa a kapab transmèt viris Zika tou, ki lakòz malfòmasyon grav lakay ti bebe ki nan vant si fanm ansent lan enfekte. Paske yo mòde pandan jounen an, lè moun yo jeneralman ap fè aktivite, ni moustikè ni kay ki gen twil pa ka bay pwoteksyon adekwa kont maladi sa yo. Malerezman, plagatòks ak remèd po kont marengwen pa dire lontan leu w ou finn mete yo. Sa vinn rann mezi sa a yo twò chè pou goumen kont marengwen.

Gen you ekip rechèch ki fèt dènyèman epi yo devlope yon zouti ki senp, fasil pou itilize ki bay plis pase 90% pwoteksyon pou plis pase 4 mwa kont marengwen ki mòde nan mitan lannwit an Afrik.
Nou envite ou patisipe nan yon etid pou evalye nouvo zouti sa a pou goumen kont marengwen ki mòde lajounen an Ayiti. Rezon ki fè etid la se pou yo demontre ke you nouvo fòm nan transfluthrin lan ka bay pwoteksyon abòdab kont marengwen ki bay pwoblem sa yo, pou omwen 6 mwa.

Pandan ke ansyen fòm transfluthrin lan (plakatòks) dwe boule epi bay lafimen, ak nouvo aparèy sa a ki pi senp, pwodwi a evapore natirèlman e li pwopaje nan lè a nan tanperati chanm kay la, se konsa ke li pwoteje espas deyò a ak yon vapè, se marengwen yo ki ap wè vapè sa a. Aparèy la senp, li genyen sèlman yon moso twal (sak) nan tèt li, sak yo konn itilize nan depo ak lan transpò sereyal tankou mayi ak diri, sak la tranpe avek transflutrine lan ki kouri dèyè marengwen, yo itilize pwodui sa a toupatou lan mond lan. Pwodui sa a pa chè li koute mwens pase 15 goud pou chak bal sak, yo trete twal la a san danje, sèl bagay yo sèvi avèk gan plastik kòm pwoteksyon. Depi plis pase dizan, itilizasyon transfluthrin toupatou lan mond lan montre yon dosye pozitif pou sekirite moun kap itilize li a. Sa fè plizyè dizènn lane depi yap sèvi ak transfluthrin lan nan aparèy kòm pwodui pou kouri dèyè marengwen toupatou nan Ewòp, Amerik, Lazi ak Lafrik epi peyi Tanzani apwouve li nan kad gwo evalyasyon eksperimantal.

Youn nan eksperyans yo te planifye nan etid sa a prevwa pou yo bay 2 nan emanatè yo nan yon ti kantite kay ke yo chwazi avek konsantman mèt kay la a, pou yo sèvi ak emanatè sa a yo pou pwoteje tèt yo kont marengwen pou yon peryòd de 6 mwa. Si oumenm ak tout moun lakay ou dakò pou patisipe nan etid la, ekip rechèch la pral vizite kay ou yon fwa chak de mwa epi rekipere emanatè yo pou apeprè 2 semèn pou yo mezire degre pwoteksyon ke yo bay kont marengwen ki mòde nan moman an. Apre ou fin pale ak ekip rechèch la sou fason pou itilize emanatèa san danje epi pou li efikas, ou pral lib pou itilize li nan fason ou panse ki pi bon pou kwape marengwen, depi ke ou pa louvri etajè pwoteksyon an oswa pa sèvi ak li nan nenpòt fason ki ta ka lakòz kontak dirèk ant kò w ak materyèl ki tranpe a.

Epitou, yo pral mande yonn nan moun nan kay la pou w anrejistre nan yon kamera plastik ki sa kay la panse, ki pi move fason ak fason ki pi efikas ke yo itilize aparèy la, se ekip rechèch la kap bay ti kamera sa a nan kay la pou anrejistreman an. Anvan ou sèvi ak kamera a, moun lan ki idantifye kòm responsab ki pou pran foto yo ap patisipe nan yon ti sesyon fòmasyon, nan fomasyon sila a yo pral eksplike li kouman yo sèvi ak kamera a nan yon fason ki akseptab, san yo pa konpwomèt sekirite, sou vi prive ak lòt dwa moun ki abite nan kay la oswa vwazen yo. Anplis de sa, chak fwa yo vizite kay yo yon fwa chak 2 mwa pou rekipere epi rapòte emanatè yo pou tès efikasite, yo pral mande w pou w ranpli yon kesyonè pou evalye nivo satisfaksyon ou de demach ke noua p menen pou pwoteje ou kont marengwen ki ap mòde ou yo. Nan kòmansman chak vizit nan kay la, ekip rechèch la ap resevwa tout kamera ki finn itilize yo, se sa a ki pral pèmèt ke yo fè devlopman foto yo en 2 kopi ,youn pou ou, youn pou ekip rechèch ke lap konsève pou si pa w la ta pèdi. Ekip la pa gen dwa pibliye foto sa a.

Nan fen etid la, yo pral mande yon granmoun fanm ki konsène nan kay la, pou rejwenn 7 lot fanm ki soti nan 7 lot kay pou patisipe nan yon diskisyon an gwoup de 8 moun sou kesyon emanatè a. Apresa, yo pral fè menm bagay la pou gason yo tou Nan chak diskisyon gwoup, yon fasilitatè k ap travay avèk ekip rechèch la ap gide diskisyon an pandan yon obsèvatè pral fè yon anrejistreman odyo epi pran nòt sou sa ki te di yo. Yap pwofite de moman sa a pou yo mande moun nan kay la ki te responsab pou pran foto yo chwazi ki foto ki pi bon e ki bay plis enfòmasyon epi diskite sou siyifikasyon foto yo ak rès gwoup la epi fotograf ki soti nan lòt kay. Se selman volontè ki pou patisipe epi yo kapab kite etid la nenpòt ki lè yo deside. Si ekip rechèch la ta vle pibliye nenpòt foto ke moun ki nan kay te pran ki montre kay la, sa ki ladan li oswa manm kay la, ekip la ap chita ak tèt fanmi an pou yon konsantman alekri avan li fè sa, e tèt fanmiy lan gen dwa pou li anpeche piblikasyon foto sa a.

Ou pap resevwa konpansasyon pou patisipasyon ou nan etid sa a. Sèlman benefis dirèk ou pral resevwa se pwoteksyon kont moustik mòde. Ou ta dwe tou benefisye endirèkteman nan etid la, paske rezilta li yo pral pi plis kontwole moustik ak deng, chikungunya ak zika viris, amelyore kondisyon lavi yo pou ou, fanmi ou ak peyi a an jeneral, pandan y ap ekonomize lajan ki ta ka depanse nan swen medikal. Vreman vre, rezilta etid sa a ta dwe gen anpil dirèk ak siyifikatif enplikasyon pou politisyen ayisyen, osi byen ke mizisyen politik nan ajans donatè yo.

Syantis ki responsab etid sa a se Pwofesè Christian Raccurt, Dwayen nan fakilte medsinn nan Inivèsite Quisqueya. Komite Bioetik Nasyonal nan Ministè Sante Piblik ak Popilasyon te apwouve etid sa a. Si w gen nenpòt kesyon sou etid sa a, tanpri kontakte Pwofesè Christian Raccurt nan 3764 2584. Pou plis enfòmasyon ou ka toujou kontakte doktè Cyrille Czeher nan 3726 5230. Si ou pa satisfè ak eksplikasyon yo bay yo, ou ka ekri doktè Gerald Lerebours, Prezidan Komite Bioethik Nasyonal la, c/o AMH, 29, Avenue de la Ligue Feminine, 1 Avenue du Travail, Pòtoprens; Telefòn 3701 5766.

Emanatè transfluthrine senp, abòdab ak fleksib pou pwoteje kont transmisyon viris Zika, Dengue ak Chikungunya

ANEX 5

AKÒ KONSANTMAN TET FANMIY LAN KI PRAL ITILIZE EMANATÈ A

NIMEWO SERI AKÒ KONSANTMAN : _________________

Marengwen Aedes egipti gen koulè nwa ak mak blan, epi an jeneral li mòde pandan gwo midi. Isit la an Ayiti, yo transmèt viris deng ak chikungunya, ki lakòz le pli souvan lafyèv, doulè nan kò ak yon kantite lòt sentòm. Anplis de sa, moustik sa a transmèt viris Zika, ki ka lakòz domaj grav lakay ti bebe ki nan vant si fanm ansent lan te enfekte.

Nou envite ou yo patisipe nan yon etid yo evalye yon nouvo zouti yo anpeche mòde soti nan moustik yo. Objektif la nan etid la se yo demontre ke yon fòmilasyon nouvo sou transfluthrin, ensektisid nan menm san danje epi yo souvan itilize nan anwoulman moustik ka bay pwoteksyon abòdab kont moustik sa yo pou omwen 6 mwa. Remake byen ke nan apwòch nouvo nou an, ensektisid natirèlman evapore epi gaye nan lè a osi vapè, pandan y ap anwoulman yo moustik yo dwe boule epi ou toujou ap pwodwi lafimen.
Youn nan eksperyans yo te planifye nan etid sa a enplike nan bay de nan sa yo emanateur nan yon ti kantite konsantman lokatè, pou yo sèvi ak yo pwoteje tèt yo kont moustik pou yon peryòd de 6 mwa. Si oumenm ak tout moun lakay ou dakò pou patisipe nan pati nan etid la, ekip rechèch la pral vizite kay ou yon fwa chak de mwa epi rekipere emanators yo pou apeprè de semèn pou yo mezire degre pwoteksyon yo bay kont moustik mòde nan moman sa a. Apre ou fin pale ak ekip rechèch la sou itilizasyon san danje epi efikas, ou pral lib yo itilize sa yo emèt nan fason ou panse pi bon an ak efikas, osi lontan ke ou pa louvri etajè a pwoteksyon oswa pa sèvi ak li nan nenpòt fason ki ta ka lakòz kontak dirèk fizik ak materyèl la enpreye.

Epitou, yo pral mande yon manm nan kay la pou w anrejistre ki sa kay la pèsepsyon pou pi piti ak pi efikas fason yo itilize aparèy la, lè l sèvi avèk yon kamera jetab ki pral founi pa kay la. rechèch ekip la. Anvan ou sèvi ak kamera a, moun nan idantifye kòm responsab pou pran foto yo ap patisipe nan yon sesyon fòmasyon kout pandan ki yo pral eksplike kouman yo sèvi ak kamera a nan yon fason ki akseptab, san yo pa konpwomèt sekirite, sou vi prive ak lòt dwa moun ki abite nan kay la oswa vwazen yo. Anplis de sa, lè chak kay ap vizite yon fwa chak de mwa pou rekipere epi rapòte emanateurs yo pou tès efikasite, yo pral mande w pou w ranpli yon kesyonè, semi-estriktire pou evalye nivo ou. satisfaksyon ak pwoteksyon kont moustik mòde. Nan kòmansman chak vizit nan kay la, nenpòt ki kamera konplètman itilize yo pral kolekte pa ekip la rechèch yo ki pèmèt devlopman nan foto yo nan kopi. Y ap bay yon kopi apre yon kèk jou, lòt la pral kenbe konfidansyèlman pa ekip rechèch la kòm doub nan ka ta gen pèt.

Nan fen etid la, yo pral mande yon fanm adilt ki konsène nan kay la pou patisipe nan yon diskisyon sou gwoup emanatè, ak jiska 8 lòt fanm k ap patisipe nan lòt kay ki te itilize tou emanators. Menm jan an tou, yon gason granmoun ki konsantman ki soti nan chak kay ap patisipe nan yon diskisyon gwoup sou emanator la ak yon gwoup gason nan menm gwosè a. Nan chak diskisyon gwoup, yon

fasilitatè k ap travay avèk ekip rechèch la ap gide diskisyon an pandan yon obsèvatè pral fè yon anrejistreman odyo epi pran nòt sou sa ki te di. Menm jan an tou, moun nan kay la ki responsab pou pran foto yo pral mande yo chwazi pi bon an ak pi enfòmatif, ak Lè sa a, diskite sou siyifikasyon yo nan ti gwoup ak fotogwaf soti nan lòt kay. Patisipasyon nan kay ou a ak chak nan manm li yo se antyèman volontè, epi ou yo gratis kite etid la nan nenpòt ki lè si ou deside. Si ekip rechèch la ta vle pibliye nenpòt foto moun ki nan kay la ki te montre kay la, sa ki ladan li oswa okipan yo, yo pral tèt la nan fanmi an premye konsilte epi yo pral mande yo bay konsantman alekri yo, ki yo te kapab refize lib.

Ou pap resevwa konpansasyon pou patisipasyon ou nan etid sa a. Sèlman benefis dirèk ou pral resevwa se pwoteksyon kont moustik mòde. Ou ta dwe tou benefisye endirèkteman nan etid la, paske rezilta li yo pral pi plis kontwole moustik ak deng, chikungunya ak zika viris, amelyore kondisyon lavi yo pou ou, fanmi ou ak peyi a an jeneral, pandan y ap ekonomize lajan ki ta ka depanse nan swen medikal. Vreman vre, rezilta etid sa a ta dwe gen anpil dirèk ak siyifikatif enplikasyon pou politisyen ayisyen, osi byen ke mizisyen politik nan ajans donatè yo.

Syantis ki responsab etid sa a se Pwofesè Christian Raccurt, Dwayen nan fakilte medsinn nan Inivèsite Quisqueya. Komite Bioetik Nasyonal nan Ministè Sante Piblik ak Popilasyon te apwouve etid sa a. Si w gen nenpòt kesyon sou etid sa a, tanpri kontakte Pwofesè Christian Raccurt nan 3764 2584. Pou plis enfòmasyon ou ka toujou kontakte doktè Cyrille Czeher nan 3726 5230. Si ou pa satisfè ak eksplikasyon yo bay yo, ou ka ekri doktè Gerald Lerebours, Prezidan Komite Bioethik Nasyonal la, c/o AMH, 29, Avenue de la Ligue Feminine ci-devant 1 Avenue du Travail, Pòtoprens; Telefòn 3701 5766.

Akò konsantman pou chak patisipan nan etid la:

Kòm tèt nan kay mwen, mwen, ki siyen .... Mwen konprann klè objektif pwojè a ki rele **"Emanatè transfluthrine senp, abòdab ak fleksib pou pwoteje kont transmisyon viris Zika, Dengue ak Chikungunya".** Mwen dakò ke moun lakay mwen ap patisipe nan etid la e yo ap sèvi avèk emanatè ke ekip rechèch la ap ba yo. Mwen aksepte tou ke manm fanmiy mwen ap patisipe nan diskisyon ke ekip la ap anrejistre sou aparèy sa yo, e youn nan manm fanmi lan ap pran foto ki montre kouman itilizasyon an fèt. Mwen menm mwen konprann ke ekip rechèch la dwe chita avèk mwen pou jwenn yon konsantman alekri anvan li pibliye nenpòt foto ki te pran nan kay mwen, oswa pou pataje li ak nenpòt moun ki soti andeyò kay la ak ekip rechèch la. Mwen konprann ke mwen ka anile konsantman mwen an epi kite etid la nan nenpòt ki lè.

Non patisipan an nan etid la: ____

Sèks (Gason / Fi): ___________________________ Laj ______________________________

Siyati patisipan an nan etid la: ________________________ Date _____________________

Non Temwen: ____

Siyati Temwen an: _______________________________________ Date ________________

<u>**Emanatè transfluthrine senp, abòdab ak fleksib pou pwoteje kont transmisyon viris Zika, Dengue ak Chikungunya**</u>

ANEX 6
AKÒ KONSANTMAN POU RESPONSAB NAN KAY KAP RESPONN KESYON SOU ITILIZASYON EMANATÈ

NIMEWO SERI AKÒ KONSANTMAN : ___________________

Marengwen Aedes egipti gen koulè nwa ak mak blan, epi an jeneral li mòde pandan gwo midi. Isit la an Ayiti, yo transmèt viris deng ak chikungunya, ki lakòz le pli souvan lafyèv, doulè nan kò ak yon kantite lòt sentòm. Anplis de sa, moustik sa a transmèt viris Zika, ki ka lakòz domaj grav lakay ti bebe ki nan vant si fanm ansent lan te enfekte.

Nou envite ou yo patisipe nan yon etid yo evalye yon nouvo zouti yo anpeche mòde soti nan moustik yo. Objektif la nan etid la se yo demontre ke yon fòmilasyon nouvo sou transfluthrin, ensektisid nan menm san danje epi yo souvan itilize nan anwoulman moustik ka bay pwoteksyon abòdab kont moustik sa yo pou omwen 6 mwa. Remake byen ke nan apwòch nouvo nou an, ensektisid natirèlman evapore epi gaye nan lè a osi vapè, pandan y ap anwoulman yo moustik yo dwe boule epi ou toujou ap pwodwi lafimen.

Youn nan eksperyans yo te planifye nan etid sa a enplike nan bay de nan sa yo emanateur nan yon ti kantite konsantman lokatè, pou yo sèvi ak yo pwoteje tèt yo kont moustik pou yon peryòd de 6 mwa. Si oumenm ak tout moun lakay ou dakò pou patisipe nan pati nan etid la, ekip rechèch la pral vizite kay ou yon fwa chak de mwa epi rekipere emanators yo pou apeprè de semèn pou yo mezire degre pwoteksyon yo bay kont moustik mòde nan moman sa a. Apre ou fin pale ak ekip rechèch la sou itilizasyon san danje epi efikas, ou pral lib yo itilize sa yo emèt nan fason ou panse pi bon an ak efikas, osi lontan ke ou pa louvri etajè a pwoteksyon oswa pa sèvi ak li nan nenpòt fason ki ta ka lakòz kontak dirèk fizik ak materyèl la enpreye. Anplis de sa, kòm yon manm nan kay la, yo pral mande w pou reponn yon kesyonè kout pou evalye nivo satisfaksyon ou avèk pwoteksyon kont moustik mòde yo. Yo pap anrejistre enfòmasyon pèsonèl yo deyò non ou, sèks ak laj, detay sa yo ap rete konfidansyèl. Patisipasyon ou ak sa nan kay ou a se antyèman volontè epi ou yo gratis yo kite etid la nan nenpòt ki lè.

Ou pap resevwa konpansasyon pou patisipasyon ou nan etid sa a. Sèlman benefis dirèk ou pral resevwa se pwoteksyon kont moustik mòde. Ou ta dwe tou benefisye endirèkteman nan etid la, paske rezilta li yo pral pi plis kontwole moustik ak deng, chikungunya ak zika viris, amelyore kondisyon lavi yo pou ou, fanmi ou ak peyi a an jeneral, pandan y ap ekonomize lajan ki ta ka depanse nan swen medikal. Vreman vre, rezilta etid sa a ta dwe gen anpil dirèk ak siyifikatif enplikasyon pou politisyen ayisyen, osi byen ke mizisyen politik nan ajans donatè yo.

Syantis ki responsab etid sa a se Pwofesè Christian Raccurt, Dwayen nan fakilte medsinn nan Inivèsite Quisqueya. Komite Bioetik Nasyonal nan Ministè Sante Piblik ak Popilasyon te apwouve etid sa a. Si w gen nenpòt kesyon sou etid sa a, tanpri kontakte Pwofesè Christian Raccurt nan 3764 2584. Pou plis enfòmasyon ou ka toujou kontakte doktè Cyrille Czeher nan 3726 5230. Si ou pa satisfè ak eksplikasyon yo bay yo, ou ka ekri doktè Gerald Lerebours, Prezidan Komite Bioethik Nasyonal la, c/o AMH, 29, Avenue de la Ligue Feminine, 1 Avenue du Travail, Pòtoprens; Telefòn 3701 5766.

Akò konsantman pou chak patisipan nan etid la:

Kòm tèt nan kay mwen, mwen, ki siyen .... Mwen konprann klè objektif pwojè a ki rele **"Emanatè transfluthrine senp, abòdab ak fleksib pou pwoteje kont transmisyon viris Zika, Dengue ak Chikungunya".** Mwen dakò ke moun lakay mwen ap patisipe nan etid la e yo ap sèvi avèk emanatè ke ekip rechèch la ap ba yo. Mwen aksepte tou ke manm fanmiy mwen ap patisipe nan diskisyon ke ekip la ap anrejistre sou aparèy sa yo, e youn nan manm fanmi lan ap pran foto ki montre kouman itilizasyon an fèt. Mwen menm mwen konprann ke ekip rechèch la dwe chita avèk mwen pou jwenn yon konsantman alekri anvan li pibliye nenpòt foto ki te pran nan kay mwen, oswa pou pataje li ak nenpòt moun ki soti andeyò kay la ak ekip rechèch la. Mwen konprann ke mwen ka anile konsantman mwen an epi kite etid la nan nenpòt ki lè.

Non patisipan an nan etid la: ____

Sèks (Gason / Fi): ____________________________ Laj ______________________________

Siyati patisipan an nan etid la: _________________________ Date _____________________

Non Temwen: ____

Siyati Temwen an: _______________________________________ Date ________________

Emanatè transfluthrine senp, abòdab ak fleksib pou pwoteje kont transmisyon viris Zika, Dengue ak Chikungunya

ANEX 7
KESYONÈ POU MONTRE NIVO SATISFAKSYON MOUN KI NAN KAY LA

Nimewo kay la: ______ Non patisipan an: __________________ Sèks (H / F): ___ Dat: ___________

Nimewo serye akò konsantman patisipan an (ICF1) __________________

1. Nan ki nivo ou panse emanatè a pwoteje ou kont marengwen kap mòde w lè ou nan kay la? Fè yon ti wonn youn nan repons ki pi ba yo, oswa sèk nòt ki anba la a : **PA GEN REPONS**

1<br>LI PA PWOTEJE	2<br>TOU PITI	3<br>BYEN	4<br>TRÈ BYEN	5<br>PWOTEGE NÈT

2. Nan ki nivo pou emanatè a pwoteje ou ak lot moun lakay ou kont lòt vèmin yo lè ou nan kay la? Fè yon ti wonn nan youn nan repons ki pi ba yo, oswa sèk nòt ki anba la a : **PA GEN REPONS**

1<br>LI PA PWOTEJE	2<br>TOU PITI	3<br>BYEN	4<br>TRÈ BYEN	5<br>PWOTEGE NÈT

3. Nan ki nivo emanatè a pwoteje ou kont marengwen lè ou deyò nan lakou a ? Fè yon ti wonn youn nan repons ki pi ba yo, oswa sèk nòt ki anba la a : **PA GEN OKENN REPONS**

1<br>LI PA PWOTEJE	2<br>TOU PITI	3<br>BYEN	4<br>TRÈ BYEN	5<br>PWOTEGE NÈT

4. Nan ki nivo emanatè a pwoteje ou ak fanmiy ou kont lòt vèmin yo lè ou deyò kay la ? Fè yon ti wonn youn nan repons ki pi ba yo, oswa sèk nòt ki anba la a : **PA GEN OKENN REPONS**

1<br>LI PA PWOTEJE	2<br>TOU PITI	3<br>BYEN	4<br>TRÈ BYEN	5<br>PWOTEGE NÈT

5. site ak dekri nenpòt lòt benefis oswa dezavantaj ke emanate a bay:

____

____

____

6. Klase enpòtans avantaj / dezavantaj sa a pa youn nan repons ki anba yo, oswa nenpòt nan bagay sa yo: **PA APLIKE** oswa **PA GEN REPONS**.

1<br>GWO DEZAVANTAJ / PA BON MEM	2<br>TI DEZAVANTAJ / PA TWÒ MAL	3<br>DEZAVANTAJ FÈB	4<br>AVANTAJ FÈB	5<br>TI AVANTAJ	6<br>GWO AVANTAJ

7. site ak dekri nenpòt lòt benefis oswa dezavantaj ke emanate a bay:

____

____

____

8. Klase enpòtans avantaj / dezavantaj sa a pa youn nan repons ki anba yo, oswa nenpòt nan bagay sa yo: **PA APLIKE** oswa **PA GEN OKENN REPONS**.

1<br>GWO DEZAVANTAJ / PA BON MEM	2<br>TI DEZAVANTAJ / PA TWÒ MAL	3<br>DEZAVANTAJ FÈB	4<br>AVANTAJ FÈB	5<br>TI AVANTAJ	6<br>GWO AVANTAJ

9. site ak dekri nenpòt lòt benefis oswa dezavantaj ke emanate a bay:

____

____

____

10. Klase enpòtans avantaj / dezavantaj sa a pa youn nan repons ki anba yo, oswa nenpòt nan bagay sa yo: **PA APLIKE** oswa **PA GEN OKENN REPONS**.

1<br>GWO DEZAVANTAJ / PA BON MEM	2<br>TI DEZAVANTAJ / PA TWÒ MAL	3<br>DEZAVANTAJ FÈB	4<br>AVANTAJ FÈB	5<br>TI AVANTAJ	6<br>GWO AVANTAJ

<u>Emanatè transfluthrine senp, abòdab ak fleksib pou pwoteje kont transmisyon viris Zika, Dengue ak Chikungunya</u>

ANEX 8
AKÒ KONSANTMAN POU PATISIPAN NAN GRAN FOKIS GWOUP KI NAN FWAYE YO POU KOUVRI ENFÒMASYON SOU EMANATÈ

NIMEWO SERI AKÒ KONSANTMAN : _________________

Marengwen Aedes egipti gen koulè nwa ak mak blan, epi an jeneral li mòde pandan gwo midi. Isit la an Ayiti, yo transmèt viris deng ak chikungunya, ki lakòz le pli souvan lafyèv, doulè nan kò ak yon kantite lòt sentòm. Anplis de sa, moustik sa a transmèt viris Zika, ki ka lakòz domaj grav lakay ti bebe ki nan vant si fanm ansent lan te enfekte.

Nou envite ou yo patisipe nan yon etid yo evalye yon nouvo zouti yo anpeche mòde soti nan moustik yo. Objektif la nan etid la se yo demontre ke yon fòmilasyon nouvo sou transfluthrin, ensektisid nan menm san danje epi yo souvan itilize nan anwoulman moustik ka bay pwoteksyon abòdab kont moustik sa yo pou omwen 6 mwa. Remake byen ke nan apwòch nouvo nou an, ensektisid natirèlman evapore epi gaye nan lè a osi vapè, pandan y ap anwoulman yo moustik yo dwe boule epi ou toujou ap pwodwi lafimen.

Youn nan eksperyans yo te planifye nan etid sa a enplike nan bay de nan sa yo emanateur nan yon ti kantite konsantman lokatè, pou yo sèvi ak yo pwoteje tèt yo kont moustik pou yon peryòd de 6 mwa. Si oumenm ak tout moun lakay ou dakò pou patisipe nan pati nan etid la, ekip rechèch la pral vizite kay ou yon fwa chak de mwa epi rekipere emanators yo pou apeprè de semèn pou yo mezire degre pwoteksyon yo bay kont moustik mòde nan moman sa a. Apre ou fin pale ak ekip rechèch la sou itilizasyon san danje epi efikas, ou pral lib yo itilize sa yo emèt nan fason ou panse pi bon an ak efikas, osi lontan ke ou pa louvri etajè a pwoteksyon oswa pa sèvi ak li nan nenpòt fason ki ta ka lakòz kontak dirèk fizik ak materyèl la enpreye.

Yo mande w pou reprezante kay ou lè ou patisipe nan yon diskisyon sou aparèy la ak yon gwoup ki gen jiska 8 lòt patisipan yo. Patisipan yo lòt nan diskisyon an gwoup ap soti nan kay yo lòt kote yo te bay egran yo. Yon fasilitatè k ap travay avèk ekip rechèch la ap gide diskisyon an pandan yon obsèvatè pral fè yon anrejistreman odyo epi pran nòt sou sa ki te di. Yo pap anrejistre enfòmasyon pèsonèl yo eksepte non w, sèks ou ak laj, epi detay sa yo ap rete konfidansyèl. Patisipasyon ou ak sa nan kay ou a se antyèman volontè epi ou yo gratis yo kite etid la nan nenpòt ki lè.

Ou pap resevwa konpansasyon pou patisipasyon ou nan etid sa a. Sèlman benefis dirèk ou pral resevwa se pwoteksyon kont moustik mòde. Ou ta dwe tou benefisye endirèkteman nan etid la, paske rezilta li yo pral pi plis kontwole moustik ak deng, chikungunya ak zika viris, amelyore kondisyon lavi yo pou ou, fanmi ou ak peyi a an jeneral, pandan y ap ekonomize lajan ki ta ka

depanse nan swen medikal. Vreman vre, rezilta etid sa a ta dwe gen anpil dirèk ak siyifikatif enplikasyon pou politisyen ayisyen, osi byen ke mizisyen politik nan ajans donatè yo.

Syantis ki responsab etid sa a se Pwofesè Christian Raccurt, Dwayen nan fakilte medsinn nan Invèsite Quisqueya. Komite Bioetik Nasyonal nan Ministè Sante Piblik ak Popilasyon te apwouve etid sa a. Si w gen nenpòt kesyon sou etid sa a, tanpri kontakte Pwofesè Christian Raccurt nan 3764 2584. Pou plis enfòmasyon ou ka toujou kontakte doktè Cyrille Czeher nan 3726 5230. Si ou pa satisfè ak eksplikasyon yo bay yo, ou ka ekri doktè Gerald Lerebours, Prezidan Komite Bioethik Nasyonal la, c/o AMH, 29, Avenue de la Ligue Feminine ci-devant 1 Avenue du Travail, Pòtoprens; Telefòn 3701 5766.

Akò konsantman pou chak patisipan nan etid la:

Kòm reprezantan nan kay mwen an, mwen, ki siyen .... Mwen konprann klè objektif pwojè a ki rele **"Emanatè transfluthrine senp, abòdab ak fleksib pou pwoteje kont transmisyon viris Zika, Dengue ak Chikungunya".** Mwen dakò ke kay mwen an ap patisipe nan etid la pandan li sèvi avèk emanatè yo ke ekip rechèch la ba li. Mwen dakò tou pou yo patisipe nan diskisyon ki ap anrejistre sou aparèy sa yo. Mwen konprann ke mwen ka anile konsantman mwen epi kite etid la nan nenpòt ki lè.

Non patisipan an nan etid la: ____

Sèks (Gason / Fi): ___________________________ Laj _____________________________

Siyati patisipan an nan etid la: _________________________ Date _____________________

Non Temwen: ____

Siyati Temwen an: _______________________________________ Date ________________

Emanatè transfluthrine senp, abòdab ak fleksib pou pwoteje kont transmisyon viris Zika, Dengue ak Chikungunya

ANEX 9
GID TEMATIC POU DISKIZYON GROUP YO

1. Pèsepsyon ki te egziste deja sou marengwen ak mwayen pou nou evite

    1a. Pwoblèm oswa benefis ki asosye avèk marengwen.

    1b. Mezi anjeneral nou konn pran pou evite marengwen mòde nou oswa pou nou touye yo.

    1c. Nivo pwoteksyon mwayen kite la a deja yo.

2. Pèsepsyon nan avantaj ak dezavantaj emanatè a

    2a. Pwoteksyon kont marengwen kap mòde moun.

    2b. Lòt benefis.

    2c. Pa gen gwo pwoteksyon.

    2d. Lòt dezavantaj.

    2e. Lòt faktè ki enfliyanse itilizasyon yo.

3. Pratik pou itilize emanatè a

    3a. Bon pratik nan itilizasyon li.

    3b. Move pratik nan itilizasyon li.

    3c. Limit ak dezavantaj.

    3d. Kijan pou amelyore fason emanatè a fonksyone.

    3e. Lide pou distribisyon ak antretyen aparèy la.

Emanatè transfluthrine senp, abòdab ak fleksib pou pwoteje kont transmisyon viris Zika, Dengue ak Chikungunya

ANEX 10

AKO KONSANTMAN POU PATISIPAN NAN KAY LA PA FE EGZESIS FOTOVOYS

NIMEWO SERI AKÒ KONSANTMAN : _________________

Marengwen Aedes egipti gen koulè nwa ak mak blan, epi an jeneral li mòde pandan gwo midi. Isit la an Ayiti, yo transmèt viris deng ak chikungunya, ki lakòz le pli souvan lafyèv, doulè nan kò ak yon kantite lòt sentòm. Anplis de sa, moustik sa a transmèt viris Zika, ki ka lakòz domaj grav lakay ti bebe ki nan vant si fanm ansent lan te enfekte.

Nou envite ou yo patisipe nan yon etid yo evalye yon nouvo zouti yo anpeche mòde soti nan moustik yo. Objektif la nan etid la se yo demontre ke yon fòmilasyon nouvo sou transfluthrin, ensektisid nan menm san danje epi yo souvan itilize nan anwoulman moustik ka bay pwoteksyon abòdab kont moustik sa yo pou omwen 6 mwa. Remake byen ke nan apwòch nouvo nou an, ensektisid natirèlman evapore epi gaye nan lè a osi vapè, pandan y ap anwoulman yo moustik yo dwe boule epi ou toujou ap pwodwi lafimen.

Youn nan eksperyans yo te planifye nan etid sa a enplike nan bay de nan sa yo emanateur nan yon ti kantite konsantman lokatè, pou yo sèvi ak yo pwoteje tèt yo kont moustik pou yon peryòd de 6 mwa. Si oumenm ak tout moun lakay ou dakò pou patisipe nan pati nan etid la, ekip rechèch la pral vizite kay ou yon fwa chak de mwa epi rekipere emanators yo pou apeprè de semèn pou yo mezire degre pwoteksyon yo bay kont moustik mòde nan moman sa a. Apre ou fin pale ak ekip rechèch la sou itilizasyon san danje epi efikas, ou pral lib yo itilize sa yo emèt nan fason ou panse pi bon an ak efikas, osi lontan ke ou pa louvri etajè a pwoteksyon oswa pa sèvi ak li nan nenpòt fason ki ta ka lakòz kontak dirèk fizik ak materyèl la enpreye.

Kòm yon reprezantan lakay ou, nou mande ou pou w anrejistre sa ki lakay ou an pèfòmans kòm fason ki pi ak pi piti efikas pou itilize aparèy sa yo, lè l sèvi avèk kamera jetab ke ekip rechèch la ap founi. Anvan ou sèvi ak kamera a, ou pral patisipe nan yon sesyon fòmasyon kout ki pral eksplike kouman yo sèvi ak kamera a nan yon fason ki akseptab, san yo pa konpwomèt sekirite, sou vi prive ak lòt dwa moun ki abite nan kay la oswa vwazen yo. Nan kòmansman chak vizit nan kay la, yon fwa chak de mwa, nenpòt ki kamera konplètman-itilize yo pral kolekte pa ekip la rechèch yo ki pèmèt devlopman nan foto yo nan de kopi. Y ap bay yon kopi apre yon kèk jou, lòt la pral kenbe konfidansyèlman pa ekip rechèch la kòm doub nan ka ta gen pèt. Nou mande ou tou pou patisipe nan yon reyinyon ak fotogwaf soti nan lòt kay nan katye ou, yo nan lòd yo chwazi foto ki pi enfòmatif ak diskite sou siyifikasyon yo. Yon fasilitatè k ap travay avèk ekip rechèch la ap gide diskisyon an pandan yon obsèvatè pral fè yon anrejistreman odyo epi pran nòt sou sa ki te di. Yo pap anrejistre enfòmasyon pèsonèl yo eksepte non w, sèks ou ak laj, epi detay sa yo ap rete konfidansyèl. Patisipasyon ou ak sa nan kay ou a se antyèman volontè epi ou yo gratis yo kite etid la nan nenpòt ki lè.

Ou pap resevwa konpansasyon pou patisipasyon ou nan etid sa a. Sèlman benefis dirèk ou pral resevwa se pwoteksyon kont moustik mòde. Ou ta dwe tou benefisye endirèkteman nan etid la, paske rezilta li yo pral pi plis kontwole moustik ak deng, chikungunya ak zika viris, amelyore kondisyon lavi yo pou ou, fanmi ou ak peyi a an jeneral, pandan y ap ekonomize lajan ki ta ka depanse nan swen medikal. Vreman vre, rezilta etid sa a ta dwe gen anpil dirèk ak siyifikatif enplikasyon pou politisyen ayisyen, osi byen ke mizisyen politik nan ajans donatè yo.

Syantis ki responsab etid sa a se Pwofesè Christian Raccurt, Dwayen nan fakilte medsinn nan Inivèsite Quisqueya. Komite Bioetik Nasyonal nan Ministè Sante Piblik ak Popilasyon te apwouve etid sa a. Si w gen nenpòt kesyon sou etid sa a, tanpri kontakte Pwofesè Christian Raccurt nan 3764 2584.

Pou plis enfòmasyon ou ka toujou kontakte doktè Cyrille Czeher nan 3726 5230. Si ou pa satisfè ak eksplikasyon yo bay yo, ou ka ekri doktè Gerald Lerebours, Prezidan Komite Bioethik Nasyonal la, c/o AMH, 29, Avenue de la Ligue Feminine, 1 Avenue du Travail, Pòtoprens; Telefòn 3701 5766.

Akò konsantman pou chak patisipan nan etid la:

Kòm reprezantan nan kay mwen an, mwen, ki siyen .... Mwen konprann klè objektif pwojè a ki rele **"Emanatè transfluthrine senp, abòdab ak fleksib pou pwoteje kont transmisyon viris Zika, Dengue ak Chikungunya".** Mwen dakò pou sèvi ak kamera pou anrejistre kijan emanatè yo te itilize, epi pou patisipe nan diskisyon sou anrejistreman aparèy sa yo. Mwen konprann ke mwen ka anile konsantman mwen epi kite etid la nan nenpòt ki lè.

Non patisipan an nan etid la: ____

Sèks (Gason / Fi): ____________________________ Laj ______________________________

Siyati patisipan an nan etid la: _________________________ Date _____________________

Non Temwen: ____

Siyati Temwen an: _______________________________________ Date ________________

Institut de Recherche pour le Développement FRANCE — CENTERS FOR DISEASE CONTROL AND PREVENTION — Université Quisqueya Hominis beneficio cognoscere et agere — LIVERPOOL SCHOOL OF TROPICAL MEDICINE

Emanatè transfluthrine senp, abòdab ak fleksib pou pwoteje kont transmisyon viris Zika, Dengue ak Chikungunya

ANEX 11
GID TEMATIC POU DISKIZYON GROUP SOUS EGZÈSIS PHOTOVOYS

1. Prezante imaj yo / foto yo epi mande patisipan yo eksplike siyifikasyon yo ak enpòtans yo.

2. Jwenn opinyon yo pandan yap eksplike kouman foto yo montre avantaj oswa dezavantaj nan sèvi ak aparèy la.

  2a. Pwoteksyon kont marengwen kap mòde moun.

  2b. Lòt benefis.

  2c. Limit nan pwoteksyon an.

  2d. Lòt dezavantaj.

  2e. Lòt faktè ki enfliyanse itilizasyon yo.

3. Pran esplikasyon sou kijan foto yo montre itilizasyon emanate a ak lide pou amelyore randman aparèy yo, distribisyon yo oswa antretyen yo, pi devan.

  3a. Bon pratik nan itilizasyon.

  3b. Move pratik nan itilizasyon.

  3c. Limit ak dezavantaj.

  3d. Lide pou amelyore aparèy la li menm.

  3e. Lide pou distribisyon an ak antretyen aparèy la.

<u>Emanatè transfluthrine senp, abòdab ak fleksib pou pwoteje kont transmisyon viris Zika, Dengue ak Chikungunya</u>

ANEX 12
FÒM KONSANTMAN ENFÒMASYON POU PIBLIKASYON YON PHOTO ESPESYAL

NIMEWO SERI /KONSANTMAN ENFÒMASYON (ICF1): ____________________

NIMEWO KAY LA: ________________

Jan ou te dokimante deja alekri (ICF1 ___________), ou menm ansanm ak tout moun lakay ou te dakò pou patisipe nan yon pwojè rechèch pou evalye yon nouvo aparèy emanatè pou pwoteksyon kont marengwen ka mòde moun. Tanpri gade fèy enfòmasyon patisipan an pou plis detay. Yon manm nan kay la tou te dakò pou pran foto pou montre kijan oumenm ak lakay ou itilize aparèy sa yo, nan lide pou amelyore konsepsyon, distribisyon ak antretyen yo. Ekip rechèch la kounye a ta renmen pibliye oswa pataje foto ke yo te pran nan kay ou a, enteryè li yo ak / oswa okipan, donk yap mande ou pèmisyon alekri yo ka fè sa. Nou mande tou pèmisyon alekri ak nenpòt ki moun ki vizib sou foto a pou pibliye oswa pataje li, e nou ta rekonesan si ou ede nou idantifye ak kontakte moun sa yo pou jwenn konsantman alekri yo sou menm dokiman an. Piblikasyon foto sa a dwe kache tout enfòmasyon pèsonèl, tankou karakteristik feminen, oswa sou kay ou, oswa efè pèsonèl ou .Jiskaske ou satisfè e pèmèt itilizasyon foto a . Patisipasyon ou nan etid sa a se antyèman volontè epi ou lib pou refize piblikasyon foto sa a.

Ou pap resevwa konpansasyon pou patisipasyon ou nan etid sa a. Sèlman benefis dirèk ou pral resevwa se pwoteksyon kont moustik mòde. Ou ta dwe tou benefisye endirèkteman nan etid la, paske rezilta li yo pral pi efektivman kontwole moustik ak deng, chikungunya ak zika viris. Sa yo alontèm endirèk benefis ta amelyore kondisyon lavi pou ou, fanmi ou ak peyi a an jeneral, pandan y ap ekonomize lajan ki ta dwe depanse nan swen medikal. Vreman vre, rezilta etid sa a ta dwe gen anpil dirèk ak siyifikatif enplikasyon pou politisyen ayisyen, osi byen ke mizisyen politik nan ajans donatè yo.

Syantis ki responsab etid sa a se Pwofesè Christian Raccurt, Dwayen nan fakilte medsinn nan Inivèsite Quisqueya. Komite Bioetik Nasyonal nan Ministè Sante Piblik ak Popilasyon te apwouve etid sa a. Si w gen nenpòt kesyon sou etid sa a, tanpri kontakte Pwofesè Christian Raccurt nan 3764 2584.

Pou plis enfòmasyon ou ka toujou kontakte doktè Cyrille Czeher nan 3726 5230. Si ou pa satisfè ak eksplikasyon yo bay yo, ou ka ekri doktè Gerald Lerebours, Prezidan Komite Bioethik Nasyonal la, c/o AMH, 29, Avenue de la Ligue Feminine ci-devant 1 Avenue du Travail, Pòtoprens; Telefòn 3701 5766.

ESPAS POU METE FOTO W LA

Akò konsantman nan tèt la nan kay la

Kòm tèt nan kay nou an, mwen, ki siyen .... byen konprann objektif pwojè a ki gen tit "**Emanatè transfluthrine senp, abòdab ak fleksib pou pwoteje kont transmisyon viris Zika, Dengue ak Chikungunya**", epi mwen aksepte ke foto ki anwo la a, ki te pran nan kay la oswa nan kay la, ka pibliye oswa pataje gratis nan fòm egzat la prezante anwo a.

Non patisipan an nan etid la: ____

Sèks (Gason / Fanm): ___________________________ Laj: _________________________

Siyati patisipan an nan etid la: _________________________Date __________________

Non Temwen: ____

Siyati Temwen an: _______________________________________Date_______________

Akò konsantman moun ki prezan nan foto a

Kòm moun nan nan foto a ki pi wo a, oswa paran / gadyen legal, Avèk prezant sa mwen .... byen klè konprann objektif pwojè ki rele "**Emanatè transfluthrine senp, abòdab ak fleksib pou pwoteje kont transmisyon viris Zika, Dengue ak Chikungunya**", epi mwen dakò ki ka foto a dwe pibliye oswa pataje lib nan fòm lan egzak prezante pi wo a.

Non patisipan an nan etid la: ____

Sèks (Gason / Fanm): ___________________________ Laj: _________________________

Siyati patisipan an nan etid la: _________________________Date __________________

Non Temwen: ____

Siyati Temwen an: _______________________________________Date_______________

Kòm moun nan nan foto a ki pi wo a, oswa paran / gadyen legal, Avèk prezant sa mwen .... byen klè konprann objektif pwojè ki rele "**Emanatè transfluthrine senp, abòdab ak fleksib pou pwoteje kont transmisyon viris Zika, Dengue ak Chikungunya**", epi mwen dakò ki ka foto a dwe pibliye oswa pataje lib nan fòm lan egzak prezante pi wo a.

Non patisipan an nan etid la: ____

Sèks (Gason / Fanm): ___________________________ Laj: _________________________

Siyati patisipan an nan etid la: _________________________Date ___________________

Non Temwen: ____

Siyati Temwen an: _______________________________________Date________________

Kòm moun nan nan foto a ki pi wo a, oswa paran / gadyen legal, Avèk prezant sa mwen .... byen klè konprann objektif pwojè ki rele "**Emanatè transfluthrine senp, abòdab ak fleksib pou pwoteje kont transmisyon viris Zika, Dengue ak Chikungunya**", epi mwen dakò ki ka foto a dwe pibliye oswa pataje lib nan fòm lan egzak prezante pi wo a.

Non patisipan an nan etid la: ____

Sèks (Gason / Fanm): ___________________________ Laj: _________________________

Siyati patisipan an nan etid la: _________________________Date ___________________

Non Temwen: ____

Siyati Temwen an: _______________________________________Date________________

Kòm moun nan nan foto a ki pi wo a, oswa paran / gadyen legal, Avèk prezant sa mwen .... byen klè konprann objektif pwojè ki rele "**Emanatè transfluthrine senp, abòdab ak fleksib pou pwoteje kont transmisyon viris Zika, Dengue ak Chikungunya**", epi mwen dakò ki ka foto a dwe pibliye oswa pataje lib nan fòm lan egzak prezante pi wo a.

Non patisipan an nan etid la: ____

Sèks (Gason / Fanm): ___________________________ Laj: _________________________

Siyati patisipan an nan etid la: _________________________Date ___________________

Non Temwen: ____

Siyati Temwen an: _______________________________________Date________________
